# Chalcogen‐Guided Control of Azoarene Photoswitching: Tuning Excited‐State Energies Through Electronic Property Modulation

**DOI:** 10.1002/chem.202501571

**Published:** 2025-07-22

**Authors:** Zoe Nonie Scheller, Jan Schulte, Christoph Wölper, Gebhard Haberhauer

**Affiliations:** ^1^ Institut für Organische Chemie Universität Duisburg‐Essen Universitätsstr. 7 D‐45117 Essen Germany

**Keywords:** azo compounds, chalcogen bonding, excited state, molecular switches, three‐electron σ bond

## Abstract

In recent years, chalcogen bonding has emerged as a promising alternative to classical supramolecular interactions such as hydrogen or halogen bonds. While its behavior in the electronic ground state has been extensively studied, its role in the excited state is gaining increasing attention. We recently demonstrated that the lack of photoswitchability of *ortho*‐tellurated azobenzenes is due to an excitation‐induced conversion of the classical chalcogen bond into a more pronounced, electron‐rich three‐electron σ bond. This transformation significantly strengthens the interaction between the chalcogen and the Lewis base center, effectively preventing isomerization. Based on these findings, we have now investigated the photoswitching behavior of *ortho*‐tellurium‐substituted azoarenes by modulation of the electronic properties of the aryl substituent and the oxidation state of the tellurium center. Our results show that electron‐donating groups destabilize the excited‐state geometry associated with the formation of a three‐electron σ bond, thereby restoring photoisomerizability. Furthermore, oxidation to the Te(IV) species disrupts this bonding interaction, leading to significantly enhanced photoswitching properties. Together, these findings provide valuable design principles for the development of multiresponsive molecular switches based on chalcogen bonding and excited‐state control.

## Graphical abstract

Photophysical properties of *ortho*‐tellurium‐substituted azoarenes are tunable via aryl substituent electronics and tellurium oxidation state. Electron‐donating groups destabilize the excited state three‐electron σ bond, enhancing photoisomerization. Oxidation removes the tellurium lone pair required for the described interaction, further boosting switching.

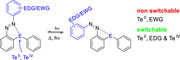

## 1 Introduction

Chalcogen bonds are σ‐hole interactions in which electron density is shifted from one or two^[^ ^1^ ^]^ Lewis base centers to the antibonding σ* orbital of a covalent chalcogen bond.^[^ ^2^, ^3^, ^4^, ^5^ ^]^ Systematic investigations have shown that chalcogen bond strength increases with the atomic weight of the chalcogen and the electron‐withdrawing nature of its substituents.^[^ ^6^, ^7^, ^8^ ^]^ To date, the majority of research on chalcogen interactions has been conducted in the solid state, where a large number of binding modes have been described.^[^ ^9^, ^10^, ^11^, ^12^, ^13^, ^14^, ^15^, ^16^, ^17^, ^18^, ^19^, ^20^ ^]^ This research has contributed to the development of numerous catalysts based on chalcogen interactions, which find application in a variety of areas.^[^ ^21^, ^22^, ^23^, ^24^, ^25^, ^26^, ^27^, ^28^, ^29^, ^30^ ^]^ Recently, the first supramolecular applications of chalcogen‐containing systems have also been described.^[^ ^31^, ^32^, ^33^, ^34^, ^35^, ^36^, ^37^, ^38^, ^39^, ^40^, ^41^, ^42^, ^43^ ^]^

Azoarenes can be switched from the stretched *trans* form to the compact, metastable *cis* form by means of light. The reverse process can be achieved either photochemically or thermally. These molecules are among the most frequently used light‐induced switching devices due to their ease of synthesis, facile functionalization, high switching reversibility, and excellent photostability.^[^ ^44^, ^45^, ^46^, ^47^, ^48^, ^49^, ^50^ ^]^ Consequently, azoarenes are used for a variety of applications.^[^ ^51^, ^52^, ^53^, ^54^, ^55^, ^56^, ^57^, ^58^ ^]^ By incorporating azobenzene into a rigid cyclic structure, for example, it becomes possible to convert the excitation energy into ring strain.^[^ ^59^, ^60^ ^]^ Currently, most studies focus on modulating supramolecular systems through azoarene photoswitching. However, there is also the possibility of influencing the photophysical properties of azoarenes by supramolecular interactions such as hydrogen bonds.^[^ ^61^, ^62^, ^63^, ^64^, ^65^ ^]^ In addition to hydrogen bonds, it has recently been demonstrated that chalcogen bonds also significantly impact the switching behavior of azobenzenes.^[^ ^66^, ^67^ ^]^ For instance, the switching ability of azobenzenes with a heavy chalcogen atom in the *ortho* position can be activated and deactivated through redox chemistry.^[^ ^66^ ^]^ Initially, this behavior was attributed to the energy difference between the most stable *trans* and *cis* isomers in the ground state. Given the significant discrepancy in the isomers of the divalent tellurium derivative, it was hypothesized that switching would be suppressed.^[^ ^66^ ^]^ However, a more thorough investigation through quantum chemical calculations revealed that this phenomenon occurs due to the excited S_1_ states.^[^ ^68^ ^]^ The non‐covalent chalcogen bond is converted into a covalent three‐electron σ bond upon excitation. This bond is particularly pronounced in the tellurium derivative, thereby suppressing switching via excited‐state rotation. In this study, we present a series of tellurium‐substituted azoarenes, which are assembled from a tellurium‐substituted phenyl moiety and a second aryl group, the latter exhibiting electron‐withdrawing or electron‐donating properties. In addition to the divalent tellurium compounds, the corresponding dichlorides and dibromides were also characterized. Our investigation demonstrates that incorporating electron‐donating aromatic groups (EDG) enables photoisomerization in divalent tellurium compounds.

## 2 Results and Discussion

### 2.1 Concept

**Figure 1:**
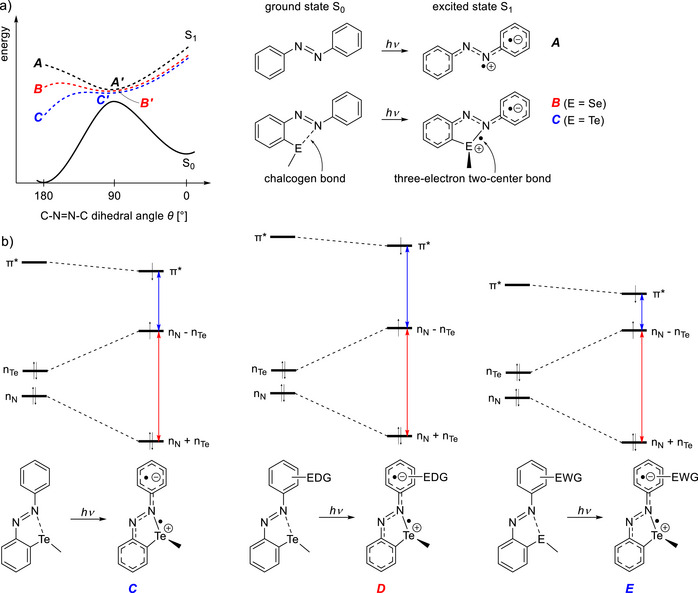
a) Potential energy profile of azobenzene (***A***) and chalcogen‐substituted azobenzenes (***B*** and ***C***) in the singlet ground (S_0_, solid) and excited states (S_1_, dashed). Upon excitation, the non‐covalent chalcogen bond present in the ground state of the *trans* isomers converts into a covalent three‐electron σ bond in the excited state, resulting in stabilization of ***B*** and ***C*** relative to their non‐bonded counterparts ***B′*** and ***C′***. b) Change in orbital energies for tellurium‐substituted azobenzenes upon light excitation. The splitting of the (n_N_ + n_Te_) and (n_N_ – n_Te_) orbitals (red arrows), caused by the formation of a covalent three‐electron σ bond, is hardly affected by the substituent on the second ring. In contrast, the energy difference between the (n_N_ – n_Te_) and π* orbitals (blue arrows) is strongly influenced by the nature of the substituent.

Recently, we demonstrated that the non‐switchability of azobenzenes with a divalent tellurium substituent in the *ortho* position to the azo group can be attributed to the energy profile of the excited state S_1_.^[^ ^68^ ^]^ In unsubstituted azobenzene, the S_1_ excited state energy at a dihedral angle *θ* of 180° (state ***A***) is higher than at 90° (state ***A'***; Figure 1a). This results in a rotation around the N–N bond in the S_1_ state, which allows the system to reach its energetic minimum at approximately 90°. This facilitates relaxation to the ground state in either the *trans* or *cis* form. In contrast, *ortho*‐chalcogen‐substituted azobenzenes display significantly different S_1_ energy profiles.^[^ ^68^ ^]^ In this case, a change in bond type can be observed upon excitation. In the ground state, a classic non‐covalent chalcogen bond is present, in which the Lewis base shifts electron density into the σ* orbital of the chalcogen center (Figure 1a). Upon n→π* excitation, an electron is lifted from the n_E_ orbital into the π* orbital. As a result of this excitation, the chalcogen atom (e.g., Te) now has a positive formal charge and a single electron in an n_Te_ orbital. Rotation around the C–E bond enables the interaction between the singly occupied n_Te_ orbital and the n_N_ orbital (Figure 1a). The singly occupied (n_N_ – n_Te_) orbital increases in energy, while the doubly occupied (n_N_ + n_Te_) orbital decreases (Figure 1b). This two‐center three‐electron interaction can be supported by the occupation of the active orbitals according to the CASSCF(12,10) calculation of the S_1_ state (Figure S70) and a natural bond orbital analysis of the triplet state at 180°:^[^ ^69^ ^]^ the Te–N σ and σ* orbitals were found only in the beta spin orbitals (Figure S70). The covalent three‐electron σ bond^[^ ^70^ ^]^ stabilizes the states ***B*** and ***C*** (*θ* = 180°) having a positive charge in the σ system and a negative charge in the perpendicular π system compared to the states ***B'*** and ***C'*** (*θ* = 90°) in which two radical π systems are perpendicular to each other (Figures 1a and S67). For the tellurium derivative, state ***C*** is significantly lower in energy than ***C′***. As a result, no rotation occurs in the excited state, and photoisomerization is suppressed. In the case of the selenium derivative, ***B*** and ***B'*** are energetically close and separated by a low energy barrier. Accordingly, rotation can occur in the excited state, which is promoted by an increase in temperature. Consequently, temperature‐dependent isomerization can be observed.

Given our strong interest in the role of electronic stabilization in modulating excited state rotations,^[^ ^71^, ^72^, ^73^, ^74^ ^]^ we aimed to restore the rotation of azoarenes bearing divalent tellurium substituents in the *ortho* position to the azo group. In those systems, the rotation is usually prevented by the formation of a covalent three‐electron σ bond. To this end, we investigated the effect of suitable substituents on the second aryl ring, aiming to reenable rotation around the N─N bond in the excited state despite the presence of a three‐electron σ bond interaction.

The central objective was to destabilize excited‐state conformation ***C***, characterized by a dihedral angle of 180° (see Figure 1b). A straightforward approach to achieve this involves introducing electron‐donating (EDG, ***D***) or electron‐withdrawing groups (EWG, ***E***) as the second aryl unit. These substituents are spatially remote from the chalcogen center and have a significant impact on the π system. Notably, the (n_N_ + n_Te_) and (n_N_ – n_Te_) orbitals, which are crucial for the covalent three‐electron σ bond, are perpendicular to the π system. Consequently, the substituents exert only minimal influence on the stabilization of these orbitals (see red arrows in Figure 1b). In contrast, the π* orbital of the azo group, into which the n_Te_ electron is promoted upon excitation, is strongly affected: EDGs destabilize the negative charge in the π system (***D***; π* orbital is raised in energy), while EWGs stabilize the negative charge (***E***; π* orbital is lowered). As a result, the energy difference between (n_N_ – n_Te_) and π* is higher in the presence of EDGs and lower in the presence of EWGs (blue arrows in Figure 1b). As EDGs destabilize the 180° conformation (***D***; Figure S67), it is expected that rotation and restoration of photoswitching behavior analogous to the selenium compound (***B***) is possible when using EDGs.

### 2.2 Synthesis and Crystal Structures

To investigate the influence of the electronic properties of suitable second aromatic moieties on the photoswitchability of *ortho*‐tellurated azoarenes, we selected a series of nitrogen‐containing heteroaromatics (**1b**–**d** and **1f**), as well as the *o*,*p*‐dimethoxy compound **1e,** alongside the reference compound **1a**. In addition, the corresponding tellurium(IV) derivatives **2** and **3** were included to further examine the role of oxidation state in modulating photoswitchability, as previously reported.^[^ ^66^ ^]^ Since we already described the temperature dependence in *ortho*‐selenium‐substituted systems such as **4**,^[^ ^68^ ^]^ we also included the selenium‐containing azobenzene **5** as a reference system.

**Figure 2:**
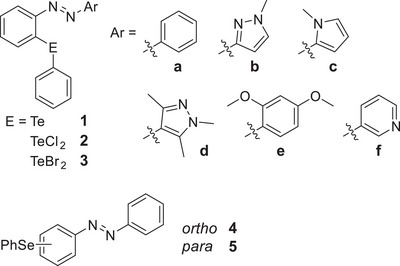
Investigated tellurium‐containing azoarenes **1**–**3** and selenium‐containing azobenzenes **4** and **5**.

**Scheme 1:**
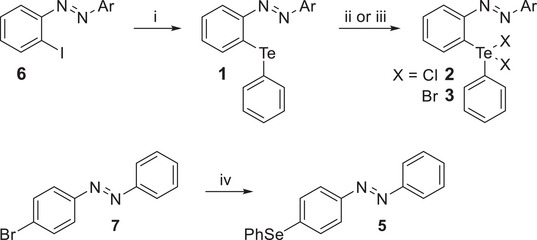
Synthesis of the *ortho*‐tellurium substituted azoarenes **1**–**3** and the selenium‐containing compound **5** (for the definition of Ar see Figure 2). Reaction conditions: i) *n*‐BuLi, Ph_2_Te_2_, diethyl ether (**1a**: 48%; **1b**: 6%, **1c**: 52%, **1d**: 16%, **1e**: 21%, **1f**: 15%). ii) SO_2_Cl_2_, dichloromethane (>99%). iii) Br_2_, diethyl ether (**3a**: 78%; **3b**: 71%, **3c**: 77%, **3d**: 84%, **3e**: 82%, **3f**: 70%). iv) KO*t*‐Bu, Ph_2_Se_2_, dimethyl sulfoxide, 47%.

The halogenated azoarenes were prepared according to known procedures via the corresponding diazonium salts (**6c**–**6e** ^[^ ^75^ ^]^) or the condensation of nitroso with amine compounds (**6a**, **6b**,^[^ ^76^ ^]^ and **6f** ^[^ ^77^ ^]^). The *ortho*‐tellurium‐substituted compounds are prepared by lithium‐halogen exchange with *n*‐BuLi and the addition of diphenyl ditelluride (Scheme 1).^[^ ^66^ ^]^ The oxidation to dichlorides **2** and dibromides **3**, respectively, was achieved using sulfuryl chloride/bromine as an oxidizing agent (Scheme 1).^[^ ^78^ ^]^ The success of the oxidation was confirmed by the characteristic downfield shift in the ^125^Te NMR relative to the Te(II) compound **1** and from the crystal structure data (Figures S75–S87). Compound **4** was synthesized according to literature known procedures.^[^ ^66^ ^]^ For the selenium azobenzene **5**, compound **7** was reacted with diphenyl diselenide and potassium *tert‐*butanolate (Scheme 1).^[^ ^79^ ^]^

**Figure 3:**
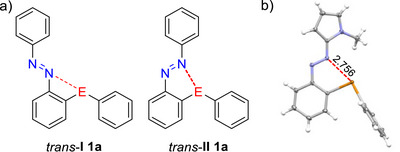
a) Stereoisomeric structures *trans*‐**I** and *trans*‐**II** of **1a** exhibiting two different chalcogen bonds. b) Solid state structure of **1c**. The Te···N interaction is visualized as a red dashed line. The distance is given in angstroms.

With the exception of compounds **1b**, **1f**, **2a**, **3b**, and **5**, single crystals were obtained for all newly synthesized systems, which were then analyzed using X‐ray crystallography. The solid‐state structure of compound **1a** has already been reported in the literature.^[^ ^66^ ^]^ For compounds **1**–**3**, several similarities and differences in the crystal packing can be observed, which will be briefly discussed below. In general, the interaction between the tellurium center and the azo bridge leads to the formation of either a four‐membered ring (*trans*‐**I**; Figure 3a) or a five‐membered ring (*trans*‐**II**), which are stabilized by different chalcogen bonds. Quantum chemical calculations indicated that as the chalcogen atom increases in size, and consequently bond strength, isomer *trans*‐**II** becomes the favored conformation.^[^ ^66^ ^]^ The tellurium‐containing azoarenes **1a** and **1c**–**e** all adopt this conformation in the solid state, with distance d(Te···N) between the tellurium center and the nitrogen atom ranging from 2.67 to 2.76 Å. DFT calculations at the B3LYP‐D3BJ/def2‐TVZP, aug‐cc‐pVTZ‐PP level reveal an energy difference between the two conformers *trans*‐**I** and *trans*‐**II**, for the ground‐state systems **1**, ranging from 2.1 kcal mol^−1^ (**1d**) to 4.9 kcal mol^−1^ (**1e**, Table S1). In all cases, the 5‐membered ring (*trans*‐**II**) is the more stable isomer, as confirmed by the solid state structures. The d(Te···N) distances obtained from quantum chemical calculations (2.65–2.78 Å) are in good agreement with the experimental data. Figure 3b shows the molecular structure of the pyrrole derivative **1c** in the solid state as an example. Aside from the intramolecular Te···N interactions, no further specific supramolecular interactions were observed in the crystal packing of **1**.

Upon oxidation of the tellurium center in the divalent tellurium compounds **1** to Te(IV)Cl_2_ (**2**) or Te(IV)Br_2_ (**3**), the preferred stereoisomer for compounds **2b**–**d**, **3a**, and **3c**–**d** shifts to *trans*‐**I**, accompanied by an increase in the Te···N distance to 2.90–2.94 Å. These distances are again in good agreement with the quantum chemical calculations (B3LYP‐D3BJ/def2‐TVZP, aug‐cc‐pVTZ‐PP), which yield values of 2.91–2.96 Å. Notably, the calculations indicate that the five‐membered ring (*trans*‐**II**) remains energetically preferred for both **2** and **3**, regardless of the aryl substituent (Figure 3a). However, the calculated energy differences between the *trans*‐**I** and *trans*‐**II** isomers, except for **2e** and **3e**, are small (0.4–1.5 kcal mol^−1^; Table S1) and are therefore within the error limits for DFT calculations.

**Figure 4:**
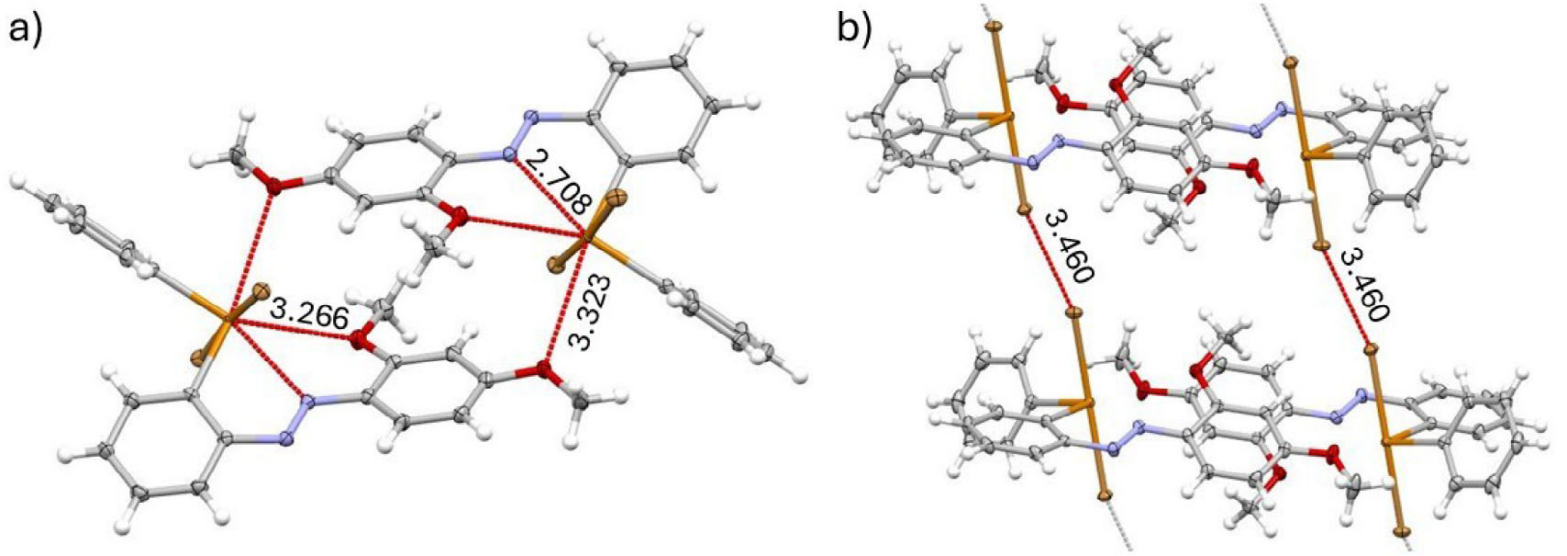
Solid state structure of **3e**. a) Inter‐ and intramolecular interactions of the tellurium center. b) Intermolecular interactions of bromine atoms. Interactions with distances shorter than the sum of the van der Waals radii are depicted as red dashed lines. Distances are given in angstroms.

A wide variety of secondary interactions is observed in the solid‐state structures of compounds **2** and **3**, where both σ holes of the tellurium center are engaged. Derivatives **2d**, **3d**, **2f**, and **3f** form dimeric structures in which the lone pair on the nitrogen atom of the aryl ring shifts electron density into the σ* orbital of the Te–aryl bond (Figure S71).^[^ ^14^, ^15^, ^38^ ^]^ Similarly, the solid state structures of **2e** and **3e** also exhibit dimers, mediated by short Te···O contacts (d(Te···O) = 3.32 Å; Figure 4a). In these cases, the σ* orbital of the Te─Ph bond participates in two intramolecular interactions: one with the lone pair of the azo nitrogen, resulting in the formation of a five‐membered ring and the other with the lone pair of the oxygen atom of the methoxy group in *ortho* position (Figure 4a). The intramolecular distances d(Te···N) = 2.71 Å and d(Te···O) = 3.27 Å are both significantly shorter than the sum of the corresponding van der Waals radii (3.61 Å for Te···N and 3.58 Å for Te···O), indicating attractive interactions. Similar bifurcated chalcogen bonding involving a single σ hole has also been observed in dimers of benzotellurazoles.^[^ ^1^ ^]^ This dual coordination is likely the reason why, unlike **2b**–**d**, **3a**, and **3c**–**d**, the *trans*‐**II** stereoisomer remains favored for **2e** and **3e** after oxidation. The additional stabilization provided by the interaction with the oxygen of the methoxy group is also reflected in the calculated energy differences between the five‐membered and four‐membered ring conformations. For **2e** and **3e**, the *trans*‐**II** isomer is approximately 3 kcal mol^−1^ more stable than the corresponding four‐membered isomer (*trans*‐**I**, Table S1). Additionally, the dimers of **3e** are linked by halogen‐halogen interactions (Figure 4b).

For **2b** and **3c**, quadratic chalcogen–halogen interactions characteristic of trigonal bipyramidal tellurium dihalides are observed (Figure S72).^[^ ^20^, ^80^, ^81^, ^82^ ^]^ In **3c**, dimeric structures are formed featuring two Te···Br interactions of equal length, while in **2b**, ribbon‐like assemblies are present, with one Te···Cl contact slightly exceeding the sum of the van der Waals radii.

### 2.3 Photoswitching Studies

In the next step, we characterized the divalent tellurium compounds **1** regarding their photophysical properties at room temperature (25 °C). We carried out switching experiments via UV/Vis (CH_2_Cl_2_, c = 1 mm, Figures S1–S6) and NMR spectroscopy (CD_2_Cl_2_, c = 5 mm, Figures S21–S26) and determined the thermal *cis* half‐lives at room temperature (Figures S45–S46). For the switching experiments, a spectrum of the initial state was recorded prior to irradiation. The sample was then irradiated with light of wavelength λ = 365 nm for 60 s (NMR) or 5 s (UV), followed by the acquisition of a second spectrum. To ensure that the photo stationary state (PSS) had been reached, irradiation was repeated until no further spectral changes were observed. In the case of all examined systems, the PSS was reached after the first irradiation. This procedure was then repeated for the remaining wavelengths (λ = 405, 530 nm).

**Figure 5:**
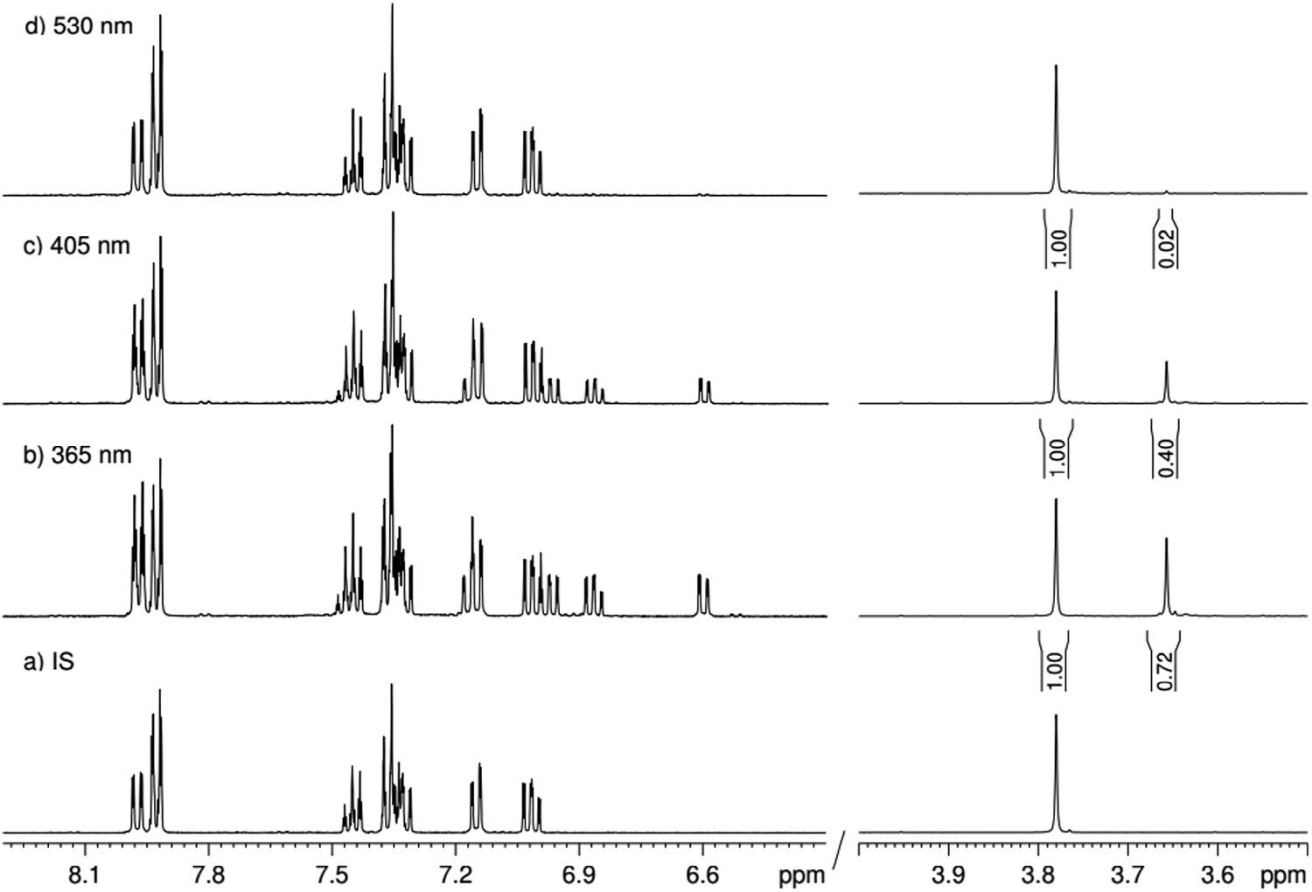
Sections from the ^1^H NMR spectra of the azoarene **1d**: a) after synthesis (initial state, *trans*/*cis*: 100/0), b) after UV irradiation with λ = 365 nm (*trans*/*cis*: 58/42), c) after irradiation with λ = 405 nm (*trans*/*cis*: 72/28) d) and after irradiation with λ = 530 nm (*trans*/*cis*: 98/2) (CD_2_Cl_2_, 400 MHz, c = 5 mm).

As an example, Figure 5 shows the ^1^H NMR spectra of the photo stationary states of **1d** after irradiation with different wavelengths. In its initial state, **1d** exists exclusively as the *trans* isomer (Figure 5a). Upon irradiation with λ = 365 nm, a *cis* ratio of 42% is observed (Figure 5b). Subsequent irradiation with λ = 405 nm and λ = 530 nm results in a gradual reduction of the *cis* ratio to 28% and eventually to 2%. This process is evidenced by the decreasing intensity of the signal at 3.65 ppm (*cis* isomer) relative to the singlet at 3.75 ppm (*trans* isomer). This is the first example of an azoarene with a divalent tellurium center in *ortho* position that shows significant *cis*/*trans* isomerization upon irradiation. We attribute this finding to the increase of the π* orbital energy by the electron‐donating pyrazole unit. This induces destabilization of the π* orbital and thus enables rotation around the N─N bond in the excited state (Figure 1b).

**Figure 6:**
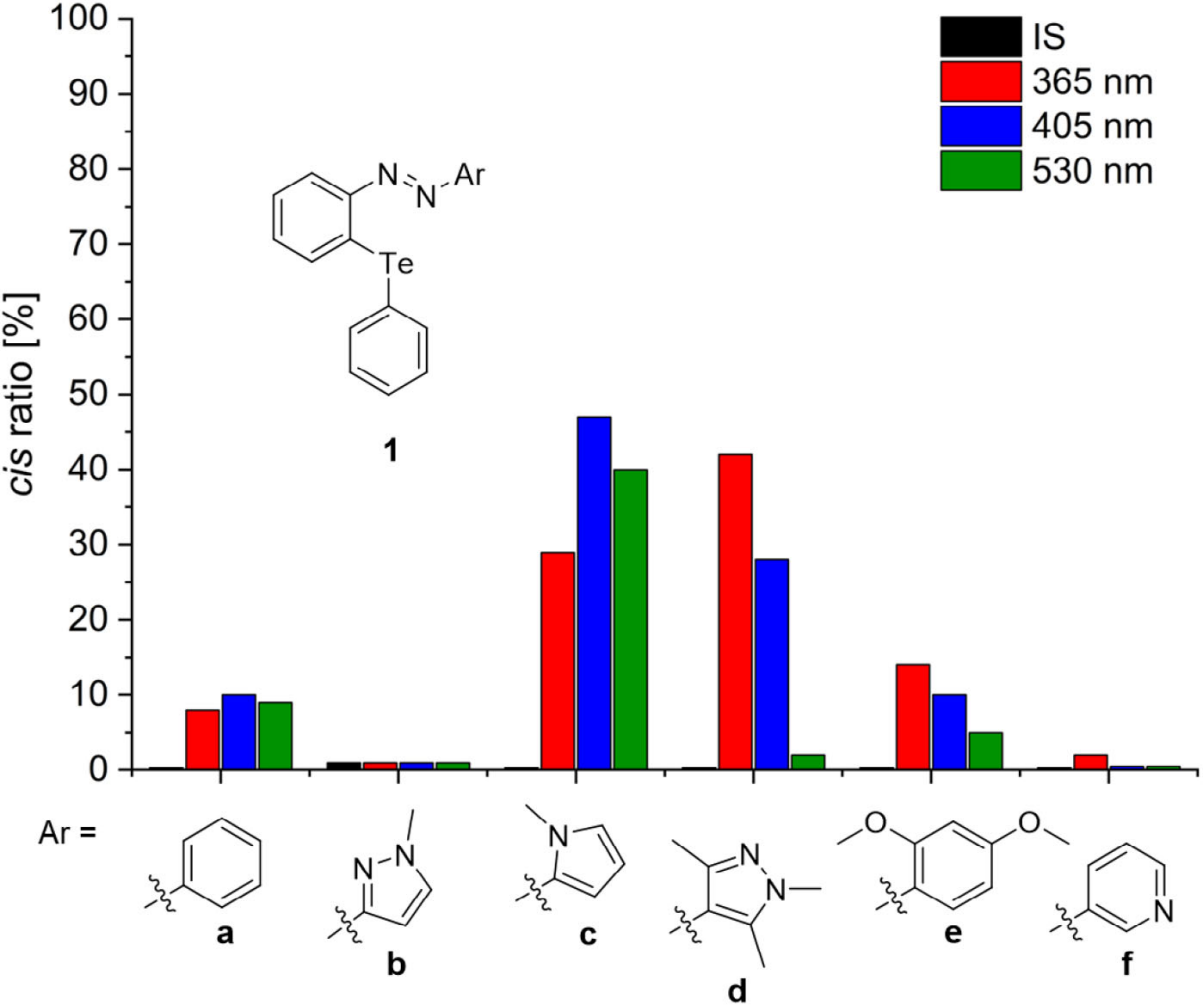
Overview of the *cis* ratio [%] for the investigated azoarenes **1** after different photochemical treatments. After synthesis (IS, black), after irradiation with λ = 365 nm (red), after irradiation with λ = 405 nm (blue) and after irradiation with λ = 530 nm (green).

The electron‐rich pyrazole derivative **1d** and the pyrrole‐containing azoarene **1c** exhibit significantly improved photoswitching behavior compared to the reference compound **1a** (Figure 6). In contrast, compounds **1b** and **1f** show no observable isomerization upon irradiation, while **1e** reaches a *cis* ratio of 14% under λ = 365 nm irradiation, displaying similar switching properties as **1a**. These findings are in good agreement with the Hammett parameters of the parent (hetero)aryl moieties, which are greater than zero (electron‐withdrawing) for the pyrazole **1b** and pyridine **1f** and less than zero (electron‐donating) for pyrrole **1c**, pyrazole **1d** and dimethoxy derivative **1e** (Figure S65).^[^ ^83^ ^]^ Given the electron‐donating nature of the aryl unit in **1e**, a higher *cis* ratio in the photo stationary state would be expected. The limited photoisomerizability of **1e** can likely be attributed to the additional intramolecular stabilization of *trans*‐**1e** via a Te···O interaction (Figure 4).

Among all the compounds investigated, **1c** displays the highest absolute *cis* ratio (47%) upon irradiation at λ = 405 nm. In comparison, **1d** exhibits the highest *cis* ratio at λ = 365 nm, reaching 42% *cis* isomer (Figure 6). Although **1d** does not attain the highest overall *cis* ratio, it emerges as the most promising photoswitch due to several reasons. Figure 6 provides an overview of the *cis* ratios observed in the PSS for all systems using different irradiation wavelengths. A glance at this summary reveals a marked difference in the responsivity of **1c** and **1d** to irradiation at λ = 530 nm. Compound **1d** undergoes complete back‐isomerization, a characteristic feature of azopyrazoles^[^ ^75^, ^84^ ^]^ enabled by a bathochromic shift of the *n→*π* band in the *cis* isomer. This shift permits selective excitation of *cis*‐**1d** at λ = 530 nm (Figure S4). In contrast, the *cis* ratio of **1c** is only marginally reduced under the same conditions (Figure S3), indicating that back‐isomerization for **1c** is primarily thermal. Furthermore, the pyrazole system **1d** displays a significantly longer thermal half‐life of approximately two days, which is two orders of magnitude greater than that of the pyrrole **1c** (τ_1/2_ = 0.3 h, Figures S45 and S46).

**Figure 7:**
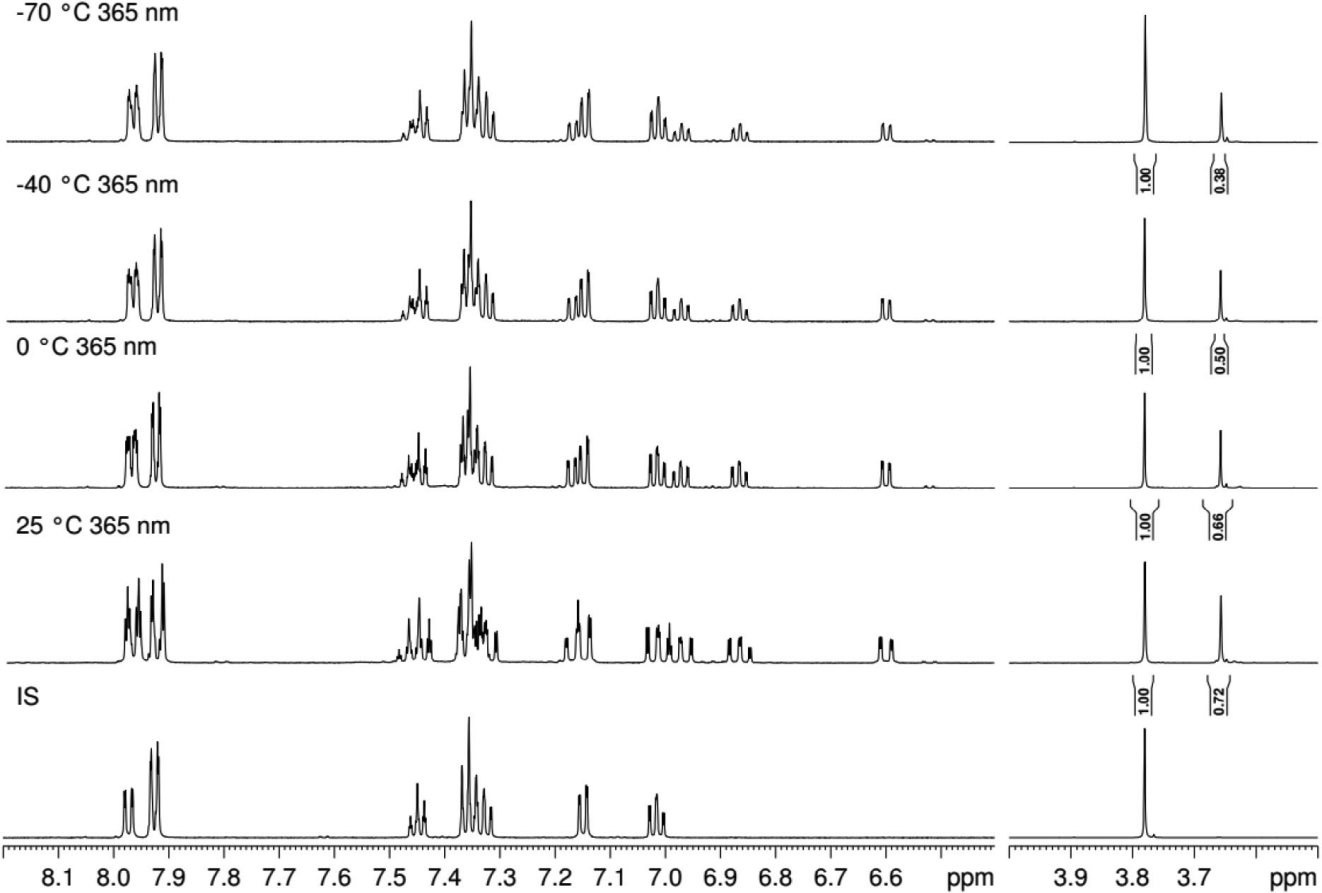
Section from the ^1^H NMR spectra of the azoarene **1d**: after synthesis (*trans*/*cis*: 99/1), after UV irradiation with λ = 365 nm at room temperature (*trans*/*cis*: 58/42), 0 °C (*trans*/*cis*: 60/40), −40 °C (*trans*/*cis*: 67/33), −70 °C (*trans*/*cis*: 72/28) (CD_2_Cl_2_, 600 MHz, c = 5 mm).

Following the determination of the photophysical properties of the azoarenes **1** at room temperature, temperature‐dependent switching experiments were carried out for the switchable tellurium‐substituted compound **1d** as well as the reference compounds **4** and **5**. These experiments were performed in dichloromethane at room temperature (25 °C), 0, −40, and −70 °C (Figures S41–S43). The NMR samples were tempered to the corresponding temperature and irradiated with light of wavelength λ = 365 nm for 60 s. The NMR spectra were then recorded at room temperature (25 °C). To ensure that the photo stationary state (PSS) was reached, this process (irradiation and measurement) was repeated until no further change in the spectrum could be observed. Figure 7 shows the ^1^H NMR spectra of the PSS obtained after irradiation at different temperatures for tellurium‐substituted azoarene **1d**. As described above, before irradiation, **1d** exists almost exclusively as the *trans* isomer (Figure 7). Upon irradiation at 25 °C, a *cis* fraction of up to 42% is observed. The reduction of the irradiation temperature is accompanied by a gradual decrease in the *cis* ratio in the PSS. The integral of the signal at 3.65 ppm (*cis*‐**1d**) relative to the signal at 3.75 ppm (*trans*‐**1d**) decreases from a ratio of 0.72 (25 °C) to 0.38 (−70 °C), corresponding to a reduction in the *cis* ratio from 42% to 28%.

**Figure 8:**
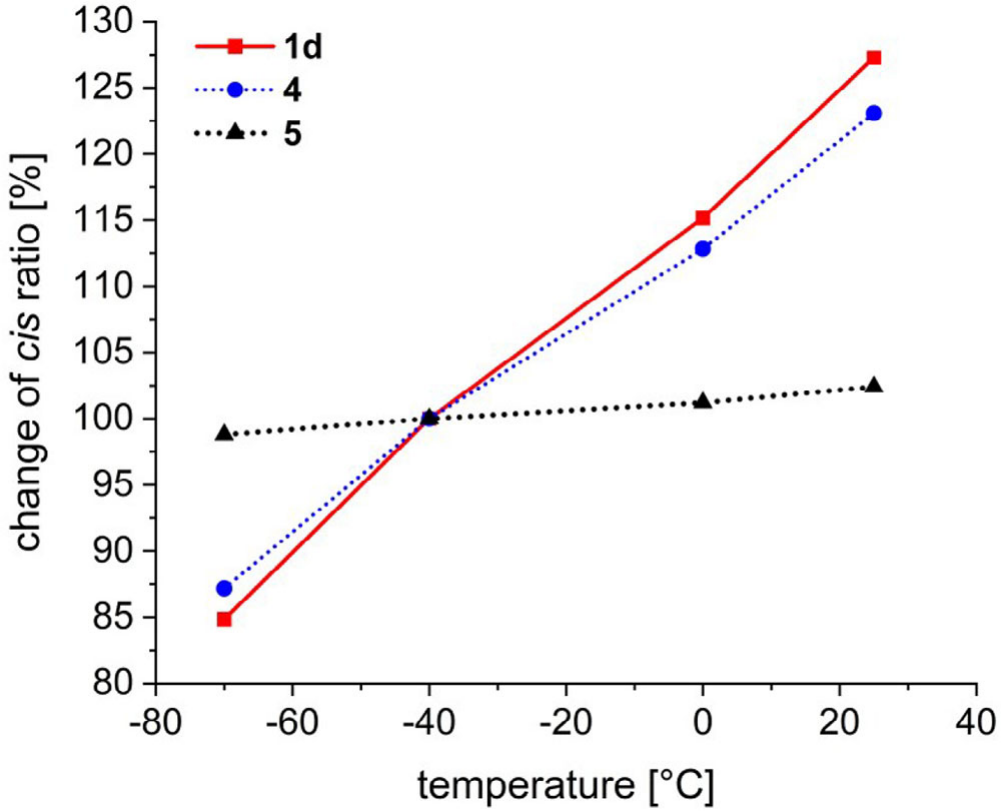
Change of the *cis* ratio of the investigated azoarenes **1d**, **4,** and **5** at different temperatures (CD_2_Cl_2_, *c* = 5 mm). The *cis* ratio at −40 °C is normalized to 100%.

A similar dependence of the *cis* ratio in the PSS is observed for the selenium‐containing compound **4** (Figure S42).^[^ ^66^ ^]^ In comparison, no pronounced temperature dependence is seen for the *para*‐selenium‐substituted compound **5** (Figure S43). For the purpose of improved discussion, the changes in the *cis* ratios as a function of temperature are presented graphically in Figure 8. To ensure comparability of the results with earlier studies,^[^ ^66^ ^]^ the *cis* ratio at −40 °C was normalized to 100%. In the case of the previously discussed selenium‐substituted azoarene **4**, the temperature dependence of the photoswitching behavior can be attributed to the equilibrium between the two minima on the potential energy surface of the excited S_1_ state (Figure 1a, ***B***). As a reminder: n→π* excitation by light can induce a change in the bond type between a divalent chalcogen atom and a Lewis base center. This interaction accounts for the unusual minimum on the S_1_ potential energy surface at a dihedral angle of θ = 180°. At higher irradiation temperatures, a higher *cis* ratio is observed, even though the *cis* isomer is thermodynamically less stable than the *trans* form. The absence of temperature‐dependence in the *para*‐substituted compound **5** further highlights that this effect arises specifically from an interaction between the chalcogen and the azo bridge, rather than from the mere presence of the chalcogen atom itself. The temperature dependence of the isomeric ratio in the tellurium‐containing system **1d** provides experimental evidence for the destabilization of the excited state geometry with a dihedral angle of θ = 180°, which corresponds to the formation of a three‐electron σ bond. The introduction of an electron‐donating substituent was found to reduce the energy difference between the two minima on the potential energy surface, thereby restoring photoisomerizability. This behavior has previously been observed in selenium‐substituted compounds (see Figure 1a, ***B***), where the stabilizing contribution of the three‐electron σ bond interaction is less pronounced compared to their tellurium analog.^[^ ^68^ ^]^

In addition, the impact of oxidation of the tellurium center on the switching behavior was investigated. For this purpose, the corresponding dichlorides **2** and dibromides **3** of the parent systems **1** were synthesized and characterized with respect to their photophysical properties. Switching experiments were carried out using UV/Vis spectroscopy (CH_2_Cl_2_, *c* = 1 mm, Figures S9–S20) and NMR spectroscopy (CD_2_Cl_2_, *c* = 5 mm, Figures S27–S38), and the thermal *cis* half‐lives at room temperature were determined (Figures S47–S56). A summary of the properties of all compounds is provided in Table 1. Except for compounds **2c** and **3c**, a significant improvement in photoswitching performance was observed for the oxidized derivatives. This enhancement can be attributed to the absence of a lone pair at the tellurium(IV) center, which prevents the formation of a three‐electron σ bond in the excited state (see also Figures S68 and S69). Figure 9 provides an overview of the *cis* ratios observed in the photostationary state for **2** and **3** under irradiation with different wavelengths.

**Table 1:** Photophysical properties of the investigated azoarenes **1**–**5** at room temperature (CD_2_Cl_2_, c = 5 mm).

			PSS [*cis* %]^[^ ^d^ ^]^		
Compound^[^ ^a^ ^]^	τ_1/2_ 25 °C [h]^[^ ^b^ ^]^	IS [*cis* %]^[^ ^c^ ^]^	365 nm	405 nm	530 nm
**1a**	‐	0	8	10	9
**2a**	18.75 ± 0.03	0	96	41	14
**3a**	18.50 ± 0.03	14	63	37	17
**1b**	‐	0	<1	<1	<1
**2b**	78.5 ± 0.13	10	93	47	7
**3b**	62.7 ± 0.11	0	80	44	5
**1c**	0.302 ± 0.001	0	29	47	40
**2c**	‐	0	0	0	0
**3c**	‐	0	decomposition		
**1d**	54.19 ± 0.09	0	42	29	2
**2d**	14.20 ± 0.02	0	90	84	3
**3d**	13.19 ± 0.02	0	53	78	4
**1e**	‐	0	14	10	5
**2e**	1.569 ± 0.003	0	54	86	15
**3e**	0.615 ± 0.001	0	38	80	13
**1f**	‐	0	<1	<1	<1
**2f**	23.52 ± 0.04	1	89	30	13
**3f**	10.09 ± 0.02	0	41	24	11
**4**	90.7 ± 0.15	1	47	59	34
**5**	63.5 ± 0.11	1	85	59	24

^[a]^ See Figure 2.

^[b]^ Thermal *cis* half‐lives [h] at room temperature.

^[c]^ *cis* ratio at the initial state determined via ^1^H NMR spectroscopy.

^[d]^ *cis* ratio of the photo stationary state at different wavelengths determined via ^1^H NMR spectroscopy.

**Figure 9:**
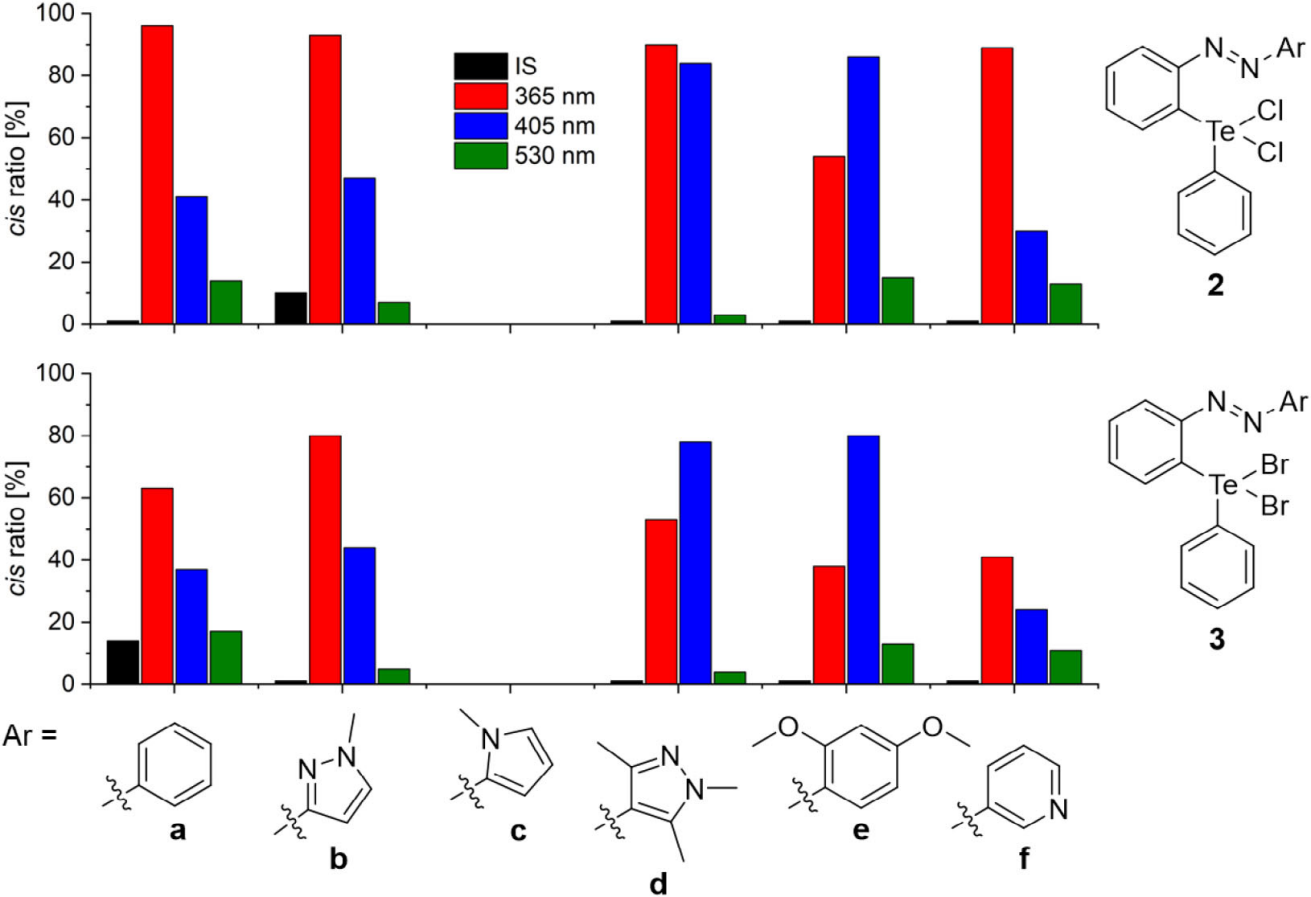
Overview of the *cis* ratios [%] for the investigated azoarenes **2** and **3** after different photochemical treatments. After synthesis (IS, black), after irradiation with λ = 365 nm (red), after irradiation with λ = 405 nm (blue) and after irradiation with λ = 530 nm (green).

For the dichlorides **2**, the maximum percentage of *cis* isomer is independent of the electronic nature of the aryl substituent and ranges between 86% and 96%. Notably, in the case of the electron‐donating compounds **2d** and **2e**, high *cis* ratios are still achieved when irradiated with 405 nm light. For **2e**, the *cis* ratio is significantly higher than under 365 nm irradiation, which can be attributed to the absorption maximum of the π→π* transition being centered at 420 nm (Figure S13). Quantitative back‐isomerization is observed only for the pyrazole derivative **2d**. Despite these differences, all compounds (except **2c**) demonstrate strong photoresponsivity, with thermal *cis* half‐lives ranging from 14 h (**2d**) to 79 h (**2b**). Thus, oxidation from **1d** to the dichloride **2d** reduces the half‐life from 54 to 14 h. If this trend is extrapolated to compound **1c** (τ_1/2_ = 20 min), it can be assumed that the thermal half‐life of dichloride **2c** is too short to allow observation of the *cis* isomer within the UV/Vis timescale. This assumption was supported by a UV/Vis switching experiment of compound **2c** at −10 °C, in which photoisomerization was observed (Figure S44). Like the reference compound **2a**, systems **2b**, **2e**, and **2f** can thus be classified as multiresponsive switches, where both the irradiation wavelength and the oxidation state of the tellurium center play a decisive role in determining the switching behavior.

The switching properties of dibromides **3** are qualitatively like those of dichlorides **2** (Figure 9). However, the maximum achievable *cis* ratio is generally lower (e.g., **3f**: 41%; **3b**, **3e**: 80%). This behavior can be attributed to differences observed in the UV/Vis spectra (Figures S9–S20): while the wavelength of the π→π* transition for the *trans* isomer remains unaffected by the halogen, the *cis* isomers of the dichlorides **2** exhibit a more pronounced hypsochromic shift. This shift results in reduced spectral overlap between the π→π* transition of *cis* and *trans* isomers, thereby enabling more efficient photoisomerization and ultimately higher *cis* ratios. In addition, a shorter thermal half‐life is observed for the dibromides **3** (Table 1).

The pyrrole derivatives **2c** and **3c** represent exceptions to the general trends described above. Oxidation to the dichloride or dibromide results in either an undetectable *cis* ratio in the PSS (**2c**) or decomposition (**3c**). The short *cis* half‐life of **2c**, which results in no observable *cis* isomer at room temperature, provides another system in which the photophysical properties are strongly dependent on the oxidation state of the tellurium center.

Diaryl chalcogens are known for degradation under UV light,^[^ ^85^, ^86^ ^]^ and two irradiation cycles with light of wavelength 365 nm of diphenyl telluride led to complete degradation to benzene as main product (Figure S64). Whereas only minor photodegradation could be observed for the divalent compound **1d**. Nevertheless, we are aware that the toxicity and environmental effects, especially of organotellurium species, make these photoswitches unsuitable for biomedical applications. UV/Vis investigations of the photofatigue resistance of the introduced compounds showed excellent photostability (50 cycles) for the tetravalent compound **2d** with no significant influence of oxygen exclusion (see Figures S61 and S62). Even the divalent trimethyl pyrazole derivative **1d** showed sufficient stability over twenty switching cycles, where again oxygen exclusion showed no pronounced difference (see Figures S59 and S60).

## 3 Conclusion

This study demonstrates that the photoswitching behavior of *ortho*‐tellurium‐substituted azoarenes can be effectively modulated by both the electronic properties of the aryl substituent and the oxidation state of the tellurium center. Electron‐donating groups were shown to destabilize the excited‐state geometry associated with the formation of a three‐electron σ bond, thereby restoring photoisomerizability. This hypothesis was supported by temperature‐dependent switching experiments, which revealed a clear correlation between increased temperature and enhanced isomerization. Moreover, the absence of temperature‐dependent switching in the *para*‐substituted reference compound confirms that the observed behavior originates from a specific geometric interaction between the chalcogen and the azo bridge, rather than from the presence of the chalcogen atom alone.

After oxidation to tetravalent Te(IV) species, the tellurium atom no longer possesses a lone pair capable of forming a stabilizing three‐electron σ bond, leading to significantly enhanced photoswitching properties in most cases. By leveraging substituent electronic properties and oxidation state as orthogonal control elements, it is possible to tune the switching properties of azoarenes. This opens the door to future applications in molecular machines, stimuli‐responsive materials, and redox‐controlled supramolecular systems.

## Supporting Information

Figures and tables, NMR and UV spectra, synthesis of new compounds, computational details, cartesian coordinates and absolute energies for all calculated compounds, crystal structure data, and ^1^H NMR, ^13^C NMR, ^77^Se NMR, and ^125^Te NMR spectra of the new compounds. Deposition Numbers 2444456 (for **1c**), 2444457 (for **1d**), 2444458 (for **1e**), 2444459 (for **2b**), 2444460 (for **2c**), 2444461 (for **2d**), 2444462 (for **2e**), 2444463 (for **2f**), 2444464 (for **3a**), 2444465 (for **3c**), 2444466 (for **3d**), 2444467 (for **3e**), 2444468 (for **3f**), 2444469 (for **6e**), 2444470 (for **6f**) contain the supplementary crystallographic data for this paper. These data are provided free of charge by the joint Cambridge Crystallographic Data Centre and Fachinformationszentrum Karlsruhe Access Structures service.

## Conflict of Interest

There is no conflict of interest to declare.

## Supporting information



Supporting Information

Supporting Information

Supporting Information

## Data Availability

The data that support the findings of this study are available in the supplementary material of this article.
