# Supplementary material for: Chalcogen‐Guided Control of Azoarene Photoswitching: Tuning Excited‐State Energies Through Electronic Property Modulation
Source: Chemistry. 2025 Jul 22;31(45):e01571. doi: 10.1002/chem.202501571 (PMC12351432; doi:10.1002/chem.202501571)
Supplement: Supplementary file 1 — Supporting Information [file CHEM-31-e01571-s001.pdf]

Supporting Information

Chalcogen-Guided Control of Azoarene Photoswitching: Tuning Excited-State Energies through Electronic Property Modulation

Zoe Nonie Scheller[†], Jan Schulte[†], Christoph Wölper[†] and Gebhard Haberhauer*[†]

[†] Institut für Organische Chemie, Universität Duisburg-Essen, Universitätsstr. 7, D-45117 Essen, Germany

1. **UV and NMR Photoswitching Experiments** **S2**
2. **Determination of *cis* Half-Lives** **S37**
3. **Photofatigue and Photostability** **S51**
4. **Hammett Constants** **S55**
5. **Synthesis of New Compounds** **S56**
5. **Computational Details** **S85**
6. **Cartesian Coordinates and Absolute Energies for All Calculated Compounds** **S90**
7. **Crystal Structure Data** **S141**
8. **^{1}H NMR, ^{13}C NMR and ^{125}Te NMR Spectra of the New Compounds** **S174**
9. **Supporting Information References** **S219**

1. UV and NMR Photoswitching Experiments

General Procedure for the Irradiation of the Samples. As radiation sources LEDs from Sahlmann Photochemical Solutions were used. The exact specifications are as following: λ = 365 nm, LED type: 3× Nichia NC4U133A, total optical power: 3300 mW; λ = 405 nm, LED type: 3× Roithner APG2C1-405, total optical power: 315 mW; λ = 530 nm, LED type: 3× Luxeon LXML-PM01, total optical power: 763 mW. For the investigation of the switching process the sample was placed in a distance of 1 cm to the light source and was irradiated with the corresponding LED for 60 s (NMR) respectively 5 s (UV/Vis). Thereafter, the ratio of the *trans*/*cis* form was determined using NMR spectroscopy. This process was repeated until the PSS was reached. In general, the first irradiation already leads to the PSS. For the temperature-dependent photoswitching studies, the sample was tempered to the corresponding temperature first and then irradiated with λ = 365 nm. The NMR spectra was then recorded at room temperature (25 °C). In this case, too, the first irradiation already led to the photo stationary state.

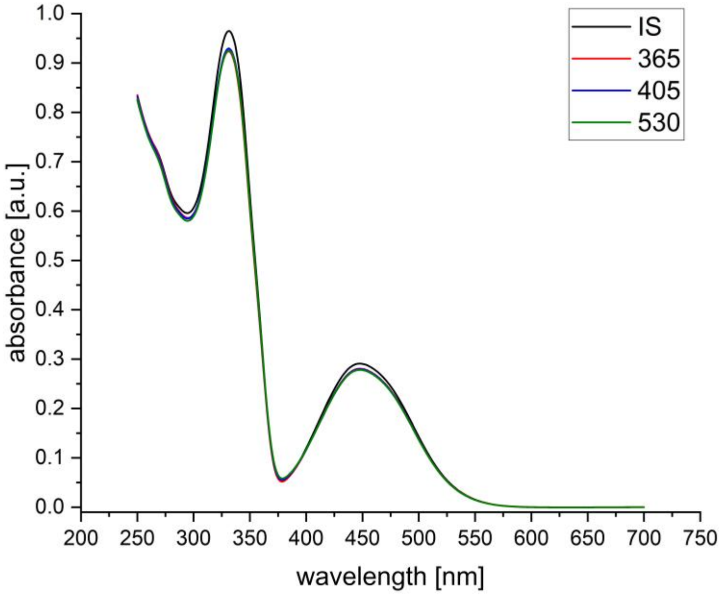

Figure S1. Normalized UV/Vis spectra of the azobenzene **1a**: after synthesis (initial state, black), after UV irradiation with λ = 365 nm (red), after irradiation with λ = 405 nm (blue) and after irradiation with λ = 530 nm (green) (CH_2Cl_2, c = 1.0 mM).

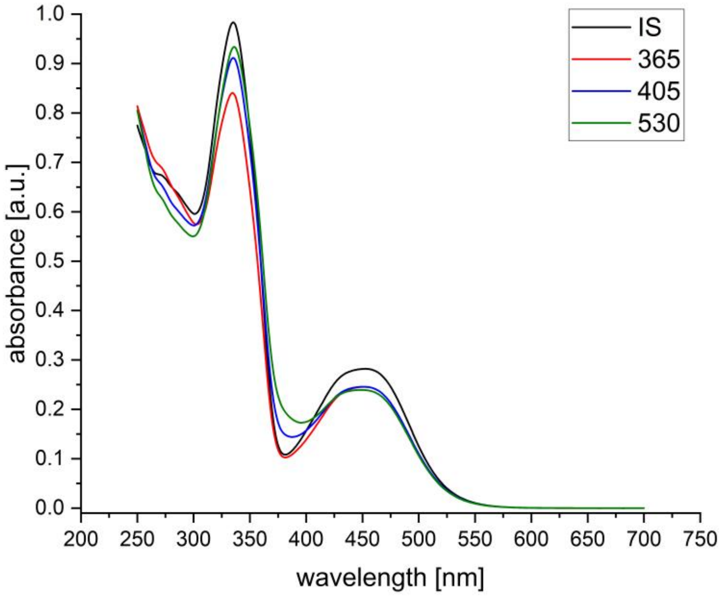

Figure S2. Normalized UV/Vis spectra of the azoarene **1b**: after synthesis (initial state, black), after UV irradiation with λ = 365 nm (red), after irradiation with λ = 405 nm (blue) and after irradiation with λ = 530 nm (green) (CH_2Cl_2, c = 1.0 mM).

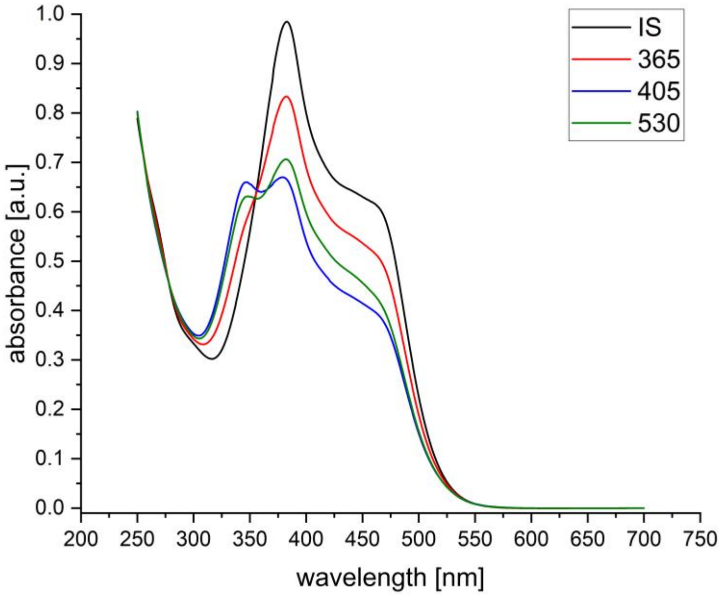

Figure S3. Normalized UV/Vis spectra of the azoarene **1c**: after synthesis (initial state, black), after UV irradiation with λ = 365 nm (red), after irradiation with λ = 405 nm (blue) and after irradiation with λ = 530 nm (green) (CH_2Cl_2, c = 1.0 mM).

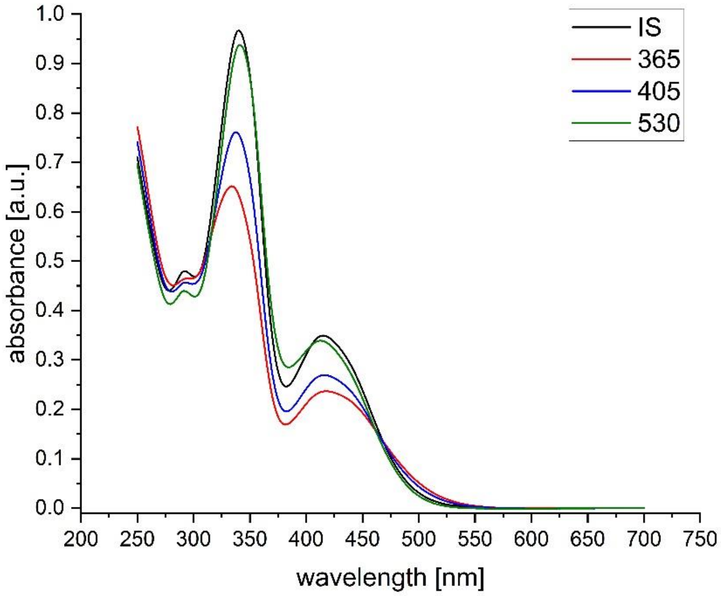

Figure S4. Normalized UV/Vis spectra of the azoarene **1d**: after synthesis (initial state, black), after UV irradiation with λ = 365 nm (red), after irradiation with λ = 405 nm (blue) and after irradiation with λ = 530 nm (green) (CH_2Cl_2, c = 1.0 mM).

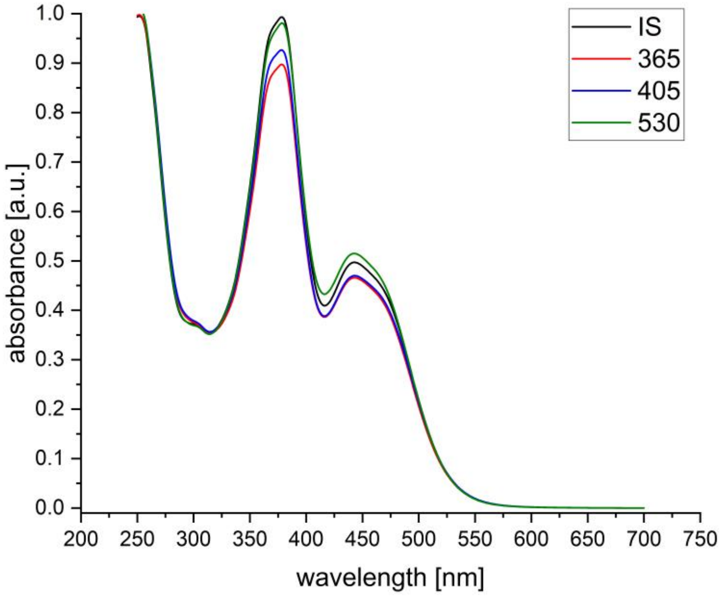

Figure S5. Normalized UV/Vis spectra of the azobenzene **1e**: after synthesis (initial state, black), after UV irradiation with λ = 365 nm (red), after irradiation with λ = 405 nm (blue) and after irradiation with λ = 530 nm (green) (CH_2Cl_2, c = 1.0 mM).

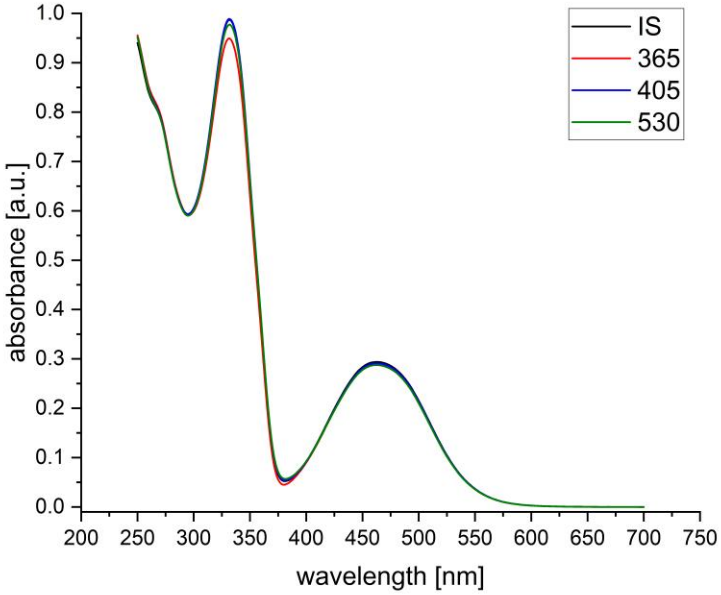

Figure S6. Normalized UV/Vis spectra of the azoarene **1f**: after synthesis (initial state, black), after UV irradiation with λ = 365 nm (red), after irradiation with λ = 405 nm (blue) and after irradiation with λ = 530 nm (green) (CH_2Cl_2, c = 1.0 mM).

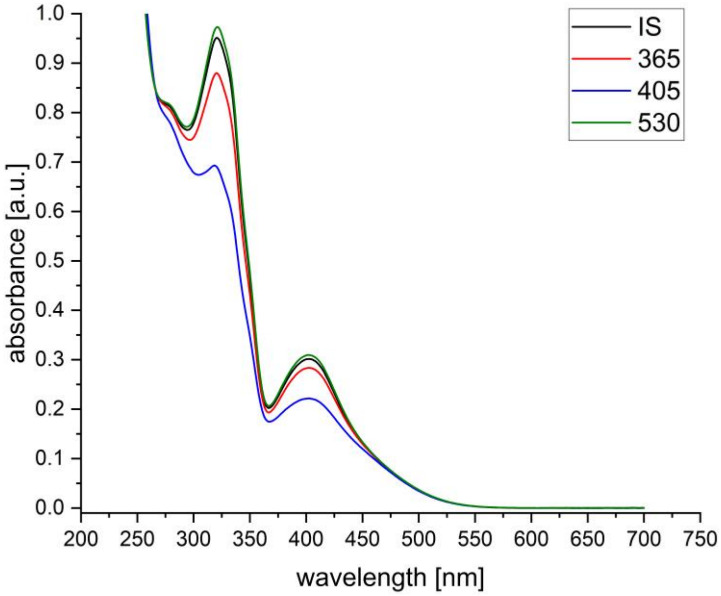

Figure S7. Normalized UV/Vis spectra of the azobenzene **4**: after synthesis (initial state, black), after UV irradiation with λ = 365 nm (red), after irradiation with λ = 405 nm (blue) and after irradiation with λ = 530 nm (green) (CH_2Cl_2, c = 1.0 mM).

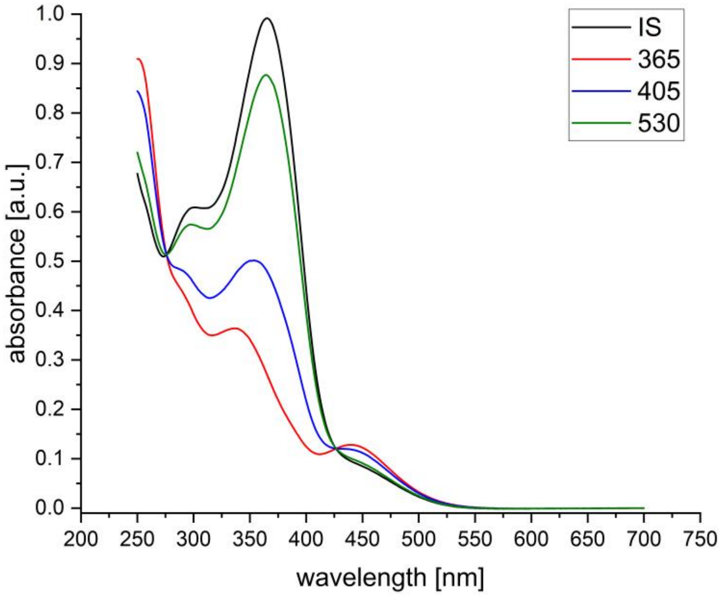

Figure S8. Normalized UV/Vis spectra of the azobenzene **5**: after synthesis (initial state, black), after UV irradiation with λ = 365 nm (red), after irradiation with λ = 405 nm (blue) and after irradiation with λ = 530 nm (green) (CH_2Cl_2, c = 1.0 mM).

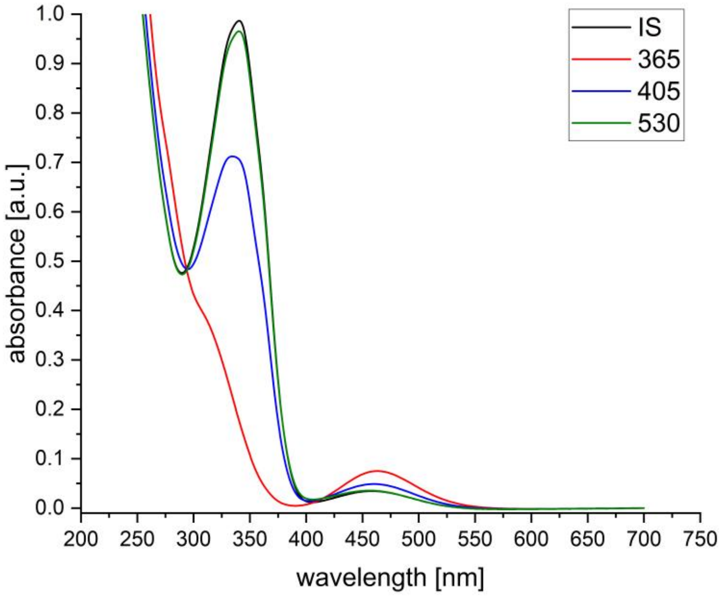

Figure S9. Normalized UV/Vis spectra of the azobenzene **2a**: after synthesis (initial state, black), after UV irradiation with λ = 365 nm (red), after irradiation with λ = 405 nm (blue) and after irradiation with λ = 530 nm (green) (CH_2Cl_2, c = 1.0 mM).

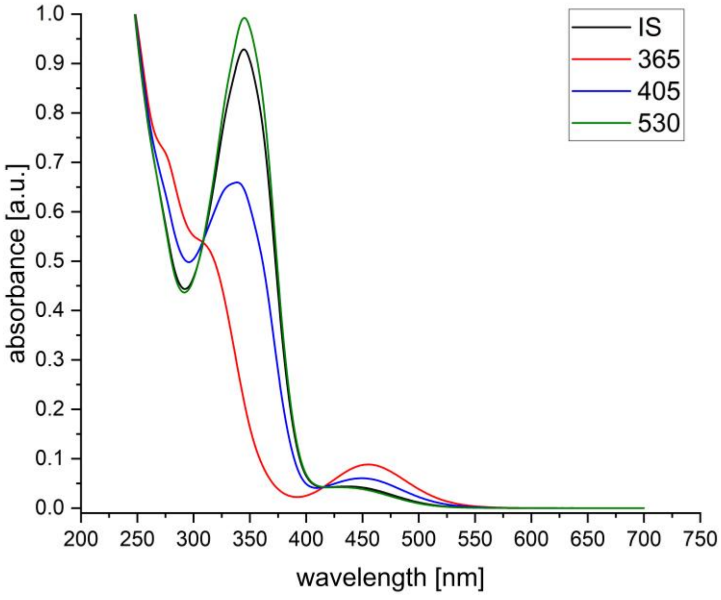

Figure S10. Normalized UV/Vis spectra of the azoarene **2b**: after synthesis (initial state, black), after UV irradiation with λ = 365 nm (red), after irradiation with λ = 405 nm (blue) and after irradiation with λ = 530 nm (green) (CH_2Cl_2, c = 1.0 mM).

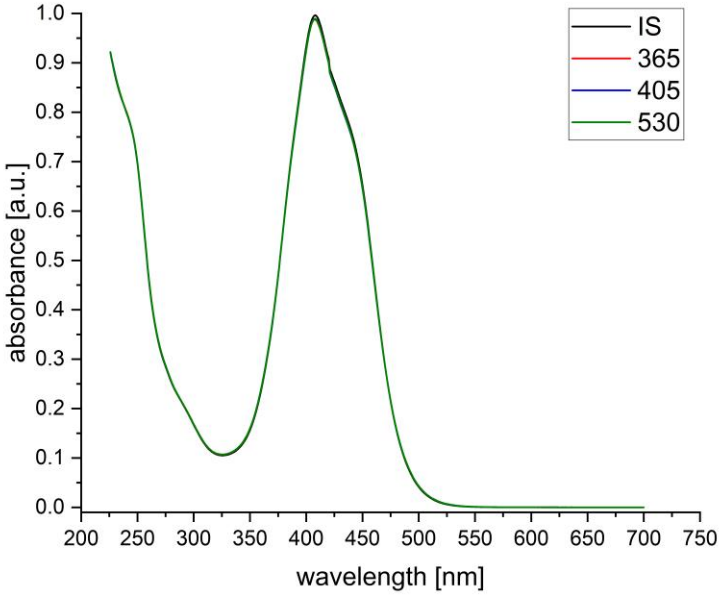

Figure S11. Normalized UV/Vis spectra of the azoarene **2c**: after synthesis (initial state, black), after UV irradiation with λ = 365 nm (red), after irradiation with λ = 405 nm (blue) and after irradiation with λ = 530 nm (green) (CH_2Cl_2, c = 1.0 mM).

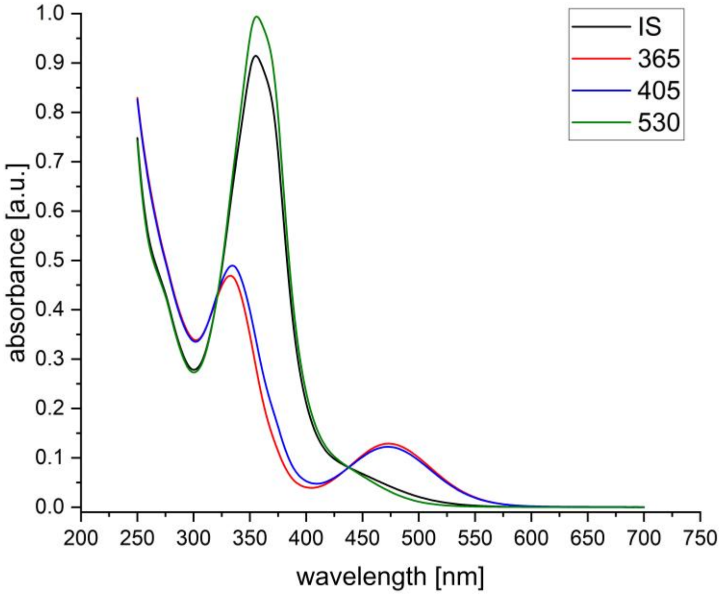

Figure S12. Normalized UV/Vis spectra of the azoarene **2d**: after synthesis (initial state, black), after UV irradiation with λ = 365 nm (red), after irradiation with λ = 405 nm (blue) and after irradiation with λ = 530 nm (green) (CH_2Cl_2, c = 1.0 mM).

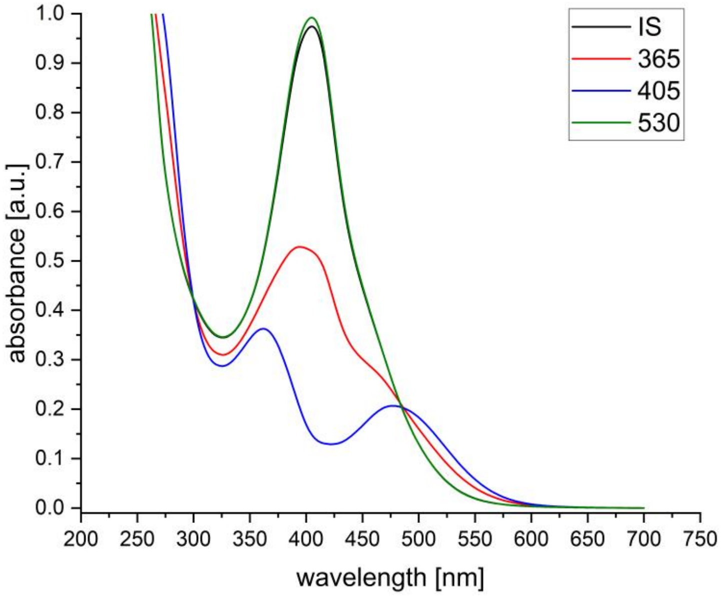

Figure S13. Normalized UV/Vis spectra of the azobenzene **2e**: after synthesis (initial state, black), after UV irradiation with λ = 365 nm (red), after irradiation with λ = 405 nm (blue) and after irradiation with λ = 530 nm (green) (CH_2Cl_2, c = 1.0 mM).

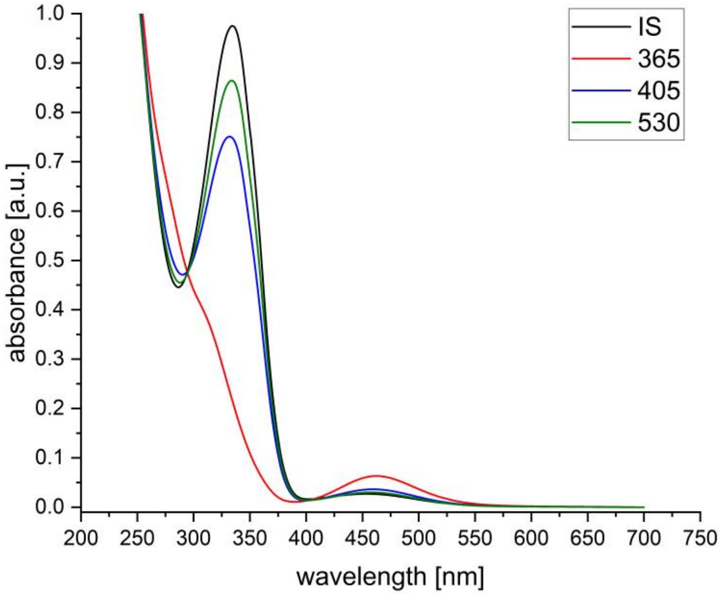

Figure S14. Normalized UV/Vis spectra of the azoarene **2f**: after synthesis (initial state, black), after UV irradiation with λ = 365 nm (red), after irradiation with λ = 405 nm (blue) and after irradiation with λ = 530 nm (green) (CH_2Cl_2, c = 1.0 mM).

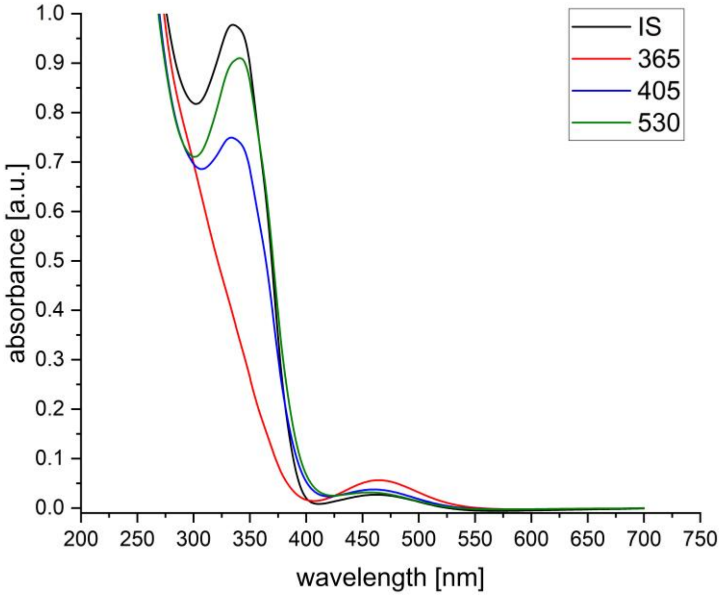

Figure S15. Normalized UV/Vis spectra of the azobenzene **3a**: after synthesis (initial state, black), after UV irradiation with λ = 365 nm (red), after irradiation with λ = 405 nm (blue) and after irradiation with λ = 530 nm (green) (CH_2Cl_2, c = 1.0 mM).

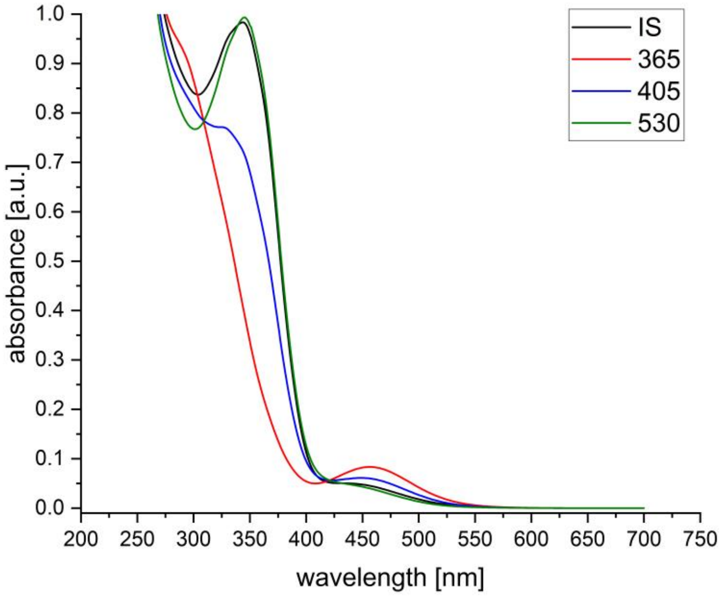

Figure S16. Normalized UV/Vis spectra of the azoarene **3b**: after synthesis (initial state, black), after UV irradiation with λ = 365 nm (red), after irradiation with λ = 405 nm (blue) and after irradiation with λ = 530 nm (green) (CH_2Cl_2, c = 1.0 mM).

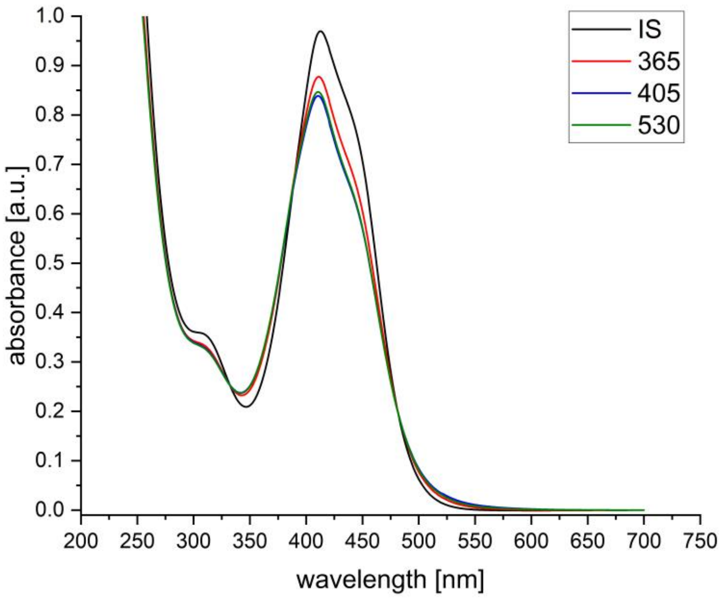

Figure S17. Normalized UV/Vis spectra of the azoarene **3c**: after synthesis (initial state, black), after UV irradiation with λ = 365 nm (red), after irradiation with λ = 405 nm (blue) and after irradiation with λ = 530 nm (green) (CH_2Cl_2, c = 1.0 mM).

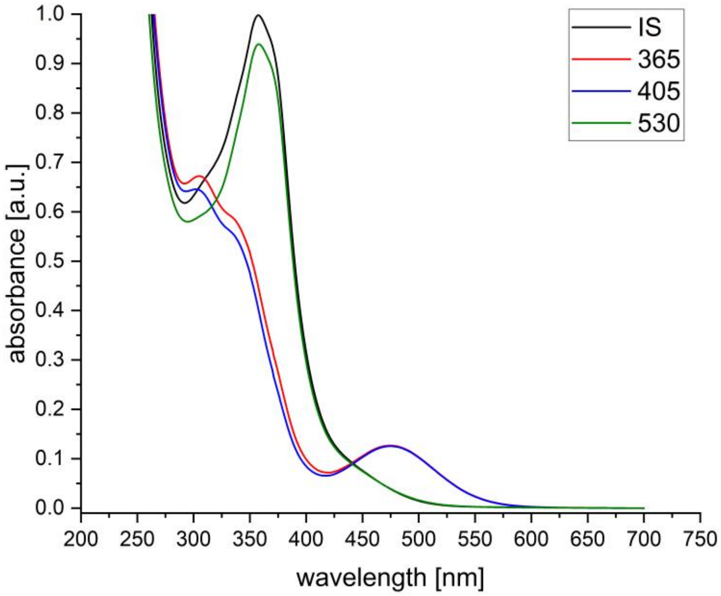

Figure S18. Normalized UV/Vis spectra of the azoarene **3d**: after synthesis (initial state, black), after UV irradiation with λ = 365 nm (red), after irradiation with λ = 405 nm (blue) and after irradiation with λ = 530 nm (green) (CH_2Cl_2, c = 1.0 mM).

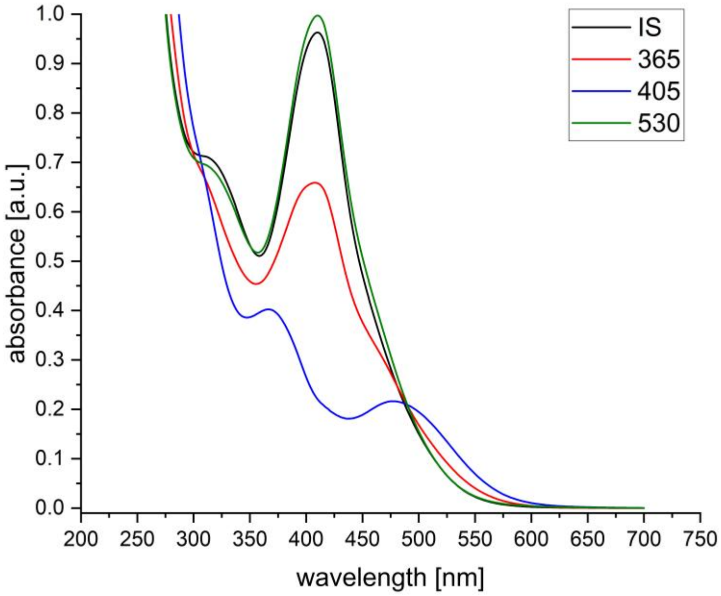

Figure S19. Normalized UV/Vis spectra of the azobenzene **3e**: after synthesis (initial state, black), after UV irradiation with λ = 365 nm (red), after irradiation with λ = 405 nm (blue) and after irradiation with λ = 530 nm (green) (CH_2Cl_2, c = 1.0 mM).

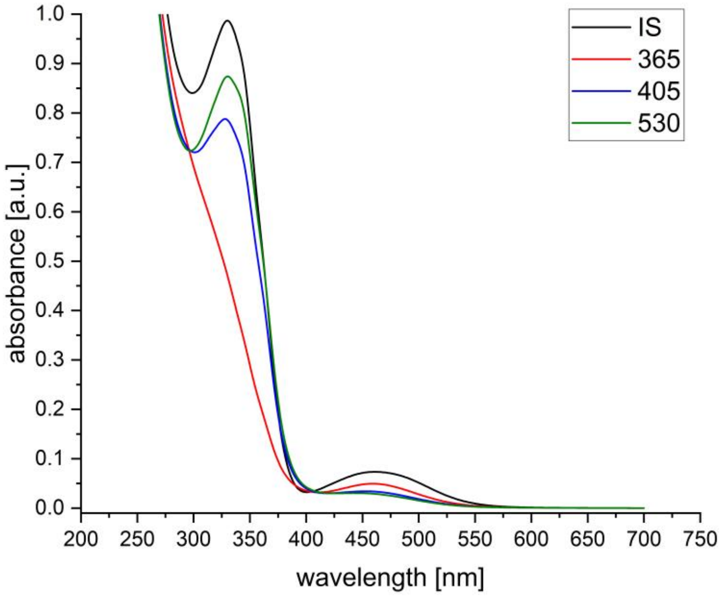

Figure S20. Normalized UV/Vis spectra of the azoarene **3f**: after synthesis (initial state, black), after UV irradiation with λ = 365 nm (red), after irradiation with λ = 405 nm (blue) and after irradiation with λ = 530 nm (green) (CH_2Cl_2, c = 1.0 mM).

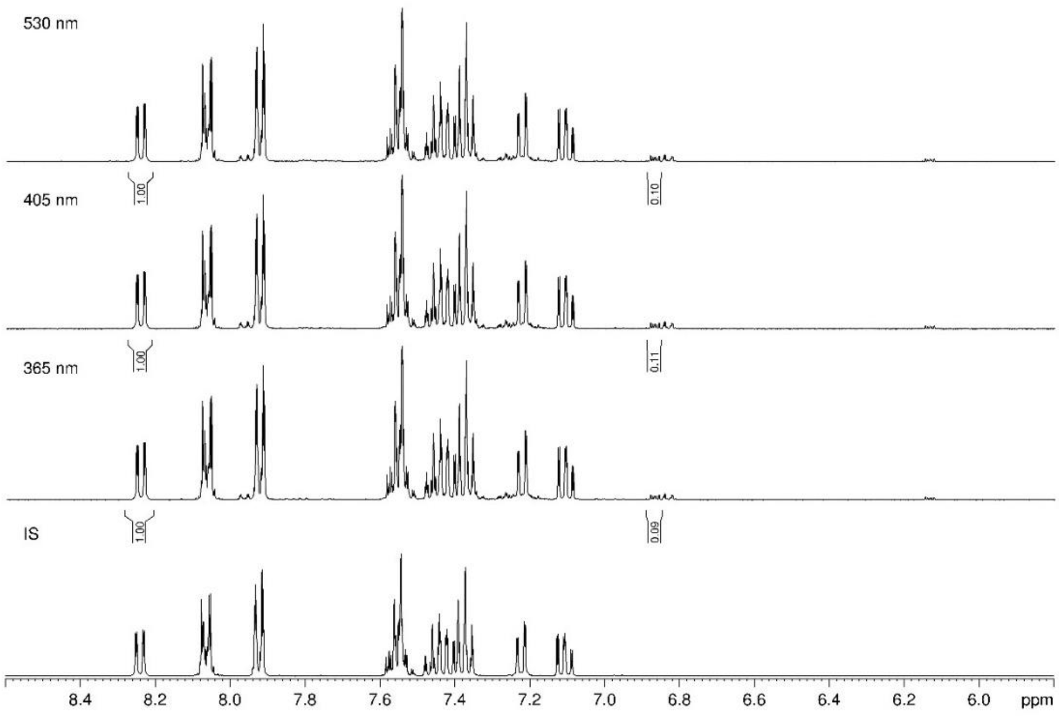

Figure S21. Section from the ^{1}H NMR spectra of the azobenzene **1a**: after synthesis (initial state, *trans*/*cis*: 100/0), after UV irradiation with λ = 365 nm (*trans*/*cis:* 92/8), after irradiation with λ = 405 nm (*trans*/*cis:* 90/10) and after irradiation with λ = 530 nm (*trans*/*cis:* 91/9) (CD_2Cl_2, 400 MHz, c = 5 mM).

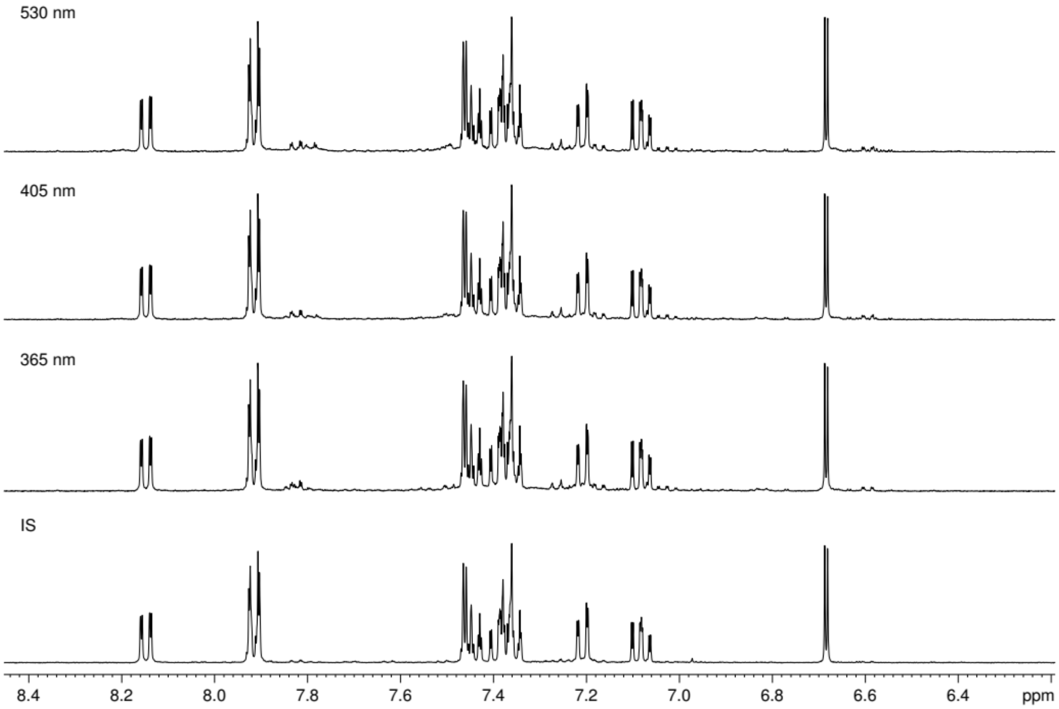

Figure S22. Section from the ^{1}H NMR spectra of the azoarene **1b**: after synthesis (initial state, *trans*/*cis*: 100/0), after UV irradiation with λ = 365 nm (*trans*/*cis*: 100/0), after irradiation with λ = 405 nm (*trans*/*cis*: 100/0) and after irradiation with λ = 530 nm (*trans*/*cis*: 100/0) (CD_2Cl_2, 400 MHz, c = 5 mM).

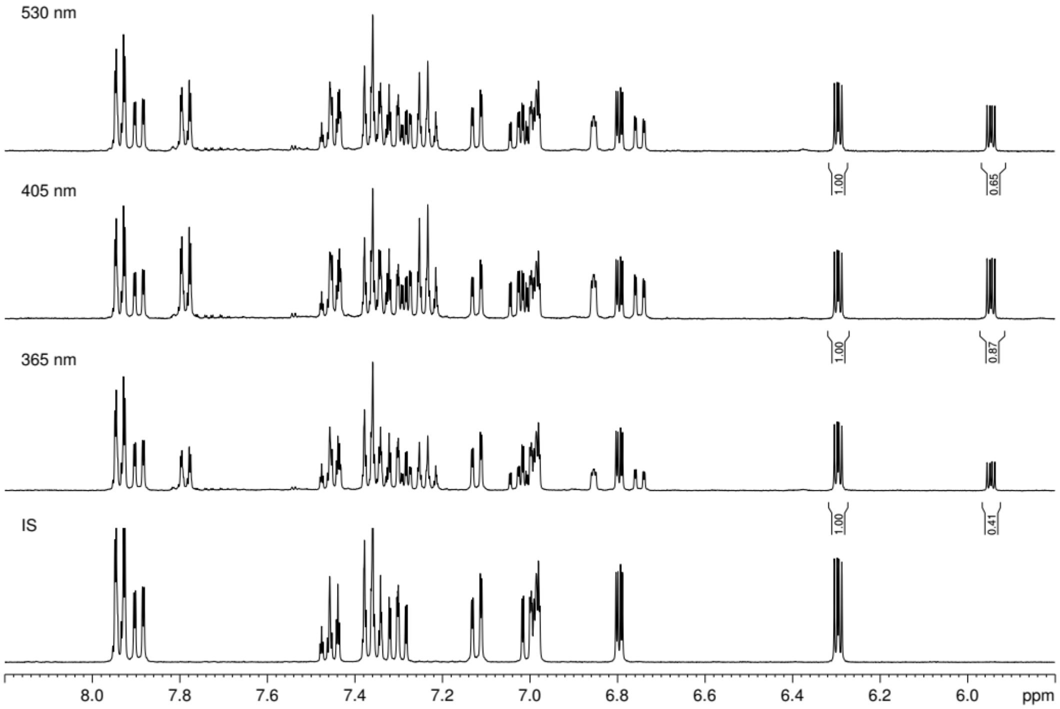

Figure S23. Section from the ^{1}H NMR spectra of the azoarene **1c**: after synthesis (initial state, *trans*/*cis:* 100/0), after UV irradiation with λ = 365 nm (*trans*/*cis:* 71/29), after irradiation with λ = 405 nm (*trans*/*cis:* 53/47) and after irradiation with λ = 530 nm (*trans*/*cis:* 60/40) (CD_2Cl_2, 400 MHz, c = 5 mM).

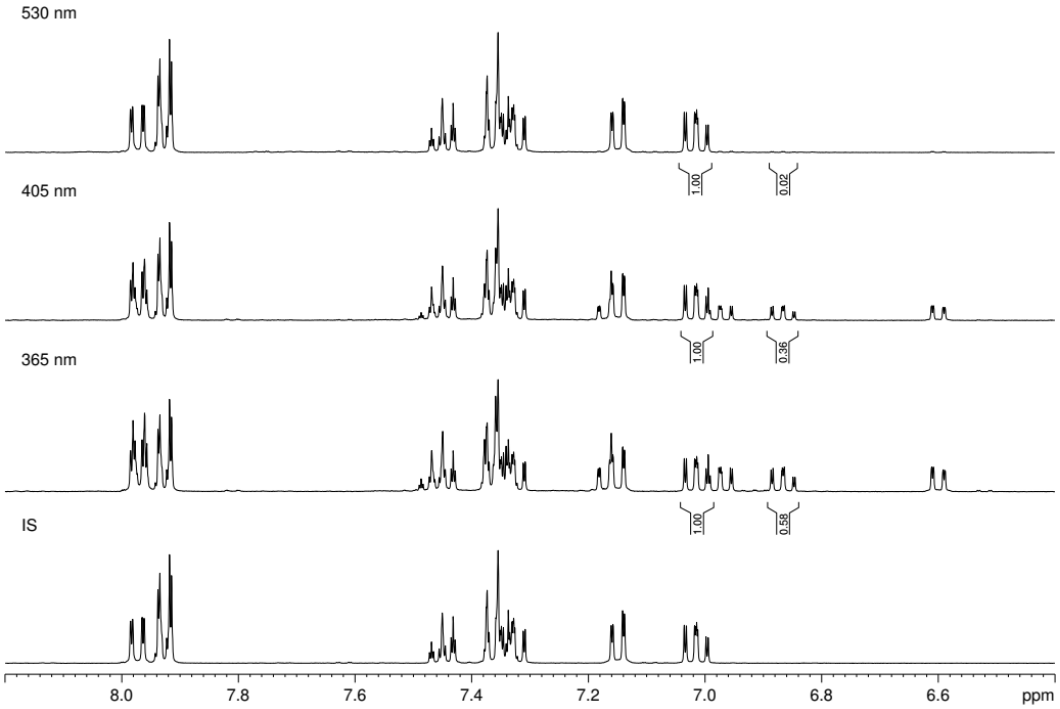

Figure S24. Section from the ^{1}H NMR spectra of the azoarene **1d**: after synthesis (initial state, *trans*/*cis*: 100/0), after UV irradiation with λ = 365 nm (*trans*/*cis:* 63/37), after irradiation with λ = 405 nm (*trans*/*cis:* 74/26) and after irradiation with λ = 530 nm (*trans*/*cis:* 98/2) (CD_2Cl_2, 400 MHz, c = 5 mM).

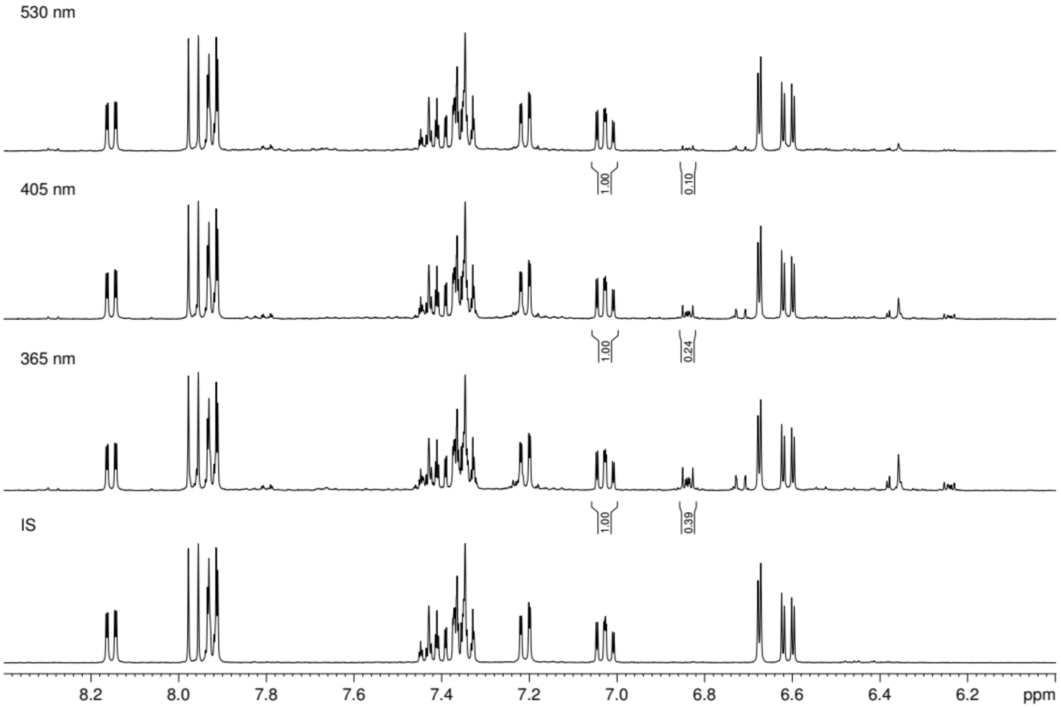

Figure S25. Section from the ^{1}H NMR spectra of the azobenzene **1e**: after synthesis (initial state, *trans*/*cis:* 100/0), after UV irradiation with λ = 365 nm (*trans*/*cis:* 86/14), after irradiation with λ = 405 nm (*trans*/*cis:* 90/10) and after irradiation with λ = 530 nm (*trans*/*cis:* 95/5) (CD_2Cl_2, 400 MHz, c = 5 mM).

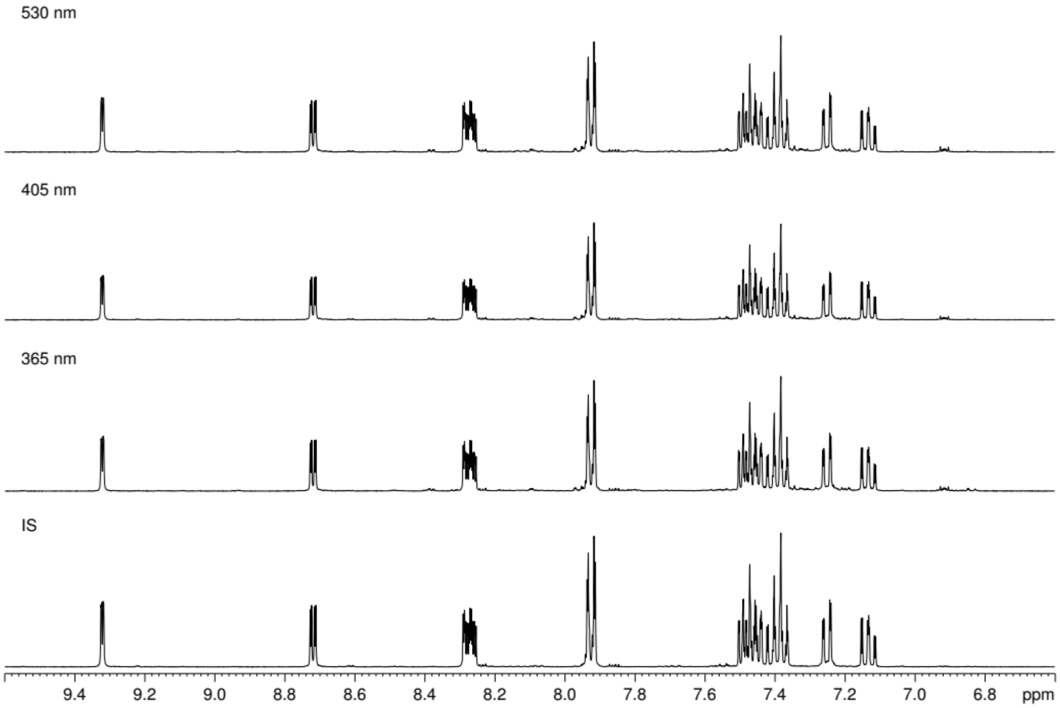

Figure S26. Section from the ^{1}H NMR spectra of the azoarene **1f**: after synthesis (initial state, *trans*/*cis*: 100/0), after UV irradiation with λ = 365 nm (*trans*/*cis*: 99/1), after irradiation with λ = 405 nm (*trans*/*cis*: 99/1) and after irradiation with λ = 530 nm (*trans*/*cis*: 99/1) (CD_2Cl_2, 400 MHz, c = 5 mM).

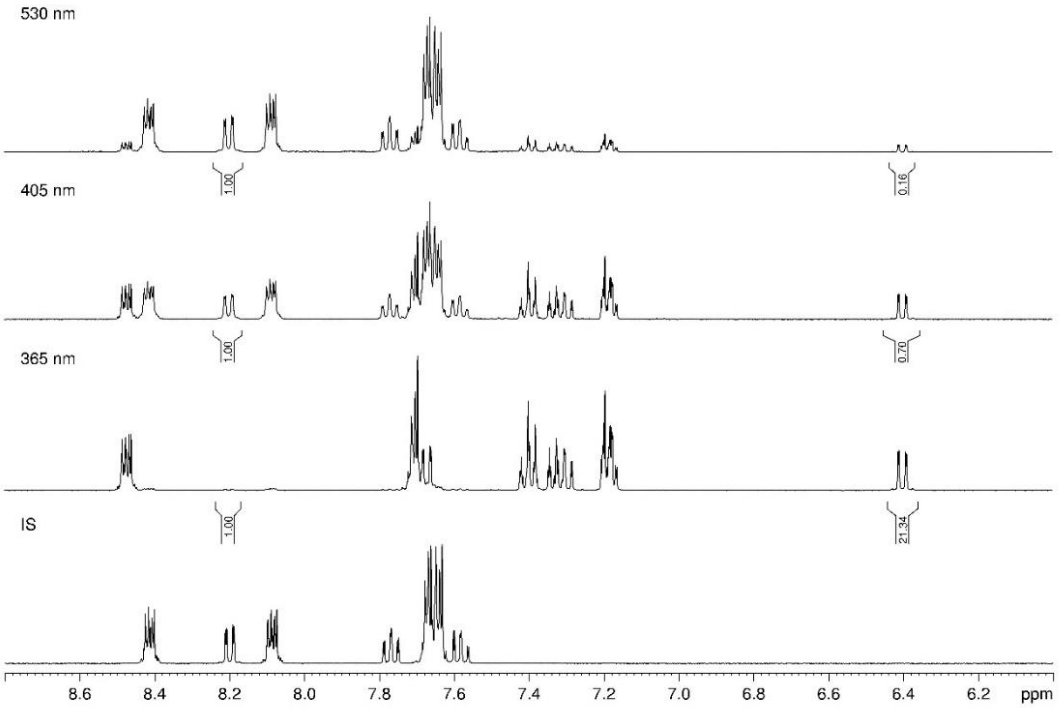

Figure S27. Section from the ^{1}H NMR spectra of the azobenzene **2a**: after synthesis (initial state, *trans*/*cis*: 100/0), after UV irradiation with λ = 365 nm (*trans*/*cis:* 4/96), after irradiation with λ = 405 nm (*trans*/*cis:* 59/41) and after irradiation with λ = 530 nm (*trans*/*cis:* 86/14) (CD_2Cl_2, 400 MHz, c = 5 mM).

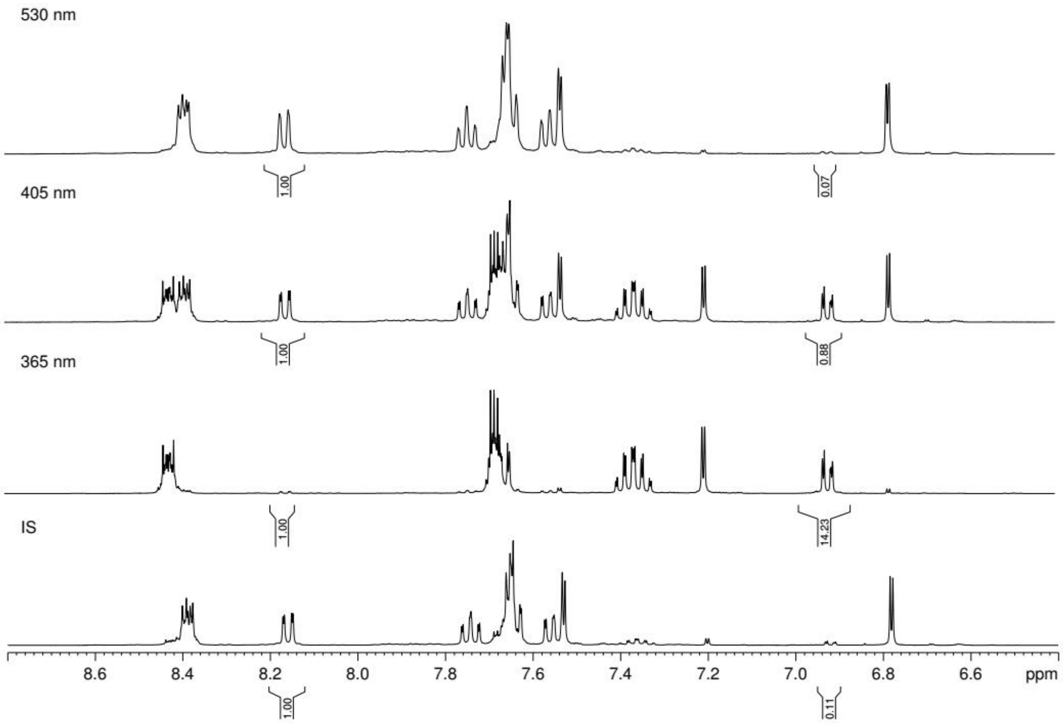

Figure S28. Section from the ^{1}H NMR spectra of the azoarene **2b**: after synthesis (initial state, *trans*/*cis*: 90/10), after UV irradiation with λ = 365 nm (*trans*/*cis:* 7/93), after irradiation with λ = 405 nm (*trans*/*cis:* 53/47) and after irradiation with λ = 530 nm (*trans*/*cis:* 93/7) (CD_2Cl_2, 400 MHz, c = 5 mM).

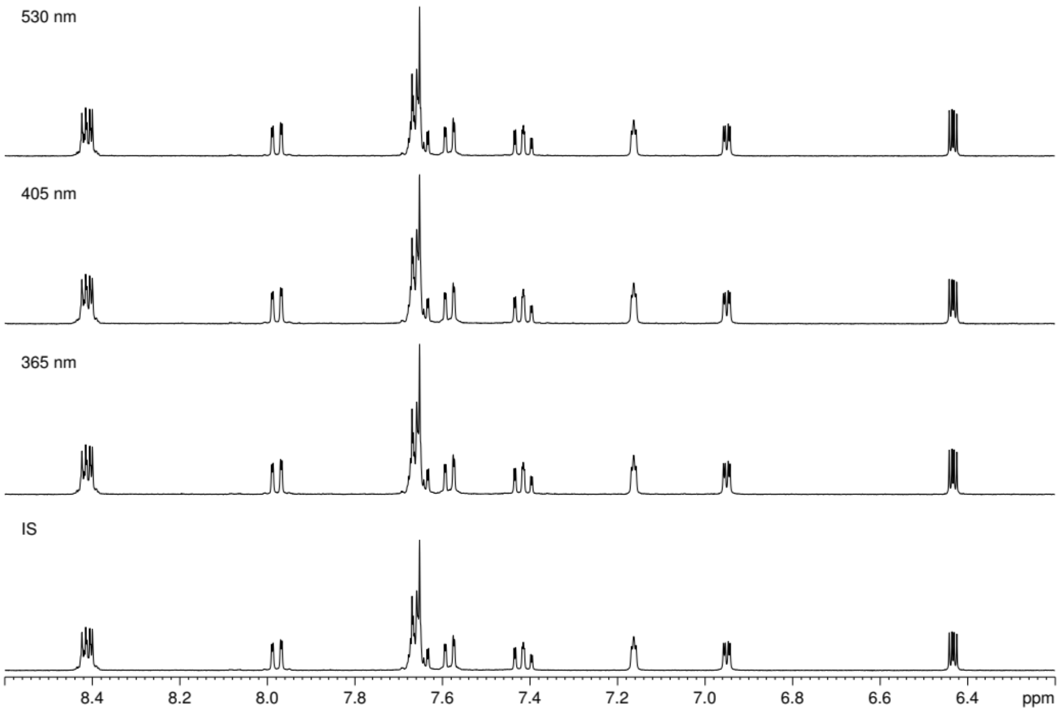

Figure S29. Section from the ^{1}H NMR spectra of the azoarene **2c**: after synthesis (initial state, *trans*/*cis:* 100/0), after UV irradiation with λ = 365 nm (*trans*/*cis:* 100/0), after irradiation with λ = 405 nm (*trans*/*cis:* 100/0) and after irradiation with λ = 530 nm (*trans*/*cis:* 100/0) (CD_2Cl_2, 400 MHz, c = 5 mM).

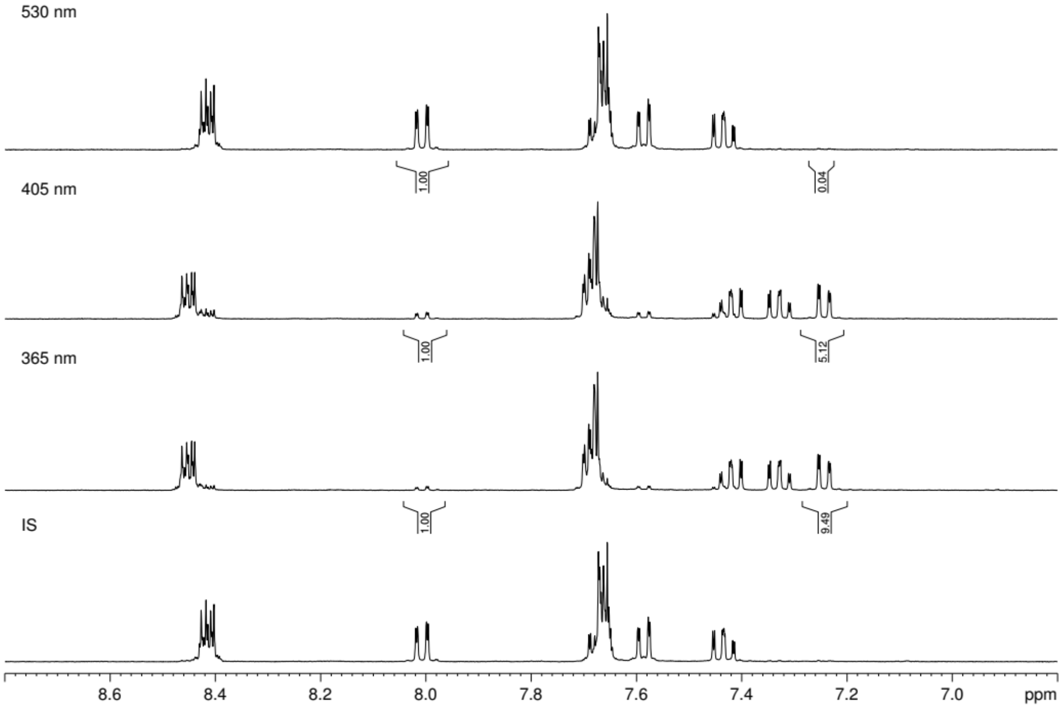

Figure S30. Section from the ^{1}H NMR spectra of the azoarene **2d**: after synthesis (initial state, *trans*/*cis*: 100/0), after UV irradiation with λ = 365 nm (*trans*/*cis*: 10/90), after irradiation with λ = 405 nm (*trans*/*cis:* 16/84) and after irradiation with λ = 530 nm (*trans*/*cis:* 97/3) (CD_2Cl_2, 400 MHz, c = 5 mM).

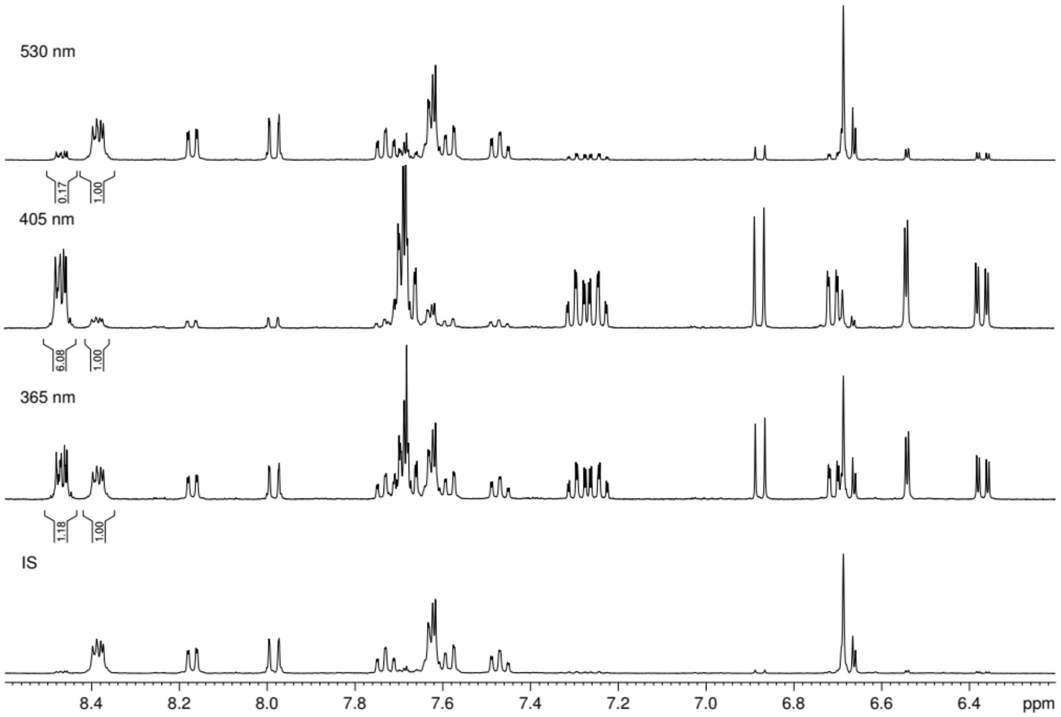

Figure S31. Section from the ^{1}H NMR spectra of the azobenzene **2e**: after synthesis (initial state, *trans*/*cis*: 100/0), after UV irradiation with λ = 365 nm (*trans*/*cis*: 46/54), after irradiation with λ = 405 nm (*trans*/*cis*: 14/86) and after irradiation with λ = 530 nm (*trans*/*cis*: 85/15) (CD_2Cl_2, 400 MHz, c = 5 mM).

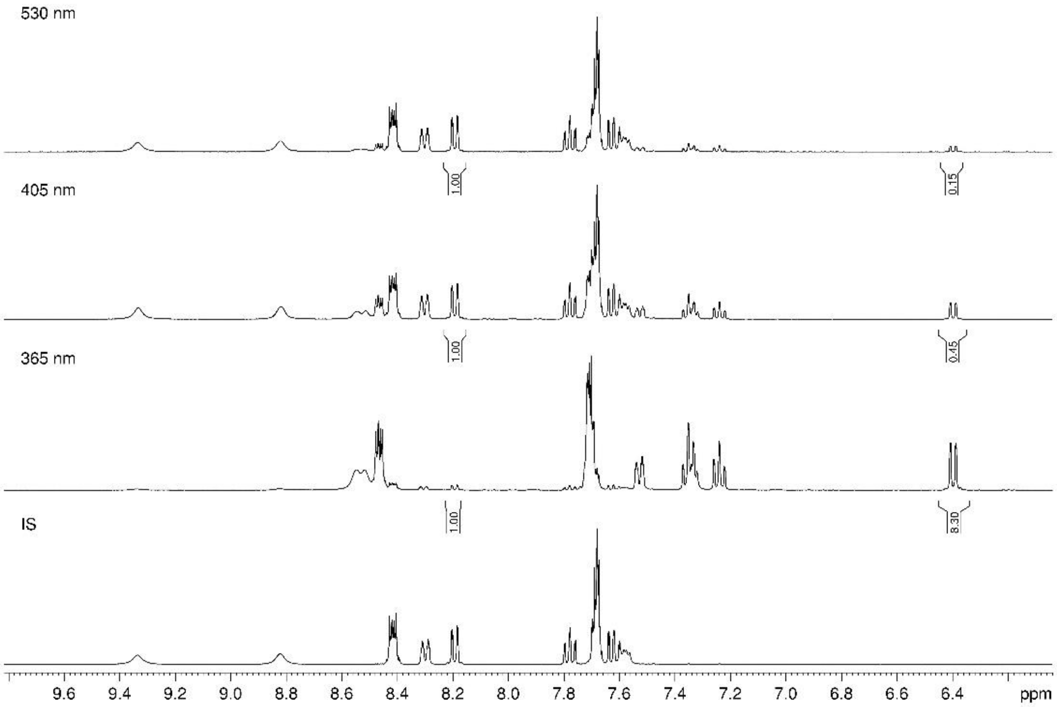

Figure S32. Section from the ^{1}H NMR spectra of the azoarene **2f**: after synthesis (initial state, *trans*/*cis*: 99/1), after UV irradiation with λ = 365 nm (*trans*/*cis*: 11/89), after irradiation with λ = 405 nm (*trans*/*cis*: 70/30) and after irradiation with λ = 530 nm (*trans*/*cis*: 87/13) (CD_2Cl_2, 400 MHz, c = 5 mM).

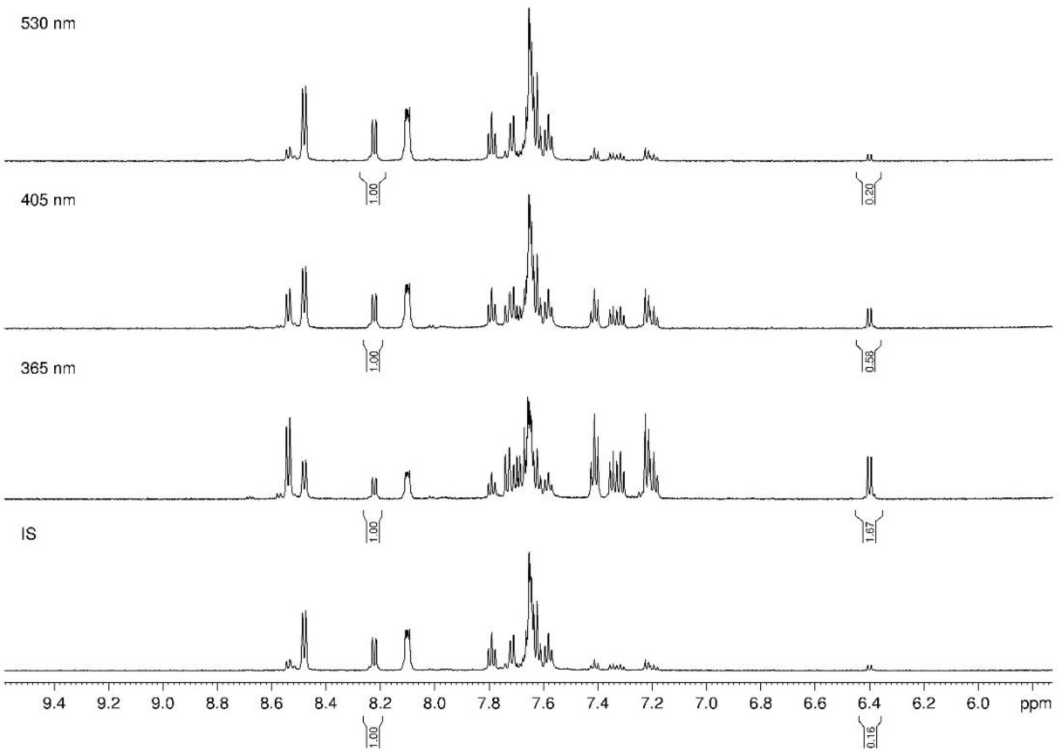

Figure S33. Section from the ^{1}H NMR spectra of the azobenzene **3a**: after synthesis (initial state, *trans*/*cis*: 86/14), after UV irradiation with λ = 365 nm (*trans*/*cis*: 37/63), after irradiation with λ = 405 nm (*trans*/*cis*: 63/37) and after irradiation with λ = 530 nm (*trans*/*cis*: 83/17) (CD_2Cl_2, 400 MHz, c = 5 mM).

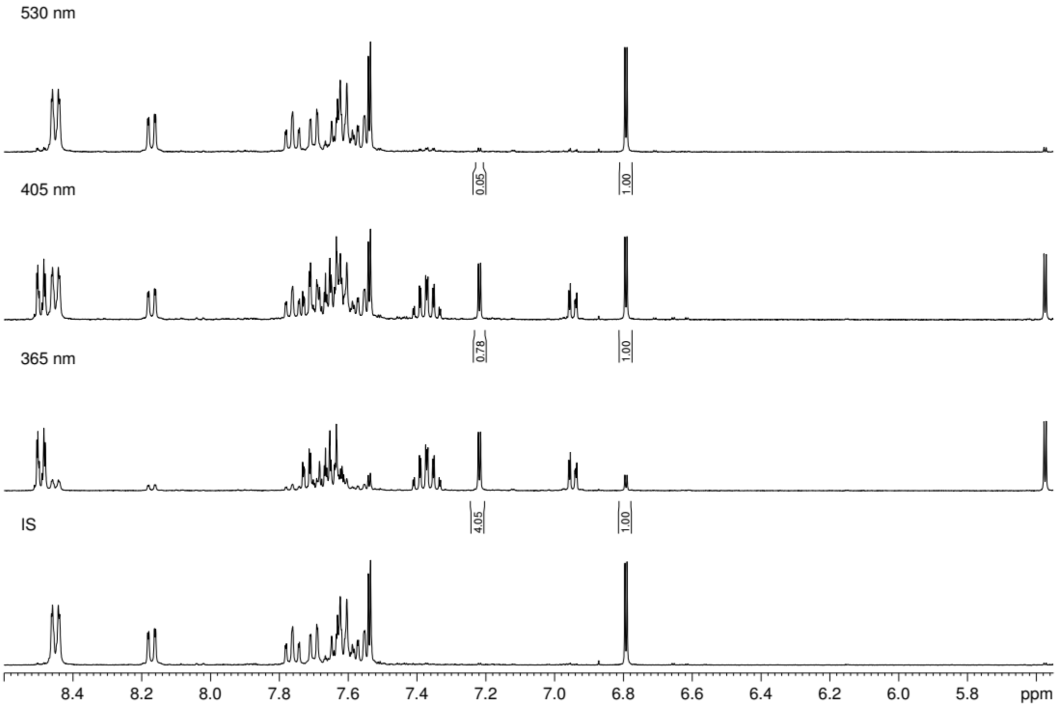

Figure S34. Section from the ^{1}H NMR spectra of the azoarene **3b**: after synthesis (initial state, *trans*/*cis*: 100/0), after UV irradiation with λ = 365 nm (*trans*/*cis:* 20/80), after irradiation with λ = 405 nm (*trans*/*cis:* 56/44) and after irradiation with λ = 530 nm (*trans*/*cis:* 95/5) (CD_2Cl_2, 400 MHz, c = 5 mM).

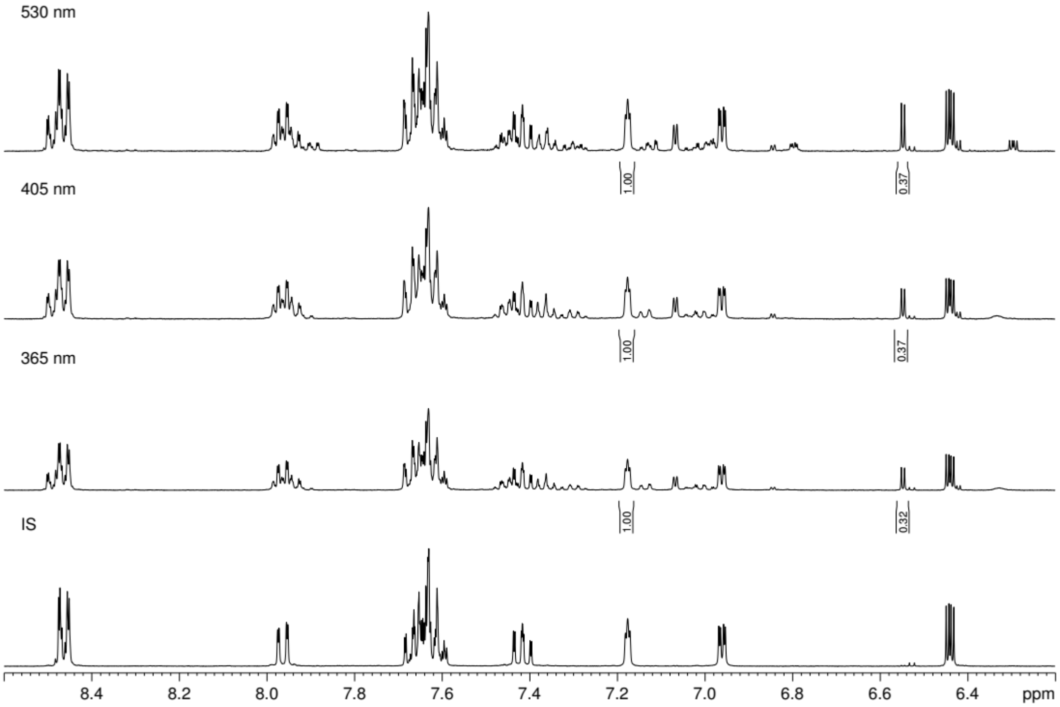

Figure S35. Section from the ^{1}H NMR spectra of the azoarene **3c**: after synthesis (initial state, *trans*/*cis*: 100/0), after UV irradiation with λ = 365 nm, 405 nm and 530 nm (successive decomposition) (CD_2Cl_2, 400 MHz, c = 5 mM).

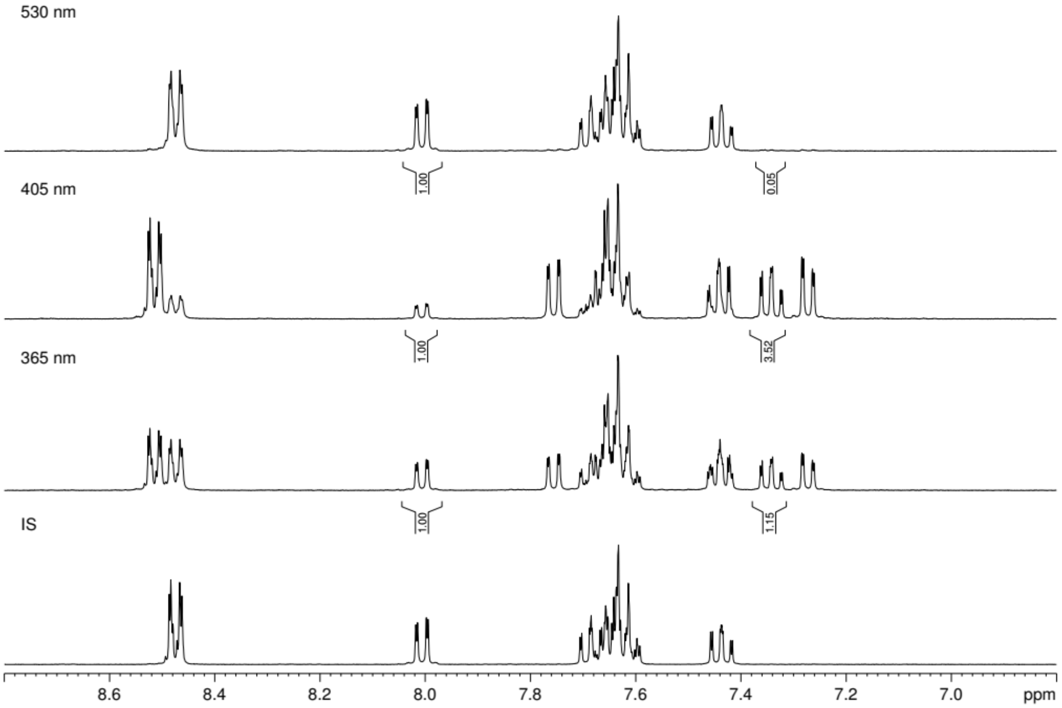

Figure S36. Section from the ^{1}H NMR spectra of the azoarene **3d**: after synthesis (initial state, *trans*/*cis*: 100/0), after UV irradiation with λ = 365 nm (*trans*/*cis*: 47/53), after irradiation with λ = 405 nm (*trans*/*cis:* 22/78) and after irradiation with λ = 530 nm (*trans*/*cis:* 96/4) (CD_2Cl_2, 400 MHz, c = 5 mM).

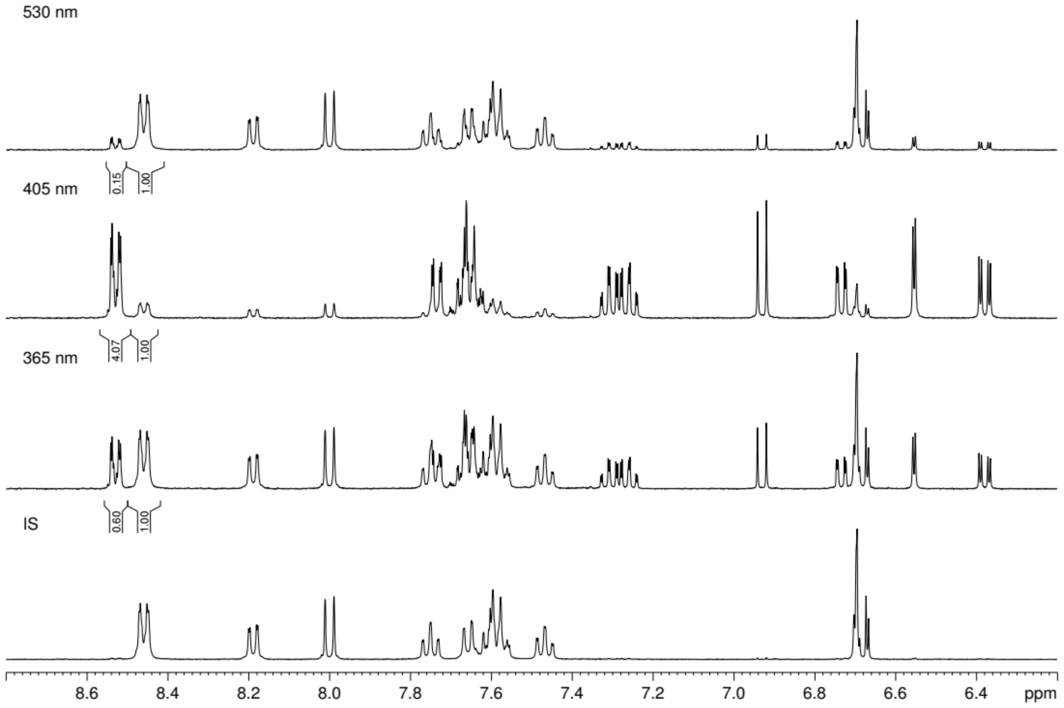

Figure S37. Section from the ^{1}H NMR spectra of the azobenzene **3e**: after synthesis (initial state, *trans*/*cis*: 100/0), after UV irradiation with λ = 365 nm (*trans*/*cis*: 72/38), after irradiation with λ = 405 nm (*trans*/*cis*: 20/80) and after irradiation with λ = 530 nm (*trans*/*cis*: 87/13) (CD_2Cl_2, 400 MHz, c = 5 mM).

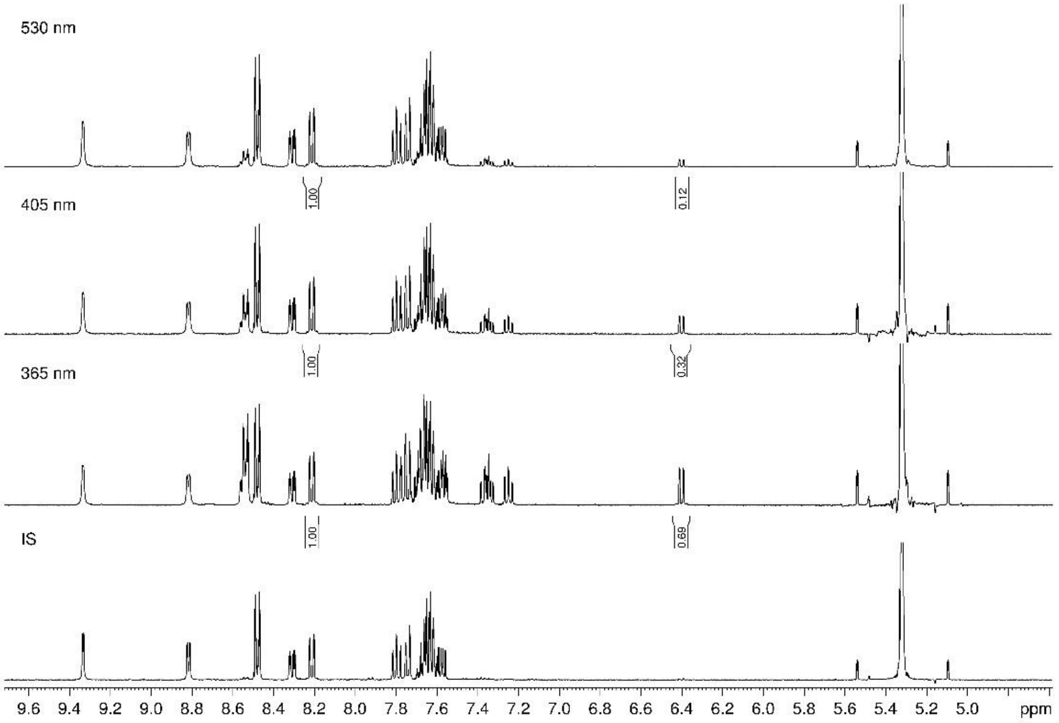

Figure S38. Section from the ^{1}H NMR spectra of the azoarene **3f**: after synthesis (initial state, *trans*/*cis*: 100/0), after UV irradiation with λ = 365 nm (*trans*/*cis*: 59/41), after irradiation with λ = 405 nm (*trans*/*cis:* 76/24) and after irradiation with λ = 530 nm (*trans*/*cis:* 89/11) (CD_2Cl_2, 400 MHz, c = 5 mM).

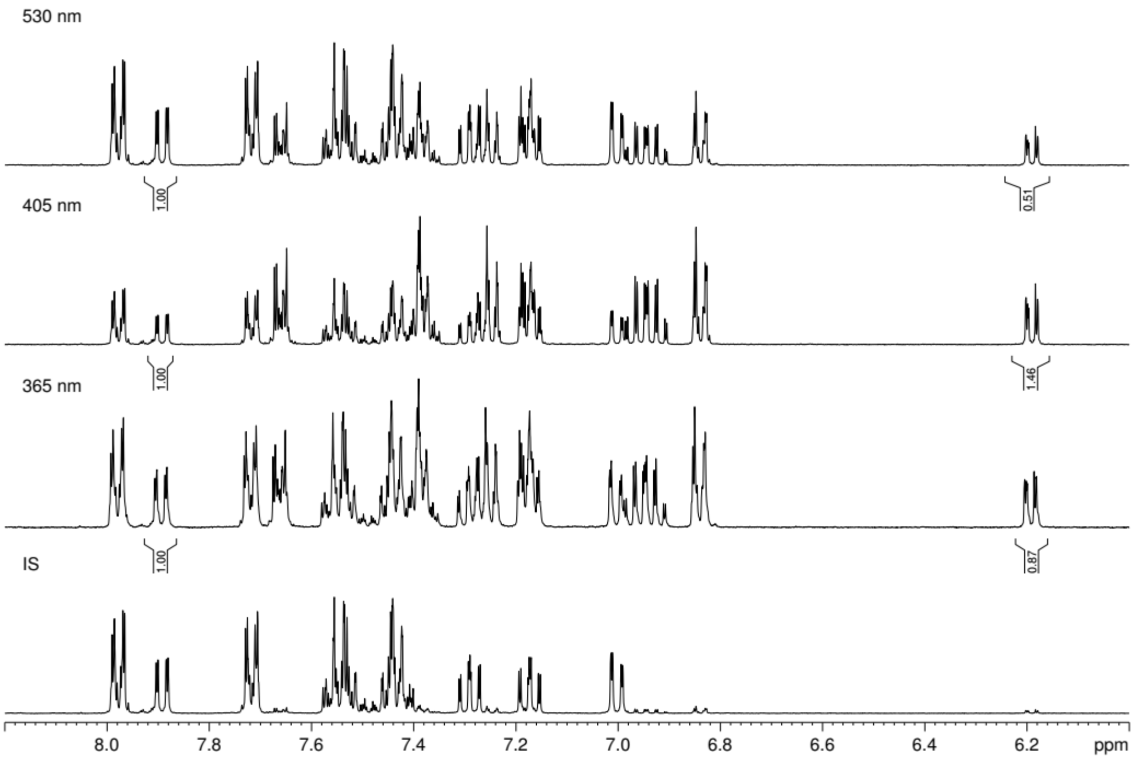

Figure S39. Section from the ^{1}H NMR spectra of the azobenzene **4**: after synthesis (initial state, *trans*/*cis*: 99/1), after UV irradiation with λ = 365 nm (*trans*/*cis:* 53/47), after irradiation with λ = 405 nm (*trans*/*cis:* 41/59) and after irradiation with λ = 530 nm (*trans*/*cis:* 66/34) (CD_2Cl_2, 400 MHz, c = 5 mM).

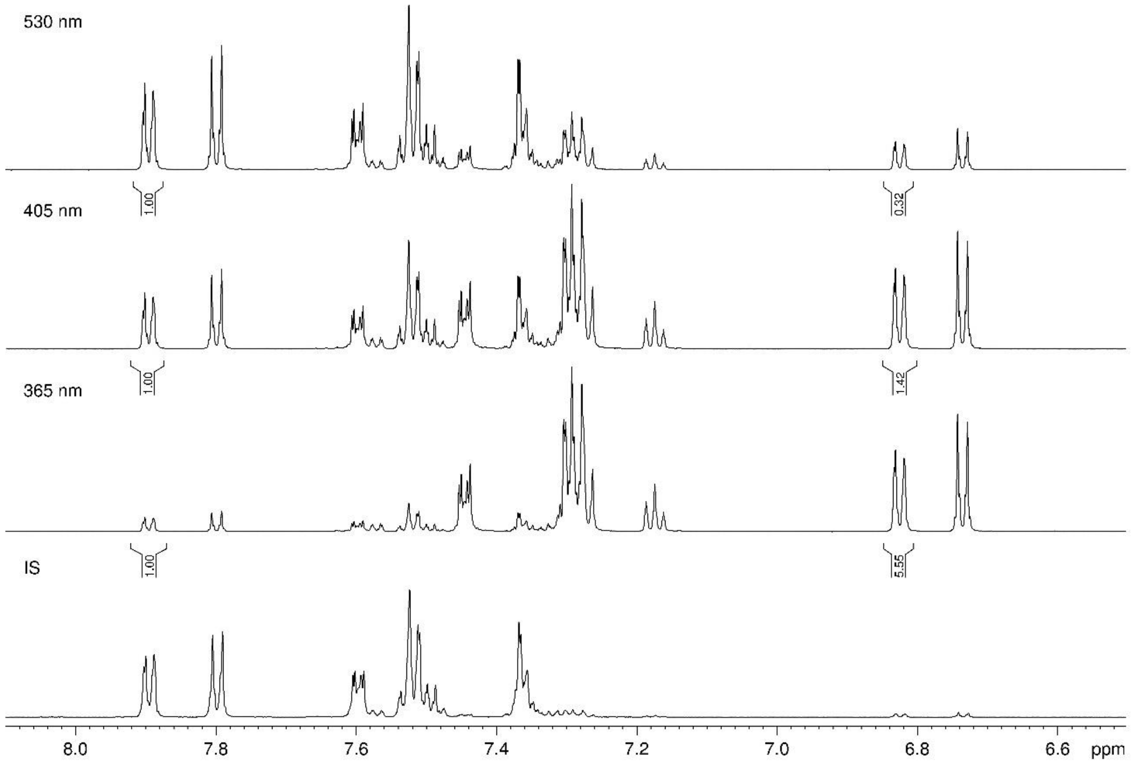

Figure S40. Section from the ^{1}H NMR spectra of the azobenzene **5**: after synthesis (initial state, *trans*/*cis*: 99/1), after UV irradiation with λ = 365 nm (*trans*/*cis*: 15/85), after irradiation with λ = 405 nm (*trans*/*cis:* 41/59) and after irradiation with λ = 530 nm (*trans*/*cis:* 76/24) (CD_2Cl_2, 600 MHz, c = 5 mM).

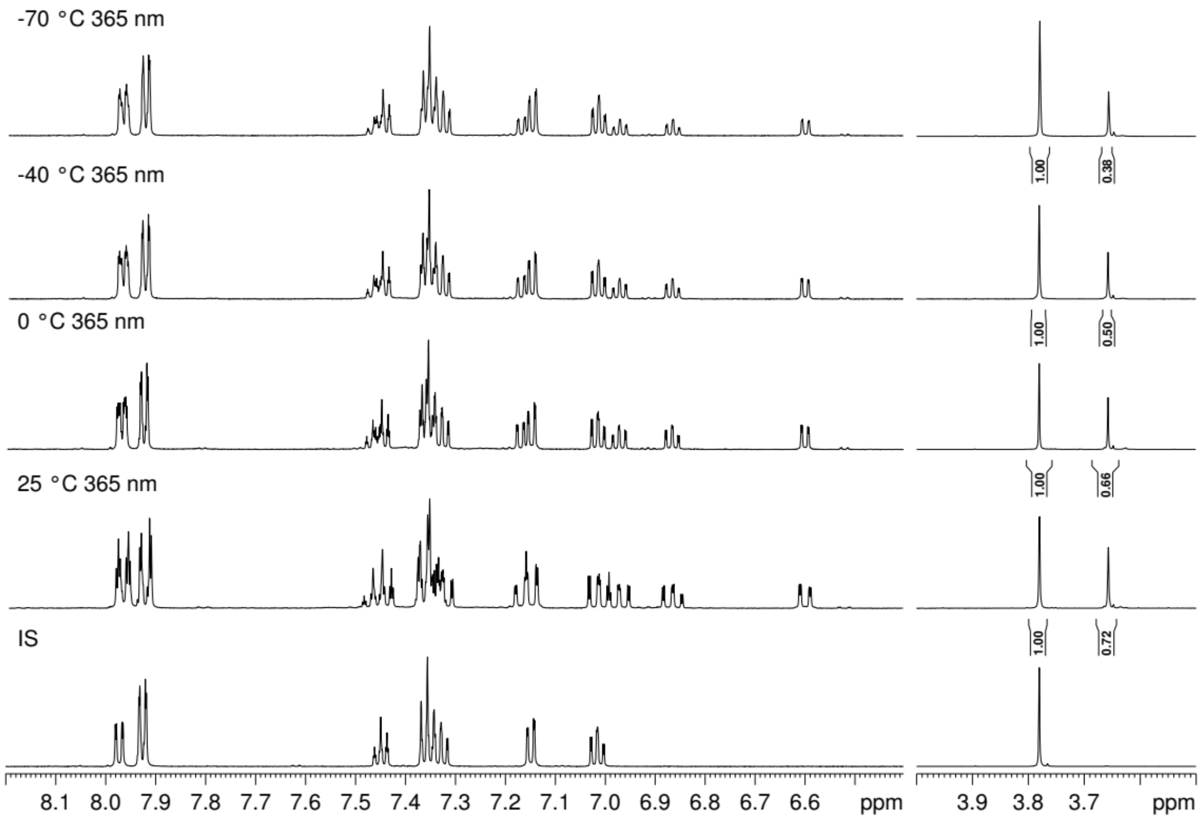

Figure S41. Section from the ^{1}H NMR spectra of the azoarene **1d**: after synthesis (initial state, *trans*/*cis*: 100/0), after UV irradiation with λ = 365 nm at room temperature (*trans*/*cis:* 58/42), 0 °C (*trans*/*cis:* 60/40), -40 °C (*trans*/*cis:* 67/33), -70 °C (*trans*/*cis:* 71/29) (CD_2Cl_2, 600 MHz, c = 5 mM).

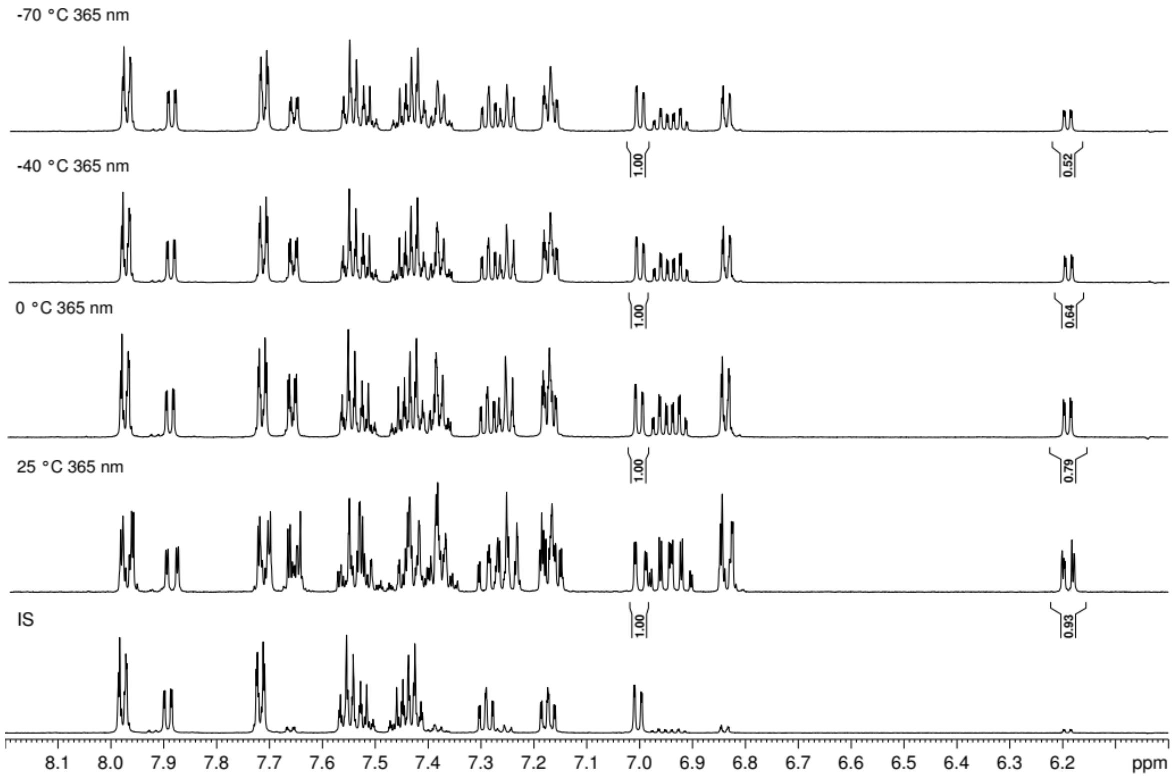

Figure S42. Section from the ^{1}H NMR spectra of the azobenzene **4**: after synthesis (initial state, *trans*/*cis*: 100/0), after UV irradiation with λ = 365 nm at room temperature (*trans*/*cis:* 52/48), 0 °C (*trans*/*cis:* 56/44), -40 °C (*trans*/*cis:* 61/39), -70 °C (*trans*/*cis:* 66/34) (CD_2Cl_2, 600 MHz, c = 5 mM).

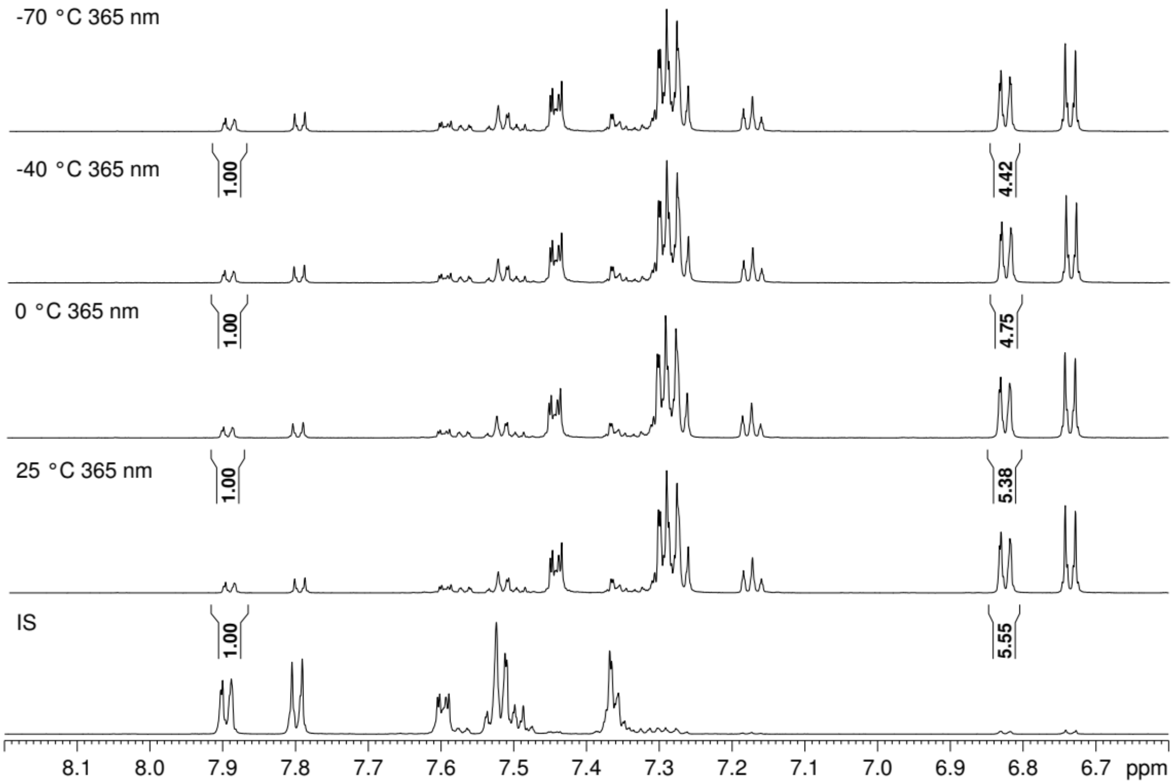

Figure S43. Section from the ^{1}H NMR spectra of the azobenzene **5**: after synthesis (initial state, *trans*/*cis*: 99/1), after UV irradiation with λ = 365 nm at room temperature (*trans*/*cis:* 85/15), 0 °C (*trans*/*cis:* 84/16), -40 °C (*trans*/*cis:* 83/17), -70 °C (*trans*/*cis:* 82/18) (CD_2Cl_2, 600 MHz, c = 5 mM).

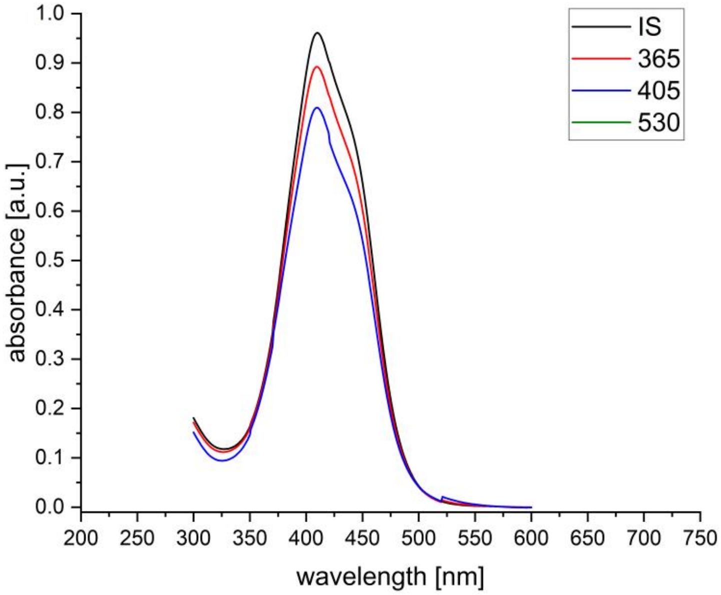

Figure S44. Normalized UV/Vis spectra of the azobenzene **2c**: after synthesis (initial state, black), after UV irradiation with λ = 365 nm (red)and after irradiation with λ = 405 nm (blue) at -10 °C (CH_2Cl_2, c = 1.0 mM).

2. Determination of *cis* Half-Lives

General Procedure. The corresponding compound was dissolved in 0.6 mL of deuterated dichloromethane (c = 5 mM) in a mass vial and irradiated with λ = 365 nm for 60 seconds. The samples were then transferred to an amber glass NMR tube and measured at fixed intervals at room temperature. The *cis/trans* ratio was determined by integration of the NMR signals. The thermal half-lives were calculated from the slope of the linearized plots.

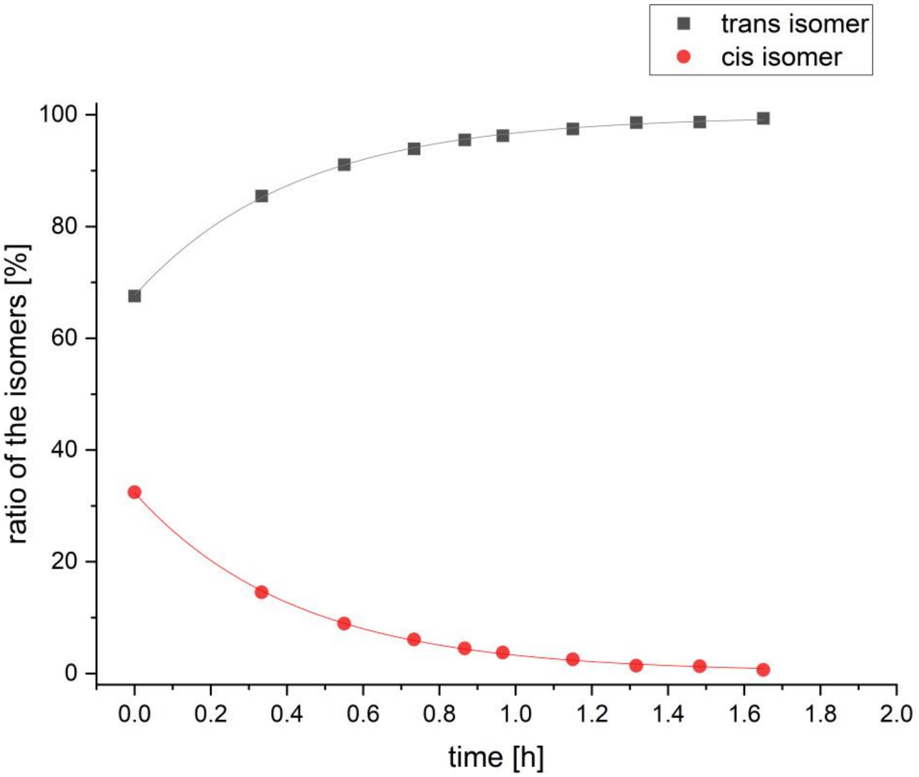

Figure S45. Ratio of the *cis*-**1c** (red) and *trans*-**1c** (grey) after irradiation with UV light (λ = 365 nm) and storage in the dark at 25 °C (CD_2Cl_2, c = 5 mM, $t_{1/2}$ = 0.302 ± 0.001 h).

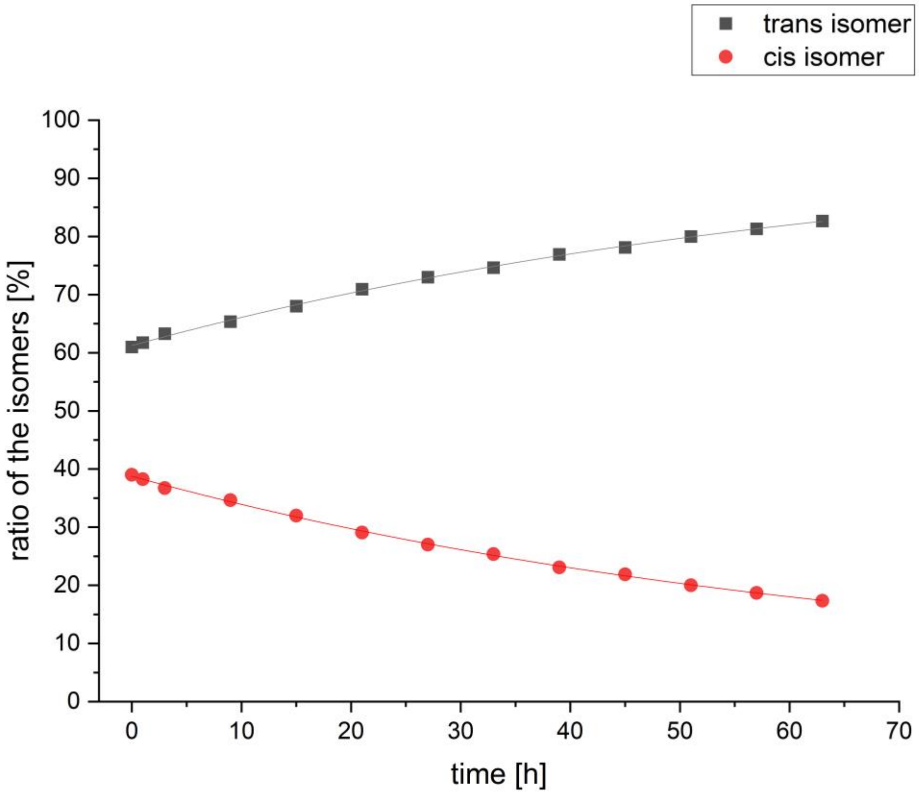

Figure S46. Ratio of the *cis*-**1d** (red) and *trans*-**1d** (grey) after irradiation with UV light (λ = 365 nm) and storage in the dark at 25 °C (CD_2Cl_2, c = 5 mM, $t_{1/2}$ = 54.19 ± 0.09 h).

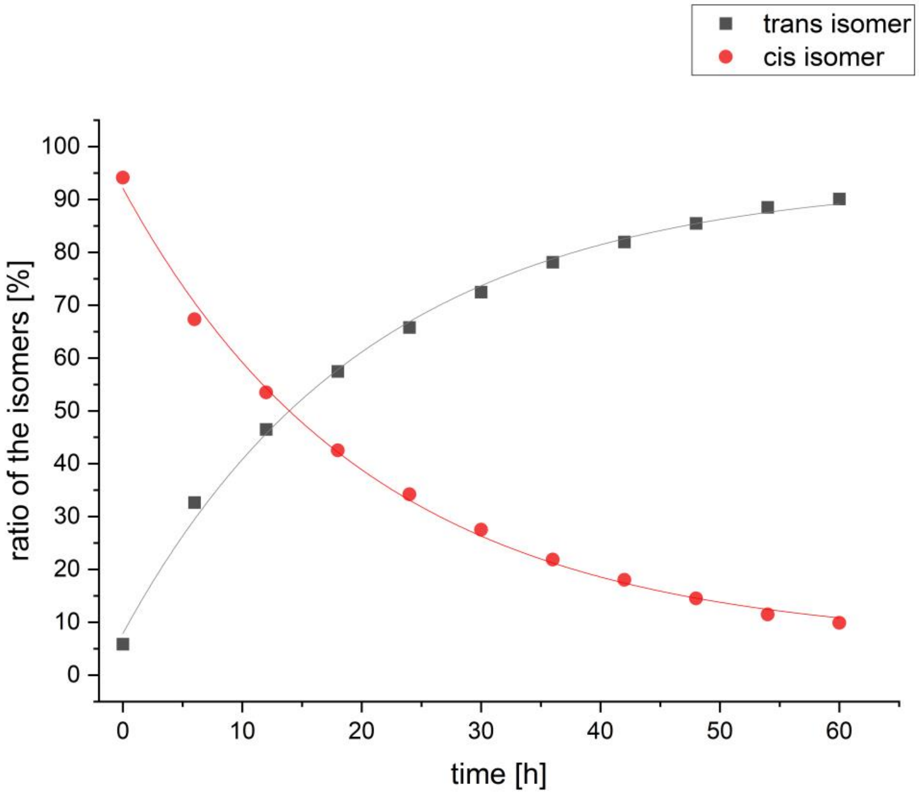

Figure S47. Ratio of the *cis*-**2a** (red) and *trans*-**2a** (grey) after irradiation with UV light (λ = 365 nm) and storage in the dark at 25 °C (CD_2Cl_2, c = 5 mM, $t_{1/2}$ = 18.75 ± 0.03 h).

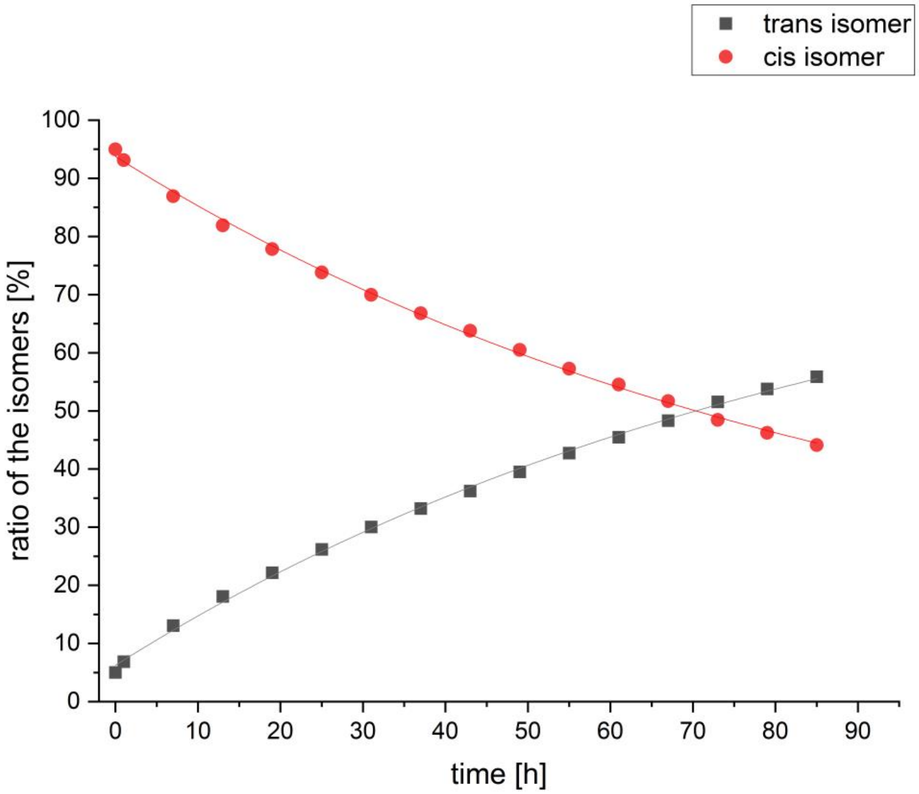

Figure S48. Ratio of the *cis*-**2b** (red) and *trans*-**2b** (grey) after irradiation with UV light (λ = 365 nm) and storage in the dark at 25 °C (CD_2Cl_2, c = 5 mM, $t_{1/2}$ = 78.5 ± 0.13 h).

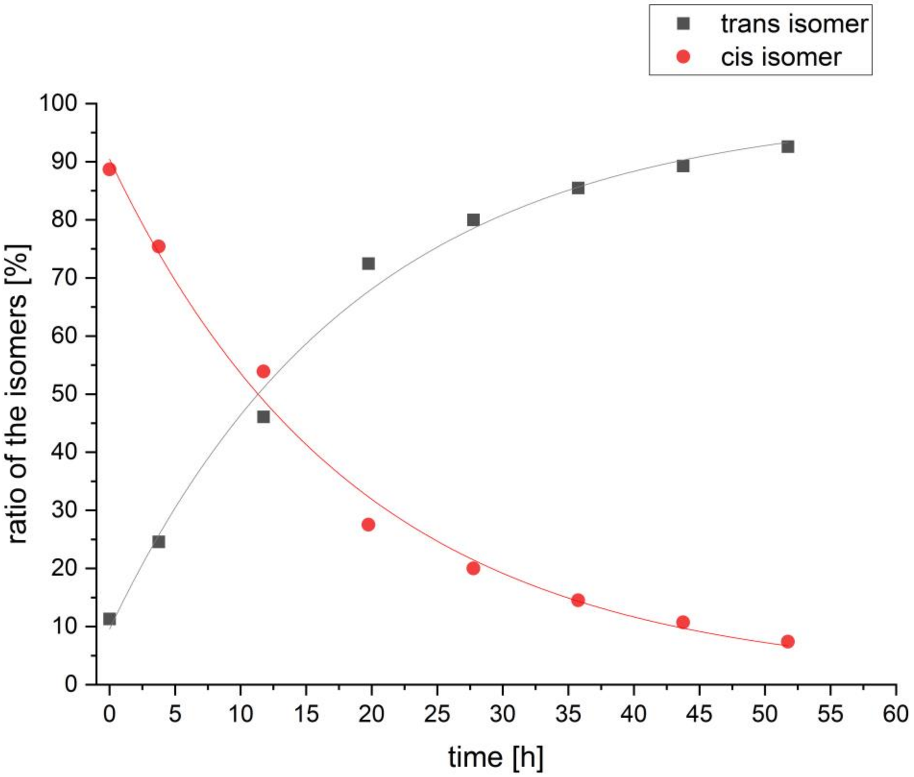

Figure S49. Ratio of the *cis*-**2d** (red) and *trans*-**2d** (grey) after irradiation with UV light (λ = 365 nm) and storage in the dark at 25 °C (CD_2Cl_2, c = 5 mM, $t_{1/2}$ = 14.20 ± 0.02 h).

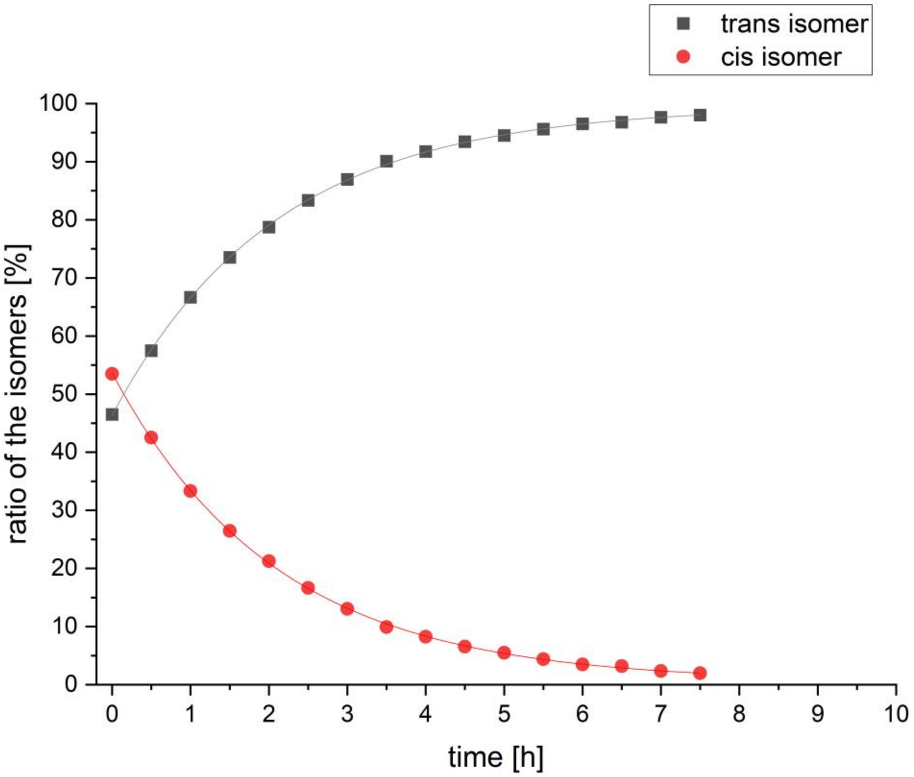

Figure S50. Ratio of the *cis*-**2e** (red) and *trans*-**2e** (grey) after irradiation with UV light (λ = 405 nm) and storage in the dark at 25 °C (CD_2Cl_2, c = 5 mM, $t_{1/2}$ = 1.569 ± 0.003 h).

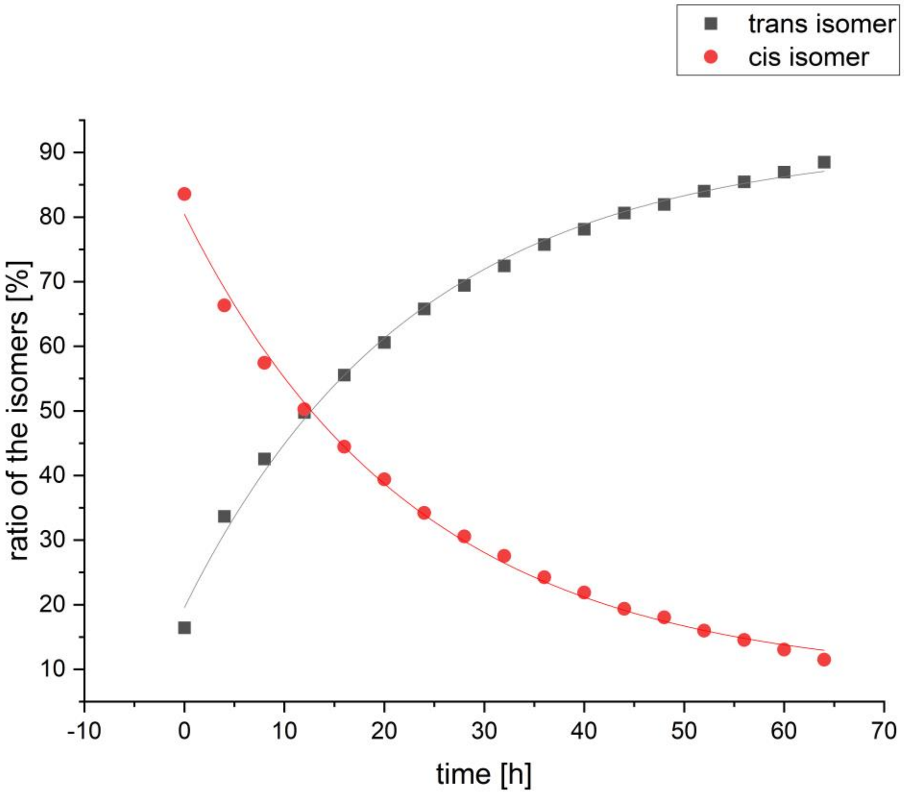

Figure S51. Ratio of the *cis*-**2f** (red) and *trans*-**2f** (grey) after irradiation with UV light (λ = 365 nm) and storage in the dark at 25 °C (CD_2Cl_2, c = 5 mM, $t_{1/2}$ = 23.52 ± 0.04 h).

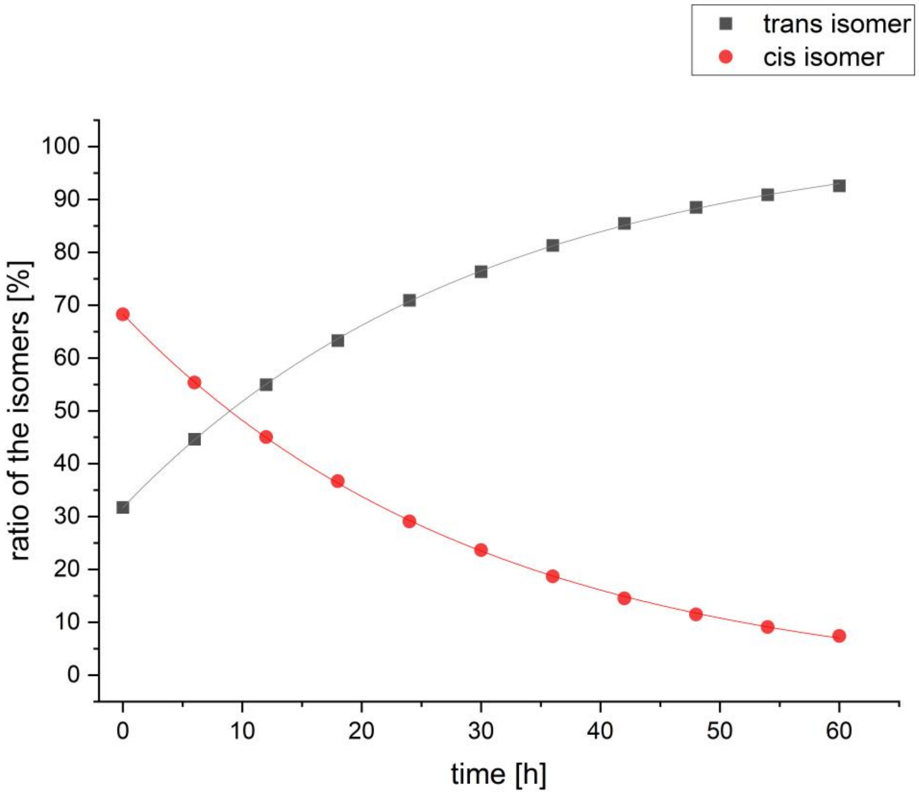

Figure S52. Ratio of the *cis*-**3a** (red) and *trans*-**3a** (grey) after irradiation with UV light (λ = 365 nm) and storage in the dark at 25 °C (CD_2Cl_2, c = 5 mM, $t_{1/2}$ = 18.50 ± 0.03 h).

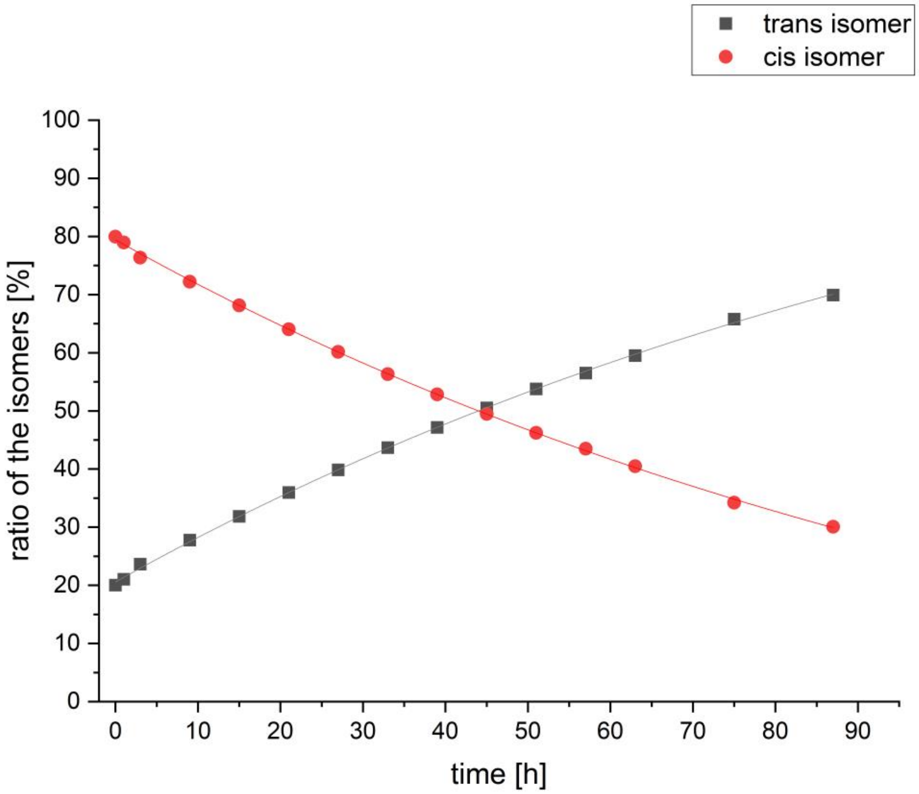

Figure S53. Ratio of the *cis*-**3b** (red) and *trans*-**3b** (grey) after irradiation with UV light (λ = 365 nm) and storage in the dark at 25 °C (CD_2Cl_2, c = 5 mM, $t_{1/2}$ = 62.7 ± 0.11 h).

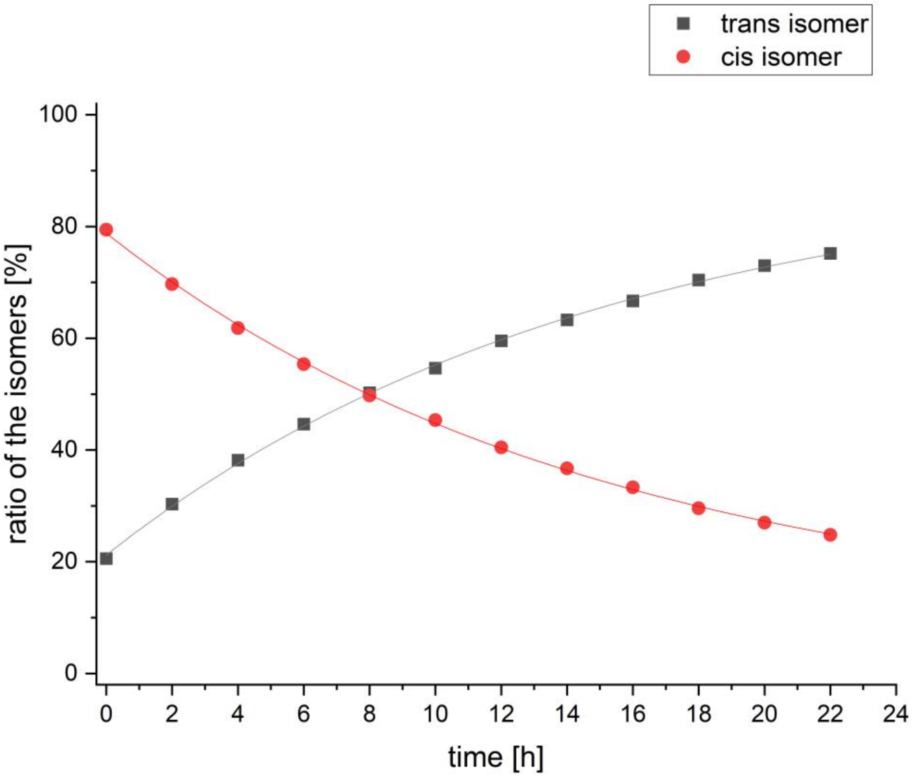

Figure S54. Ratio of the *cis*-**3d** (red) and *trans*-**3d** (grey) after irradiation with UV light (λ = 365 nm) and storage in the dark at 25 °C (CD_2Cl_2, c = 5 mM, $t_{1/2}$ = 13.19 ± 0.02 h).

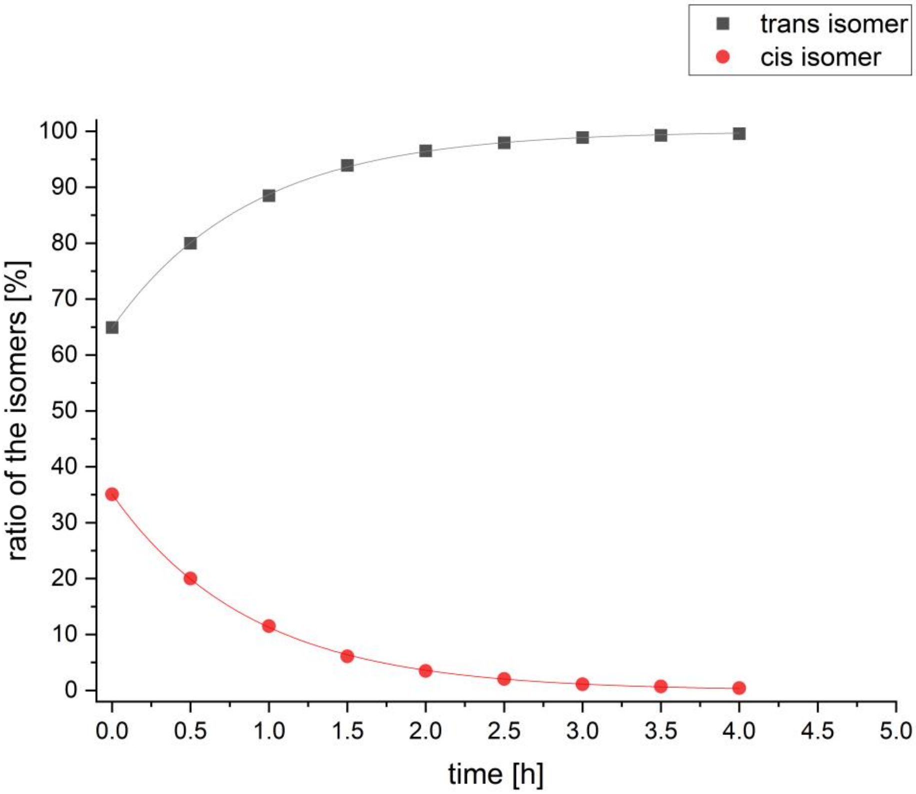

Figure S55. Ratio of the *cis*-**3e** (red) and *trans*-**3e** (grey) after irradiation with UV light (λ = 405 nm) and storage in the dark at 25 °C (CD_2Cl_2, c = 5 mM, $t_{1/2}$ = 0.615 ± 0.001 h).

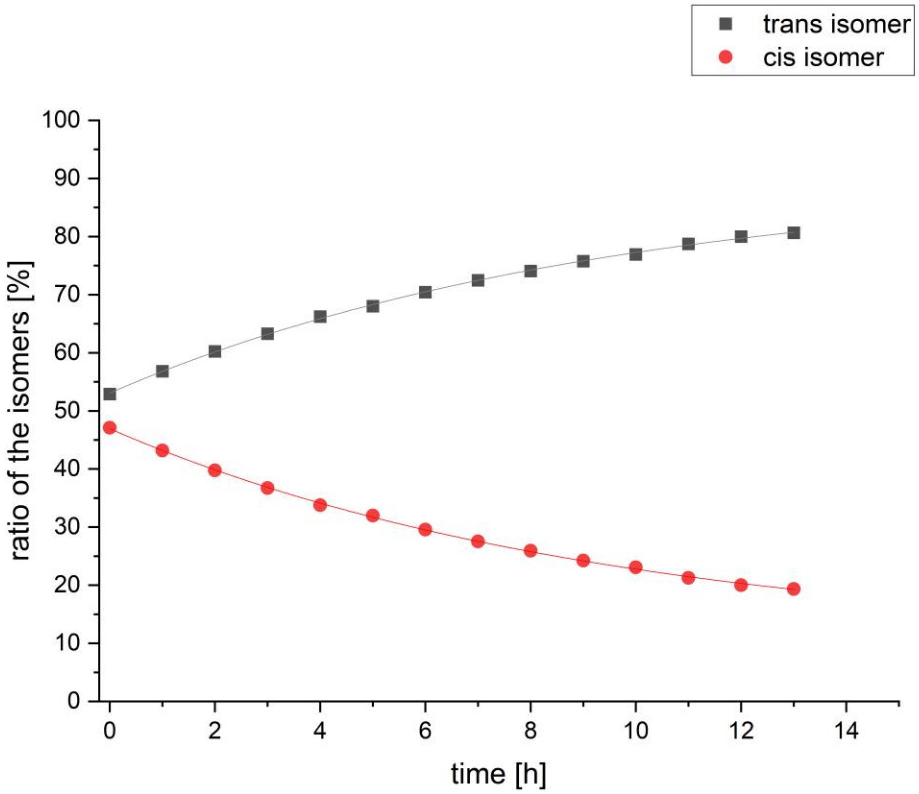

Figure S56. Ratio of the *cis*-**3f** (red) and *trans*-**3f** (grey) after irradiation with UV light (λ = 365 nm) and storage in the dark at 25 °C (CD_2Cl_2, c = 5 mM, $t_{1/2}$ = 10.09 ± 0.02 h).

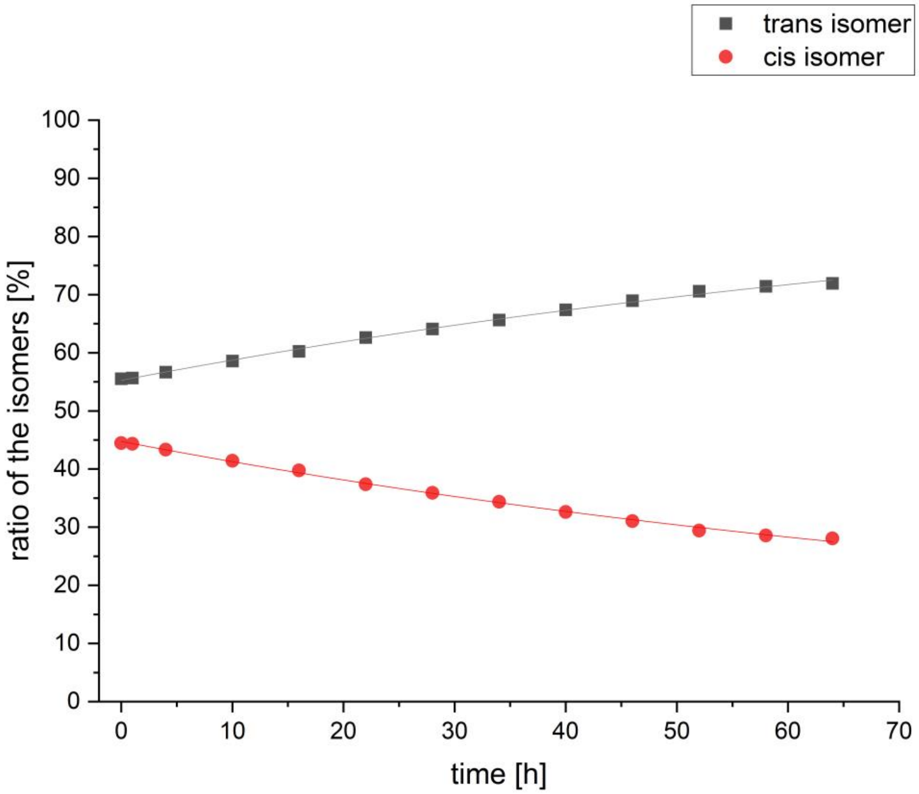

Figure S57. Ratio of the *cis*-**4** (red) and *trans*-**4** (grey) after irradiation with UV light (λ = 365 nm) and storage in the dark at 25 °C (CD_2Cl_2, c = 5 mM, $t_{1/2}$ = 90.7 ± 0.15 h).

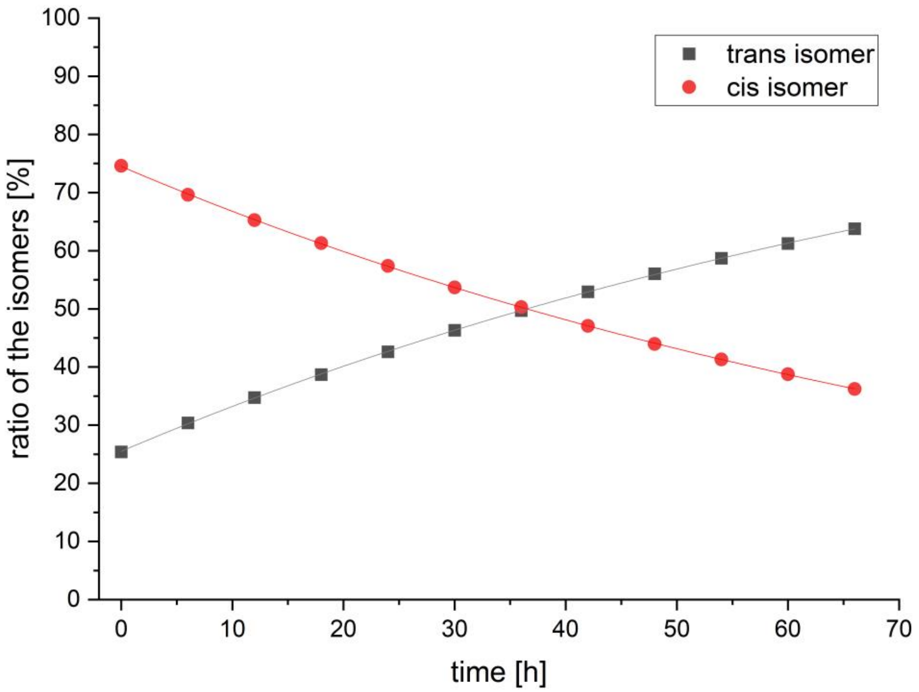

Figure S58. Ratio of the *cis*-**5** (red) and *trans*-**5** (grey) after irradiation with UV light (λ = 365 nm) and storage in the dark at 25 °C (CD_2Cl_2, c = 5 mᴍ, $t_{1/2}$ = 63.5 ± 0.11 h).

3. Photofatigue and Photostability

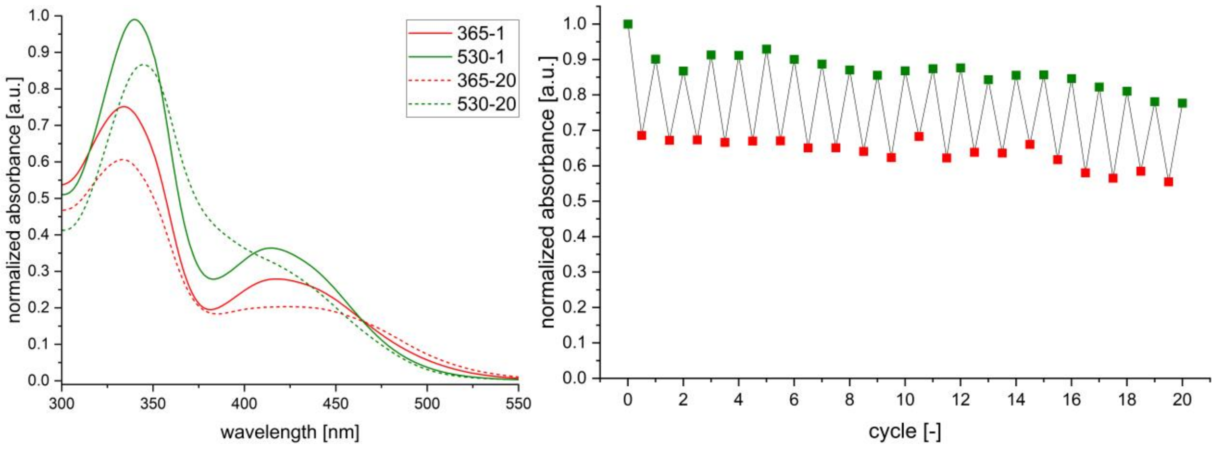

Figure S59. Normalized UV/Vis spectra (CH_2Cl_2, c = 1.0 mM) of compound **1d** (no oxygen exclusion) after the first (solid) and 20th (dashed) irradiation cycle with λ = 365 nm (red) respectively λ = 530 nm (green). Normalized absorbance of *trans*-**1d** (measured at 340 nm) and *cis*-**1d** (measured at 334 nm) after ongoing switching cycles.

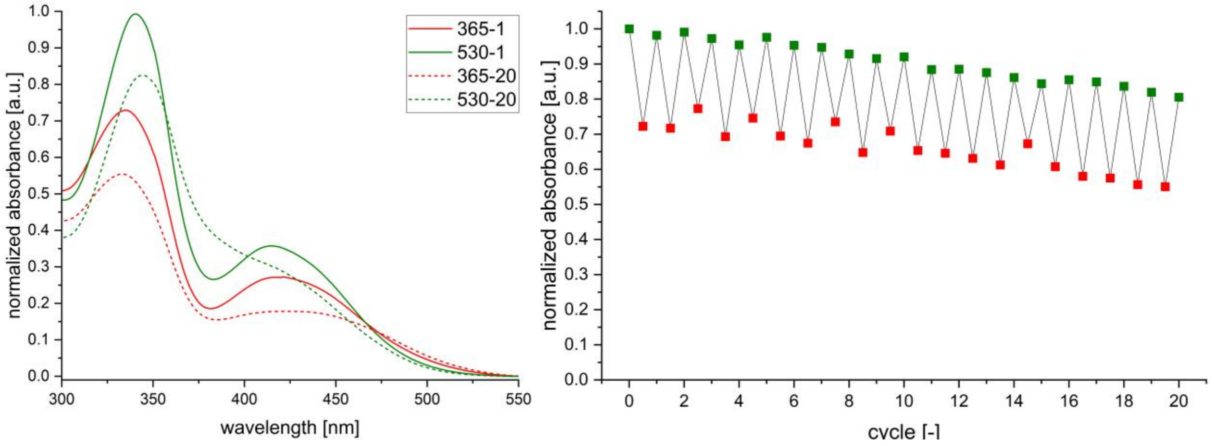

Figure S60. Normalized UV/Vis spectra (CH_2Cl_2, c = 1.0 mM) of compound **1d** (oxygen exclusion) after degassing with argon after the first (solid) and 20th (dashed) irradiation cycle with λ = 365 nm (red) respectively λ = 530 nm (green). Normalized absorbance of *trans*-**1d** (measured at 340 nm) and *cis*-**1d** (measured at 334 nm) after ongoing switching cycles.

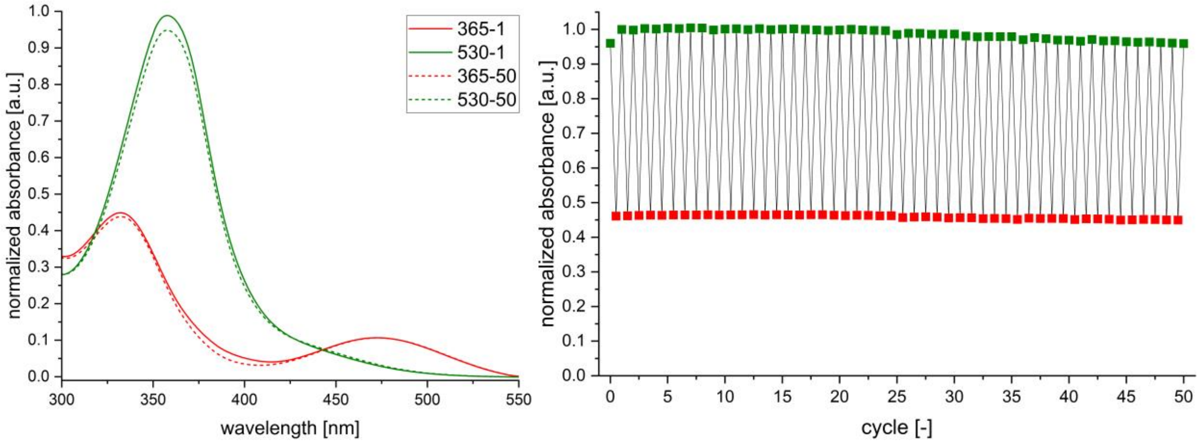

Figure S61. Normalized UV/Vis spectra (CH_2Cl_2, c = 1.0 mM) of compound **2d** (no oxygen exclusion) after the first (solid) and 50th (dashed) irradiation cycle with λ = 365 nm (red) respectively λ = 530 nm (green). Normalized absorbance of *trans*-**2d** (measured at 358 nm) and *cis*-**2d** (measured at 330 nm) after ongoing switching cycles.

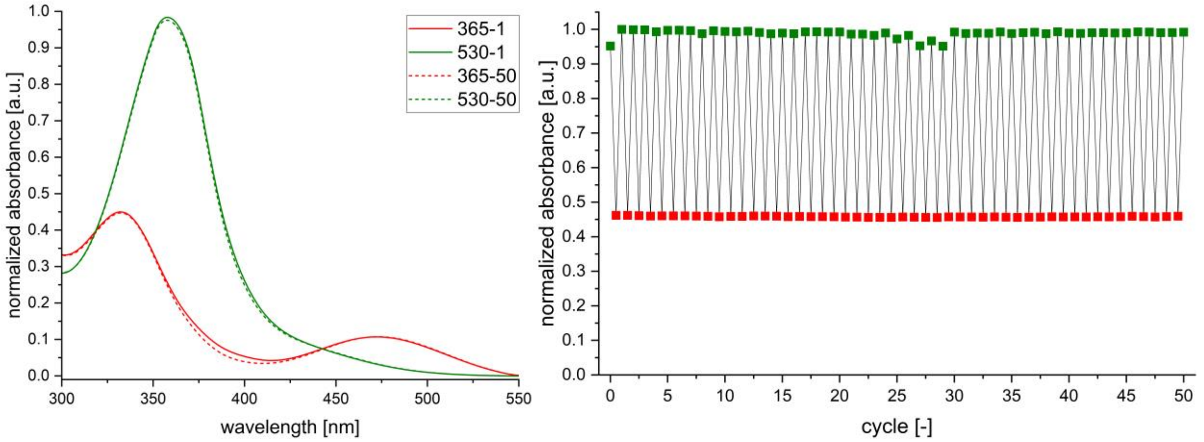

Figure S62. Normalized UV/Vis spectra (CH_2Cl_2, c = 1.0 mM) of compound **2d** (oxygen exclusion) after degassing with argon after the first (solid) and 50th (dashed) irradiation cycle with λ = 365 nm (red) respectively λ = 530 nm (green). Normalized absorbance of *trans*-**2d** (measured at 358 nm) and *cis*-**2d** (measured at 330 nm) after ongoing switching cycles.

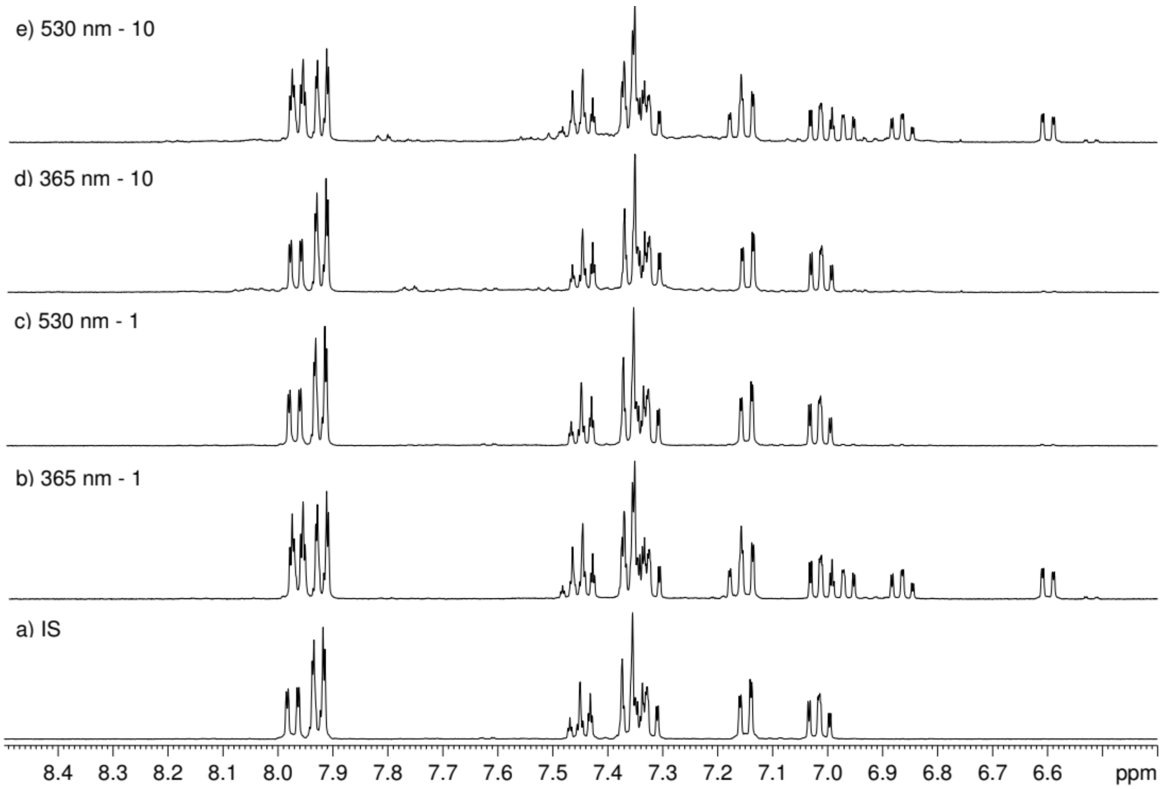

Figure S63. Section from the ^{1}H NMR spectra of the azobenzene **1d** after synthesis (a) and after the first (b and c) and 10th (d and e) irradiation cycle with λ = 365 nm (b and d) respectively λ = 530 nm (c and e) (CD_2Cl_2, 400 MHz, c = 5 mM).

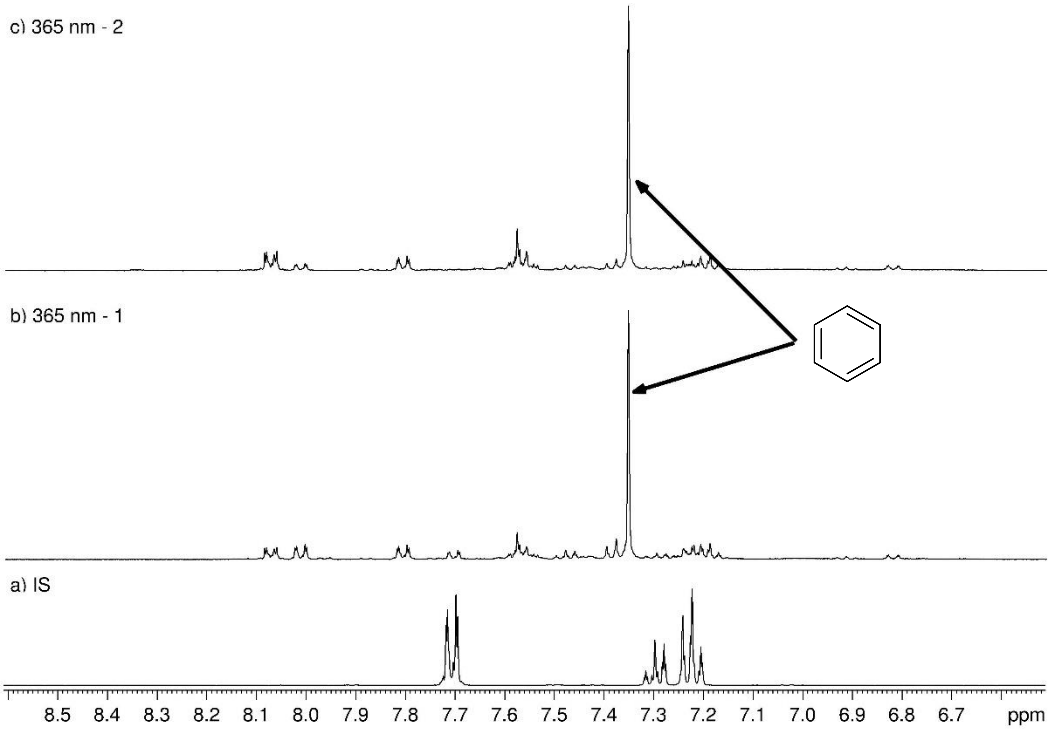

Figure S64. Section from the ^{1}H NMR spectra of the diphenyl telluride (Ph_2Te) after synthesis (a) and after the first (b) and second (c) irradiation cycle with λ = 365 nm (CD_2Cl_2, 400 MHz, c = 5 mM).

4. Hammett Constants

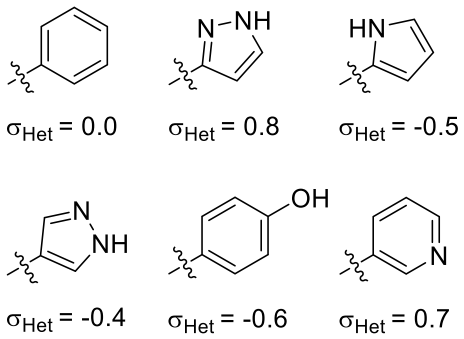

Figure S65. (Hetero)aryl Hammett constants for the parent (hetero)aryl moieties.[1]

5. Synthesis of New Compounds

General Remarks. All chemicals were reagent grade and were used as purchased from ABCR, Alfa Aesar, Acros Organics, Fisher, TCI, or Sigma-Aldrich. Reactions were monitored by TLC analysis with silica gel 60 F254 thin-layer plates. Flash chromatography was carried out on silica 60 (40–63 µm, 230–400 mesh). ^{1}H, ^{13}C, ^{77}Se and ^{125}Te NMR spectra were measured with Bruker Avance NEO 400 and Avance HD 600 spectrometers. All chemical shifts (δ) are given in ppm. The spectra were referenced to the peak for the protium impurity in the deuterated solvents indicated in brackets in the analytical data ($CDCl_3$, ^{1}H: 7.26 ppm, ^{13}C: 77.16 ppm, CD_2Cl_2, ^{1}H: 5.32 ppm, ^{13}C: 53.84 ppm). Signal multiplicity for ^{1}H NMR was determined as s (singlet), d (doublet), t (triplet), dd (doublet of doublets), dt (doublet of triplets) and m (multiplet). ^{13}C NMR spectra were measured with ^{1}H decoupling. The ^{13}C signals were referred to p (primary), s (secondary), t (tertiary), and q (quaternary) carbon atoms. HRMS spectra were recorded with a Bruker BioTOF III mass spectrometer with electrospray ionization (ESI) as ionization source. UV/Vis absorption spectra were obtained with a Jasco V-550 spectrophotometer. IR absorption spectra were recorded with a Shimadzu IR Tracer 100 IR spectrometer. Melting points were measured with a Büchi melting point apparatus Model B-540 with an open capillary and are uncorrected. Compounds **4** and **6a** were synthesized according to literature known procedures.[2-3]

General Procedure (*A*) for the Synthesis of the *ortho* Iodoazoarenes.

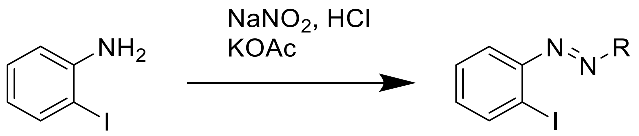

2-Iodoaniline (1.938 g, 8.85 mmol, 1 eq) was dissolved in a mixture of glacial acid (12.5 mL) and hydrochloric acid (2.5 mL). The reaction mixture was cooled in an ice bath and sodium nitrite (0.760 g, 11.01 mmol, 1.24 eq) in water (3 mL) was added and stirred for additional 30 minutes. The (hetero)aryl compound or acetylacetone (11.47 mmol, 1.3 eq) and potassium acetate (2.789 g, 28.2 mmol, 3.18 eq) were dissolved in 10 mL ethanol/water (1:1) and cooled to 0 °C. The diazonium salt solution was added dropwise to this solution and the resulting mixture was stirred for 30 minutes up to 18 hours. Purification depended on the derivate.

General Procedure (*B*) for the Synthesis of *ortho*-tellurated Azoarenes.

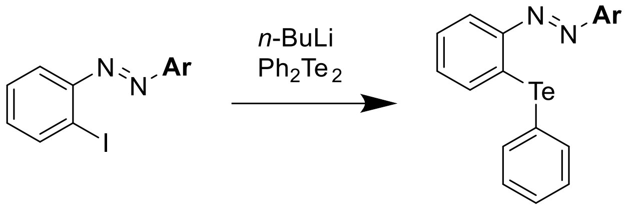

4 **1**

To a solution of aryl iodide (0.15 mmol, 1 eq) in 8 mL dry diethyl ether, *n*-butyllithium in hexane (2.5 M, 0.1 mL) was added at -78 °C under argon atmosphere and the mixture was stirred at this temperature for 10 minutes. This step was followed by dropwise addition of a solution of diphenyl ditelluride (0.15 mmol, 1 eq) in dry diethyl ether (8 mL). The resulting mixture was stirred at -78 °C for 1-2 h. The reaction mixture was allowed to reach room temperature and was then washed with saturated $NaHCO_3$. The organic layer was dried over $MgSO_4$ and concentrated in vacuo. The residue was purified by flash column chromatography with silica gel to provide the desired azoarene.

General Procedure (*C*) for Chlorination of Diaryltellurium Compounds.

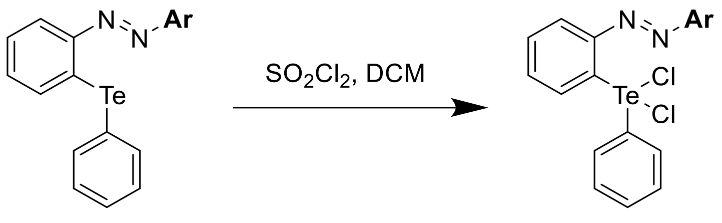

1 2

The diary tellurium compound (0.1 mmol, 1 eq) was dissolved in 20 mL dichloromethane and cooled to 0 °C. After the addition of 0.15 mL sulfuryl chloride solution (1 M in dichloromethane, 0.15 mmol, 1.5 eq) the reaction mixture was stirred for one hour. The solvent was removed in vacuo to give the desired product.

General Procedure (*D*) for Bromination of Diaryltellurium Compounds.

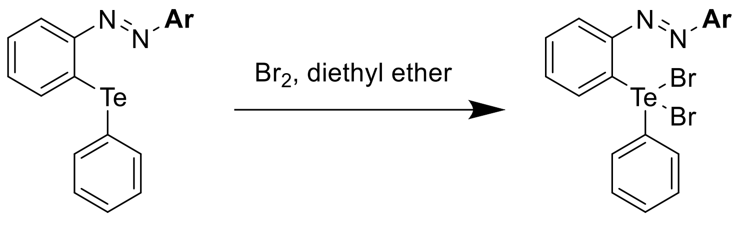

1 3

The diary tellurium compound (0.1 mmol, 1 eq) was dissolved in 20 mL diethyl ether and cooled to 0 °C. After the addition of bromine (16 mg, 5.2 uL, 1 eq) the reaction mixture was stirred for one hour. The precipitation was filtered off and dried in vacuo.

Synthesis of 6b:

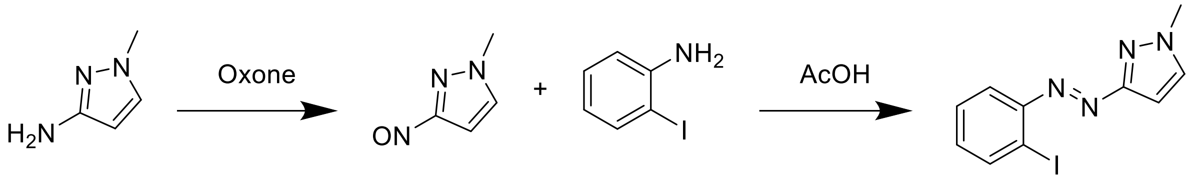

Aminomethylpyrazole (**7**, 729 mg, 7.0 mmol, 1 eq) was dissolved in 15 mL dichloromethane. Oxone (**8**, 9600 mg, 63.2 mmol, 9 eq) was dissolved in 100 mL water. The two-phase mixture was stirred for 10 minutes at room temperature. The organic layer was separated and dried in vacuo. 2-Iodoaniline (**9**, 550 mg, 2.5 mmol, 0.4 eq) and the nitroso compound were dissolved in 75 mL glacial acid and stirred for multiple days. The glacial acid was removed in vacuo and the crude product was purified via column chromatography.

Yield: 298 mg, 0.96 mmol, 38%

R_f (DCM) = 0.5

M.p.: 109–110 °C

^{1}H NMR (400 MHz, $CDCl_3$): δ = 8.01 (dd, $^3J_{H,H}$ = 7.9 Hz, $^4J_{H,H}$ = 1.1 Hz, 1 H, $C_{ar}H$), 7.74 (dd, $^3J_{H,H}$ = 8.0 Hz, $^4J_{H,H}$ = 1.5 Hz, 1 H, $C_{ar}H$), 7.44 −7.37 (m, 2 H, $C_{ar}H$), 7.15 (dt, $^3J_{H,H}$ = 7.5 Hz, $^4J_{H,H}$ = 1.6 Hz, 1 H, $C_{ar}H$), 6.70 (d, $^3J_{H,H}$ = 2.5 Hz, 1 H, $C_{ar}H$), 4.04 ppm (s, 3 H, N-CH_3).

^{13}C NMR (151 MHz, $CDCl_3$): δ = 163.9 (q, C_{ar}), 151.5 (q, C_{ar}), 139.9 (t, $C_{ar}H$), 132.4 (t, $C_{ar}H$), 132.0 (t, $C_{ar}H$), 129.1 (t, $C_{ar}H$), 117.6 (t, $C_{ar}H$), 102.8 (q, C_{ar}), 96.0 (t, $C_{ar}H$), 39.9 ppm (p, N-CH_3).

IR (ATR): $\tilde{\nu}$ = 2970, 1570, 1466, 1456, 1437, 1418, 1389, 1362, 1304, 1207, 1055, 1016, 991, 957, 903, 783, 719, 700, 644, 621 cm^{-1}.

UV/Vis (CH_3CN): λ_{max} (log ε) = 327 nm (3.10).

HRMS (ESI): [$C_{10}H_9N_4I$+H^+]: calculated: 312.9945; observed: 312.9942.

Synthesis of 6c:

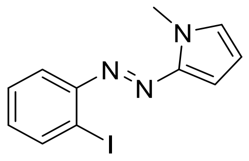

6c

Azoarene **6c** was synthesized according to general procedure (*A*). The reaction mixture was extracted with ethyl acetate and the combined organic phases were dried over magnesium sulfate. The solvent was removed in vacuo. The crude product was purified via column chromatography.

Yield: 2200 mg, 7.1 mmol, 80%

R_f (*n*-Hexane/DCM 2:1) = 0.4

^{1}H NMR (400 MHz, $CDCl_3$): δ = 7.97 (dd, $^3J_{H,H}$ = 7.9 Hz, $^4J_{H,H}$ = 1.1 Hz, 1 H, $C_{ar}H$), 7.60 (dd, $^3J_{H,H}$ = 8.1 Hz, $^4J_{H,H}$ = 1.5 Hz, 1 H, $C_{ar}H$), 7.37 (dt, $^3J_{H,H}$ = 7.6 Hz, $^4J_{H,H}$ = 1.2 Hz 1 H, $C_{ar}H$), 7.05 (dt, $^3J_{H,H}$ = 7.7 Hz, $^4J_{H,H}$ = 1.6 Hz 1 H, $C_{ar}H$), 6.97−6.94 (m, 1 H, $C_{ar}H$), 6.92−6.88 (m, 1 H, $C_{ar}H$), 6.34−6.31 (m, 1 H, $C_{ar}H$), 4.00 ppm (s, 3 H, N-CH_3).

^{13}C NMR (151 MHz, $CDCl_3$): δ = 152.6 (q, C_{ar}), 146.2 (q, C_{ar}), 145.9 (t, $C_{ar}H$), 139.8 (t, $C_{ar}H$), 130.5 (t, $C_{ar}H$), 128.8 (t, $C_{ar}H$), 128.4 (t, $C_{ar}H$), 117.0 (t, $C_{ar}H$), 110.7 (t, $C_{ar}H$), 101.2 (q, C_{ar}), 34.9 ppm (p, CH_3).

IR (ATR): $\tilde{\nu}$ = 3053, 2918, 1575, 1510, 1491, 1433, 1400, 1344, 1321, 1246, 1209, 1167, 1088, 1049, 1016, 997, 935, 880, 860, 851, 806, 793, 760, 738, 714, 694, 650 cm^{-1}.

UV/Vis (CH_2Cl_2): λ_{max} (log ε) = 394 nm (3.13).

HRMS (ESI): [$C_{16}H_{11}N_2OI+H^+$]: calculated: 311.9992; observed: 311.9993.

Synthesis of 10:

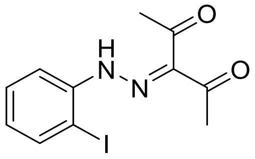

10

Azo compound **10** was synthesized according to general procedure (*A*). The crude product was filtered off, washed with water, and used without further purification.

Yield: 2208 mg, 6.7 mmol, 76%

M.p.: 170–171 °C

^{1}H NMR (400 MHz, $CDCl_3$): δ = 14.77 (s, 1 H, N−H), 7.82 (dd, $^{3}J_{H,H}$ = 8.0 Hz, $^{4}J_{H,H}$ = 1.2 Hz, 1 H, C_{ar}H), 7.73 (dd, $^{3}J_{H,H}$ = 8.2 Hz, $^{4}J_{H,H}$ = 1.2 Hz, 1 H, C_{ar}H), 7.42 (t, $^{3}J_{H,H}$ = 7.6 Hz, 1 H, C_{ar}H), 6.93 (dt, $^{3}J_{H,H}$ = 7.6 Hz, $^{4}J_{H,H}$ = 1.5 Hz, 1 H, C_{ar}H), 2.64 (s, 3 H, CH_3), 2.51 ppm (s, 3 H, CH_3).

^{13}C NMR (151 MHz, $CDCl_3$): δ = 197.8 (q, C_{ar}), 197.3 (q, C_{ar}), 142.4 (q, C_{ar}), 139.7 (t, C_{ar}H), 134.2 (q, C_{ar}), 129.7 (t, C_{ar}H), 127.0 (t, C_{ar}H), 116.9 (t, C_{ar}H), 85.5 (q, C_{ar}), 31.8 (p, C_{ar}H), 26.84 ppm (p, C_{ar}H).

IR (ATR): $\tilde{\nu}$ = 3065, 1666, 1622, 1570, 1500, 1406, 1352, 1315, 1273, 1256, 1175, 1015, 984, 926, 864, 833, 781, 748, 638, 625 cm^{-1}.

UV/Vis (CH_3CN): λ_{max} (log ε) = 371 nm (3.29).

HRMS (ESI): [$C_{11}H_{11}N_2O_2I+H^{+}$]: calculated: 330.9938; observed: 330.9935.

Synthesis of 6d:

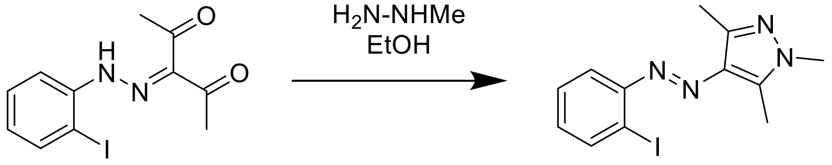

10 **6d**

Diketone **6d** (1000 mg, 3.03 mmol, 1 eq) was suspended in 15 mL ethanol and methylhydrazine (0.16 mL, 3.21 mmol, 1.05 eq) was added dropwise. The reaction mixture was stirred at room temperature for two hours. The solvent was removed in vacuo and the product was used without further purification.

Yield: 1005 mg, 3.0 mmol, 97%

M.p.: 126–127 °C

^{1}H NMR (400 MHz, $CDCl_3$): δ = 7.98 (dd, $^3J_{H,H}$ = 8.0 Hz, $^4J_{H,H}$ = 1.1 Hz, 1 H, $C_{ar}H$), 7.61 (dd, $^3J_{H,H}$ = 8.1 Hz, $^4J_{H,H}$ = 1.4 Hz, 1 H, $C_{ar}H$), 7.39 (dt, $^3J_{H,H}$ = 7.5 Hz, $^4J_{H,H}$ = 1.1 Hz, 1 H, $C_{ar}H$), 7.07 (dt, $^3J_{H,H}$ = 7.6 Hz, $^4J_{H,H}$ = 1.5 Hz, 1 H, $C_{ar}H$), 3.80 (s, 3 H, N-CH_3), 2.63 (s, 3 H, CH_3), 2.59 ppm (s, 3 H, CH_3).

^{13}C NMR (151 MHz, $CDCl_3$): δ = 152.8 (q, C_{ar}), 143.4 (q, C_{ar}), 139.8 (t, $C_{ar}H$), 139.8 (t, $C_{ar}H$), 135.6 (t, $C_{ar}H$), 130.7 (t, $C_{ar}H$), 128.8 (t, $C_{ar}H$), 116.9 (t, $C_{ar}H$), 101.2 (q, $C_{ar}H$), 36.2 (p, N-CH_3), 14.5 (p, CH_3) 10.6 ppm (p, CH_3).

IR (ATR): $\tilde{\nu}$ = 2943, 1962, 1547, 1520, 1452, 1423, 1389, 1369, 1298, 1256, 1234, 1192, 1153, 1107, 1040, 1013, 991, 953, 889, 858, 787, 766, 719, 667, 640 cm^{-1}.

UV/Vis (CH_3CN): λ_{max} (log ε) = 351 (3.03), 425 nm (1.98).

HRMS (ESI): [$C_{12}H_{13}N_4I$+H $^+$]: calculated: 341.0258; observed: 341.0258.

Synthesis of 11:

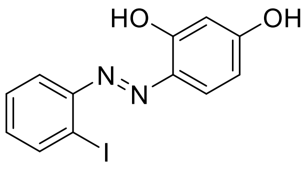

11

Azobenzene **11** was synthesized according to general procedure (*A*). The reaction mixture was extracted with dichloromethane and the combined organic phases were dried over magnesium sulfate. The solvent was removed in vacuo. The crude product was purified via column chromatography.

Yield: 2040 mg, 6.0 mmol, 67%

R_f (DCM/ethyl acetate 9:1) = 0.8

M.p.: 176 °C

^{1}H NMR (400 MHz, DMSO-d$_6$): δ = 12.65 (s, 1 H, OH), 10.80 (s, 1 H, OH), 8.06 (dd, $^3J_{H,H}$ = 7.9 Hz, $^4J_{H,H}$ = 1.2 Hz, 1 H, C_{ar}H), 7.79–7.72 (m, 2 H, C_{ar}H), 7.53 (dt, $^3J_{H,H}$ = 7.6 Hz, $^4J_{H,H}$ = 1.3 Hz, 1 H, C_{ar}H), 7.24 (dt, $^3J_{H,H}$ = 7.5 Hz, $^4J_{H,H}$ = 1.6 Hz, 1 H, C_{ar}H), 6.58 (dd, $^3J_{H,H}$ = 8.8 Hz, $^4J_{H,H}$ = 2.6 Hz, 1 H, C_{ar}H), 6.39 ppm (d, $^4J_{H,H}$ = 2.5 Hz, 1 H, C_{ar}H).

^{13}C NMR (151 MHz, DMSO-d$_6$): δ = 163.8 (q, C_{ar}), 155.7 (q, C_{ar}), 149.8 (q, C_{ar}), 139.6 (t, C_{ar}H), 132.6 (t, C_{ar}H), 132.2 (q, C_{ar}), 131.8 (t, C_{ar}H), 129.4 (t, C_{ar}H), 117.3 (t, C_{ar}H), 109.7 (t, C_{ar}H), 102.9 (t, C_{ar}H), 100.4 ppm (q, C_{ar}).

IR (ATR): $\tilde{\nu}$ = 3659, 3361, 3064, 2980, 2888, 1621, 1589, 1516, 1505, 1472, 1454, 1438, 1397, 1372, 1314, 1291, 1229, 1184, 1160, 1153, 1107, 1035, 1015, 975, 951, 917, 896, 859, 843, 816, 795, 758, 701, 650, 618, 606 cm^{-1}.

UV/Vis (CH_2Cl_2): λ_{max} (log ε) = 394 nm (3.05).

HRMS (ESI): [$C_{12}H_9N_2O_2I$+H $^+$]: calculated: 340.9782; observed: 340.9785.

Synthesis of 6e:

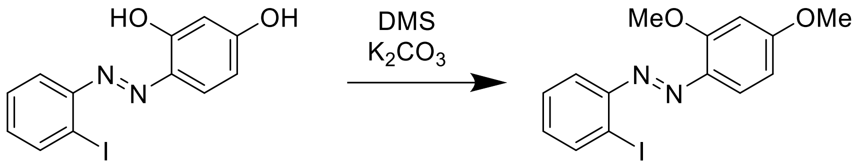

11 **6e**

Diol **11** (1020 mg, 3.0 mmol, 1 eq) and potassium carbonate (1500 mg, 10.9 mmol, 11 eq) were suspended in 20 mL acetonitrile. After addition of dimethyl sulfate (DMS, 0.6 mL, 6 mmol, 2 eq) the reaction mixture was heated to 100 °C and stirred overnight. The mixture was quenched with water and extracted with diethyl ether. The organic phase was dried over magnesium sulfate and dried in vacuo. The crude product was purified via column chromatography.

Yield: 940 mg, 2.6 mmol, 85%

R_f (*n*-Hexane/DCM 2:1) = 0.2

M.p.: 124 °C

^{1}H NMR (400 MHz, $CDCl_3$): δ = 7.97 (dd, $^3J_{H,H}$ = 7.9 Hz, $^4J_{H,H}$ = 1.3 Hz, 1 H, $C_{ar}H$), 7.88 (d, $^3J_{H,H}$ = 9.4 Hz, 1 H, $C_{ar}H$), 7.59 (dd, $^3J_{H,H}$ = 8.0 Hz, $^4J_{H,H}$ = 1.6 Hz, 1 H, $C_{ar}H$), 7.39 (dt, $^3J_{H,H}$ = 7.8 Hz, $^4J_{H,H}$ = 1.3 Hz, 1 H, $C_{ar}H$), 7.09 (dt, $^3J_{H,H}$ = 7.6 Hz, $^4J_{H,H}$ = 1.6 Hz, 1 H, $C_{ar}H$), 6.60–6.57 (m, 2 H, CH_3), 4.02 (s, 3 H, OCH_3), 3.90 ppm (s, 3 H, OCH_3).

^{13}C NMR (151 MHz, $CDCl_3$): δ = 164.4 (q, C_{ar}), 159.4 (q, C_{ar}), 152.2 (q, C_{ar}), 139.7 (t, $C_{ar}H$), 136.8 (q, C_{ar}), 131.3 (t, $C_{ar}H$), 129.1 (t, $C_{ar}H$), 119.2 (t, $C_{ar}H$), 117.9 (t, $C_{ar}H$), 106.1 (t, $C_{ar}H$), 101.7 (q, C_{ar}), 99.1 (t, $C_{ar}H$), 56.5 (p, OCH_3), 55.8 ppm (p, OCH_3).

IR (ATR): $\tilde{\nu}$ = 3006, 2980, 2941, 2922, 2837, 1597, 1575, 1563, 1494, 1474, 1463, 1455, 1422, 1408, 1316, 1289, 1246, 1208, 1178, 1157, 1118, 1099, 1036, 1026, 1012, 956, 928, 726, 703, 664, 631 cm^{-1}.

UV/Vis (CH_2Cl_2): λ_{max} (log ε) = 382 nm (3.22).

HRMS (ESI): [$C_{14}H_{13}N_2O_2I+H^+$]: calculated: 390.9914; observed: 390.9910.

Synthesis of 6f:

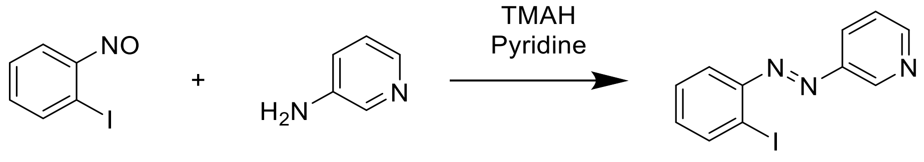

12 **13** **6f**

3-Aminopyridine (**13**, 333 mg, 3.4 mmol, 1 eq) and 3 mL aqueous tetramethylammonium hydroxide (TMAH) solution (60wt%) were dissolved in 5 mL pyridine and heated to 80 °C. 1-Iodo-2-nitrosobenzene (**12**, 800 mg, 3.4 mmol, 1 eq) was dissolved in pyridine and added dropwise to the reaction solution over 1 hour. The reaction mixture was stirred an additional 2 hours, cooled to room temperature, and extracted with toluene. The organic phase was dried over magnesium sulfate and dried in vacuo. The crude product was purified via column chromatography.

Yield: 220 mg, 0.71 mmol, 21%

R_f (*n*-hexane/ethyl acetate 2:1) = 0.4

M.p.: 62 °C

^{1}H NMR (400 MHz, $CDCl_3$): δ = 9.27 (d, $^4J_{H,H}$ = 2.2 Hz, 1 H, $C_{ar}H$), 8.74 (dd, $^3J_{H,H}$ = 4.8 Hz, $^4J_{H,H}$ = 1.6 Hz, 1 H, $C_{ar}H$), 8.25−8.21 (m, 1 H, $C_{ar}H$), 8.05 (dd, $^3J_{H,H}$ = 7.9 Hz, $^4J_{H,H}$ = 1.3 Hz, 1 H, $C_{ar}H$), 6.70 (dd, $^3J_{H,H}$ = 8.0 Hz, $^4J_{H,H}$ = 1.6 Hz, 1 H, $C_{ar}H$), 7.50−7.42 (m, 2 H, $C_{ar}H$), 7.21 ppm (dt, $^3J_{H,H}$ = 7.6 Hz, $^4J_{H,H}$ = 1.6 Hz, 1 H, $C_{ar}H$).

^{13}C NMR (151 MHz, $CDCl_3$): δ = 152.4 (t, $C_{ar}H$), 151.2 (q, C_{ar}), 148.4 (t, $C_{ar}H$), 147.7 (q, C_{ar}), 140.2 (t, $C_{ar}H$), 133.1 (t, $C_{ar}H$), 129.1 (t, $C_{ar}H$), 127.4 (t, $C_{ar}H$), 124.3 (t, $C_{ar}H$), 117.4 (t, $C_{ar}H$), 103.3 ppm (q, C_{ar}).

IR (ATR): $\tilde{\nu}$ = 3331, 3059, 2922, 2359, 1587, 1501, 1447, 1395, 1225, 1148, 1111, 1016, 997, 837, 733, 692 cm^{-1}.

UV/Vis (CH_3CN): λ_{max} (log ε) = 323 nm (2.97).

HRMS (ESI): [$C_{11}H_8N_3I$+H^+]: calculated: 309.9836; observed: 309.9841.

Synthesis of 1a:

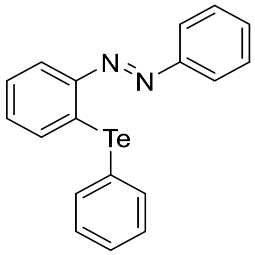

1a

Azobenzene **1a** was synthesized according to general procedure (*B*).

Yield: 28 mg, 0.07 mmol, 48%

R_f (*n*-pentane/DCM 9:1) = 0.4

M.p.: 50–52 °C

^{1}H NMR (600 MHz, $CDCl_3$): δ = 8.24 ($^3J_{H,H}$ = 7.8 Hz, $^4J_{H,H}$ = 1.2 Hz, 1 H, C_{ar}H), 8.07–8.05 (m, 2 H, C_{ar}H), 7.93–7.92 (m, 2 H, C_{ar}H), 7.55–7.51 (m, 3 H, C_{ar}H), 7.46–7.44 (m, 1 H, C_{ar}H), 7.42–7.39 (m, 1 H, C_{ar}H), 7.38–7.35 (m, 2 H, C_{ar}H), 7.23–7.21 (m, 1 H, C_{ar}H), 7.11–7.08 ppm (m, 1 H, C_{ar}H).

^{13}C NMR (151 MHz, $CDCl_3$): δ = 152.0 (q, C_{ar}), 151.1 (q, C_{ar}), 140.6 (t, C_{ar}), 134.0 (t, C_{ar}), 133.7 (t, C_{ar}), 131.2 (t, C_{ar}), 130.6 (t, C_{ar}), 129.7 (t, C_{ar}), 129.5 (t, C_{ar}), 128.8 (t, C_{ar}), 126.6 (t, C_{ar}), 122.6 (t, C_{ar}), 122.3 (q, C_{ar}), 116.9 ppm (q, C_{ar}).

^{125}Te NMR (189 MHz, $CDCl_3$), δ = 736 ppm.

IR (ATR): $\tilde{\nu}$ = 3051, 1582, 1433, 1298, 1180, 1016, 768, 687 cm^{-1}.

UV/Vis ($CHCl_3$): λ_{max} (log ε) = 331 (4.16), 447 nm (3.64).

HRMS (ESI): [$C_{18}H_{14}N_2{}^{130}Te+H^+$]: calculated: 389.0292; observed: 389.0287.

Synthesis of 1b:

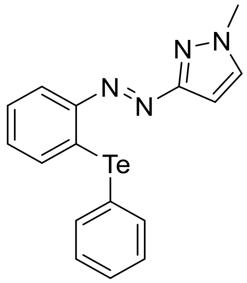

1b

Azoarene **1b** was synthesized according to general procedure (*B*).

Yield: 10 mg, 0.03 mmol, 6%

R_f (*n*-hexane/DCM 1:1) = 0.5

^{1}H NMR (400 MHz, $CDCl_3$): δ = 8.19 (dd, $^3J_{H,H}$ = 7.9 Hz, $^4J_{H,H}$ = 1.1 Hz, 1 H, C_{ar}H), 7.9 (d, $^3J_{H,H}$ = 7.6 Hz, 2 H, C_{ar}H), 7.49–7.40 (m, 2 H, C_{ar}H), 7.40–7.31 (m, 3 H, C_{ar}H), 7.21 (dd, $^3J_{H,H}$ = 8.4 Hz, $^4J_{H,H}$ = 1.1 Hz, 1 H, C_{ar}H), 7.07 (dt, $^3J_{H,H}$ = 7.6 Hz, $^4J_{H,H}$ = 1.4 Hz, 1 H, C_{ar}H), 6.74 (d, $^3J_{H,H}$ = 2.6 Hz, 1 H, C_{ar}H), 4.05 ppm (s, 3 H, N-CH_3).

^{13}C NMR (151 MHz, $CDCl_3$): δ = 161.6 (q, C_{ar}), 152.0 (q, C_{ar}), 140.6 (t, C_{ar}H), 133.9 (t, C_{ar}H), 132,8 (t, C_{ar}H), 132.2 (t, C_{ar}H), 130.6 (t, C_{ar}H), 129.6 (t, C_{ar}H), 128.7 (t, C_{ar}H), 126.4 (t, C_{ar}H), 122.4 (q, C_{ar}), 117.5 (q, C_{ar}), 97.2 (t, C_{ar}H), 40.0 ppm (s, 3 H, N-CH_3).

^{125}Te NMR (189 MHz, $CDCl_3$), δ = 738 ppm.

IR (ATR): $\tilde{\nu}$ = 3057, 2922, 2853, 2359, 1589, 1508, 1470, 1431, 1406, 1366, 1302, 1260, 1213, 1155, 1111, 1050, 1016, 997, 912, 864, 764, 733, 718, 694, 642, 617 cm^{-1}.

UV/Vis ($CHCl_3$): λ_{max} (log ε) = 336 (2.68), 454 nm (2.08).

HRMS (ESI): [$C_{16}H_{14}N_4{}^{130}Te+H^+$]: calculated: 393.0354; observed: 393.0354.

Synthesis of 1c:

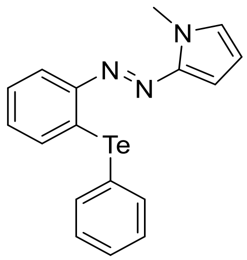

1c

Azoarene **1c** was synthesized according to general procedure (*B*).

Yield: 95 mg, 0.24 mmol, 52%

R_f (*n*-hexane/DCM 2:1) = 0.2

M.p.: 109–110 °C

^{1}H NMR (400 MHz, $CDCl_3$): δ = 7.98–7.88 (m, 3 H, C_{ar}H), 7.45 (dt, $^3J_{H,H}$ = 7.4 Hz, $^4J_{H,H}$ = 1.3 Hz, 1 H, C_{ar}H), 7.35 (t, $^3J_{H,H}$ = 7.6 Hz, 2 H, C_{ar}H), 7.29 (dt, $^3J_{H,H}$ = 7.4 Hz, $^4J_{H,H}$ = 1.3 Hz, 1 H, C_{ar}H), 7.13 (dd, $^3J_{H,H}$ = 7.9 Hz, $^4J_{H,H}$ = 1.2 Hz, 1 H, C_{ar}H), 6.99 (dt, $^3J_{H,H}$ = 7.7 Hz, $^4J_{H,H}$ = 1.5 Hz 1 H, C_{ar}H) 6.93 (t, $^3J_{H,H}$ = 2.1 Hz, 1 H, C_{ar}H), 6.83 (dd, $^3J_{H,H}$ = 4.2 Hz, $^4J_{H,H}$ = 1.7 Hz, 1 H, C_{ar}H), 6.29 (dd, $^3J_{H,H}$ = 4.3 Hz, $^4J_{H,H}$ = 2.6 Hz, 1 H, C_{ar}H), 4.07 ppm (s, 3 H, N-CH_3).

^{13}C NMR (151 MHz, $CDCl_3$): δ = 152.3 (q, C_{ar}), 146.3 (q, C_{ar}), 141.1 (t, C_{ar}H), 133.6 (t, C_{ar}H), 129.8 (t, C_{ar}H), 129.5 (t, C_{ar}H), 128.9 (t, C_{ar}H), 128.1 (t, C_{ar}H), 127.7 (t, C_{ar}H), 126.7 (t, C_{ar}H), 119.0 (q, C_{ar}), 115.9 (q, C_{ar}), 110.4 (t, C_{ar}H), 103.1 (t, C_{ar}H), 35.8 ppm (p, N-CH_3).

^{125}Te NMR (189 MHz, $CDCl_3$), δ = 686 ppm.

IR (ATR): $\tilde{\nu}$ = 3119, 3055, 2361, 1757, 1574, 1541, 1510, 1491, 1449, 1433, 1400, 1344, 1321, 1246, 1209, 1167, 1049, 1016, 997, 976, 880, 851, 806, 793, 760, 730, 714, 694, 650 cm^{-1}.

UV/Vis ($CHCl_3$): λ_{max} (log ε) = 384 nm (3.26).

HRMS (ESI): [$C_{17}H_{15}N3^{130}Te+H^{+}$]: calculated: 392.0402; observed: 392.0402.

Synthesis of 1d:

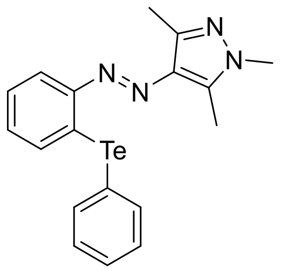

1d

Azoarene **1d** was synthesized according to general procedure (*B*).

Yield: 106 mg, 0.25 mmol, 16%

R_f (*n*-hexane/DCM 1:1) = 0.7

^{1}H NMR (400 MHz, $CDCl_3$): δ = 7.99 (dd, $^3J_{H,H}$ = 8.0 Hz, $^4J_{H,H}$ = 1.2 Hz, 1 H, C_{ar}H), 7.93 (d, $^3J_{H,H}$ = 7.1 Hz, 2 H, C_{ar}H), 7.44 (t, $^3J_{H,H}$ = 7.6 Hz, 1 H, C_{ar}H), 7.38−7.29 (m, 3 H, C_{ar}H), 7.16 (dd, $^3J_{H,H}$ = 8.2 Hz, $^4J_{H,H}$ = 0.8 Hz, 1 H, C_{ar}H), 7.02 (dt, $^3J_{H,H}$ = 7.7 Hz, $^4J_{H,H}$ = 1.3 Hz, 1 H, C_{ar}H), 3.81 (s, 3 H, N-CH_3), 2.69 (s, 3 H, CH_3), 2.58 ppm (s, 3 H, CH_3).

^{13}C NMR (151 MHz, $CDCl_3$): δ = 152.2 (q, C_{ar}), 143.0 (q, C_{ar}), 140.9 (t, C_{ar}H), 138.0 (q, C_{ar}), 135.0 (q, C_{ar}), 133.6 (t, C_{ar}H), 129.7 (t, C_{ar}H), 129.6 (t, C_{ar}H), 129.5 (t, C_{ar}H), 128.8 (t, C_{ar}H), 126.5 (t, C_{ar}H), 120.1 (q, C_{ar}), 114.5 (q, C10), 36.4 (p, N-CH_3), 14.9 (p, CH_3), 11.5 ppm (p, CH_3).

^{125}Te NMR (189 MHz, $CDCl_3$), δ = 700 ppm.

IR (ATR): $\tilde{\nu}$ = 2945, 2913, 2359, 1699, 1549, 1506, 1427, 1391, 1366, 1300, 1256, 1223, 1123, 1065, 1018, 995, 910, 862, 849, 787, 764, 735, 719, 692, 664 cm^{-1}.

UV/Vis ($CHCl_3$): λ_{max} (log ε) = 339 (3.14), 413 nm (2.64).

HRMS (ESI): [$C_{18}H_{18}N_4{}^{130}Te+H^+$]: calculated: 421.0667; observed: 421.0069.

Synthesis of 1e:

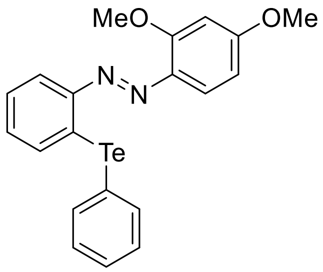

1e

Azobenzene **1e** was synthesized according to general procedure (*B*).

Yield: 15 mg, 0.03 mmol, 21%

R_f (*n*-hexane/ethyl acetate 4:1) = 0.4

M.p.: 145 °C

^{1}H NMR (400 MHz, $CDCl_3$): δ = 8.17 (dd, $^3J_{H,H}$ = 7.8 Hz, $^4J_{H,H}$ = 1.4 Hz, 1 H, C_{ar}H), 7.98 (d, $^3J_{H,H}$ = 9.0 Hz, 1 H, C_{ar}H), 7.95 (dd, $^3J_{H,H}$ = 8.1 Hz, $^4J_{H,H}$ = 1.3 Hz, 2 H, C_{ar}H), 7.44 (dt, $^3J_{H,H}$ = 7.5 Hz, $^4J_{H,H}$ = 2.3 Hz, 1 H, C_{ar}H), 7.40−7.32 (m, 3 H, C_{ar}H), 7.22 (dd, $^3J_{H,H}$ = 8.0 Hz, $^4J_{H,H}$ = 1.2 Hz, 1 H, C_{ar}H), 7.03 (dt, $^3J_{H,H}$ = 7.6 Hz, $^4J_{H,H}$ = 1.5 Hz, 1 H, C_{ar}H), 6.65 (d, $^4J_{H,H}$ = 2.1 Hz, 1 H, C_{ar}H), 7.03 (dt, $^3J_{H,H}$ = 8.9 Hz, $^4J_{H,H}$ = 2.6 Hz, 1 H, C_{ar}H), 4.09 (s, 3 H, OCH_3), 3.91 ppm (s, 3 H, OCH_3).

^{13}C NMR (151 MHz, $CDCl_3$): δ = 163.7 (q, C_{ar}), 158.6 (q, C_{ar}), 151.8 (q, C_{ar}), 140.8 (t, C_{ar}H), 135.4 (q, C_{ar}), 134.0 (t, C_{ar}H), 133.3 (t, C_{ar}H), 129.6 (t, C_{ar}H), 129.6 (t, C_{ar}H), 128.5 (t, C_{ar}H), 126.2 (t, C_{ar}H), 123.5 (q, C_{ar}), 118.9 (t, C_{ar}H), 115.6 (q, C_{ar}), 106.0 (t, C_{ar}H), 98.9 (t, C_{ar}H), 55.8 (p, OCH_3), 55.8 ppm (p, OCH_3).

^{125}Te NMR (189 MHz, $CDCl_3$), δ = 763 ppm.

IR (ATR): $\tilde{\nu}$ = 3052, 2940, 2838, 1605, 1597, 1577, 1492, 1469, 1453, 1438, 1427, 1392, 1317, 1292, 1260, 1247, 1230, 1208, 1180, 1163, 1154, 1116, 1035, 1026, 999, 831, 797, 770, 733, 120, 708, 693, 637 cm^{-1}.

UV/Vis (CH_2Cl_2): λ_{max} (log ε) = 375 (3.26), 443 nm (2.97).

HRMS (ESI): [$C_{20}H_{18}N_2O_2{}^{130}Te+H^+$]: calculated: 449.0503; observed: 449.0503.

Synthesis of 1f:

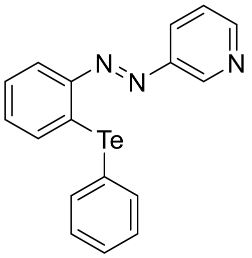

1f

Azoarene **1f** was synthesized according to general procedure (*B*).

Yield: 23 mg, 0.06 mmol, 15%

R_f (DCM) = 0.1

M.p.: 115 °C

^{1}H NMR (400 MHz, $CDCl_3$): δ = 9.34 (d, $^4J_{H,H}$ = 2.4 Hz, 1 H, C_{ar}H), 8.73 (dd, $^3J_{H,H}$ = 4.8 Hz, $^4J_{H,H}$ = 1.6 Hz, 1 H, C_{ar}H), 8.29−8.24 (m, 2 H, C_{ar}H), 7.92 (d, $^3J_{H,H}$ = 8.2 Hz, 2 H, C_{ar}H), 7.49−7.34 (m, 5 H, C_{ar}H), 7.24 (dd, $^3J_{H,H}$ = 8-0 Hz, $^4J_{H,H}$ = 1.2 Hz, 1 H, C_{ar}H), 7.12 ppm (dt, $^3J_{H,H}$ = 7.5 Hz, $^4J_{H,H}$ = 1.6 Hz, 1 H, C_{ar}H).

^{13}C NMR (151 MHz, $CDCl_3$): δ = 152.1 (q, C_{ar}), 151.8 (t, C_{ar}H), 146.8 (q, C_{ar}), 146.3 (t, C_{ar}H), 140.6 (t, C_{ar}H), 134.2 (t, C_{ar}H), 134.1 (t, C_{ar}H), 131.2 (t, C_{ar}H), 129.8 (t, C_{ar}H), 129.0 (t, C_{ar}H), 127.2 (t, C_{ar}H), 126.7 (t, C_{ar}H), 124.4 (t, C_{ar}H), 121.7 (q, C_{ar}), 117.7 ppm (q, C_{ar}).

^{125}Te NMR (189 MHz, $CDCl_3$), δ = 745 ppm.

IR (ATR): $\tilde{\nu}$ = 3048, 2981, 2972, 2921, 2892, 2852, 1809, 1713, 1629, 1571, 1548, 1470, 1461, 1420, 1394, 1321, 1305, 1256, 1227, 1218, 1194, 1173, 1155, 1112, 1091, 1061, 1015, 996, 979, 962, 949, 930, 906, 861, 850, 833, 808, 763, 732, 710, 693, 640, 615 cm^{-1}.

UV/Vis (CH_3Cl_3): λ_{max} (log ε) = 340 (3.12), 459 nm (2.43).

HRMS (ESI): [$C_{17}H_{13}N_3{}^{130}Te+H^+$]: calculated: 390.0245; observed: 390.0245.

Synthesis of 5:

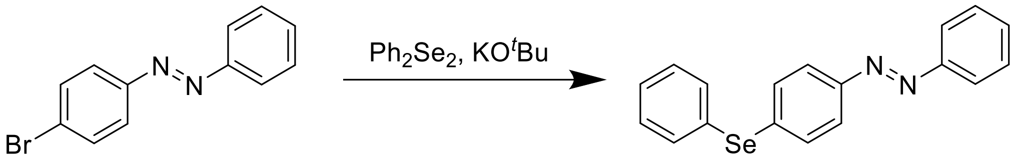

9 5

Azobenzene **9** (392 mg, 1.5 mmol, 1.0 eq) and diphenyl diselenide (250 mg, 0.8 mmol, 0.5 eq) were dissolved in 1.5 mL DMSO and stirred at 45 °C. Potassium *tert*-butanolate (210 mg, 1.9 mmol, 1.3 eq) was added in several portions. Afterwards the reaction solution was stirred at 45 °C for 15 minutes and then over night at 80 °C. After cooling to room temperature, the reaction mixture was diluted with water and extracted with dichloromethane. The organic phase was dried over magnesium sulfate and dried in vacuo. The crude product was purified by flash column chromatography with silica gel to provide the desired azobenzene as orange solid.

Yield: 240 mg, 0.71 mmol, 47%

R_f (*n*-hexane/DCM 9:1) = 0.3

M.p.: 80 °C

^{1}H NMR (400 MHz, $CDCl_3$): δ = 7.89 (dd, $^3J_{H,H}$ = 8.4 Hz, $^4J_{H,H}$ = 1.6 Hz, 2 H, C_{ar}H), 7.80 (d, $^3J_{H,H}$ = 8.6 Hz, 2 H, C_{ar}H), 7.61 7.55 (m, 2 H, C_{ar}H), 7.54–7.43 (m, 5 H, C_{ar}H), 7.37–7.43 ppm (m, 3 H, C_{ar}H).

^{13}C NMR (151 MHz, $CDCl_3$): δ = 152.6 (q, C_{ar}), 151.4 (q, C_{ar}), 136.4 (q, C_{ar}), 134.3 (t, C_{ar}H), 132.0 (t, C_{ar}H), 131.0 (t, C_{ar}H), 129.7 (q, C_{ar}), 129.6 (t, C_{ar}H), 129.1 (t, C_{ar}H), 128.1 (t, C_{ar}H), 123.5 (t, C_{ar}H), 122.8 ppm (t, C_{ar}H).

IR (ATR): $\tilde{\nu}$ = 3049, 2359, 2322, 1574, 1566, 1476, 1464, 1437, 1396, 1300, 1225, 1153, 1101, 1070, 1061, 1018, 1007, 997, 930, 833, 770, 743, 737, 710, 685, 667 cm^{-1}.

UV/Vis (CH_2Cl_2): λ_{max} (log ε) = 299 (2.92), 365 nm (3.12).

HRMS (ESI): [$C_{18}H_{14}N_2{}^{80}Se+H^+$]: calculated: 339.0396; observed: 339.0394.

Synthesis of 2a:

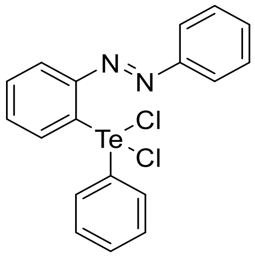

2a

Azobenzene **2a** was synthesized according to general procedure (*C*).

Yield: 45 mg, 0.1 mmol, quant.

M.p.: 147–149 °C

^{1}H NMR (400 MHz, $CDCl_3$): δ = 8.45–8.42 (m, 2 H, C_{ar}H), 8.20–8.17 (m, 1 H, C_{ar}H), 8.08–8.06 (m, 2 H, C_{ar}H), 7.75–7.71 (m, 1 H, C_{ar}H), 7.67–7.64 (m, 4 H, C_{ar}H), 7.62–7.60 (m, 3 H, C_{ar}H), 7.57–7.53 ppm (m, 1 H, C_{ar}H).

^{13}C NMR (151 MHz, $CDCl_3$): δ = 151.3 (q, C_{ar}), 148.4 (q, C_{ar}), 135.9 (t, C_{ar}H), 133.4 (t, C_{ar}H), 133.0 (t, C_{ar}H), 132.6 (t, C_{ar}H), 132.0 (t, C_{ar}H), 131.8 (t, C_{ar}H), 130.1 (t, C_{ar}H), 129.7 (q, C_{ar}H), 129.2 (t, C_{ar}H), 124.6 (t, C_{ar}H), 123.6 (t, C_{ar}H), 123.0 ppm (t, C_{ar}H).

^{125}Te NMR (189 MHz, $CDCl_3$), δ = 910 ppm.

IR (ATR): $\tilde{\nu}$ = 3059, 1574, 1476, 1227, 1150, 997, 772, 683 cm^{-1}.

UV/Vis ($CHCl_3$): λ_{max} (log ε) = 336 nm (4.08).

HRMS (ESI): [$C_{18}H_{14}N_2Cl_2{}^{130}Te$-2Cl+OH $^+$]: calculated: 405.0242; observed: 405.0243.

Synthesis of 2b:

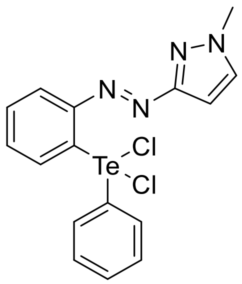

2b

Azoarene **2b** was synthesized according to general procedure (*C*).

Yield: 46 mg, 0.1 mmol, quant.

M.p.: 193 °C

^{1}H NMR (400 MHz, $CDCl_3$): δ = 8.45–8.37 (m, 2 H, $C_{ar}H$), 8.16 (dd, $^{3}J_{H,H}$ = 8.0 Hz, $^{4}J_{H,H}$ = 1.3 Hz, 1 H, $C_{ar}H$), 7.69 (dt, $^{3}J_{H,H}$ = 7.3 Hz, $^{4}J_{H,H}$ = 1.3 Hz, 1 H, $C_{ar}H$), 7.66–7.59 (m, 4 H, $C_{ar}H$), 7.52 (dt, $^{3}J_{H,H}$ = 7.7 Hz, $^{4}J_{H,H}$ = 1.5 Hz, 1 H, $C_{ar}H$), 7.5 (d, $^{3}J_{H,H}$ = 2.4 Hz, 1 H, $C_{ar}H$), 6.77 (d, $^{3}J_{H,H}$ = 2.5 Hz, 1 H, $C_{ar}H$), 4.08 ppm (s, 3 H, N-CH_3).

^{13}C NMR (151 MHz, $CDCl_3$): δ = 162.4 (q, C_{ar}), 148.7 (q, C_{ar}), 138.7 (q, C_{ar}), 135.8 (t, $C_{ar}H$), 133.3 (t, $C_{ar}H$), 132.7 (t, $C_{ar}H$), 132.6 (t, $C_{ar}H$), 132.2 (q, C_{ar}), 131.7 (t, $C_{ar}H$), 131.7 (t, $C_{ar}H$), 130.1 (t, $C_{ar}H$), 123.1 (t, $C_{ar}H$), 96.9 (t, $C_{ar}H$), 40.2 ppm (p, CH_3).

^{125}Te NMR (189 MHz, $CDCl_3$), δ = 908 ppm.

IR (ATR): $\tilde{\nu}$ = 3139, 3096, 3055, 2980, 2902, 2889, 1571, 1542, 1510, 1473, 1434, 1423, 1390, 1363, 1302, 1256, 1223, 1198, 1158, 1113, 1074, 1054, 1010, 995, 967, 954, 922, 903, 892, 864, 818, 778, 764, 733, 726, 721, 683, 652 cm^{-1}.

UV/Vis (CH_2Cl_2): λ_{max} (log ε) = 345 nm (3.36).

HRMS (ESI): [$C_{16}H_{14}N_4Cl_2{}^{130}Te$-2Cl+OH $^{+}$]: calculated:409.0303; observed: 409.0304.

Synthesis of 2c:

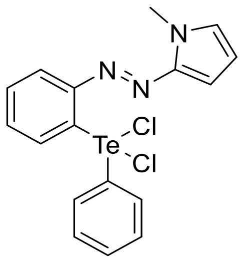

2c

Azoarene **2c** was synthesized according to general procedure (*C*).

Yield: 46 mg, 0.1 mmol, quant

M.p.: degradation >170 °C

^{1}H NMR (400 MHz, $CDCl_3$): δ = 8.47–8.40 (m, 2 H, $C_{ar}H$), 7.94 (dd, $^{3}J_{H,H}$ = 8.1 Hz, $^{4}J_{H,H}$ = 1.0 Hz, 1 H, $C_{ar}H$), 7.67−7.56 (m, 5 H, $C_{ar}H$), 7.38 (dt, $^{3}J_{H,H}$ = 7.7 Hz, $^{4}J_{H,H}$ = 1.4 Hz, 1 H, $C_{ar}H$), 7.09 (t, $^{3}J_{H,H}$ = 1.9 Hz, 1 H, $C_{ar}H$), 6.95 (dd, $^{3}J_{H,H}$ = 4.4 Hz, $^{4}J_{H,H}$ = 1.6 Hz, 1 H, $C_{ar}H$), 6.40 (dd, $^{3}J_{H,H}$ = 4.3 Hz, $^{3}J_{H,H}$ = 2.5 Hz, 1 H, $C_{ar}H$), 4.03 ppm (s, 3 H, N-CH_3).

^{13}C NMR (151 MHz, $CDCl_3$): δ = 150.1 (q, C_{ar}), 146.4 (q, C_{ar}), 141.8 (q, C_{ar}), 135.9 (t, $C_{ar}H$), 132.6 (t, $C_{ar}H$), 131.7 (t, $C_{ar}H$), 131.0 (t, $C_{ar}H$), 130.8 (t, $C_{ar}H$), 130.4 (t, CarH), 130.1 (t, $C_{ar}H$), 118.2 (t, $C_{ar}H$), 112.1 (t, $C_{ar}H$), 105.1 (t, $C_{ar}H$), 34.2 ppm (p, CH_3).

^{125}Te NMR (189 MHz, $CDCl_3$), δ = 893 ppm.

IR (ATR): $\tilde{\nu}$ = 3047, 2919, 1571, 1560, 1512, 1492, 1473, 1467, 1447, 1434, 1362, 1328, 1255, 1238, 1211, 1184, 1160, 1107, 995, 860, 738, 714, 683, 651 cm^{-1}.

UV/Vis (CH_2Cl_2): λ_{max} (log ε) = 409 nm (3.27).

HRMS (ESI): [$C_{17}H_{15}N_3Cl_2{}^{130}Te$-2Cl+OH $^{+}$]: calculated: 408.0351; observed: 408.0351.

Synthesis of 2d:

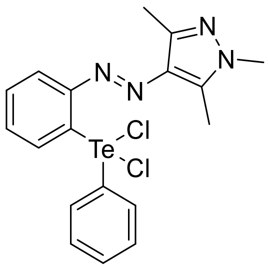

2d

Azoarene **2d** was synthesized according to general procedure (*C*).

Yield: 49 mg, 0.1 mmol, quant

M.p.: 218 °C

^{1}H NMR (400 MHz, $CDCl_3$): δ = 8.47−8.40 (m, 2 H, $C_{ar}H$), 7.94 (dd, $^{3}J_{H,H}$ = 8.0 Hz, $^{4}J_{H,H}$ = 1.2 Hz, 1 H, $C_{ar}H$),7.67−7.60 (m, 4 H, $C_{ar}H$), 7.57 (dd, $^{3}J_{H,H}$ = 8.0 Hz, $^{4}J_{H,H}$ = 1.2 Hz, 1 H, $C_{ar}H$), 7.41 (dt, $^{3}J_{H,H}$ = 7.6 Hz, $^{4}J_{H,H}$ = 1.3 Hz, 1 H, $C_{ar}H$), 3.84 (s, 3 H, $C_{ar}H$), 2.69 (s, 3 H, N-CH_3), 2.67 ppm (s, 3 H, N-CH_3).

^{13}C NMR (151 MHz, $CDCl_3$): δ = 149.9 (q, C_{ar}), 143.1 (q, C_{ar}), 141.3 (q, C_{ar}), 138.5 (q, C_{ar}), 135.9 (t, $C_{ar}H$), 135.3 (q, C_{ar}), 132.5 (t, $C_{ar}H$), 131.8 (t, $C_{ar}H$), 131.4 (t, $C_{ar}H$), 130.9 (t, $C_{ar}H$), 130.7 (q, C_{ar}), 130.1 (t, $C_{ar}H$), 120.8 (t, $C_{ar}H$), 36.4 (p, CH_3), 14.7 (p, CH_3), 10.7 ppm (p, CH_3).

^{125}Te NMR (189 MHz, $CDCl_3$), δ = 908 ppm.

IR (ATR): $\tilde{\nu}$ = 3087, 3074, 3054, 2980, 2931, 2889, 1586, 1545, 1417, 1474, 1444, 1435, 1405, 1394, 1366, 1305, 1291, 1260, 1233, 1201, 1180, 1153, 1111, 1070, 1052, 1044, 1019, 996, 942, 893, 866, 790, 764, 740, 720, 687, 669, 646 cm^{-1}.

UV/Vis (CH_2Cl_2): λ_{max} (log ε) = 354 nm (3.53).

HRMS (ESI): [$C_{18}H_{18}N_4Cl_2{}^{130}Te$-2Cl+OH $^{+}$]: calculated: 437.0616; observed: 437.0617.

Synthesis of 2e:

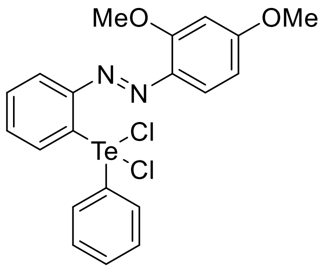

2e

Azobenzene **2e** was synthesized according to general procedure (*C*).

Yield: 51 mg, 0.1 mmol, quant

M.p.: degradation >190 °C

^{1}H NMR (400 MHz, $CDCl_3$): δ = 8.45–8.39 (m, 2 H, $C_{ar}H$), 8.16 (dd, $^{3}J_{H,H}$ = 7.9 Hz, $^{4}J_{H,H}$ = 1.3 Hz, 1 H, $C_{ar}H$), 7.97 (d, $^{3}J_{H,H}$ = 9.7 Hz, 1 H, $C_{ar}H$), 7.68 (dt, $^{3}J_{H,H}$ = 7.5 Hz, $^{4}J_{H,H}$ = 1.1 Hz, 1 H, $C_{ar}H$), 7.63–7.56 (m, 4 H, $C_{ar}H$), 7.43 (dt, $^{3}J_{H,H}$ = 7.4 Hz, $^{4}J_{H,H}$ = 1.3 Hz, 1 H, $C_{ar}H$), 6.66–6.61 (m, $C_{ar}H$), 4.08 (s, 3 H, OCH_3), 3.93 ppm (s, 3 H, OCH_3).

^{13}C NMR (151 MHz, $CDCl_3$): δ = 165.7 (q, C_{ar}), 160.1 (q, C_{ar}), 148.5 (q, C_{ar}), 136.1 (t, $C_{ar}H$), 134.9 (q, C_{ar}), 134.6 (q, C_{ar}), 132.6 (t, $C_{ar}H$), 132.3 (t, $C_{ar}H$), 132.1 (t, $C_{ar}H$), 131.3 (t, $C_{ar}H$), 129.8 (t, $C_{ar}H$), 129.7 (q, C_{ar}), 129.6 (t, $C_{ar}H$), 119.6 (t, $C_{ar}H$), 106.9 (t, $C_{ar}H$), 98.9 (t, $C_{ar}H$), 56.2 (p, OCH_3), 56.0 ppm (p, OCH_3).

^{125}Te NMR (189 MHz, $CDCl_3$), δ = 975 ppm.

IR (ATR): $\tilde{\nu}$ = 3074, 3054, 3002, 2970, 2938, 2832, 1607, 1598, 1574, 1556, 1536, 1486, 1475, 1464, 1452, 1435, 1427, 1417, 1411, 1322, 1291, 1264, 1253, 1210, 1182, 1158, 1119, 1055, 1030, 1020, 952, 996, 953, 932, 874, 850, 817, 796, 757, 751, 732, 708, 687, 608 cm^{-1}.

UV/Vis (CH_2Cl_2): λ_{max} (log ε) = 404 nm (3.05).

HRMS (ESI): [$C_{20}H_{18}N_2O_2Cl_2{}^{130}Te$-2Cl+OH $^{+}$]: calculated: 465.0454; observed: 465.0454.

Synthesis of 2f:

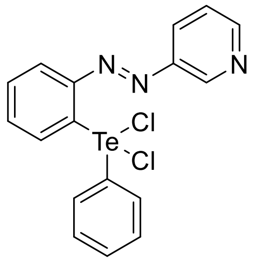

2f

Azoarene **2f** was synthesized according to general procedure (*C*).

Yield: 45 mg, 0.1 mmol, 99%

M.p.: 202 °C

^{1}H NMR (400 MHz, $CDCl_3$): δ = 9.35 (s, 1 H, C_{ar}H), 8.83 (d, $^{3}J_{H,H}$ = 3.8 Hz, 1 H, C_{ar}H), 8.43 (m, 2 H, C_{ar}H), 8.31 (td, $^{3}J_{H,H}$ = 8.3 Hz, $^{4}J_{H,H}$ = 1.7 Hz, 1 H, C_{ar}H), 8.17 (dd, $^{3}J_{H,H}$ = 8.0 Hz, $^{4}J_{H,H}$ = 1.3 Hz, 1 H, C_{ar}H), 7.74 (dt, $^{3}J_{H,H}$ = 7.3 Hz, $^{4}J_{H,H}$ = 1.3 Hz, 1 H, C_{ar}H), 7.70–7.63 (m, 4 H, C_{ar}H), 7.62–7.55 ppm (m, 2 H, C_{ar}H).

^{13}C NMR (151 MHz, $CDCl_3$): δ = 152.3 (t, C_{ar}H), 148.4 (q, C_{ar}), 147.1 (q, C_{ar}H), 147.0 (t, C_{ar}), 140.1 (q, C_{ar}), 135.6 (t, C_{ar}H), 134.1 (t, C_{ar}H), 132.6 (t, C_{ar}H), 131.9 (t, C_{ar}H), 131.8 (t, C_{ar}H), 131.0 (q, C_{ar}), 130.1 (t, C_{ar}H), 128.1 (t, C_{arH}), 124.7 (t, C_{ar}H), 122.6 ppm (t, C_{ar}H).

^{125}Te NMR (189 MHz, $CDCl_3$): δ = 896 ppm.

IR (ATR): $\tilde{\nu}$ = 3055, 2920, 2851, 1589, 1574, 1474, 1435, 1420, 1358, 1300, 1233, 1188, 1175, 1113, 1055, 1024, 964, 930, 881, 816, 772, 729, 719, 694, 683, 623 cm^{-1}.

UV/Vis (CH_2Cl_2): λ_{max} (log ε) = 335 nm (3.31).

HRMS (ESI): [$C_{17}H_{13}N_3Cl_2{}^{130}Te$-2Cl+OH $^{+}$]: 406.0194; observed: 406.0191.

Synthesis of 3a:

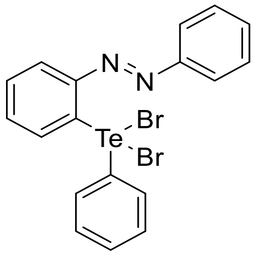

3a

Azobenzene **3a** was synthesized according to general procedure (*D*).

Yield: 45 mg, 0.08 mmol, 78%

M.p.: 157–159 °C

^{1}H NMR (600 MHz, $CDCl_3$): δ = 8.49 (d, $^{3}J_{H,H}$ = 7.2 Hz, 2 H, $C_{ar}H$), 8.21 (d, $^{3}J_{H,H}$ = 7.7 Hz, 1 H, $C_{ar}H$), 8.11–8.06 (m, 2 H, $C_{ar}H$), 7.75 (t, $^{3}J_{H,H}$ = 7.5 Hz, 1 H, $C_{ar}H$), 7.71 (d, $^{3}J_{H,H}$ = 7.6 Hz, 1 H, $C_{ar}H$), 7.65–7.51 ppm (m, 7 H, $C_{ar}H$).

^{13}C NMR (151 MHz, $CDCl_3$): δ = 151.1 (q, C_{ar}), 148.2 (q, C_{ar}), 137.4 (t, C_{ar}), 133.5 (t, C_{ar}), 133.48 (t, C_{ar}), 133.1 (t, C_{ar}), 132.7 (t, C_{ar}), 132.5 (q, C_{ar}), 131.8 (t, C_{ar}), 130.2 (t, C_{ar}), 129.8 (t, C_{ar}), 128.9 (q, C_{ar}), 126.4 (t, C_{ar}), 123.6 ppm (t, C_{ar}).

^{125}Te NMR (189 MHz, $CDCl_3$), δ = 884 ppm.

IR (ATR): $\tilde{\nu}$ = 2974, 1474, 1486, 1452, 1435, 1229, 1109, 1096, 1074, 995, 828, 768, 733, 710, 669, 615 cm^{-1}.

UV/Vis ($CHCl_3$): λ_{max} (log ε) = 333 (4.21), 465 nm (3.17).

HRMS (ESI): [$C_{18}H_{14}N_2Br_2{}^{130}Te$-Br $^{+}$] calculated: 466.9380; found: 466.9377.

Synthesis of 3b:

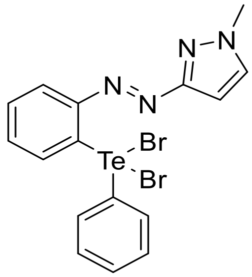

3b

Azoarene **3b** was synthesized according to general procedure (*D*).

Yield: 38 mg, 0.07 mmol, 71%

M.p.: 180 °C

^{1}H NMR (400 MHz, CDCl$_3$): δ = 8.47 (dd, $^3J_{H,H}$ = 8.0 Hz, $^4J_{H,H}$ = 1.5 Hz, 2 H, C$_{ar}$H), 8.16 (dd, $^3J_{H,H}$ = 8.3 Hz, $^4J_{H,H}$ = 1.5 Hz, 1 H, C$_{ar}$H), 7.73–7.67 (m, 2 H, C$_{ar}$H), 7.64–7.55 (m, 3 H, C$_{ar}$H, C$_{ar}$H), 7.52 (dt, $^3J_{H,H}$ = 8.0 Hz, $^4J_{H,H}$ = 1.3 Hz, 1 H, C$_{ar}$H), 7.48 (d, $^3J_{H,H}$ = 2.5 Hz, 1 H, C$_{ar}$H), 6.79 (d, $^3J_{H,H}$ = 2.6 Hz, 1 H, C$_{ar}$H), 4.09 ppm (s, 3 H, N-CH$_3$).

^{13}C NMR (151 MHz, CDCl$_3$): δ = 162.2 (q, C$_{ar}$), 148.5 (q, C$_{ar}$), 137.3 (t, C$_{ar}$H), 134.1 (q, C$_{ar}$), 133.4 (t, C$_{ar}$H), 132.6 (t, C$_{ar}$H), 132.6 (t, C$_{ar}$H), 132.4 (t, C$_{ar}$H), 131.7 (t, C$_{ar}$H), 130.1 (t, C$_{ar}$H), 129.1 (q, C$_{ar}$), 124.3 (t, C$_{ar}$H), 97.1 (t, C$_{ar}$H), 40.2 ppm (p, N-CH$_3$).

^{125}Te NMR (189 MHz, CDCl$_3$), δ = 879 ppm.

IR (ATR): $\tilde{\nu}$ = 3144, 3137, 3120, 3047, 2912, 1470, 1443, 1426, 1403, 1360, 1298, 1261, 1216, 1157, 1111, 1062, 1050, 1008, 996, 768, 759, 738, 717, 696, 687 cm^{-1}.

UV/Vis (CH$_2$Cl$_2$): λ_{max} (log ε) = 344 (3.25), 421 nm (2.00).

HRMS (ESI): [C$_{16}$H$_{14}$N$_4$Br$_2$^{130}Te-2Br+OH $^+$]: calculated:409.0303; observed: 409.0303.

Synthesis of 3c:

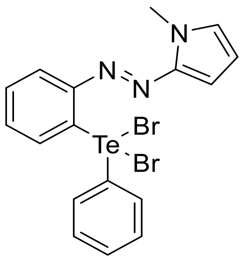

3c

Azoarene **3c** was synthesized according to general procedure (*D*).

Yield: 42 mg, 0.08 mmol, 77%

M.p.: degradation >170 °C

^{1}H NMR (400 MHz, $CDCl_3$): δ = 8.48 (dd, $^{3}J_{H,H}$ = 7.9 Hz, $^{4}J_{H,H}$ = 1.5 Hz, 2 H, C_{ar}H), 7.93 (dd, $^{3}J_{H,H}$ = 8.1 Hz, $^{4}J_{H,H}$ = 1.2 Hz, 1 H, C_{ar}H), 7.66–7.55 (m, 5 H, C_{ar}H), 7.38 (dt, $^{3}J_{H,H}$ = 7.7 Hz, $^{4}J_{H,H}$ = 1.4 Hz, 1 H, C_{ar}H), 7.10 (t, $^{3}J_{H,H}$ = 1.9 Hz, 1 H, C_{ar}H), 6.96 (dd, $^{3}J_{H,H}$ = 4.4 Hz, $^{4}J_{H,H}$ = 1.6 Hz, 1 H, C_{ar}H), 6.41 (dd, $^{3}J_{H,H}$ = 4.4 Hz, $^{3}J_{H,H}$ = 2.5 Hz, 1 H, C_{ar}H), 4.04 ppm (s, 3 H, N-CH_3).

^{13}C NMR (151 MHz, $CDCl_3$): δ = 150.0 (q, C_{ar}), 146.4 (q, C_{ar}), 137.9 (q, C_{ar}), 137.3 (t, C_{ar}H), 132.6 (t, C_{ar}H), 131.6 (t, C_{ar}H), 131.5 (t, C_{ar}H), 131.1 (t, C_{ar}H), 130.5 (t, C_{ar}H), 130.1 (t, C_{ar}H), 127.8 (q, C_{ar}), 118.7 (t, C_{ar}H), 112.1 (t, C_{ar}H), 105.4 (t, C_{ar}H), 34.3 ppm (p, N-CH_3).

^{125}Te NMR (189 MHz, $CDCl_3$), δ = 855 ppm.

IR (ATR): $\tilde{\nu}$ = 3106, 3049, 2988, 1571, 1510, 1494, 1473, 1447, 1434, 1416, 1362, 1345, 1327, 1254, 1237, 1204, 1167, 1109, 1072, 1041, 1013, 994, 866, 831, 762, 728, 712, 682, 644 cm^{-1}.

UV/Vis (CH_2Cl_2): λ_{max} (log ε) = 412 nm (2.89).

HRMS (ESI): [$C_{17}H_{15}N_3Br_2{}^{130}$Te-2Br+OH $^{+}$]: calculated: 408.0351; observed: 408.0349.

Synthesis of 3d:

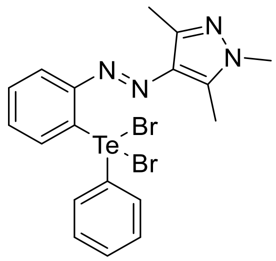

3d

Azoarene **3d** was synthesized according to general procedure (*D*).

Yield: 44 mg, 0.08 mmol, 84%

M.p.: 232 °C

^{1}H NMR (400 MHz, $CDCl_3$): δ = 8.49 (dd, $^{3}J_{H,H}$ = 8.0 Hz, $^{4}J_{H,H}$ = 1.4 Hz, 2 H, $C_{ar}H$), 7.94 (dd, $^{3}J_{H,H}$ = 8.4 Hz, $^{4}J_{H,H}$ = 1.3 Hz, 1 H, $C_{ar}H$), 7.67–7.56 (m, 5 H, $C_{ar}H$), 7.41 (dt, $^{3}J_{H,H}$ = 7.9 Hz, $^{4}J_{H,H}$ = 1.4 Hz, 1 H, $C_{ar}H$), 3.84 (s, 3 H, $C_{ar}H$), 2.70 (s, 3 H, N-CH_3), 2.67 ppm (s, 3 H, N-CH_3).

^{13}C NMR (151 MHz, $CDCl_3$): δ = 149.8 (q, C_{ar}), 143.3 (q, C_{ar}), 141.3 (q, C_{ar}), 137.4 (t, $C_{ar}H$), 135.3 (q, C_{ar}), 133.9 (q, C_{ar}), 132.5 (t, $C_{ar}H$), 131.7 (t, $C_{ar}H$), 131.6 (t, $C_{ar}H$), 131.3 (t, $C_{ar}H$), 130.1 (t, $C_{ar}H$), 127.5 (q, C_{ar}), 122.0 (t, $C_{ar}H$), 36.5 (p, N-CH_3), 14.8 (p, CH_3), 10.8 ppm (p, CH_3).

^{125}Te NMR (189 MHz, $CDCl_3$), δ = 878 ppm.

IR (ATR): $\tilde{\nu}$ = 3052, 2927, 1545, 1516, 1474, 1455, 1443, 1435, 1422, 1404, 1393, 1365, 1233, 1110, 1051, 1042, 1019, 996, 892, 755, 732, 717, 684, 670 cm^{-1}.

UV/Vis (CH_2Cl_2): λ_{max} (log ε) = 357 nm (3.03).

HRMS (ESI): [$C_{18}H_{18}$ $N_4Br_2{}^{130}$Te-2Br+OH $^{+}$]: calculated: 437.0616; observed: 436.0614.

Synthesis of 3e:

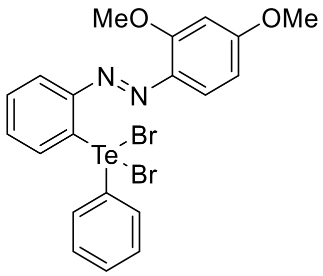

3e

Azobenzene **3e** was synthesized according to general procedure (*D*).

Yield: 50 mg, 0.08 mmol, 82%

M.p.: degradation >200 °C

^{1}H NMR (400 MHz, $CDCl_3$): δ = 8.52–8.45 (m, 2 H, $C_{ar}H$), 8.17 (dd, $^{3}J_{H,H}$ = 7.8 Hz, $^{4}J_{H,H}$ = 1.1 Hz, 1 H, $C_{ar}H$), 7.99 (d, $^{3}J_{H,H}$ = 9.7 Hz, 1 H, $C_{ar}H$), 7.75–7.62 (m, 2 H, $C_{ar}H$), 7.62–7.51 (m, 3 H, $C_{ar}H$), 7.43 (dt, $^{3}J_{H,H}$ = 7.5 Hz, $^{4}J_{H,H}$ = 1.0 Hz, 1 H, $C_{ar}H$), 6.68–6.62 (m, 2 H, $C_{ar}H$), 4.10 (s, 3 H, OCH_3), 3.93 ppm (s, 3 H, OCH_3).

^{13}C NMR (151 MHz, $CDCl_3$): δ = 165.7 (q, C_{ar}), 160.1 (q, C_{ar}), 148.3 (q, C_{ar}), 137.7 (t, $C_{ar}H$), 134.7 (q, C_{ar}), 133.3 (t, $C_{ar}H$), 132.3 (t, $C_{ar}H$), 132.1 (t, $C_{ar}H$), 131.6 (q, C_{ar}), 131.2 (t, $C_{ar}H$), 130.9 (t, $C_{ar}H$), 129.8 (t, $C_{ar}H$), 125.1 (q, C_{ar}), 119.6 (t, $C_{ar}H$), 107.0 (t, $C_{ar}H$), 98.9 (t, $C_{ar}H$), 56.2 (p, OCH_3), 56.0 ppm (p, OCH_3).

^{125}Te NMR (189 MHz, $CDCl_3$): δ = 958 ppm.

IR (ATR): $\tilde{\nu}$ = 3065, 3045, 3002, 2966, 2929, 2836, 1597, 1573, 1556, 1537, 1519, 1485, 1474, 1463, 1453, 1434, 1426, 1416, 1321, 1290, 1264, 1252, 1209, 1157, 1118, 1030, 1020, 996, 849, 796, 756, 730, 686, 624 cm^{-1}.

UV/Vis (CH_2Cl_2): λ_{max} (log ε) = 410 nm (3.15).

HRMS (ESI): [$C_{20}H_{18}N_2O_2Br_2{}^{130}Te$-2Br+OH $^{+}$]: calculated: 465.0454; observed: 465.0452.

Synthesis of 3f:

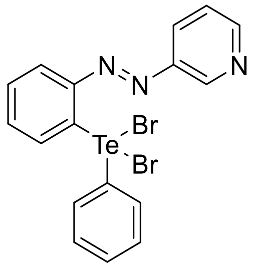

3f

Azoarene **3f** was synthesized according to general procedure (*D*).

Yield: 14 mg, 0.03 mmol, 70%

M.p.: degradation >200 °C

^{1}H NMR (400 MHz, $CDCl_3$): δ = 9.37 (s, 1 H, $C_{ar}H$), 8.84 (s, 1 H, $C_{ar}H$), 8.48 (dd, $^{3}J_{H,H}$ = 8.3 Hz, $^{4}J_{H,H}$ = 1.7 Hz, 2 H, $C_{ar}H$), 8.31 (d, $^{3}J_{H,H}$ = 8.3 Hz, 1 H, $C_{ar}H$), 8.19 (dd, $^{3}J_{H,H}$ = 8.0 Hz, $^{4}J_{H,H}$ = 1.3 Hz, 1 H, $C_{ar}H$), 7.78–7.70 (m, 2 H, $C_{ar}H$), 7.67–7.54 ppm (m, 2 H, $C_{ar}H$).

^{13}C NMR (151 MHz, $CDCl_3$): δ = 153.1 (t, $C_{ar}H$), 148.3 (q, C_{ar}), 147.5 (t, $C_{ar}H$), 137.3 (t, $C_{ar}H$), 135.2 (q, C_{ar}), 134.2 (t, $C_{ar}H$), 132.7 (t, $C_{ar}H$), 132.6 (t, $C_{ar}H$), 132.0 (t, $C_{ar}H$), 130.3 (t, $C_{ar}H$), 128.0 (q, C_{ar}), 128.0 (t, $C_{ar}H$), 127.9 (t, $C_{ar}H$), 124.7 (t, $C_{ar}H$), 124.3 ppm (t, $C_{ar}H$).

^{125}Te NMR (189 MHz, $CDCl_3$), δ = 868 ppm.

IR (ATR): $\tilde{\nu}$ = 3048, 2980, 1568, 1473, 1463, 1446, 1432, 1417, 1298, 1231, 1185, 1173, 1157, 1112, 1085, 1067, 1052, 1022, 994, 962, 928, 910, 880, 814, 770, 727, 717, 693, 682, 642, 622 cm^{-1}.

UV/Vis (CH_2Cl_2): λ_{max} (log ε) = 330 (3.50), 459 nm (2.38).

HRMS (ESI): [$C_{17}H_{13}N_3Br_2{}^{130}Te$-2Br+OH $^{+}$]: calculated: 406.0194; observed: 406.0193.

5. Computational Details

All calculations were performed by using the programs Gaussian 16[4] and Orca 5.0[5]. The geometrical parameters of the stereoisomeric structures **I–IV** of the investigated monosubstituted azoarenes **1**–**3** were optimized by means of B3LYP[6-8] with additional dispersion correction with Becke-Johnson damping[9]. The basis sets applied were def2-TZVP[10] for the light elements (H, C, O, Cl and Br) and aug-cc-pVTZ-PP[11] for tellurium. This method is abbreviated in the following as B3LYP-D3BJ/def2-TZVP, aug-cc-pVTZ-PP. For all stationary points no symmetry restriction was applied. Frequency calculations were carried out at each of the structures to verify the nature of the stationary point. It turned out that all structures have none.

The potential scans for the rotation around the N=N bond for **S1** and **S2** were performed using the functional PBE0[12] together with the dispersion correction D3[13]. As basis set def2-TZVP[10] was employed. Furthermore, implicit dichloromethane (DCM) solvation using the solvation model based on density (SMD[14]) was employed. In order to adequately describe the diradical character of the structures along the rotation, we used the open-shell variant to calculate the structures in the S_0 state, which is implemented in Gaussian via the "guess=mix" command. This command requests the HOMO and LUMO to be mixed to break α-β and spatial symmetries, which is useful for generating UHF wave functions for singlet states. For all structures C_1 symmetry was applied. Frequency calculations were carried out at the *trans* and *cis* isomers as well as at the transition states for the S_0 states. It turned out that all *trans* and *cis* isomers have no imaginary frequency and all transition states have exactly one. The structures in the excited T_1 state were computed by means of UPBE0-D3(SMD,DCM)/def2-TZVP. For these calculations, the C-N=N-C dihedral angles θ were fixed to certain values, whereas all other degrees of freedom were fully relaxed. Furthermore, on the structure of **S1** showing a C-N=N-C dihedral angles of θ = 180° a state-averaged CASSCF calculations which include two singlet states was performed. An active space of twelve electrons in ten orbitals was used. As basis set def2-TZVP and the auxiliary basis def2-TZVP/C[15] were employed for both methods.

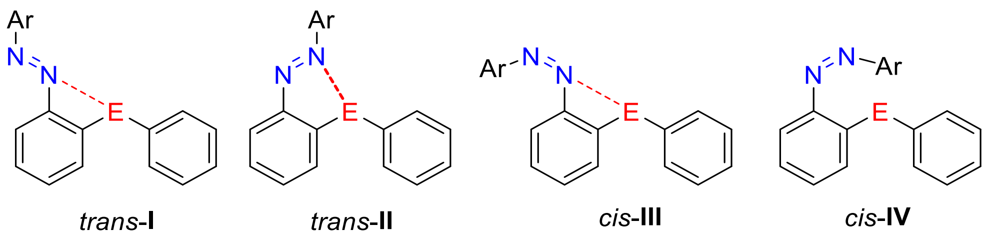

Figure S66. Stereoisomeric structures **I–IV** of the investigated monosubstituted azoarenes **1**–**3**.

Table S1. Relative energies for the different structural isomers [kcal/mol] calculated by means of B3LYP-D3BJ/def2-TZVP,aug-cc-pVTZ-PP. Energie difference ΔE between the most stable *trans*- and *cis*-isomers.

	trans-**I**	*trans*-**II**	*cis*-**III**	*cis*-**IV**	
	E [kcal/mol]	E [kcal/mol]	E [kcal/mol]	E [kcal/mol]	ΔE [kcal/mol]
**1a**	3.6	0.0	16.3	18.1	16.3
**2a**	0.8	0.0	12.9	16.0	12.9
**3a**	1.1	0.0	13.6	16.6	13.6
**1b**	3.7	0.0	17.4	18.3	17.4
**2b**	1.2	0.0	14.6	14.4	14.4
**3b**	1.5	0.0	15.2	14.9	14.9
**1c**	2.3	0.0	14.3	15.0	14.3
**2c**	0.4	0.0	12.9	20.1	12.9
**3c**	0.6	0.0	13.4	20.8	13.4
**1d**	2.1	0.0	15.9	16.5	15.9
**2d**	0.9	0.0	15.4	15.5	15.4
**3d**	1.2	0.0	16.1	16.1	16.1
**1e**	4.9	0.0	16.1	18.3	16.1
**2e**	2.9	0.0	15.4	17.5	15.4
**3e**	3.0	0.0	16.1	17.0	16.1
**1f**	3.5	0.0	16.6	18.4	16.6
**2f**	0.7	0.0	12.7	16.1	12.7
**3f**	0.4	0.0	13.7	18.4	13.7

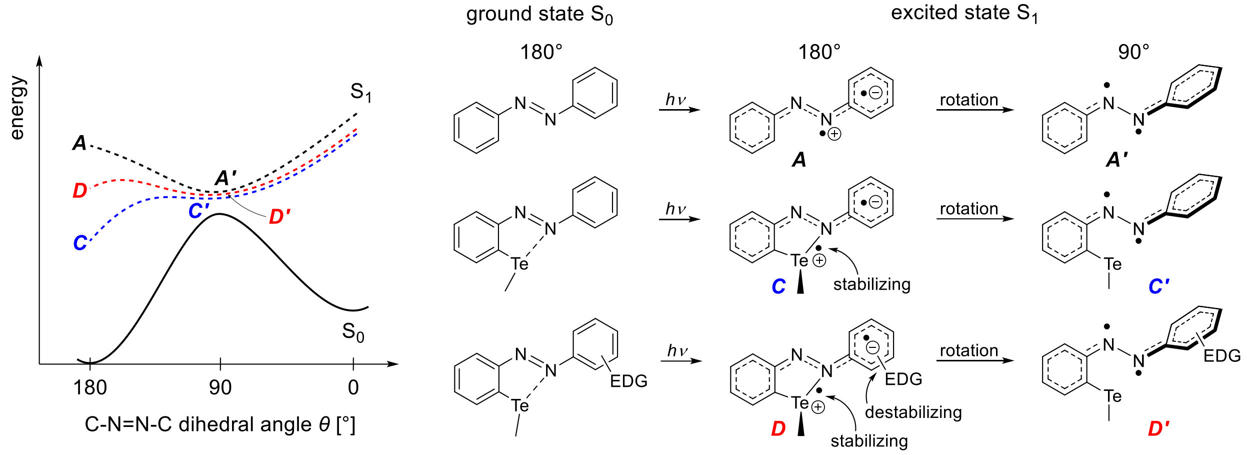

Figure S67. Potential energy profile of azobenzene (***A***) and chalcogen-substituted azo arenes (***C*** and ***D***) in the singlet ground (S_0, solid) and excited states (S_1, dashed). Upon excitation, the non-covalent chalcogen bond present in the ground state of the *trans* isomers converts into a covalent three-electron σ bond in the excited state, resulting in stabilization of ***C*** and ***D*** relative to their non-bonded counterparts ***C′*** and ***D′***.

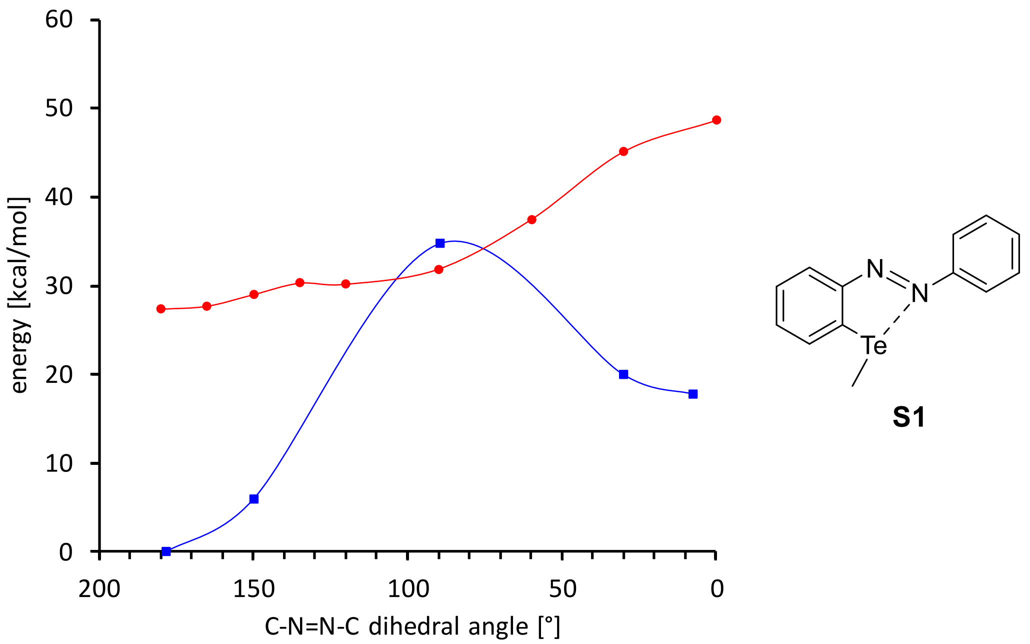

Figure S68. UPBE0-D3/def2-TZVP potential energy scan of the singlet state S_0 (blue) and the triplet state T_1 (red) of the azobenzene **S1**.

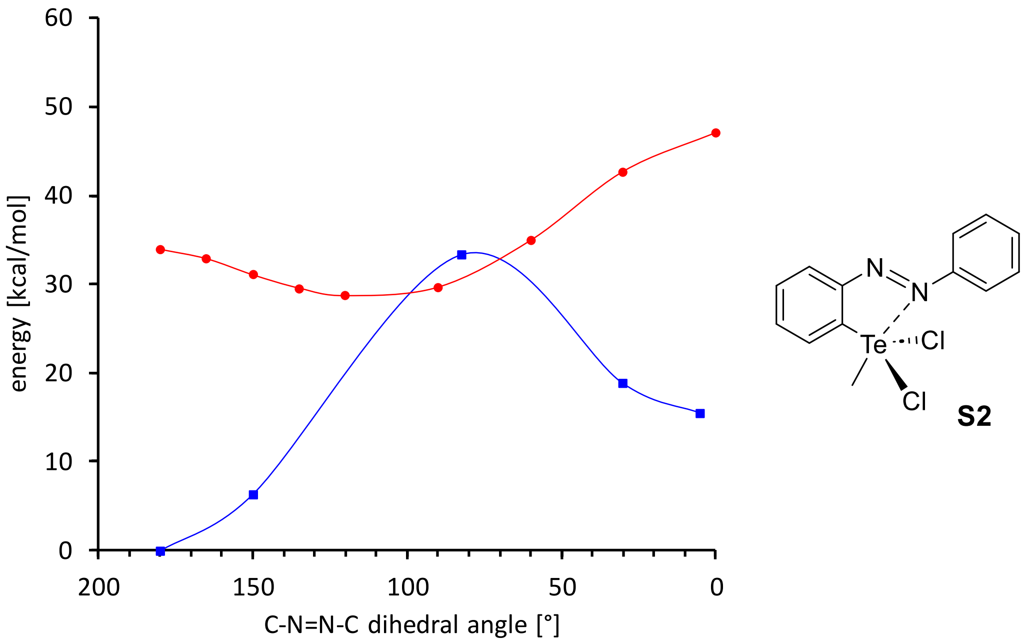

Figure S69. UPBE0-D3/def2-TZVP potential energy scan of the singlet state S_0 (blue) and the triplet state T_1 (red) of the azobenzene **S2**.

a)

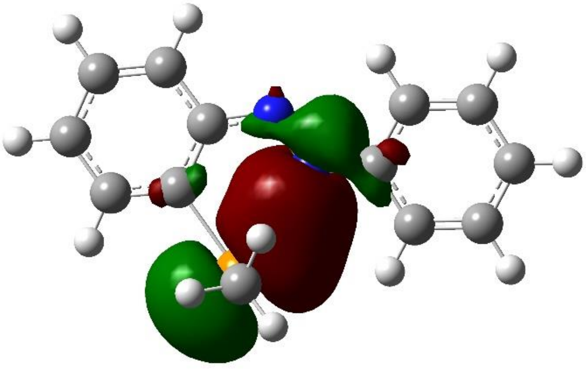

b)

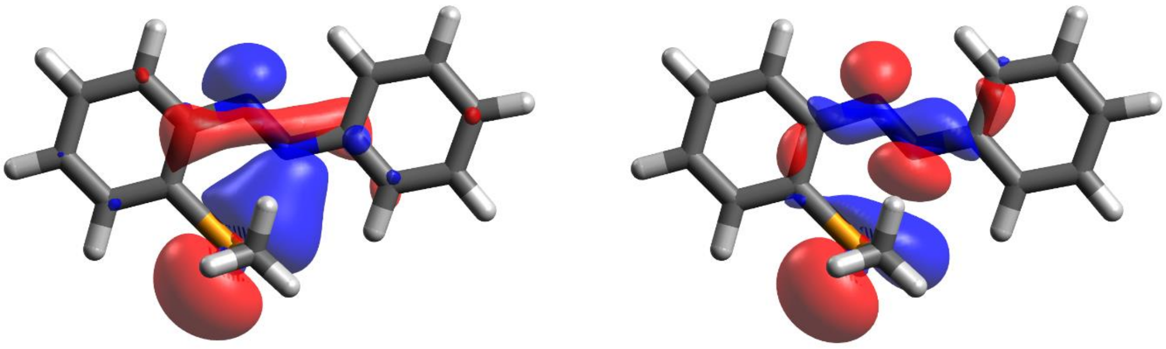

Figure S70. a) Beta spin natural bond orbitals of **S1** (triplet state; C-N=N-C dihedral angles θ = 180°) related to the Te···N intramolecular bonding interactions calculated using UPBE0-D3/def2-TZVP. Isovalue = 0.02 a.u. b) The active natural orbitals from the CASSCF(12,10) calculation of **S1** (C-N=N-C dihedral angles θ = 180°) related to the Te···N intramolecular bonding interactions. In the dominant root (weight: 0.96) of the excited state S_1, the natural orbital having a binding interaction between n_N and n_{Te} (left) shows a double occupation, while the natural orbital with an antibonding interaction between n_N and n_{Te} (right) exhibits only a single occupation. This corresponds to a covalent three-electron σ bond in the S_1 state.

6. Cartesian Coordinates and Absolute Energies for All Calculated Compounds

Table S2. Absolute energies [au] calculated by means of B3LYP-D3BJ/def2-TZVP,aug-cc-pVTZ-PP.

Compound	*E*	*G*
trans-**I** of **1a**	-1072.292631	-1072.074399
trans-**II** of **1a**	-1072.298296	-1072.078201
cis-**III** of **1a**	-1072.272274	-1072.053412
cis-**IV** of **1a**	-1072.269441	-1072.049799
trans-**I** of **2a**	-1992.802134	-1992.582386
trans-**II** of **2a**	-1992.800816	-1992.583864
cis-**III** of **2a**	-1992.781557	-1992.562675
cis-**IV** of **2a**	-1992.776634	-1992.556885
trans-**I** of **3a**	-6220.721701	-6220.506058
trans-**II** of **3a**	-6220.723483	-6220.507947
cis-**III** of **3a**	-6220.701889	-6220.485845
cis-**IV** of **3a**	-6220.697009	-6220.480188
trans-**I** of **1b**	-1105.569457	-1105.354064
trans-**II** of **1b**	-1105.575356	-1105.358409
cis-**III** of **1b**	-1105.547597	-1105.332597
cis-**IV** of **1b**	-1105.546185	-1105.330441
trans-**I** of **2b**	-2026.077644	-2025.864001
trans-**II** of **2b**	-2026.079605	-2025.864582
cis-**III** of **2b**	-2026.056420	-2025.841711
cis-**IV** of **2b**	-2026.056681	-2025.839880
trans-**I** of **3b**	-6253.998550	-6253.787055
trans-**II** of **3b**	-6254.000991	-6253.788557
cis-**III** of **3b**	-6253.976773	-6253.764747
cis-**IV** of **3b**	-6253.977226	-6253.763306
trans-**I** of **1c**	-1089.537935	-1089.308214
trans-**II** of **1c**	-1089.541633	-1089.311503
cis-**III** of **1c**	-1089.518888	-1089.291794
cis-**IV** of **1c**	-1089.517800	-1089.289776
trans-**I** of **2c**	-2010.048042	-2009.819451
trans-**II** of **2c**	-2010.048615	-2009.819612
cis-**III** of **2c**	-2010.028082	-2009.801088
cis-**IV** of **2c**	-2010.016498	-2009.786845
trans-**I** of **3c**	-6237.969050	-6237.743225
trans-**II** of **3c**	-6237.970033	-6237.743920
cis-**III** of **3c**	-6237.948724	-6237.724625
cis-**IV** of **3c**	-6237.936887	-6237.710209
trans-**I** of **1d**	-1184.253014	-1183.984942
trans-**II** of **1d**	-1184.256349	-1183.987488
cis-**III** of **1d**	-1184.230945	-1183.962357
cis-**IV** of **1d**	-1184.230033	-1183.960746
trans-**I** of **2d**	-2104.762807	-2104.496145
trans-**II** of **2d**	-2104.764216	-2104.496557
cis-**III** of **2d**	-2104.739693	-2104.472217
cis-**IV** of **2d**	-2104.739544	-2104.470022
trans-**I** of **3d**	-6332.683859	-6332.419963
trans-**II** of **3d**	-6332.685725	-6332.420631
cis-**III** of **3d**	-6332.660054	-6332.395644
cis-**IV** of **3d**	-6332.660009	-6332.393423
trans-**I** of **1e**	-1301.444660	-1301.169007
trans-**II of 1e**	-1301.452448	-1301.173457
cis-**III** of **1e**	-1301.426809	-1301.149068
cis-**IV** of **1e**	-1301.423327	-1301.144743
trans-**I** of **2e**	-2221.954953	-2221.677624
trans-**II** of **2e**	-2221.959508	-2221.682218
cis-**III** of **2e**	-2221.934906	-2221.657411
cis-**IV** of **2e**	-2221.932371	-2221.654530
trans-**I** of **3e**	-6449.876247	-6449.601554
trans-**II** of **3e**	-6449.881001	-6449.606388
cis-**III** of **3e**	-6449.855278	-6449.580666
cis-**IV** of **3e**	-6449.853053	-6449.578408

trans-**I** of **1f**	-1088.331549	-1088.124804
trans-**II** of **1f**	-1088.337109	-1088.129006
cis-**III** of **1f**	-1088.310709	-1088.103514
cis-**IV** of **1f**	-1088.307754	-1088.099822
trans-**I** of **2f**	-2008.839297	-2008.632447
trans-**II** **2f**	-2008.839951	-2008.633252
cis-**III** of **2f**	-2008.81973	-2008.612550
cis-**IV** of **2f**	-2008.814348	-2008.606314
trans-**I** of **3f**	-6236.760140	-6236.556128
trans-**II** **3f**	-6236.761185	-6236.557398
cis-**III** of **3f**	-6236.739303	-6236.535089
cis-**IV** of **3f**	-6236.734530	-6236.529455

Cartesian coordinates of the optimized geometry for *trans*-**I** of **1a** at B3LYP-D3BJ/def2-TZVP,aug-cc-pVTZ-PP level of theory (number of imaginary frequencies = 0):

N	2.90870000	1.08390000	0.00000000
C	3.88970000	0.06730000	0.00000000
C	5.21270000	0.50860000	0.00000000
H	5.39930000	1.57420000	0.00010000
C	6.25420000	-0.40890000	0.00000000
H	7.27940000	-0.06290000	0.00010000
C	5.97650000	-1.77020000	0.00000000
H	6.78590000	-2.48870000	0.00000000
C	4.65340000	-2.21270000	0.00000000
H	4.44080000	-3.27400000	-0.00010000
C	3.61020000	-1.30430000	0.00000000
C	0.72380000	1.67940000	0.00000000
C	-0.58710000	1.18720000	0.00000000
C	0.96360000	3.05670000	0.00000000
C	-1.65390000	2.07890000	0.00000000
C	-0.10290000	3.93700000	0.00000000
H	1.98650000	3.40560000	0.00000000
C	-1.40930000	3.44620000	0.00000000
H	-2.67100000	1.71310000	0.00000000
H	0.07530000	5.00420000	0.00000000
H	-2.24450000	4.13530000	0.00000000
N	1.72540000	0.69130000	0.00000000
Te	-0.79640000	-0.92820000	0.00000000
C	-2.92970000	-0.87470000	0.00000000
C	-3.63210000	-0.85620000	1.20420000
C	-3.63210000	-0.85630000	-1.20420000
C	-5.02210000	-0.81330000	1.20300000
H	-3.09310000	-0.86930000	2.14220000
C	-5.02210000	-0.81330000	-1.20300000
H	-3.09310000	-0.86930000	-2.14220000
C	-5.71810000	-0.79130000	0.00000000
H	-6.80000000	-0.75840000	0.00000000
H	-5.56000000	-0.79710000	2.14250000
H	-5.56000000	-0.79720000	-2.14240000
H	2.58220000	-1.63500000	-0.00010000

Cartesian coordinates of the optimized geometry for *trans*-**II** of **1a** at B3LYP-D3BJ/def2-TZVP,aug-cc-pVTZ-PP level of theory (number of imaginary frequencies = 0):

N	-1.99690000	0.31310000	-0.07690000
C	-3.28210000	-0.26670000	-0.01370000
C	-3.36440000	-1.62880000	-0.30480000
H	-2.46290000	-2.16130000	-0.57510000
C	-4.59020000	-2.27620000	-0.25880000
H	-4.65080000	-3.33140000	-0.49040000
C	-5.73680000	-1.56990000	0.08590000
H	-6.69320000	-2.07470000	0.12650000
C	-5.65290000	-0.21100000	0.38390000
H	-6.54550000	0.33670000	0.65800000
C	-4.43430000	0.44440000	0.33580000

C	-0.65510000	2.10250000	-0.04920000
C	0.53290000	1.34190000	-0.07250000
C	-0.59830000	3.50310000	-0.05190000
C	1.74910000	2.02340000	-0.10580000
C	0.61480000	4.15930000	-0.08550000
H	-1.53630000	4.04190000	-0.02990000
C	1.79080000	3.41000000	-0.11300000
H	2.67540000	1.46810000	-0.12390000
H	0.65280000	5.24030000	-0.08990000
H	2.75120000	3.90930000	-0.13860000
N	-1.94210000	1.56390000	-0.01600000
Te	0.45380000	-0.77050000	-0.04260000
C	2.59840000	-0.99140000	0.04700000
C	3.33860000	-1.16560000	-1.12180000
C	3.25750000	-1.00050000	1.27600000
C	4.71780000	-1.33830000	-1.06380000
H	2.83890000	-1.15940000	-2.08240000
C	4.63630000	-1.17160000	1.33500000
H	2.69380000	-0.86670000	2.19080000
C	5.36790000	-1.34020000	0.16480000
H	6.44110000	-1.47440000	0.21050000
H	5.28340000	-1.47020000	-1.97770000
H	5.13850000	-1.17430000	2.29430000
H	-4.35200000	1.49610000	0.56760000

Cartesian coordinates of the optimized geometry for *cis*-**III** of **1a** at B3LYP-D3BJ/def2-TZVP,aug-cc-pVTZ-PP level of theory (number of imaginary frequencies = 0):

N	-2.70800000	-1.45210000	-0.76530000
C	-3.58670000	-0.66660000	0.04190000
C	-3.15290000	-0.05790000	1.22030000
H	-2.10910000	-0.10510000	1.49790000
C	-4.07050000	0.59200000	2.03060000
H	-3.73650000	1.05590000	2.94960000
C	-5.41240000	0.65430000	1.66620000
H	-6.12220000	1.17140000	2.29820000
C	-5.84180000	0.03100000	0.49950000
H	-6.88690000	0.06240000	0.22030000
C	-4.93630000	-0.65300000	-0.29950000
C	-1.16230000	0.31110000	-1.01270000
C	0.17320000	0.47060000	-0.63350000
C	-1.93460000	1.41620000	-1.36990000
C	0.70530000	1.75180000	-0.53710000
C	-1.38270000	2.68560000	-1.31400000
H	-2.95600000	1.27470000	-1.69370000
C	-0.06950000	2.85280000	-0.88180000
H	1.72790000	1.89230000	-0.21610000
H	-1.97570000	3.54340000	-1.60180000
H	0.36090000	3.84460000	-0.82800000
N	-1.60790000	-1.03630000	-1.15650000
Te	1.25810000	-1.30610000	-0.20120000
C	3.05890000	-0.28060000	0.29740000
C	3.98310000	0.03390000	-0.69780000
C	3.32000000	0.08100000	1.61790000
C	5.15380000	0.71130000	-0.37480000
H	3.78570000	-0.24490000	-1.72430000
C	4.49240000	0.75690000	1.93830000
H	2.60600000	-0.16020000	2.39410000
C	5.40920000	1.07330000	0.94270000
H	6.32150000	1.59920000	1.19320000
H	5.86590000	0.95420000	-1.15310000
H	4.68790000	1.03600000	2.96590000
H	-5.25720000	-1.17100000	-1.19370000

Cartesian coordinates of the optimized geometry for *cis*-**IV** of **1a** at B3LYP-D3BJ/def2-TZVP,aug-cc-pVTZ-PP level of theory (number of imaginary frequencies = 0):

N	2.50410000	0.41740000	-1.75060000
C	2.87170000	-0.30310000	-0.57080000
C	3.05110000	-1.67670000	-0.74100000
H	2.84560000	-2.10680000	-1.71200000
C	3.46970000	-2.46170000	0.32230000
H	3.58050000	-3.53000000	0.19130000
C	3.76550000	-1.87240000	1.54600000
H	4.10650000	-2.48060000	2.37340000
C	3.64020000	-0.49500000	1.70000000
H	3.89150000	-0.03110000	2.64490000
C	3.18780000	0.29260000	0.65300000
C	1.03570000	1.87720000	-0.61920000
C	0.08310000	1.12200000	0.07710000
C	1.20610000	3.23260000	-0.32230000
C	-0.63920000	1.73990000	1.09910000
C	0.49940000	3.82280000	0.70930000
H	1.91010000	3.80130000	-0.91590000
C	-0.42980000	3.07130000	1.42380000
H	-1.37950000	1.17110000	1.64470000
H	0.65890000	4.86660000	0.94550000
H	-1.00050000	3.52350000	2.22440000
N	1.76050000	1.40830000	-1.75590000
Te	-0.35800000	-0.91280000	-0.38370000
C	-2.45300000	-0.67670000	-0.08460000
C	-3.17770000	0.24390000	-0.84080000
C	-3.10910000	-1.46340000	0.85900000
C	-4.54470000	0.38380000	-0.64130000
H	-2.67460000	0.85320000	-1.57960000
C	-4.48280000	-1.33380000	1.04170000
H	-2.54980000	-2.16840000	1.46010000
C	-5.20080000	-0.40770000	0.29650000
H	-6.26760000	-0.30120000	0.44470000
H	-4.98670000	-1.94990000	1.77570000
H	-5.10040000	1.10550000	-1.22660000
H	3.08900000	1.35950000	0.78190000

Cartesian coordinates of the optimized geometry for *trans*-**I** of **2a** at B3LYP-D3BJ/def2-TZVP,aug-cc-pVTZ-PP level of theory (number of imaginary frequencies = 0):

N	2.10880000	0.43200000	0.17630000
C	3.36320000	-0.18710000	0.03330000
C	3.46200000	-1.48890000	0.52460000
H	2.59940000	-1.93680000	0.99890000
C	4.66190000	-2.17660000	0.41950000
H	4.74260000	-3.18330000	0.80710000
C	5.75520000	-1.57180000	-0.18910000
H	6.68940000	-2.11040000	-0.28030000
C	5.64990000	-0.27460000	-0.69080000
H	6.50160000	0.18720000	-1.17300000
C	4.46040000	0.42290000	-0.58250000
C	0.75000000	2.20720000	0.08530000
C	-0.43960000	1.47350000	0.19460000
C	0.69980000	3.60110000	0.08970000
C	-1.64340000	2.13650000	0.34850000
C	-0.50950000	4.26260000	0.23340000
H	1.63290000	4.13920000	-0.00920000
C	-1.68020000	3.52920000	0.36970000
H	-2.56250000	1.57840000	0.45080000
H	-0.53770000	5.34390000	0.24250000
H	-2.63050000	4.03310000	0.48730000
N	2.04390000	1.65800000	-0.02730000
Te	-0.42700000	-0.66030000	0.12620000
C	-2.55310000	-0.86360000	-0.21250000
C	-3.33120000	-1.47510000	0.76590000

C	-3.12500000	-0.45200000	-1.41300000
C	-4.68970000	-1.67030000	0.54010000
H	-2.89230000	-1.77640000	1.70620000
C	-4.48500000	-0.64210000	-1.62470000
H	-2.51680000	0.00680000	-2.17870000
C	-5.26810000	-1.25240000	-0.65150000
H	-6.32640000	-1.40220000	-0.82250000
H	-5.29340000	-2.14590000	1.30220000
H	-4.92990000	-0.31500000	-2.55570000
H	4.35620000	1.42480000	-0.97250000
Cl	0.03110000	-0.44630000	-2.34310000
Cl	-0.74640000	-0.58920000	2.63410000

Cartesian coordinates of the optimized geometry for *trans*-**II** of **2a** at B3LYP-D3BJ/def2-TZVP,aug-cc-pVTZ-PP level of theory (number of imaginary frequencies = 0):

N	-3.02000000	1.15900000	-0.11680000
C	-3.94110000	0.09220000	-0.08760000
C	-5.26170000	0.42710000	0.21140000
H	-5.50180000	1.46290000	0.41050000
C	-6.23080000	-0.56390000	0.26350000
H	-7.25280000	-0.30800000	0.50980000
C	-5.88800000	-1.88270000	-0.01270000
H	-6.64570000	-2.65500000	0.01460000
C	-4.57370000	-2.21080000	-0.34320000
H	-4.31670000	-3.23470000	-0.58140000
C	-3.59570000	-1.23240000	-0.37970000
C	-0.86140000	1.85020000	-0.12800000
C	0.45600000	1.41490000	-0.00950000
C	-1.12570000	3.20620000	-0.32740000
C	1.51670000	2.30060000	-0.06540000
C	-0.06800000	4.09710000	-0.39440000
H	-2.15170000	3.53180000	-0.42310000
C	1.24690000	3.65010000	-0.26290000
H	2.53390000	1.95400000	0.04680000
H	-0.26310000	5.15040000	-0.54750000
H	2.06530000	4.35620000	-0.31160000
N	-1.82320000	0.82650000	-0.03570000
Te	0.66740000	-0.67330000	0.32260000
C	2.77220000	-0.75410000	-0.10110000
C	3.24590000	-0.42030000	-1.36560000
C	3.64260000	-1.17780000	0.89670000
C	4.60890000	-0.49810000	-1.62360000
H	2.56270000	-0.11420000	-2.14400000
C	5.00300000	-1.26600000	0.62210000
H	3.27390000	-1.41240000	1.88460000
C	5.48730000	-0.92340000	-0.63380000
H	6.54750000	-0.98820000	-0.84180000
H	4.98060000	-0.23020000	-2.60420000
H	5.68160000	-1.59810000	1.39690000
H	-2.58010000	-1.46670000	-0.66440000
Cl	0.12720000	-1.07420000	-2.11140000
Cl	1.14720000	-0.10160000	2.73470000

Cartesian coordinates of the optimized geometry for *cis*-**III** of **2a** at B3LYP-D3BJ/def2-TZVP,aug-cc-pVTZ-PP level of theory (number of imaginary frequencies = 0):

N	2.78110000	-1.07000000	-1.14850000
C	3.76240000	-0.17450000	-0.63250000
C	3.44480000	1.14590000	-0.31310000
H	2.42460000	1.49110000	-0.40790000
C	4.45550000	2.00590000	0.08780000
H	4.21670000	3.03500000	0.32270000
C	5.76820000	1.55440000	0.18870000
H	6.54990000	2.22950000	0.51120000
C	6.07860000	0.24180000	-0.15180000

H	7.10120000	-0.10740000	-0.09330000
C	5.08200000	-0.61770000	-0.59150000
C	1.33950000	-0.79530000	0.71040000
C	0.00000000	-0.45250000	0.86590000
C	2.16820000	-0.83630000	1.83200000
C	-0.51530000	-0.08750000	2.09650000
C	1.65090000	-0.50000000	3.07330000
H	3.20100000	-1.13520000	1.73180000
C	0.32080000	-0.11120000	3.20700000
H	-1.55610000	0.18410000	2.19930000
H	2.29090000	-0.54110000	3.94480000
H	-0.07350000	0.15080000	4.17970000
N	1.69400000	-1.26460000	-0.59220000
Te	-1.17930000	-0.57960000	-0.89590000
C	-2.84520000	0.55710000	-0.15360000
C	-4.09290000	-0.05260000	-0.08850000
C	-2.68120000	1.88790000	0.21480000
C	-5.18730000	0.68220000	0.35430000
H	-4.21200000	-1.09280000	-0.35400000
C	-3.77940000	2.60710000	0.67010000
H	-1.71470000	2.36330000	0.13770000
C	-5.03190000	2.00810000	0.73730000
H	-5.88530000	2.57490000	1.08630000
H	-6.15990000	0.21020000	0.40250000
H	-3.65230000	3.64020000	0.96650000
H	5.30730000	-1.63250000	-0.89150000
Cl	-0.04180000	1.55320000	-1.67470000
Cl	-2.16210000	-2.68660000	0.05070000

Cartesian coordinates of the optimized geometry for *cis*-**IV** of **2a** at B3LYP-D3BJ/def2-TZVP,aug-cc-pVTZ-PP level of theory (number of imaginary frequencies = 0):

N	-2.63530000	0.88780000	1.55770000
C	-2.96330000	-0.12420000	0.60250000
C	-3.06270000	-1.41830000	1.11680000
H	-2.83840000	-1.57770000	2.16320000
C	-3.43330000	-2.46600000	0.28920000
H	-3.47660000	-3.47290000	0.68230000
C	-3.76530000	-2.21840000	-1.03740000
H	-4.06400000	-3.03360000	-1.68250000
C	-3.71990000	-0.92080000	-1.53420000
H	-3.99170000	-0.72540000	-2.56300000
C	-3.31050000	0.12810000	-0.72640000
C	-1.21130000	2.05170000	0.08290000
C	-0.19720000	1.22220000	-0.39820000
C	-1.48760000	3.24280000	-0.59120000
C	0.47550000	1.54620000	-1.57060000
C	-0.82490000	3.55720000	-1.76460000
H	-2.24020000	3.90160000	-0.17850000
C	0.15650000	2.70540000	-2.26170000
H	1.24470000	0.88920000	-1.95020000
H	-1.06690000	4.47310000	-2.28750000
H	0.68460000	2.94810000	-3.17380000
N	-1.90940000	1.86040000	1.31610000
Te	0.38700000	-0.56480000	0.61190000
C	2.44510000	-0.51020000	0.02400000
C	3.21670000	0.61260000	0.29800000
C	2.99170000	-1.62490000	-0.59900000
C	4.55430000	0.62020000	-0.07600000
H	2.78780000	1.46430000	0.80470000
C	4.33690000	-1.61190000	-0.95170000
H	2.37780000	-2.48210000	-0.83360000
C	5.11590000	-0.49090000	-0.69550000
H	6.16060000	-0.48200000	-0.97780000
H	4.76960000	-2.47930000	-1.43280000
H	5.15760000	1.49590000	0.12450000

H	-3.27390000	1.13220000	-1.11980000
Cl	0.94570000	0.79870000	2.63510000
Cl	-0.14830000	-1.84140000	-1.50280000

Cartesian coordinates of the optimized geometry for *trans*-**I** of **3a** at B3LYP-D3BJ/def2-TZVP,aug-cc-pVTZ-PP level of theory (number of imaginary frequencies = 0):

N	3.10230000	0.99670000	0.72810000
C	4.01740000	0.15190000	0.06900000
C	5.32680000	0.62320000	-0.02880000
H	5.56100000	1.59330000	0.38850000
C	6.29140000	-0.14360000	-0.66510000
H	7.30410000	0.22670000	-0.75450000
C	5.95650000	-1.39240000	-1.17670000
H	6.71110000	-1.99760000	-1.66200000
C	4.65460000	-1.87560000	-1.05040000
H	4.40440000	-2.85820000	-1.42850000
C	3.68060000	-1.11050000	-0.43360000
C	0.95140000	1.51920000	1.21420000
C	-0.37280000	1.20460000	0.91880000
C	1.23200000	2.53410000	2.13000000
C	-1.42500000	1.89220000	1.49560000
C	0.18340000	3.21820000	2.72140000
H	2.26310000	2.76590000	2.35560000
C	-1.13840000	2.90300000	2.40610000
H	-2.44780000	1.65660000	1.24050000
H	0.39120000	4.00770000	3.43170000
H	-1.94970000	3.44960000	2.86860000
N	1.90090000	0.74000000	0.52720000
Te	-0.59580000	-0.34040000	-0.52410000
C	-2.68760000	-0.68110000	-0.17960000
C	-3.14660000	-1.02310000	1.08800000
C	-3.56280000	-0.60230000	-1.25680000
C	-4.50050000	-1.27010000	1.27750000
H	-2.45960000	-1.11030000	1.91700000
C	-4.91330000	-0.86700000	-1.05870000
H	-3.20770000	-0.31330000	-2.23570000
C	-5.38340000	-1.19610000	0.20620000
H	-6.43620000	-1.39590000	0.35760000
H	-4.86100000	-1.52850000	2.26470000
H	-5.59560000	-0.80810000	-1.89660000
H	2.67620000	-1.48570000	-0.30120000
Br	-1.14160000	1.60390000	-2.32510000
Br	0.07380000	-2.17440000	1.34970000

Cartesian coordinates of the optimized geometry for *trans*-**II** of **3a** at B3LYP-D3BJ/def2-TZVP,aug-cc-pVTZ-PP level of theory (number of imaginary frequencies = 0):

N	-2.12890000	0.40860000	-0.34470000
C	-3.37860000	-0.17360000	-0.07080000
C	-3.43640000	-1.56520000	-0.15040000
H	-2.54810000	-2.11180000	-0.43650000
C	-4.62970000	-2.22130000	0.11170000
H	-4.67900000	-3.29970000	0.04210000
C	-5.75730000	-1.49120000	0.46830000
H	-6.68670000	-2.00250000	0.68240000
C	-5.69310000	-0.10090000	0.55940000
H	-6.57160000	0.46130000	0.84800000
C	-4.51020000	0.56370000	0.29040000
C	-0.80050000	2.15960000	-0.75520000
C	0.40040000	1.45270000	-0.59650000
C	-0.76390000	3.48900000	-1.17490000
C	1.60290000	2.06440000	-0.90060000
C	0.44380000	4.10360000	-1.46530000
H	-1.70560000	4.01120000	-1.27650000
C	1.62620000	3.38780000	-1.33500000

H	2.53060000	1.52020000	-0.80330000
H	0.46190000	5.13320000	-1.79620000
H	2.57530000	3.85340000	-1.56580000
N	-2.09090000	1.63840000	-0.53320000
Te	0.39250000	-0.56550000	0.09960000
C	2.51630000	-0.65890000	0.49820000
C	3.27690000	-1.60500000	-0.18240000
C	3.10150000	0.14790000	1.47010000
C	4.63010000	-1.73830000	0.10950000
H	2.83010000	-2.21960000	-0.95110000
C	4.45640000	0.01520000	1.74710000
H	2.50760000	0.86840000	2.01390000
C	5.22130000	-0.92800000	1.07020000
H	6.27560000	-1.03110000	1.29230000
H	5.21990000	-2.47430000	-0.42160000
H	4.91090000	0.64770000	2.49870000
H	-4.43740000	1.63890000	0.36470000
Br	-0.16080000	0.40330000	2.55870000
Br	0.69250000	-1.29830000	-2.49270000

Cartesian coordinates of the optimized geometry for *cis*-**III** of **3a** at B3LYP-D3BJ/def2-TZVP,aug-cc-pVTZ-PP level of theory (number of imaginary frequencies = 0):

N	2.84980000	-0.83580000	-1.30740000
C	3.92370000	-0.24090000	-0.58460000
C	3.75590000	0.95670000	0.11110000
H	2.78120000	1.42320000	0.15080000
C	4.85530000	1.54860000	0.71310000
H	4.73290000	2.48520000	1.24130000
C	6.10850000	0.94720000	0.64110000
H	6.96010000	1.41070000	1.12130000
C	6.27030000	-0.23630000	-0.07210000
H	7.24680000	-0.69650000	-0.14670000
C	5.18490000	-0.81780000	-0.71170000
C	1.43280000	-0.96670000	0.59000000
C	0.11950000	-0.59280000	0.86010000
C	2.25280000	-1.38240000	1.63970000
C	-0.37380000	-0.55950000	2.15160000
C	1.75620000	-1.37810000	2.93390000
H	3.26170000	-1.71230000	1.44230000
C	0.45610000	-0.95370000	3.19490000
H	-1.39500000	-0.26490000	2.34530000
H	2.38850000	-1.70980000	3.74680000
H	0.07770000	-0.95440000	4.20840000
N	1.74600000	-1.06680000	-0.80040000
Te	-1.05560000	-0.16260000	-0.85800000
C	-2.67940000	0.78440000	0.18110000
C	-3.96680000	0.30110000	-0.02250000
C	-2.45270000	1.88850000	0.99540000
C	-5.03770000	0.93150000	0.60100000
H	-4.13840000	-0.57050000	-0.63760000
C	-3.52870000	2.50010000	1.62660000
H	-1.45460000	2.27940000	1.12730000
C	-4.82030000	2.02590000	1.42810000
H	-5.65590000	2.51030000	1.91650000
H	-6.04080000	0.55840000	0.44110000
H	-3.35350000	3.35400000	2.26790000
H	5.29570000	-1.72040000	-1.29790000
Br	0.25680000	2.21010000	-1.06200000
Br	-2.18610000	-2.58590000	-0.54420000

Cartesian coordinates of the optimized geometry for *cis*-**IV** of **3a** at B3LYP-D3BJ/def2-TZVP,aug-cc-pVTZ-PP level of theory (number of imaginary frequencies = 0):

N	-2.47510000	-1.87210000	-0.81540000
C	-2.96560000	-0.54620000	-0.60510000

C	-3.09220000	0.23170000	-1.75770000
H	-2.76110000	-0.18300000	-2.70060000
C	-3.62810000	1.50680000	-1.67710000
H	-3.69410000	2.12000000	-2.56570000
C	-4.09610000	1.98720000	-0.45980000
H	-4.52420000	2.97850000	-0.39710000
C	-4.01710000	1.19200000	0.67770000
H	-4.39180000	1.56110000	1.62310000
C	-3.44360000	-0.06810000	0.61700000
C	-1.14990000	-1.87620000	1.13440000
C	-0.22540000	-0.83050000	1.12420000
C	-1.43570000	-2.51420000	2.34350000
C	0.34480000	-0.38760000	2.31290000
C	-0.87580000	-2.06440000	3.52590000
H	-2.11400000	-3.35710000	2.32920000
C	0.01390000	-0.99430000	3.51460000
H	1.04110000	0.43820000	2.30280000
H	-1.12570000	-2.55490000	4.45750000
H	0.46100000	-0.64100000	4.43380000
N	-1.72470000	-2.46770000	-0.03260000
Te	0.36060000	0.14750000	-0.68110000
C	2.35910000	0.60880000	-0.07200000
C	3.16900000	-0.37870000	0.47500000
C	2.83400000	1.89690000	-0.28080000
C	4.47180000	-0.06000000	0.83450000
H	2.79870000	-1.38440000	0.60910000
C	4.14680000	2.19850000	0.06500000
H	2.18920000	2.66630000	-0.68040000
C	4.96260000	1.22440000	0.62590000
H	5.98130000	1.46490000	0.90070000
H	4.52430000	3.19910000	-0.09960000
H	5.10480000	-0.82160000	1.27080000
H	-3.38350000	-0.67910000	1.50440000
Br	1.23330000	-2.17840000	-1.69210000
Br	-0.48180000	2.49690000	0.37710000

Cartesian coordinates of the optimized geometry for *trans*-**I** of **1b** at B3LYP-D3BJ/def2-TZVP,aug-cc-pVTZ-PP level of theory (number of imaginary frequencies = 0):

C	1.75959100	2.06811800	0.00009600
C	0.69882400	1.16960600	0.00002900
C	-0.61529600	1.65207300	0.00001400
C	-0.86574000	3.02728800	0.00006900
C	0.19498500	3.91490500	0.00013600
C	1.50487400	3.43391200	0.00015000
H	2.77920100	1.70953400	0.00010700
H	-1.89157100	3.36814100	0.00005600
H	0.00905300	4.98089400	0.00017900
H	2.33511000	4.12905700	0.00020300
N	-1.60829800	0.65586800	-0.00006300
N	-2.79641500	1.04401600	-0.00010000
Te	0.91811200	-0.94529100	-0.00005700
C	3.05188700	-0.87914700	-0.00001200
C	3.75456100	-0.85625700	1.20394300
C	3.75460600	-0.85608300	-1.20393600
C	5.14432800	-0.80464100	1.20296800
H	3.21558500	-0.87219300	2.14194500
C	5.14437300	-0.80446600	-1.20290200
H	3.21566500	-0.87188400	-2.14196100
C	5.84028700	-0.77819500	0.00004800
H	5.68206100	-0.78477100	2.14248300
H	5.68214000	-0.78445800	-2.14239400
H	6.92200800	-0.73823200	0.00007100
C	-3.73247300	0.01729200	-0.00009300
C	-3.56890800	-1.39071400	-0.00017100
N	-5.68605800	-0.79810000	-0.00041100

C	-4.85094200	-1.87558700	-0.00028000
H	-2.64785600	-1.94355400	-0.00029700
H	-5.23432600	-2.88197000	-0.00039000
N	-5.02496700	0.35886700	-0.00019400
C	-7.13272600	-0.79983300	0.00082000
H	-7.48054300	-1.83031000	-0.00517200
H	-7.50531300	-0.29500400	0.89158600
H	-7.50624400	-0.28431400	-0.88332200

Cartesian coordinates of the optimized geometry for *trans*-**II** of **1b** at B3LYP-D3BJ/def2-TZVP,aug-cc-pVTZ-PP level of theory (number of imaginary frequencies = 0):

C	1.94897000	1.96679500	-0.00000400
C	0.67916300	1.38855700	0.00001200
C	-0.43978800	2.24900000	-0.00002200
C	-0.26586500	3.63999500	-0.00005900
C	0.99859700	4.19130100	-0.00007000
C	2.10754000	3.34469300	-0.00004400
H	2.82552500	1.33578000	0.00001700
H	-1.15564300	4.25567800	-0.00008200
H	1.12812300	5.26522200	-0.00010100
H	3.10702600	3.76121900	-0.00005500
N	-1.76612900	1.81677100	-0.00002600
N	-1.90659500	0.56793200	-0.00003900
Te	0.41290500	-0.71061500	0.00008600
C	2.53873600	-1.10670700	0.00002000
C	3.23050000	-1.25682200	1.20167000
C	3.23043700	-1.25674200	-1.20167500
C	4.59181400	-1.54259100	1.20275400
H	2.70600500	-1.14328200	2.14231400
C	4.59175000	-1.54251500	-1.20284900
H	2.70589400	-1.14313600	-2.14228400
C	5.27421000	-1.68467300	-0.00007000
H	5.11882700	-1.65408400	2.14222900
H	5.11871400	-1.65394700	-2.14235800
H	6.33365000	-1.90725600	-0.00010500
C	-3.21638900	0.10637000	-0.00003000
C	-4.44388300	0.81116400	0.00001900
N	-4.73490300	-1.36869300	-0.00002400
C	-5.39509600	-0.17821200	0.00001400
H	-4.58606100	1.87583500	0.00006000
H	-6.47105000	-0.13372900	0.00004000
N	-3.40904600	-1.21512100	-0.00006200
C	-5.30347600	-2.69863600	-0.00011600
H	-6.38783100	-2.61459200	0.00025200
H	-4.98071900	-3.24174700	-0.88780700
H	-4.98014000	-3.24208000	0.88715600

Cartesian coordinates of the optimized geometry for *cis*-**III** of **1b** at B3LYP-D3BJ/def2-TZVP,aug-cc-pVTZ-PP level of theory (number of imaginary frequencies = 0):

C	0.88529500	1.88578500	-0.01727800
C	0.30052700	0.73757300	-0.54278700
C	-0.99536400	0.80703800	-1.05669800
C	-1.67107800	2.02459600	-1.11665300
C	-1.06634900	3.17077500	-0.62620800
C	0.20507400	3.09704800	-0.06296800
H	1.87752100	1.84183700	0.40964500
H	-2.66267100	2.05977500	-1.54798500
H	-1.58492800	4.11889600	-0.67993800
H	0.67821400	3.98886200	0.32747200
N	-1.52786800	-0.37419300	-1.64880400
N	-2.61345300	-0.88155100	-1.31549000
Te	1.24634100	-1.16641500	-0.58870900
C	3.06521900	-0.47200500	0.27653800
C	3.26929900	-0.56344900	1.65224100
C	4.05871500	0.07868200	-0.53168000

C	4.45505200	-0.10276700	2.21456500
H	2.50101000	-0.98897800	2.28385300
C	5.24167000	0.54145400	0.03377900
H	3.90559600	0.15092100	-1.60012200
C	5.44098500	0.45096800	1.40655800
H	4.60641600	-0.17605900	3.28406600
H	6.00771700	0.97041100	-0.59962200
H	6.36325700	0.80960700	1.84519400
C	-3.34803500	-0.45162600	-0.19284000
C	-2.96164300	0.12415700	1.04639900
N	-5.09831700	-0.38789100	1.00488700
C	-4.11740400	0.14134400	1.78516500
H	-1.98969400	0.46101700	1.35326600
H	-4.31502800	0.47954700	2.78826000
N	-4.64597200	-0.77237100	-0.18796700
C	-6.50145200	-0.53877200	1.32468700
H	-6.64335100	-0.33390100	2.38345100
H	-7.10171000	0.15589200	0.73644500
H	-6.81590000	-1.55702700	1.10361400

Cartesian coordinates of the optimized geometry for *cis*-**IV** of **1b** at B3LYP-D3BJ/def2-TZVP,aug-cc-pVTZ-PP level of theory (number of imaginary frequencies = 0):

C	0.86164400	1.74253300	1.08105100
C	0.07277400	1.22180700	0.05603900
C	-0.89273000	2.04982100	-0.53252000
C	-1.01930500	3.37901000	-0.12501000
C	-0.24430300	3.87386700	0.90971600
C	0.70176200	3.05105100	1.51405500
H	1.61256400	1.11848200	1.54566000
H	-1.74112200	4.00544600	-0.63298400
H	-0.36617100	4.89914900	1.23340700
H	1.32403900	3.42932000	2.31466700
N	-1.67480200	1.65729100	-1.65899000
N	-2.48386200	0.71412800	-1.63258700
Te	0.37271400	-0.79611700	-0.57219200
C	2.45456400	-0.78972900	-0.12936200
C	2.96195800	-1.65375300	0.83829700
C	3.32005100	0.06138400	-0.81595400
C	4.32591600	-1.66936800	1.11488500
H	2.29369100	-2.30690900	1.38395900
C	4.67771000	0.05621000	-0.52388300
H	2.93276800	0.72972400	-1.57328500
C	5.18384300	-0.81193000	0.43856100
H	4.71336100	-2.34523200	1.86673700
H	5.34293600	0.72445700	-1.05589600
H	6.24345800	-0.81883200	0.65907300
C	-2.83267000	0.03979800	-0.44305900
C	-2.90437500	0.44608100	0.91507200
N	-3.63351500	-1.60661400	0.62849500
C	-3.42619600	-0.64355500	1.56562800
H	-2.61797900	1.38528900	1.34845100
H	-3.66089200	-0.80619500	2.60383100
N	-3.29930500	-1.20437300	-0.59633600
C	-4.10022800	-2.96041600	0.83728300
H	-4.52290400	-3.03605000	1.83666800
H	-4.86282700	-3.19532400	0.09768100
H	-3.27416400	-3.66513700	0.73715900

Cartesian coordinates of the optimized geometry for *trans*-**I** of **2b** at B3LYP-D3BJ/def2-TZVP,aug-cc-pVTZ-PP level of theory (number of imaginary frequencies = 0):

C	-1.60091900	2.28363100	0.06484200
C	-0.55034900	1.38453200	0.06220200
C	0.77336200	1.81215400	-0.00559500
C	1.05422000	3.17713000	-0.08887700

C	0.00672100	4.08244500	-0.09561800
C	-1.31441000	3.64176100	-0.01731200
H	-2.62323000	1.94100100	0.13359600
H	2.08518300	3.49697800	-0.14291000
H	0.21525100	5.14249000	-0.15806600
H	-2.12472800	4.35894800	-0.01691600
N	1.72461500	0.77631100	0.02193700
N	2.92466900	1.11126500	-0.05674600
Te	-0.78313800	-0.72173600	0.20248700
C	-2.90909900	-0.73477500	-0.12702700
C	-3.43089000	-0.32725400	-1.35040800
C	-3.74535300	-1.18899000	0.88674000
C	-4.80567900	-0.36170000	-1.54988200
H	-2.77570900	0.00356600	-2.14265800
C	-5.11837400	-1.23218100	0.67047800
H	-3.33888600	-1.48263700	1.84344900
C	-5.64981600	-0.81617200	-0.54345300
H	-5.21342400	-0.03635500	-2.49822200
H	-5.76974700	-1.58756500	1.45831900
H	-6.71949300	-0.84648400	-0.70589600
C	3.81721000	0.05050800	-0.05180000
C	3.59729700	-1.34957400	-0.06256100
N	5.73582200	-0.83829700	-0.06213500
C	4.85879100	-1.88365400	-0.06870400
H	2.65651300	-1.86730300	-0.08385600
H	5.20263200	-2.90393100	-0.08265500
N	5.12303300	0.34175600	-0.05463700
C	7.18209800	-0.89921100	-0.04487900
H	7.48872900	-1.93803400	-0.14180700
H	7.58314700	-0.32079800	-0.87556400
H	7.56350700	-0.49248200	0.89147400
Cl	-0.34544400	-0.87800000	-2.27419600
Cl	-1.12822700	-0.38707500	2.68505400

Cartesian coordinates of the optimized geometry for *trans*-**II** of **2b** at B3LYP-D3BJ/def2-TZVP,aug-cc-pVTZ-PP level of theory (number of imaginary frequencies = 0):

C	1.83411100	2.09310900	0.23547500
C	0.57906700	1.52415400	0.11851000
C	-0.54781500	2.35076200	-0.00126300
C	-0.38541400	3.73567400	-0.03615000
C	0.87552800	4.30071200	0.06879000
C	1.98464800	3.47803500	0.21051300
H	2.70626600	1.46610900	0.34816800
H	-1.27369900	4.34439200	-0.13867400
H	0.99145900	5.37593500	0.04473200
H	2.97452000	3.90577900	0.30077600
N	-1.88307500	1.90538300	-0.08850700
N	-2.02272000	0.67583100	0.06458700
Te	0.39001500	-0.60661000	0.14479000
C	2.50146600	-0.99457200	-0.14577600
C	3.21111900	-1.62090200	0.87477700
C	3.12680800	-0.69229400	-1.35238100
C	4.55150900	-1.94054800	0.68583500
H	2.73374300	-1.83756100	1.81970200
C	4.46914700	-1.00559000	-1.52816400
H	2.57180000	-0.22183300	-2.15076000
C	5.18252500	-1.63131200	-0.51220600
H	5.10056500	-2.42730800	1.48163900
H	4.95474200	-0.76236300	-2.46460400
H	6.22700200	-1.87731400	-0.65478100
C	-3.30428600	0.16791100	-0.03715500
C	-4.54013400	0.79618800	-0.32005500
N	-4.75187000	-1.37197800	-0.01031500
C	-5.44627300	-0.23286900	-0.29351700
H	-4.71689100	1.83820400	-0.51216700

H	-6.51122300	-0.25047200	-0.45104800
N	-3.45068100	-1.14836900	0.14726800
C	-5.26964700	-2.71920300	0.10676700
H	-6.35127000	-2.68759300	-0.00033800
H	-4.84357500	-3.35060400	-0.67222100
H	-5.01039600	-3.12879900	1.08179300
Cl	0.69977700	-0.41432600	2.65104400
Cl	0.00554400	-0.46358200	-2.34495600

Cartesian coordinates of the optimized geometry for *cis*-**III** of **2b** at B3LYP-D3BJ/def2-TZVP,aug-cc-pVTZ-PP level of theory (number of imaginary frequencies = 0):

C	0.69798500	-0.72101600	-2.01444100
C	0.13792100	-0.76181400	-0.74939100
C	-1.18948300	-1.12812500	-0.55434900
C	-1.95525000	-1.52596900	-1.65051000
C	-1.39335900	-1.51163600	-2.91721700
C	-0.07729100	-1.09690900	-3.10530000
H	1.72899700	-0.42722600	-2.15016300
H	-2.97533500	-1.85052600	-1.50095400
H	-1.98461900	-1.82978100	-3.76585900
H	0.35457600	-1.09032500	-4.09711600
N	-1.61619700	-1.27001500	0.79816200
N	-2.71120000	-0.88593100	1.23897600
Te	1.23184500	-0.32269900	1.01499200
C	2.86559300	0.68702300	0.05201800
C	2.64164700	1.84992900	-0.67639000
C	4.14953300	0.17671500	0.20480200
C	3.71772900	2.49594400	-1.27240400
H	1.64517800	2.25538200	-0.76885100
C	5.22029700	0.84113500	-0.38314100
H	4.31625500	-0.73946200	0.75194600
C	5.00626000	1.99602400	-1.12432000
H	3.54475100	3.39586200	-1.84824700
H	6.22102900	0.44738900	-0.26240600
H	5.84181500	2.50695300	-1.58496300
C	-3.57360700	-0.05150800	0.50920900
C	-3.29995800	1.02888500	-0.36644200
N	-5.44725200	0.82878100	0.06255900
C	-4.53427900	1.56902200	-0.62430000
H	-2.34323700	1.38079600	-0.70380800
H	-4.82919700	2.41558600	-1.22072700
N	-4.87965800	-0.14379900	0.77631200
C	-6.88587600	0.98481000	0.07663700
H	-7.14116300	1.91856400	-0.41918200
H	-7.36303600	0.15429200	-0.44432500
H	-7.23762000	1.00918600	1.10632600
Cl	2.39625400	-2.52720700	0.71863300
Cl	-0.06809800	1.85281600	1.13842000

Cartesian coordinates of the optimized geometry for *cis*-**IV** of **2b** at B3LYP-D3BJ/def2-TZVP,aug-cc-pVTZ-PP level of theory (number of imaginary frequencies = 0):

C	0.34996400	1.17716300	1.81666500
C	-0.20925400	1.11816700	0.54751900
C	-1.25635200	1.96961700	0.19267600
C	-1.68463000	2.93032800	1.10657400
C	-1.12931500	2.98693700	2.37340300
C	-0.11389400	2.10782900	2.73524400
H	1.14154400	0.49553300	2.09273000
H	-2.46915200	3.61244300	0.80781100
H	-1.48654500	3.72425400	3.08046000
H	0.32629400	2.15264000	3.72225500
N	-1.81545800	2.02615000	-1.11944500
N	-2.50759200	1.11916000	-1.61258700
Te	0.51379000	-0.34618200	-0.81566500

C	2.49572100	-0.48454700	-0.02368100
C	2.99680400	-1.73322400	0.32114800
C	3.27101200	0.66205700	0.10019600
C	4.29937500	-1.83167400	0.79818700
H	2.37796700	-2.61536800	0.24739900
C	4.56407400	0.55257000	0.59503700
H	2.87996900	1.62489800	-0.19357500
C	5.08021000	-0.69178900	0.93959600
H	4.69702100	-2.80236600	1.06393300
H	5.16884500	1.44328000	0.70440200
H	6.09114400	-0.77189400	1.31766800
C	-2.90604400	-0.02907200	-0.90401400
C	-3.29295100	-1.22581100	-1.54468700
N	-3.49691600	-1.35388800	0.63557400
C	-3.64425500	-2.06314900	-0.51319600
H	-3.30091900	-1.42451800	-2.60127600
H	-3.97810100	-3.08590500	-0.50267500
N	-3.04110800	-0.12199400	0.42472800
C	-3.66852000	-1.82677400	1.99362200
H	-4.25074800	-2.74518600	1.97493300
H	-2.69199100	-2.01704700	2.43772600
H	-4.19346200	-1.06806500	2.56973600
Cl	1.27025400	1.46140900	-2.35502400
Cl	-0.20631800	-2.16252600	0.83675600

Cartesian coordinates of the optimized geometry for *trans*-**I** of **3b** at B3LYP-D3BJ/def2-TZVP,aug-cc-pVTZ-PP level of theory (number of imaginary frequencies = 0):

C	1.49769600	1.66912000	1.73373100
C	0.45353400	1.06027400	1.06188700
C	-0.87487400	1.32500400	1.38615600
C	-1.16816900	2.20825800	2.42636900
C	-0.12763600	2.81255000	3.11143300
C	1.19849500	2.54858300	2.76815600
H	2.52373000	1.47409100	1.45790600
H	-2.20262500	2.40378400	2.67092600
H	-0.34543400	3.50058400	3.91777700
H	2.00310800	3.03352200	3.30510100
N	-1.81398800	0.64285700	0.59252400
N	-3.01988200	0.83511800	0.85224200
Te	0.69332100	-0.28144700	-0.56819500
C	2.78635300	-0.65097400	-0.25881600
C	3.24015700	-1.15642600	0.95480100
C	3.66801500	-0.42176900	-1.30884200
C	4.59487500	-1.41648300	1.11988800
H	2.54835700	-1.35905800	1.75929700
C	5.01945300	-0.70043900	-1.13784000
H	3.31668100	-0.00611800	-2.24238000
C	5.48416000	-1.19271200	0.07502200
H	4.95109900	-1.80185300	2.06641600
H	5.70672600	-0.52430900	-1.95499400
H	6.53770200	-1.40290000	0.20642100
C	-3.89753500	0.10403200	0.06768000
C	-3.65971500	-0.92161900	-0.88131100
N	-5.80188100	-0.49284900	-0.63006800
C	-4.91259500	-1.27696600	-1.30619000
H	-2.71518200	-1.34410900	-1.16959300
H	-5.24340800	-2.01665800	-2.01525500
N	-5.20558600	0.34771900	0.20978200
C	-7.24279000	-0.47217900	-0.76869100
H	-7.55062700	-1.33621200	-1.35304400
H	-7.70109900	-0.51503000	0.21739100
H	-7.56171200	0.44054900	-1.27262600
Br	0.02610800	-2.35048900	1.03538800
Br	1.23711500	1.88333100	-2.10622100

Cartesian coordinates of the optimized geometry for *trans*-**II** of **3b** at B3LYP-D3BJ/def2-TZVP,aug-cc-pVTZ-PP level of theory (number of imaginary frequencies = 0):

C	1.79820100	2.17640900	0.12874500
C	0.54250200	1.60125600	0.05629100
C	-0.59289600	2.42213200	-0.02078200
C	-0.43895100	3.80771900	-0.06166800
C	0.82218700	4.37890600	-0.00474200
C	1.94001200	3.56186200	0.09709500
H	2.67690100	1.55477900	0.21479300
H	-1.33365600	4.41179700	-0.12974900
H	0.93184400	5.45463400	-0.03331100
H	2.93016900	3.99474900	0.15266600
N	-1.92647200	1.96758600	-0.05400300
N	-2.04716500	0.73468100	0.08962400
Te	0.35253600	-0.52919300	0.09996600
C	2.45857200	-0.92972100	-0.21276500
C	3.14479800	-1.64849100	0.76191800
C	3.09939500	-0.56051000	-1.39244700
C	4.47637100	-1.99261000	0.55547400
H	2.65774300	-1.92011800	1.68787400
C	4.43315800	-0.89823700	-1.58573800
H	2.56333300	-0.02105400	-2.15988200
C	5.12252100	-1.61584900	-0.61481700
H	5.00676400	-2.55086000	1.31613900
H	4.93015800	-0.60367500	-2.50116800
H	6.16029900	-1.88093300	-0.77110300
C	-3.32440400	0.21005400	0.04171000
C	-4.58122100	0.82267700	-0.17423600
N	-4.74535800	-1.35252500	0.11584300
C	-5.46947100	-0.22082400	-0.11902900
H	-4.78255800	1.86415200	-0.34380600
H	-6.54026300	-0.25308800	-0.22650900
N	-3.44202700	-1.11064200	0.21506000
C	-5.23660400	-2.70924000	0.23892300
H	-6.32238100	-2.69335000	0.18267900
H	-4.83764000	-3.32362500	-0.56750200
H	-4.92574300	-3.12672500	1.19531100
Br	0.66392200	-0.30574300	2.78730300
Br	-0.13491300	-0.42270100	-2.55712800

Cartesian coordinates of the optimized geometry for *cis*-**III** of **3b** at B3LYP-D3BJ/def2-TZVP,aug-cc-pVTZ-PP level of theory (number of imaginary frequencies = 0):

C	-0.56420000	-0.75528400	2.13188200
C	-0.02206000	-0.73840200	0.85861500
C	1.27283600	-1.18419000	0.61306200
C	2.01839500	-1.72211800	1.66233800
C	1.47350500	-1.76385600	2.93581300
C	0.19429000	-1.26889500	3.17734000
H	-1.57120500	-0.40517800	2.30673100
H	3.00866400	-2.11055500	1.47143100
H	2.04817400	-2.19048600	3.74739000
H	-0.22520900	-1.30881300	4.17365400
N	1.66200600	-1.25244000	-0.75559300
N	2.78077400	-0.94424300	-1.19650400
Te	-1.09904500	-0.11963400	-0.86377300
C	-2.67450500	0.90923200	0.16953300
C	-2.38400800	1.93234600	1.06489600
C	-3.98999200	0.56419300	-0.11745600
C	-3.42668200	2.60151900	1.69341700
H	-1.36104000	2.21766900	1.26107800
C	-5.02556200	1.25267300	0.50436100
H	-4.21259700	-0.24654900	-0.79611000
C	-4.74607100	2.26607900	1.41209900
H	-3.20309200	3.39195900	2.39804000

H	-6.05043100	0.98788900	0.27968800
H	-5.55483900	2.79544400	1.89888700
C	3.74210900	-0.26327500	-0.43409900
C	3.60963500	0.74676500	0.55208900
N	5.70783800	0.39992000	-0.00606000
C	4.89870100	1.14768100	0.79409800
H	2.70717400	1.14761300	0.97348100
H	5.29695400	1.90262600	1.45044700
N	5.02191200	-0.44372900	-0.77604800
C	7.15292200	0.42926800	-0.08127800
H	7.51509500	1.28520800	0.48357100
H	7.57329500	-0.48612800	0.33596000
H	7.45941000	0.52088800	-1.12153300
Br	-2.45547200	-2.44124300	-0.71887900
Br	0.40794100	2.14068300	-0.89333000

Cartesian coordinates of the optimized geometry for *cis*-**IV** of **3b** at B3LYP-D3BJ/def2-TZVP,aug-cc-pVTZ-PP level of theory (number of imaginary frequencies = 0):

C	-0.17468800	-0.11841200	2.29772000
C	0.23815300	-0.73802600	1.12531700
C	1.16175000	-1.78359100	1.16161600
C	1.60611800	-2.24989100	2.39727100
C	1.19474900	-1.63234100	3.56556200
C	0.30814300	-0.56088800	3.52031000
H	-0.86513000	0.71157200	2.25931200
H	2.29088300	-3.08691800	2.41694300
H	1.56461300	-1.99048100	4.51744600
H	-0.01710800	-0.07765500	4.43167200
N	1.55572800	-2.51647500	0.00292500
N	2.28296200	-2.04952000	-0.89064200
Te	-0.49874600	-0.01299100	-0.73442200
C	-2.36907700	0.72662800	-0.01157000
C	-2.77803900	1.99627200	-0.39745100
C	-3.17335900	-0.07961500	0.78443500
C	-4.01746800	2.46485300	0.02419700
H	-2.13552900	2.62692100	-0.99476500
C	-4.40058200	0.40714300	1.21484200
H	-2.85784400	-1.07588700	1.05720700
C	-4.82497700	1.67502200	0.83238100
H	-4.34401800	3.45162100	-0.27659900
H	-5.02740600	-0.21159200	1.84352200
H	-5.78589600	2.04610600	1.16428200
C	2.88977500	-0.78319600	-0.81810300
C	3.34630300	-0.08865500	-1.95887300
N	3.81310800	0.97964600	-0.10086900
C	3.91535000	1.05531900	-1.45314200
H	3.25730100	-0.39512000	-2.98554300
H	4.37254000	1.90159000	-1.93504600
N	3.18568300	-0.11997200	0.30714500
C	4.21379900	1.97358100	0.87322200
H	4.85497000	2.70357300	0.38492500
H	3.32879600	2.46868800	1.27118700
H	4.75693700	1.48527400	1.67964400
Br	-1.65001600	-2.36480500	-1.25513300
Br	0.61754800	2.43557300	-0.19688300

Cartesian coordinates of the optimized geometry for *trans*-**I** of **1c** at B3LYP-D3BJ/def2-TZVP,aug-cc-pVTZ-PP level of theory (number of imaginary frequencies = 0):

C	1.52221900	2.11933300	0.00003600
C	0.48366400	1.19562000	0.00005200
C	-0.84530500	1.63717100	-0.00003900
C	-1.12199300	3.00880200	-0.00015300
C	-0.08415800	3.92319100	-0.00017200
C	1.23778700	3.47903100	-0.00007700

H	2.54981800	1.78420100	0.00011100
H	-2.15429400	3.32831000	-0.00022400
H	-0.29943500	4.98385000	-0.00026200
H	2.05075200	4.19404800	-0.00008900
N	-1.82258100	0.62463500	-0.00000900
N	-3.01851100	1.02578000	-0.00007200
C	-4.02618000	0.11342400	-0.00013500
C	-5.37699400	0.46424800	-0.00025500
N	-3.95561700	-1.27905300	-0.00008700
C	-6.12779800	-0.71638700	-0.00032000
H	-5.73247600	1.48053400	-0.00029800
C	-5.22395700	-1.76668300	-0.00025400
H	-7.20014900	-0.81065700	-0.00043700
H	-5.39673500	-2.83021400	-0.00024600
Te	0.77665700	-0.90886200	0.00020400
C	2.90561100	-0.78589600	0.00006200
C	3.60697500	-0.74420800	1.20417100
C	3.60683000	-0.74447700	-1.20414100
C	4.99484700	-0.65621500	1.20290200
H	3.06861100	-0.77351300	2.14214200
C	4.99470300	-0.65648200	-1.20305800
H	3.06835300	-0.77399200	-2.14204000
C	5.68968100	-0.61189000	-0.00012500
H	5.53193000	-0.62173800	2.14232600
H	5.53167300	-0.62221500	-2.14255400
H	6.76994800	-0.54342500	-0.00019700
C	-2.76750500	-2.11459100	0.00013300
H	-2.15937700	-1.92167200	-0.88108300
H	-2.15955600	-1.92146500	0.88142400
H	-3.08883100	-3.15469000	0.00022200

Cartesian coordinates of the optimized geometry for *trans*-**II** of **1c** at B3LYP-D3BJ/def2-TZVP,aug-cc-pVTZ-PP level of theory (number of imaginary frequencies = 0):

C	1.64938600	2.08790000	-0.00057600
C	0.44422600	1.38704000	-0.00035200
C	-0.76134000	2.11933900	-0.00005300
C	-0.71881300	3.52279100	0.00008900
C	0.48269700	4.20080300	-0.00014300
C	1.67239600	3.47514500	-0.00048200
H	2.58448400	1.54736400	-0.00081100
H	-1.66563000	4.04614800	0.00036600
H	0.49922900	5.28249500	-0.00005400
H	2.62508300	3.98936300	-0.00066100
N	-2.04867900	1.58990300	0.00019600
N	-2.14486200	0.32901200	-0.00046100
C	-3.43265500	-0.14901200	0.00012900
C	-4.67102000	0.49124200	0.00170300
N	-3.66161400	-1.51363600	-0.00087700
C	-5.65507500	-0.50683800	0.00163800
H	-4.80665300	1.55801900	0.00274700
C	-5.00392700	-1.73045700	0.00003500
H	-6.72268400	-0.36514900	0.00262800
H	-5.39730700	-2.73264400	-0.00053800
Te	0.42635900	-0.72672700	-0.00040200
C	2.56787600	-0.91307600	0.00038000
C	3.26829700	-0.99219100	1.20350300
C	3.26910700	-0.99270800	-1.20223300
C	4.65104700	-1.14115500	1.20415100
H	2.73413500	-0.92953700	2.14303700
C	4.65186000	-1.14169100	-1.20188600
H	2.73558300	-0.93043600	-2.14215600
C	5.34407100	-1.21552700	0.00138100
H	5.18621300	-1.19853300	2.14368900
H	5.18765900	-1.19947700	-2.14103800
H	6.42031600	-1.33149500	0.00177000

C	-2.65254200	-2.55316900	-0.00249700
H	-2.02198800	-2.47665300	-0.88760800
H	-2.02187700	-2.47938500	0.88278300
H	-3.15469400	-3.51783700	-0.00396000

Cartesian coordinates of the optimized geometry for *cis*-**III** of **1c** at B3LYP-D3BJ/def2-TZVP,aug-cc-pVTZ-PP level of theory (number of imaginary frequencies = 0):

C	0.93959200	2.00673500	0.32195400
C	0.17800500	1.11298000	-0.42417700
C	-1.10731300	1.47969100	-0.82413300
C	-1.60566800	2.74461800	-0.52850700
C	-0.83621500	3.63562800	0.20648300
C	0.43159300	3.26152800	0.63998200
H	1.93151100	1.73002300	0.65121800
H	-2.59822400	3.01221000	-0.86742400
H	-1.22533700	4.61787800	0.44045700
H	1.03610500	3.95174500	1.21414400
N	-1.86186200	0.57472300	-1.62941600
N	-2.73861800	-0.16626600	-1.12229100
C	-3.05218200	-0.23646400	0.21400700
C	-2.60732500	0.30378100	1.43095800
N	-4.06464200	-1.14177900	0.51740700
C	-3.36175400	-0.28601300	2.45011600
H	-1.82824700	1.03088200	1.55472400
C	-4.25221600	-1.16965100	1.85387000
H	-3.27726200	-0.09634700	3.50679800
H	-4.99988500	-1.80739400	2.29447400
Te	0.85165500	-0.82486500	-0.99248900
C	2.70367000	-0.67989400	0.04745000
C	2.78787000	-1.06500200	1.38459500
C	3.83769700	-0.19560600	-0.60227500
C	3.99494800	-0.95967900	2.06664700
H	1.90994100	-1.44069200	1.89289500
C	5.04404200	-0.09293500	0.08224900
H	3.77648000	0.10714900	-1.63895500
C	5.12354300	-0.47388300	1.41646500
H	4.05260400	-1.25814200	3.10575200
H	5.92044400	0.28583200	-0.42816100
H	6.06293000	-0.39337100	1.94817500
C	-4.79346400	-1.93549500	-0.45666000
H	-5.34135700	-1.29245400	-1.14320800
H	-4.10672400	-2.54954000	-1.03542900
H	-5.49214800	-2.57360100	0.07992600

Cartesian coordinates of the optimized geometry for *cis*-**IV** of **1c** at B3LYP-D3BJ/def2-TZVP,aug-cc-pVTZ-PP level of theory (number of imaginary frequencies = 0):

C	0.88524100	1.96625200	0.94098600
C	0.06433800	1.22691100	0.09125200
C	-0.91678900	1.89663400	-0.65597800
C	-1.04006000	3.28470000	-0.54930300
C	-0.23501300	4.00168600	0.31884600
C	0.73823300	3.34083400	1.06237200
H	1.64864800	1.45998300	1.51616400
H	-1.77815400	3.78093400	-1.16588400
H	-0.35501500	5.07377200	0.40456700
H	1.38422000	3.89223300	1.73302000
N	-1.69146500	1.26872900	-1.66696000
N	-2.46388900	0.31173700	-1.44031500
C	-2.81825500	-0.14313500	-0.18472700
C	-2.83128000	0.36500100	1.12033300
N	-3.50157700	-1.35209800	-0.16113700
C	-3.51976100	-0.55623700	1.91994100
H	-2.40201800	1.29757600	1.43626900
C	-3.90432200	-1.61066600	1.10494600

H	-3.71510600	-0.47349300	2.97558900
H	-4.42199700	-2.52400200	1.34440400
Te	0.33353700	-0.88365300	0.00266000
C	2.45956200	-0.77549600	-0.05164500
C	3.21015400	-1.55182300	0.82811800
C	3.10814200	0.04187200	-0.97640700
C	4.60043900	-1.51696200	0.77800400
H	2.71421700	-2.17421000	1.56186700
C	4.49555800	0.08963800	-1.00829600
H	2.53157000	0.64351700	-1.66617400
C	5.24510400	-0.69327000	-0.13561400
H	5.17693600	-2.12464300	1.46403500
H	4.99245000	0.73243100	-1.72397800
H	6.32637000	-0.65890700	-0.16787300
C	-3.66309400	-2.23393100	-1.30287100
H	-4.43822700	-2.96120400	-1.07122900
H	-3.94806500	-1.65077800	-2.17454700
H	-2.73039200	-2.75390600	-1.52540000

Cartesian coordinates of the optimized geometry for *trans*-**I** of **2c** at B3LYP-D3BJ/def2-TZVP,aug-cc-pVTZ-PP level of theory (number of imaginary frequencies = 0):

C	1.36732900	2.32513600	0.07275100
C	0.34727000	1.39060900	0.06278400
C	-0.99415900	1.76551600	-0.00038300
C	-1.31103500	3.12520300	-0.07522400
C	-0.29562300	4.06596100	-0.07583000
C	1.04024800	3.67382400	0.00015200
H	2.40003000	2.01482200	0.13942500
H	-2.35045200	3.41499200	-0.12806300
H	-0.54289700	5.11820600	-0.13200800
H	1.82645200	4.41707400	0.00559800
N	-1.92298700	0.71001000	0.02026500
N	-3.13354700	1.06011300	-0.04441100
C	-4.10654900	0.11815800	-0.05268100
C	-5.46988200	0.42761700	-0.06794900
N	-3.99294400	-1.27199500	-0.05245200
C	-6.18196200	-0.77332400	-0.07193200
H	-5.85619000	1.43259000	-0.07117800
C	-5.24325800	-1.79579600	-0.06566400
H	-7.25051400	-0.90229700	-0.08150700
H	-5.38438500	-2.86398500	-0.07605100
Te	0.67876600	-0.69708600	0.19276700
C	2.80342900	-0.61988700	-0.12037000
C	3.65164400	-1.03086200	0.90170000
C	3.31415100	-0.19617100	-1.34300000
C	5.02663800	-1.01395700	0.69432100
H	3.25197000	-1.33764700	1.85711900
C	4.69031000	-0.17167700	-1.53353800
H	2.65042400	0.10284400	-2.14067800
C	5.54699800	-0.58238200	-0.51890700
H	5.68790200	-1.33507400	1.48857700
H	5.08950100	0.16592900	-2.48118700
H	6.61798700	-0.56627800	-0.67441700
Cl	0.25847300	-0.88303000	-2.29629400
Cl	0.97082400	-0.40403100	2.68415400
C	-2.77805600	-2.07098400	-0.06823800
H	-2.15579900	-1.80529700	-0.91991000
H	-2.20987300	-1.91850000	0.84742700
H	-3.06665400	-3.11766500	-0.14243000

Cartesian coordinates of the optimized geometry for *trans*-**II** of **2c** at B3LYP-D3BJ/def2-TZVP,aug-cc-pVTZ-PP level of theory (number of imaginary frequencies = 0):

C	1.54939300	2.13847500	-0.57423400
C	0.35869300	1.49358300	-0.28693400

C	-0.83340300	2.22555200	-0.17766600
C	-0.78158000	3.61651300	-0.30637100
C	0.41218500	4.26111700	-0.58389900
C	1.57899700	3.52146700	-0.72832700
H	2.46412900	1.57282800	-0.67398300
H	-1.70850100	4.16326300	-0.19785700
H	0.43196900	5.33758500	-0.69033600
H	2.51704600	4.01304700	-0.94941700
N	-2.11392900	1.70303700	0.05150300
N	-2.22748100	0.46087800	-0.12606200
C	-3.45232600	-0.08219500	0.12206800
C	-4.61954200	0.43115700	0.69138300
N	-3.68914100	-1.40469600	-0.21732500
C	-5.56335600	-0.59969200	0.69660000
H	-4.72963200	1.43665000	1.05727300
C	-4.96150700	-1.71599500	0.12683900
H	-6.57276100	-0.55454400	1.06879300
H	-5.35575600	-2.70066500	-0.05897000
Te	0.37452300	-0.60969300	0.02650200
C	2.51656300	-0.78742800	0.22899300
C	3.16695200	-0.25280300	1.33729900
C	3.22772000	-1.49753000	-0.73334100
C	4.53965200	-0.42031700	1.47144900
H	2.61020700	0.28399800	2.09146400
C	4.60005700	-1.66779000	-0.58416800
H	2.72571600	-1.89525800	-1.60341800
C	5.25715000	-1.12845400	0.51414600
H	5.04598000	0.00214300	2.32979000
H	5.15262600	-2.21967400	-1.33354800
H	6.32586500	-1.25945600	0.62490500
Cl	0.49535500	-0.88183500	-2.49636300
Cl	0.08385000	-0.14485200	2.48230200
C	-2.74354000	-2.31544400	-0.83997600
H	-2.17020700	-1.80052700	-1.60700800
H	-2.05806800	-2.72705800	-0.09841000
H	-3.29907600	-3.13041800	-1.29834000

Cartesian coordinates of the optimized geometry for *cis*-**III** of **2c** at B3LYP-D3BJ/def2-TZVP,aug-cc-pVTZ-PP level of theory (number of imaginary frequencies = 0):

C	0.84226700	1.41487600	1.71485100
C	0.13973000	1.06664500	0.57254400
C	-1.19325600	1.42375200	0.39829500
C	-1.82031100	2.19306600	1.37981400
C	-1.12059200	2.55692100	2.51901400
C	0.20351700	2.16121200	2.69696100
H	1.87781900	1.12836800	1.83078200
H	-2.84842800	2.49817600	1.23946100
H	-1.60887300	3.15870100	3.27456000
H	0.74461100	2.45252100	3.58713700
N	-1.78572400	1.15026800	-0.86207500
N	-2.84797300	0.49748200	-1.00639000
C	-3.51076400	-0.18623200	-0.02918200
C	-3.25699700	-0.62659500	1.27948200
N	-4.71491200	-0.76401700	-0.42671900
C	-4.31673600	-1.45196900	1.65575600
H	-2.38515400	-0.39814400	1.86194600
C	-5.20393200	-1.50828100	0.58490100
H	-4.43231700	-1.96762300	2.59390900
H	-6.14262400	-2.02719700	0.48746500
Te	1.01796400	0.02964700	-1.05434000
C	2.70895800	-0.73848700	0.02463900
C	2.51899600	-1.58647800	1.10994900
C	3.98775400	-0.40038800	-0.40180900
C	3.62672600	-2.08554700	1.78392100
H	1.52288900	-1.86567500	1.41966600

C	5.08881500	-0.91918000	0.27086000
H	4.12974400	0.27853700	-1.22976800
C	4.91056500	-1.75659300	1.36441400
H	3.48158400	-2.73765600	2.63545100
H	6.08551700	-0.66000500	-0.06166900
H	5.77015000	-2.15337200	1.88896100
Cl	2.29945200	2.13215300	-1.58181900
Cl	-0.34172600	-2.00349700	-0.40229800
C	-5.34656000	-0.56669200	-1.71939800
H	-5.77671000	0.43226800	-1.79398500
H	-4.60876200	-0.68537400	-2.50843600
H	-6.13369900	-1.30863000	-1.83359500

Cartesian coordinates of the optimized geometry for *cis*-**IV** of **2c** at B3LYP-D3BJ/def2-TZVP,aug-cc-pVTZ-PP level of theory (number of imaginary frequencies = 0):

N	-2.70545300	0.33186300	1.61647800
C	-1.35463900	1.92144200	0.46140400
C	-0.25477900	1.28154800	-0.12788700
C	-1.61344500	3.25266600	0.10523400
C	0.51888500	1.94874100	-1.07463900
C	-0.85849800	3.90096500	-0.85185200
H	-2.43056800	3.75312100	0.60781800
C	0.21688200	3.24799000	-1.44831000
H	1.35507000	1.44429100	-1.53593200
H	-1.09585600	4.92141100	-1.12294900
H	0.82594600	3.74869700	-2.18858900
N	-2.13923200	1.44141100	1.53656200
Te	0.32786700	-0.72939700	0.30383100
C	2.43007400	-0.42246300	0.03641600
C	3.08665700	0.57017100	0.75400400
C	3.12058300	-1.25837500	-0.83187100
C	4.45494500	0.73470300	0.58320000
H	2.54455000	1.20004300	1.44383200
C	4.49370500	-1.09523100	-0.98113800
H	2.59806600	-2.00854900	-1.40755600
C	5.15923200	-0.09863000	-0.27918300
H	6.22669900	0.02976300	-0.40315400
H	5.03760200	-1.74627200	-1.65294200
H	4.96999900	1.51301100	1.13100900
C	-2.86632700	-0.63296300	0.64441800
C	-3.00725500	-1.97756000	0.98925100
C	-3.12223800	0.63729800	-1.57839400
H	-2.15888500	0.98416800	-1.94622000
H	-3.75794000	0.38453800	-2.42423500
H	-3.60035200	1.42960000	-1.00983300
Cl	0.13612700	-1.19696200	-2.20710200
Cl	0.56824600	-0.16384600	2.71455600
N	-2.97197300	-0.54869000	-0.74931900
C	-3.14287100	-2.71582300	-0.19336000
C	-3.14874800	-1.80765000	-1.23489400
H	-2.96747300	-2.33720200	2.00324900
H	-3.24415300	-3.78306000	-0.28946400
H	-3.28251900	-1.96423400	-2.29143500

Cartesian coordinates of the optimized geometry for *trans*-**I** of **3c** at B3LYP-D3BJ/def2-TZVP,aug-cc-pVTZ-PP level of theory (number of imaginary frequencies = 0):

C	1.27756600	-1.61600900	1.83805400
C	0.26813200	-1.00084000	1.11956600
C	-1.07903600	-1.21663800	1.40770900
C	-1.41383600	-2.06297300	2.46851700
C	-0.40956500	-2.67328600	3.20026500
C	0.93230900	-2.45733300	2.88889400
H	2.31512200	-1.45269900	1.58585800
H	-2.45766600	-2.22767900	2.69316900

H	-0.67035600	-3.33028200	4.01988800
H	1.70955600	-2.94657500	3.46076400
N	-1.99179000	-0.54948500	0.57255900
N	-3.20852700	-0.73176400	0.85348300
C	-4.16950700	-0.12798700	0.11607200
C	-5.53664300	-0.30482700	0.35163800
N	-4.04089900	0.74414800	-0.96511300
C	-6.23531300	0.45686100	-0.58635400
H	-5.93381900	-0.93152200	1.13184100
C	-5.28517700	1.08876900	-1.37720800
H	-7.30236700	0.55099400	-0.69140500
H	-5.41487400	1.76549100	-2.20563300
Te	0.60824400	0.29163200	-0.52397700
C	2.72980100	0.48344600	-0.24879100
C	3.57834000	0.09623200	-1.27978400
C	3.23860100	1.03176700	0.92421500
C	4.95126100	0.25785700	-1.13002900
H	3.18246300	-0.34945500	-2.18091500
C	4.61290500	1.17598100	1.06782800
H	2.57553900	1.35437200	1.71353000
C	5.46950000	0.79295100	0.04220700
H	5.61254100	-0.04232900	-1.93234600
H	5.01048100	1.59519600	1.98296800
H	6.53904900	0.91246800	0.15688500
C	-2.81747900	1.24410800	-1.57126000
H	-2.21347200	1.76884500	-0.83402000
H	-2.23781400	0.42402100	-1.99058300
H	-3.09498600	1.93229700	-2.36724600
Br	0.88671500	-1.90109100	-2.08800300
Br	0.10669200	2.43146900	1.06195500

Cartesian coordinates of the optimized geometry for *trans*-**II** of **3c** at B3LYP-D3BJ/def2-TZVP,aug-cc-pVTZ-PP level of theory (number of imaginary frequencies = 0):

C	1.51201100	1.88958800	-1.32438700
C	0.32226400	1.36538000	-0.84765000
C	-0.88572300	2.04964800	-1.05411600
C	-0.85106800	3.29220100	-1.69443200
C	0.34122200	3.82132700	-2.15824900
C	1.52475800	3.11528400	-1.98269500
H	2.43787100	1.35120900	-1.18628400
H	-1.79052200	3.81173300	-1.82597600
H	0.34715400	4.77984700	-2.65988700
H	2.46185000	3.51425000	-2.34746900
N	-2.16499800	1.61632500	-0.68412000
N	-2.25149200	0.40435800	-0.34903700
C	-3.48090900	-0.02879100	0.04787300
C	-4.68252300	0.63856900	0.29657700
N	-3.68670900	-1.37871800	0.28311100
C	-5.61553500	-0.32443900	0.68941700
H	-4.82160000	1.70095400	0.20228600
C	-4.97281100	-1.55718900	0.66796700
H	-6.64412100	-0.15964800	0.96194300
H	-5.34651400	-2.54113000	0.89531800
Te	0.35597000	-0.48132100	0.20498600
C	2.48673800	-0.51029000	0.54297500
C	3.10046700	0.47483800	1.31118900
C	3.22565400	-1.57636300	0.04001400
C	4.46442900	0.39634300	1.56182500
H	2.52263500	1.29192000	1.71834900
C	4.58877200	-1.65186100	0.30569500
H	2.75425800	-2.33063400	-0.57394000
C	5.20916200	-0.66642500	1.06254400
H	4.94227800	1.16624900	2.15376600
H	5.16295100	-2.48105800	-0.08683300
H	6.27096400	-0.72559800	1.26394400

C	-2.70121700	-2.43702200	0.14648900
H	-2.12298100	-2.30250500	-0.76493300
H	-2.02648500	-2.44793300	1.00293500
H	-3.22343500	-3.38962800	0.09664800
Br	-0.11689100	0.86569400	2.49044700
Br	0.56756200	-1.73563300	-2.20259100

Cartesian coordinates of the optimized geometry for *cis*-**III** of **3c** at B3LYP-D3BJ/def2-TZVP,aug-cc-pVTZ-PP level of theory (number of imaginary frequencies = 0):

C	-0.74038000	-0.86554000	2.17087500
C	-0.06587000	-0.83515300	0.96137400
C	1.22386200	-1.34099700	0.82977400
C	1.83065500	-1.93709200	1.93644300
C	1.15804800	-1.98311700	3.14703500
C	-0.11864600	-1.43921700	3.27270900
H	-1.74390900	-0.47322800	2.25096300
H	2.82103400	-2.35994800	1.83663600
H	1.62943800	-2.45265900	4.00065000
H	-0.63955600	-1.48445200	4.21960700
N	1.76155200	-1.40148700	-0.48120900
N	2.88098200	-0.93597500	-0.80664800
C	3.68934100	-0.15915400	-0.03095900
C	3.59283000	0.56168500	1.17050300
N	4.91203700	0.17709600	-0.60935400
C	4.76153800	1.31051700	1.30414100
H	2.75365900	0.55979900	1.83936600
C	5.55849700	1.04390400	0.19418100
H	5.00885900	1.98447200	2.10661500
H	6.53541600	1.41630000	-0.06435600
Te	-0.92530400	-0.12247700	-0.84205100
C	-2.52484000	0.99043700	0.05680800
C	-2.25496700	1.96554000	1.01028900
C	-3.82524400	0.74994700	-0.37005700
C	-3.30607400	2.69293200	1.55425100
H	-1.23946800	2.16907200	1.31666300
C	-4.86754200	1.49608400	0.16868000
H	-4.03388400	-0.02539600	-1.09312400
C	-4.61061300	2.46254100	1.13254900
H	-3.09998800	3.44600600	2.30373600
H	-5.88064900	1.31274900	-0.16452900
H	-5.42525000	3.03683200	1.55434900
C	5.40529000	-0.34172500	-1.87309600
H	5.68422000	-1.39118100	-1.77973800
H	4.63177600	-0.25548000	-2.63177800
H	6.27673200	0.23984100	-2.16518900
Br	-2.45061700	-2.34971100	-0.88915300
Br	0.69404700	2.05082700	-0.71872500

Cartesian coordinates of the optimized geometry for *cis*-**IV** of **3c** at B3LYP-D3BJ/def2-TZVP,aug-cc-pVTZ-PP level of theory (number of imaginary frequencies = 0):

N	-2.65881700	-1.70896200	-0.50168500
C	-1.39712900	-1.43404400	1.50228900
C	-0.32430600	-0.55782900	1.28081900
C	-1.69004200	-1.79834900	2.82451100
C	0.38683700	-0.03490400	2.35902300
C	-0.99777800	-1.25856900	3.88980700
H	-2.48284800	-2.51836000	2.97849500
C	0.04953300	-0.37014300	3.65965900
H	1.20030500	0.65306600	2.18282500
H	-1.26165600	-1.54095500	4.90060800
H	0.60917300	0.05048400	4.48394800
N	-2.10962000	-2.16366100	0.52304900
Te	0.29946700	0.09734800	-0.65784700
C	2.38233100	0.25266100	-0.19292800
C	3.03967800	-0.79383700	0.44232200

C	3.06362200	1.39582800	-0.58962100
C	4.39968100	-0.68088500	0.69799900
H	2.50632500	-1.68942600	0.72551600
C	4.42933800	1.48972000	-0.34476600
H	2.54046200	2.21724200	-1.05806000
C	5.09527800	0.45648300	0.30161200
H	6.15672100	0.53606600	0.49712500
H	4.96648700	2.37642400	-0.65470700
H	4.91567900	-1.48801800	1.20131300
C	-2.87614500	-0.39560900	-0.85866200
C	-2.98603100	-0.02703400	-2.20017500
C	-3.29683300	0.86786000	1.34220800
H	-2.36152400	1.05619100	1.86527900
H	-3.97939200	1.69507200	1.52397600
H	-3.74796400	-0.04451800	1.72162000
N	-3.08419300	0.75809700	-0.09266300
C	-3.20655100	1.35496100	-2.25235800
C	-3.29364700	1.79739800	-0.94588700
H	-2.87094800	-0.71914600	-3.01681400
H	-3.31008300	1.96747700	-3.13130300
H	-3.50542600	2.77901500	-0.55855200
Br	-0.02947000	2.68701700	0.20844900
Br	0.69672200	-2.40625400	-1.47842800

Cartesian coordinates of the optimized geometry for *trans*-**I** of **1d** at B3LYP-D3BJ/def2-TZVP,aug-cc-pVTZ-PP level of theory (number of imaginary frequencies = 0):

C	-0.59881600	2.99212900	-0.00125000
C	0.43440600	3.91189400	0.00006100
C	1.75821000	3.47248500	0.00117900
C	2.04911300	2.11451200	0.00098200
C	1.01477400	1.18488600	-0.00033700
C	-0.31479400	1.62291200	-0.00146400
H	-1.63245800	3.30723000	-0.00220100
H	0.21505600	4.97175300	0.00020600
H	2.56798000	4.19123400	0.00221200
H	3.07827600	1.78429700	0.00183700
N	-1.28436200	0.60017700	-0.00288800
N	-2.48226300	0.97792000	-0.00409800
C	-3.44951500	0.00270600	-0.00418100
C	-4.80834500	0.33043800	-0.00789600
C	-3.40315200	-1.43002900	-0.00599800
N	-5.46865500	-0.83939200	-0.01771700
N	-4.63014100	-1.91691300	-0.01080400
C	-2.22550000	-2.33570000	-0.00903600
H	-1.59776700	-2.15604400	-0.88346100
H	-1.59685600	-2.16011300	0.86549800
H	-2.56127800	-3.37170600	-0.01130800
C	-6.89578000	-1.05611000	0.03596300
H	-7.27259700	-0.94971000	1.05582900
H	-7.40868000	-0.34380900	-0.60905000
H	-7.08894800	-2.06692600	-0.31154900
C	-5.46510800	1.66157100	-0.00085900
H	-6.10415100	1.78976400	0.87624700
H	-4.69890800	2.43322700	0.01296700
H	-6.08669600	1.80713700	-0.88802800
Te	1.30852500	-0.91985800	-0.00091900
C	3.43866300	-0.78139200	0.00172400
C	4.13914500	-0.73596800	1.20633300
C	4.14208000	-0.73555300	-1.20115500
C	5.52649800	-0.63934900	1.20716900
H	3.59984800	-0.76958700	2.14370300
C	5.52943400	-0.63895400	-1.19857200
H	3.60506600	-0.76883100	-2.13984700
C	6.22277900	-0.59023900	0.00515300
H	6.06216300	-0.60232800	2.14736900

H	6.06739200	-0.60162400	-2.13745000
H	7.30265900	-0.51536600	0.00648400

Cartesian coordinates of the optimized geometry for *trans*-**II** of **1d** at B3LYP-D3BJ/def2-TZVP,aug-cc-pVTZ-PP level of theory (number of imaginary frequencies = 0):

C	0.06875600	3.60403100	-0.17997800
C	-1.17318900	4.20002900	-0.27022700
C	-2.31136800	3.39686800	-0.30934900
C	-2.19995200	2.01485800	-0.25636700
C	-0.95324400	1.39706100	-0.16623300
C	0.19774200	2.20899000	-0.13658000
H	0.97754500	4.19019200	-0.14459100
H	-1.26032000	5.27767400	-0.30819700
H	-3.29355200	3.84711000	-0.37808500
H	-3.09676400	1.41319900	-0.28102500
N	1.51410500	1.74710900	-0.04583300
N	1.65838900	0.49793200	-0.13209300
C	2.94669700	0.01728400	-0.00799500
C	3.27959700	-1.32630300	-0.19366600
C	4.17293900	0.67319000	0.33050200
N	4.60440800	-1.40768700	0.03178400
N	5.15947100	-0.20500200	0.35119100
C	4.42640700	2.10431500	0.64821800
H	4.18777600	2.74141700	-0.20413800
H	3.79144400	2.43883200	1.46964600
H	5.47348200	2.23438600	0.91908700
C	5.45301700	-2.57537500	-0.04838100
H	5.06867400	-3.37749200	0.58227200
H	5.53200000	-2.93508200	-1.07618300
H	6.43656100	-2.27871900	0.30281700
C	2.43644700	-2.49316000	-0.55956900
H	2.00964100	-2.96841800	0.32806000
H	1.60732500	-2.17443100	-1.18871000
H	3.01055100	-3.24560000	-1.10054900
Te	-0.78626000	-0.70964000	-0.05834000
C	-2.91095800	-1.02298900	0.11353200
C	-3.66779000	-1.34920300	-1.01093700
C	-3.54057400	-0.93925100	1.35521700
C	-5.03478100	-1.58241800	-0.89747300
H	-3.19104600	-1.41361000	-1.98094600
C	-4.90710500	-1.16846700	1.46901200
H	-2.96326700	-0.68688400	2.23570300
C	-5.65582800	-1.49070100	0.34236100
H	-5.61370100	-1.83267600	-1.77770300
H	-5.38674700	-1.09711100	2.43722100
H	-6.71959400	-1.67052300	0.43113100

Cartesian coordinates of the optimized geometry for *cis*-**III** of **1d** at B3LYP-D3BJ/def2-TZVP,aug-cc-pVTZ-PP level of theory (number of imaginary frequencies = 0):

C	-1.14310300	1.17723200	1.46267000
C	-0.56274300	0.04857400	0.89428800
C	0.77448600	-0.25387500	1.16495000
C	1.50317800	0.53180000	2.05925700
C	0.90848300	1.63558000	2.64942200
C	-0.40914800	1.96636800	2.33979500
H	-2.16945400	1.43192000	1.23827000
H	2.52654600	0.27037000	2.28935900
H	1.47038200	2.23767400	3.35133800
H	-0.87273500	2.83191000	2.79537700
N	1.26186900	-1.47715100	0.62610900
N	2.39537400	-1.62329100	0.12449200
C	3.25841100	-0.56597500	-0.17601600
C	3.02085400	0.67001200	-0.78386000
Te	-1.56636900	-1.27497100	-0.43003800

C	-3.38548700	-0.16257500	-0.42372200
C	-3.60807700	0.82915300	-1.37766400
C	-4.36017800	-0.42143000	0.53868700
C	-4.79161800	1.55912800	-1.36434000
H	-2.85565200	1.03398800	-2.12754600
C	-5.54240500	0.31084300	0.55063300
H	-4.19327000	-1.18993400	1.28160300
C	-5.75894900	1.30140800	-0.40001800
H	-4.95679600	2.32881700	-2.10760800
H	-6.29376000	0.10542900	1.30250200
H	-6.68004000	1.86981700	-0.39062100
C	5.44800200	-1.81389500	0.49156300
H	5.37925600	-1.83188600	1.58193300
H	5.05462700	-2.76480100	0.12895800
H	6.49721800	-1.72396200	0.21517500
C	4.67151000	-0.68181400	-0.08285100
C	1.76869900	1.30974400	-1.26333700
H	1.02657200	0.55247200	-1.51246000
H	1.32757600	1.95231200	-0.49717300
H	1.94941200	1.91069100	-2.15488000
N	4.24437400	1.21146500	-0.97470300
N	5.25527100	0.41164500	-0.54371800
C	4.56758600	2.44944200	-1.64853800
H	4.42589600	2.36068400	-2.72803800
H	3.94809100	3.26271800	-1.27199400
H	5.61210000	2.66327400	-1.44302700

Cartesian coordinates of the optimized geometry for *cis*-**IV** of **1d** at B3LYP-D3BJ/def2-TZVP,aug-cc-pVTZ-PP level of theory (number of imaginary frequencies = 0):

N	2.17008500	0.75004100	-1.67749800
C	0.50255100	2.12245800	-0.73395800
C	-0.36861400	1.28632600	-0.01738200
C	0.42595500	3.50883100	-0.56012900
C	-1.24857500	1.86376700	0.89984100
C	-0.43823800	4.06385500	0.36381700
H	1.07133100	4.13071000	-1.16669600
C	-1.28004600	3.23491700	1.10127800
H	-1.92297200	1.23146800	1.46052900
H	-0.46617700	5.13662000	0.50255400
H	-1.96818900	3.65431400	1.82351700
N	1.34969300	1.68311400	-1.78293000
Te	-0.46706200	-0.82179400	-0.31201700
C	-2.57805300	-0.91801000	-0.03607100
C	-3.43573100	-0.23124600	-0.89477700
C	-3.10824700	-1.68906000	0.99559800
C	-4.81019300	-0.30653400	-0.71182500
H	-3.03003700	0.36582800	-1.70060700
C	-4.48706600	-1.77709400	1.16444300
H	-2.44784900	-2.21123300	1.67568700
C	-5.33865500	-1.08307700	0.31489100
H	-6.41057500	-1.14496600	0.45131600
H	-4.89210200	-2.37935400	1.96782900
H	-5.47016500	0.23502900	-1.37765500
C	2.54393700	0.12973900	-0.47765500
C	2.99557600	-1.19325400	-0.51075000
C	2.77772700	0.57228200	0.86715600
N	3.40291900	-1.47965900	0.73802800
N	3.29890700	-0.41592300	1.57610500
C	3.83447500	-2.75419400	1.26394900
H	4.36466200	-2.56348100	2.19252000
H	2.97935400	-3.40345900	1.46390700
H	4.50025000	-3.24805200	0.55771700
C	3.00305100	-2.15054500	-1.64379700
H	3.94786100	-2.69386200	-1.70379100
H	2.19845600	-2.88572400	-1.55461000

H	2.85062900	-1.59857600	-2.56932200
C	2.62251200	1.91519600	1.49196300
H	2.98620900	2.70185200	0.83096300
H	1.57886000	2.13630100	1.71610900
H	3.19226300	1.94072300	2.41941600

Cartesian coordinates of the optimized geometry for *trans*-**I** of **2d** at B3LYP-D3BJ/def2-TZVP,aug-cc-pVTZ-PP level of theory (number of imaginary frequencies = 0):

C	-1.60091900	328363100	0.06484200
C	-0.55034900	1.38453200	0.06220200
C	0.77336200	1.81215400	-0.00559500
C	1.05422000	3.17713000	-0.08887700
C	0.00672100	4.08244500	-0.09561800
C	-1.31441000	3.64176100	-0.01731200
H	-2.62323000	1.94100100	0.13359600
H	2.08518300	3.49697800	-0.14291000
H	0.21525100	5.14249000	-0.15806600
H	-2.12472800	4.35894800	-0.01691600
N	1.72461500	0.77631100	0.02193700
N	2.92466900	1.11126500	-0.05674600
Te	-0.78313800	-0.72173600	0.20248700
C	-2.90909900	-0.73477500	-0.12702700
C	-3.43089000	-0.32725400	-1.35040800
C	-3.74535300	-1.18899000	0.88674000
C	-4.80567900	-0.36170000	-1.54988200
H	-2.77570900	0.00356600	-2.14265800
C	-5.11837400	-1.23218100	0.67047800
H	-3.33888600	-1.48263700	1.84344900
C	-5.64981600	-0.81617200	-0.54345300
H	-5.21342400	-0.03635500	-2.49822200
H	-5.76974700	-1.58756500	1.45831900
H	-6.71949300	-0.84648400	-0.70589600
C	3.81721000	0.05050800	-0.05180000
C	3.59729700	-1.34957400	-0.06256100
N	5.73582200	-0.83829700	-0.06213500
C	4.85879100	-1.88365400	-0.06870400
H	2.65651300	-1.86730300	-0.08385600
H	5.20263200	-2.90393100	-0.08265500
N	5.12303300	0.34175600	-0.05463700
C	7.18209800	-0.89921100	-0.04487900
H	7.48872900	-1.93803400	-0.14180700
H	7.58314700	-0.32079800	-0.87556400
H	7.56350700	-0.49248200	0.89147400
Cl	-0.34544400	-0.87800000	-2.27419600
Cl	-1.12822700	-0.38707500	2.68505400

Cartesian coordinates of the optimized geometry for *trans*-**II** of **2d** at B3LYP-D3BJ/def2-TZVP,aug-cc-pVTZ-PP level of theory (number of imaginary frequencies = 0):

C	0.13766000	3.67770600	-0.61371700
C	-1.10901200	4.21120500	-0.89870900
C	-2.21868400	3.37815100	-0.96494400
C	-2.08107700	2.01351100	-0.72802600
C	-0.83748900	1.48108100	-0.43447200
C	0.29391400	2.30701900	-0.40367900
H	1.02140800	4.29950400	-0.56343300
H	-1.21448000	5.27413000	-1.07070500
H	-3.19630900	3.78424000	-1.18833600
H	-2.95137800	1.37477700	-0.76709400
N	1.61333400	1.87587100	-0.16437000
N	1.78996500	0.64467700	-0.33374800
C	3.02512500	0.12227300	-0.05577800
C	3.37266800	-1.19290500	-0.38251600
C	4.16256000	0.66888500	0.62056900
N	4.61932600	-1.36045000	0.08517300

N	5.11232400	-0.24218100	0.69843100
C	4.33431100	2.01802100	1.21997500
H	4.30856000	2.79211100	0.45197200
H	3.51746900	2.23689600	1.90954600
H	5.28379500	2.06347300	1.75117300
C	5.46451900	-2.52720300	-0.03640000
H	4.89893800	-3.42911700	0.19368000
H	5.87747700	-2.60955600	-1.04404500
H	6.27718900	-2.41532200	0.67503800
C	2.61269200	-2.24238800	-1.10927800
H	2.24592900	-3.01221600	-0.42481400
H	1.75760100	-1.80580100	-1.62030900
H	3.23632100	-2.73484100	-1.85756300
Te	-0.66534100	-0.59750000	-0.00362200
C	-2.77122500	-0.90417300	0.36783200
C	-3.48520100	-1.75501000	-0.47008600
C	-3.38986600	-0.30823300	1.46281300
C	-4.82838100	-2.00565800	-0.20980600
H	-3.01019400	-2.20057300	-1.33227800
C	-4.73450000	-0.55712400	1.70842200
H	-2.82916900	0.33840000	2.12202800
C	-5.45434300	-1.40635100	0.87557800
H	-5.38334300	-2.66700300	-0.86262900
H	-5.21698500	-0.08748600	2.55594700
H	-6.50081000	-1.60016200	1.07297000
Cl	-0.95438500	-1.03072600	-2.48689700
Cl	-0.24743600	0.04074400	2.39878700

Cartesian coordinates of the optimized geometry for *cis*-**III** of **2d** at B3LYP-D3BJ/def2-TZVP,aug-cc-pVTZ-PP level of theory (number of imaginary frequencies = 0):

C	-1.61446100	0.09484900	2.18975000
C	-0.98881500	-0.52621200	3.25972300
C	0.35396800	-0.88715200	3.18738300
C	1.09646500	-0.58448600	2.05098000
C	0.47390600	0.06646600	1.00211700
C	-0.88383200	0.37647000	1.03339600
H	-2.65890600	0.36466400	2.24606200
H	-1.55464200	-0.73507200	4.15824100
H	0.83137400	-1.37765600	4.02504800
H	2.14947100	-0.82258700	2.00495600
N	-1.34101700	1.16037100	-0.05426600
N	-2.48842600	1.14247500	-0.54064600
C	-3.47377200	0.19713800	-0.28460300
C	-4.81553900	0.55186700	-0.45755900
C	-3.48377700	-1.22260700	-0.06127300
N	-5.52372700	-0.57355000	-0.27216700
N	-4.73087400	-1.66072300	-0.06756900
C	-2.36043600	-2.19364700	0.04957900
H	-1.54586400	-1.93002800	-0.62371500
H	-1.95212000	-2.22187100	1.06087100
H	-2.72462300	-3.18802900	-0.20346600
C	-6.95696000	-0.74583000	-0.35331000
H	-7.46358500	0.05315200	0.18691000
H	-7.29443800	-0.74557600	-1.39188000
H	-7.19793200	-1.70287500	0.09969400
C	-5.40620800	1.87787800	-0.76519600
H	-5.87070900	2.33085500	0.11504300
H	-4.61538200	2.54296300	-1.10803100
H	-6.16883200	1.80300500	-1.54252300
Te	1.51097100	0.81541000	-0.69380400
C	3.29203700	-0.35380000	-0.40451200
C	3.23699400	-1.74268600	-0.45031000
C	4.50071900	0.30411300	-0.20749700
C	4.40447900	-2.47634100	-0.27931300
H	2.29978700	-2.24847300	-0.62912300

C	5.66524100	-0.44021900	-0.05331400
H	4.53641000	1.38208200	-0.14834400
C	5.61855600	-1.82792800	-0.08451500
H	4.36164200	-3.55751500	-0.30514100
H	6.60722200	0.07104700	0.09665800
H	6.52624700	-2.40385200	0.04150500
Cl	2.41998700	2.60971700	0.82387300
Cl	0.50950200	-1.03929300	-2.09984600

Cartesian coordinates of the optimized geometry for *cis*-**IV** of **2d** at B3LYP-D3BJ/def2-TZVP,aug-cc-pVTZ-PP level of theory (number of imaginary frequencies = 0):

N	-2.35723000	0.82272000	1.75331900
C	-0.80269700	2.21585200	0.61146000
C	0.16585500	1.44529400	-0.04486100
C	-0.87388200	3.58221800	0.31304000
C	1.00190100	2.03085500	-0.99219000
C	-0.06084400	4.15367100	-0.64677200
H	-1.58914700	4.17635200	0.86642900
C	0.88724200	3.37605400	-1.30393700
H	1.73794700	1.42865000	-1.50413100
H	-0.15215800	5.20863600	-0.86976900
H	1.54165800	3.81205600	-2.04651200
N	-1.62357500	1.82485300	1.70862100
Te	0.44314200	-0.65109300	0.26818200
C	2.56043700	-0.64877100	-0.05024000
C	3.37623700	0.19873900	0.69002900
C	3.09682400	-1.53752000	-0.97346900
C	4.74951100	0.16279300	0.48731400
H	2.95171600	0.87069700	1.42153500
C	4.47503200	-1.57567500	-1.15624400
H	2.45414500	-2.17452700	-1.56394400
C	5.29949200	-0.72508300	-0.43117200
H	6.37102500	-0.75316200	-0.58063400
H	4.89889500	-2.26841400	-1.87138900
H	5.38914400	0.82717700	1.05352400
C	-2.61826600	-0.05489400	0.69839600
C	-2.84394100	0.11155500	-0.67871500
C	-2.90545300	-1.42678400	0.96598500
N	-3.19791200	-1.10869900	-1.12623500
N	-3.21790200	-2.05669300	-0.15083800
C	-3.51557200	-1.48763200	-2.48660100
H	-2.68491500	-1.24157500	-3.14635500
H	-3.67483600	-2.56113900	-2.48665000
H	-4.42292200	-0.98637400	-2.82761700
C	-2.82448900	1.30541400	-1.56217600
H	-1.85218900	1.41421400	-2.04805400
H	-3.58373900	1.22518700	-2.34004500
H	-3.01727200	2.20843000	-0.98946400
C	-2.81485100	-2.12168600	2.27752500
H	-1.77867700	-2.19517800	2.61509400
H	-3.35409200	-1.55965300	3.04142300
H	-3.23174200	-3.12376800	2.19551300
Cl	0.10972600	-0.95295300	-2.25689800
Cl	0.80879600	-0.27366000	2.70026800

Cartesian coordinates of the optimized geometry for *trans*-**I** of **3d** at B3LYP-D3BJ/def2-TZVP,aug-cc-pVTZ-PP level of theory (number of imaginary frequencies = 0):

C	-1.00796700	3.19475000	-0.35828900
C	-0.00468600	4.14330000	-0.46491100
C	1.33715900	3.77159400	-0.39245300
C	1.68475500	2.43712400	-0.21838300
C	0.67634000	1.49515000	-0.12432400
C	-0.66928400	1.85163700	-0.18149000
H	-2.05202800	3.46874900	-0.40305200

H	-0.26558300	5.18507300	-0.59947800
H	2.11354100	4.52153700	-0.46657300
H	2.72270200	2.14573400	-0.15139600
N	-1.57464200	0.78306600	-0.03573600
N	-2.79157700	1.08004600	-0.12205200
C	-3.70546200	0.07149000	0.01631400
C	-5.07990700	0.32731900	-0.06399400
C	-3.59219600	-1.34131500	0.24264200
N	-5.68242600	-0.85851400	0.10074200
N	-4.79350200	-1.88035900	0.29370300
C	-2.37495700	-2.17889400	0.39788700
H	-1.74636500	-2.11201700	-0.49167500
H	-1.77879900	-1.84082700	1.24726600
H	-2.66465300	-3.21655600	0.55505200
C	-7.09847000	-1.14009100	0.16443700
H	-7.48889500	-0.96046000	1.16849400
H	-7.63550400	-0.51353800	-0.54565600
H	-7.24078200	-2.18623600	-0.09120700
C	-5.79525000	1.60933900	-0.28074500
H	-6.45787900	1.84465300	0.55541700
H	-5.06583600	2.40951400	-0.38114700
H	-6.40286600	1.57926900	-1.18849800
Te	1.00055800	-0.58196800	0.15956500
C	3.13041900	-0.53006100	-0.13120700
C	3.95721700	-0.97377800	0.89505800
C	3.66732500	-0.11033800	-1.34416600
C	5.33428500	-0.99205300	0.70346100
H	3.54072400	-1.27999000	1.84387500
C	5.04556500	-0.11993900	-1.51971500
H	3.02199300	0.20873500	-2.14947800
C	5.87957700	-0.56250500	-0.49955500
H	5.97765400	-1.33861700	1.50168900
H	5.46396200	0.21355700	-2.46053300
H	6.95228100	-0.57368000	-0.64319300
Br	1.23872300	-0.07297300	2.81200000
Br	0.54886500	-0.98070100	-2.47763700

Cartesian coordinates of the optimized geometry for *trans*-**II** of **3d** at B3LYP-D3BJ/def2-TZVP,aug-cc-pVTZ-PP level of theory (number of imaginary frequencies = 0):

C	0.25640600	3.42212000	-1.65228900
C	-0.98854000	3.89252200	-2.03705900
C	-2.11724700	3.10828500	-1.83351300
C	-1.99955100	1.86125000	-1.22762700
C	-0.75714100	1.39570100	-0.83131700
C	0.39242100	2.16131800	-1.06927100
H	1.15447600	4.00499900	-1.80656100
H	-1.07818000	4.86718800	-2.49796500
H	-3.09359500	3.46405300	-2.13459300
H	-2.88302400	1.26156600	-1.06533600
N	1.70994000	1.77536700	-0.75980100
N	1.85323600	0.54420000	-0.55890600
C	3.08712700	0.09006600	-0.17674500
C	3.39777400	-1.27223800	-0.10256700
C	4.25872900	0.78130300	0.27046500
N	4.65773800	-1.32744000	0.35535100
N	5.19365400	-0.09135300	0.58988700
C	4.47664300	2.24226400	0.43397900
H	4.43917300	2.75276600	-0.52917700
H	3.68817000	2.67924300	1.04885500
H	5.44454800	2.41685100	0.90131900
C	5.47337400	-2.50166800	0.57219100
H	4.91401600	-3.25562800	1.12486100
H	5.80880400	-2.92674500	-0.37604700
H	6.33778100	-2.19208400	1.15164800
C	2.59217500	-2.47070700	-0.45086600

H	2.20508500	-2.96169300	0.44629000
H	1.74798900	-2.19233000	-1.07775400
H	3.18888600	-3.20280400	-0.99746600
Te	-0.60736900	-0.49148500	0.14378100
C	-2.69548600	-0.62162500	0.67606700
C	-3.42247500	-1.72720900	0.24673300
C	-3.28595600	0.33791700	1.49298600
C	-4.75027600	-1.86883600	0.63569200
H	-2.97159700	-2.46173500	-0.40565000
C	-4.61584200	0.19395300	1.86722000
H	-2.71494300	1.18567200	1.84333300
C	-5.34817700	-0.90900800	1.44204800
H	-5.31544700	-2.72879400	0.30038300
H	-5.07641300	0.94382900	2.49738300
H	-6.38297300	-1.01935600	1.73960700
Br	0.00448600	0.80137200	2.43321000
Br	-1.01749400	-1.66067600	-2.27784500

Cartesian coordinates of the optimized geometry for *cis*-**III** of **3d** at B3LYP-D3BJ/def2-TZVP,aug-cc-pVTZ-PP level of theory (number of imaginary frequencies = 0):

C	-1.77215700	1.18757400	1.96107200
C	-1.16244100	1.18345900	3.20623200
C	0.16333300	0.78286600	3.34883400
C	0.90780900	0.42508700	2.22990500
C	0.30220000	0.46472800	0.98794700
C	-1.04153200	0.80181900	0.83515200
H	-2.80335500	1.49139900	1.85652600
H	-1.72753400	1.49277200	4.07574100
H	0.62820900	0.77852900	4.32557600
H	1.95086500	0.16130200	2.32776000
N	-1.46304600	0.93346600	-0.50993400
N	-2.61682200	0.75474200	-0.94603800
C	-3.67142700	0.15948900	-0.26772400
C	-4.98174800	0.47231400	-0.64597900
C	-3.79660300	-0.93098400	0.66059600
N	-5.77796500	-0.33391500	0.07322100
N	-5.07540200	-1.21221100	0.84003800
C	-2.75664700	-1.78393000	1.29893000
H	-1.92634900	-1.96615200	0.61771600
H	-2.34700300	-1.31567700	2.19510500
H	-3.20175400	-2.73700900	1.57979100
C	-7.22126400	-0.41646100	0.04347200
H	-7.65437600	0.58294000	0.04165600
H	-7.56522900	-0.95776700	-0.84035000
H	-7.53828900	-0.95050800	0.93432200
C	-5.46314400	1.48351100	-1.61932900
H	-5.87882900	2.36318800	-1.12027100
H	-4.62306100	1.80918000	-2.23044500
H	-6.23724500	1.07258000	-2.26988000
Te	1.34462900	0.21677500	-0.84783100
C	3.10091800	-0.68501200	0.00001300
C	3.00415700	-1.84451000	0.76172900
C	4.33875900	-0.11378200	-0.27143100
C	4.15980900	-2.42345900	1.27083600
H	2.04361900	-2.30299900	0.94567100
C	5.49045500	-0.71089300	0.22901100
H	4.41045700	0.80000700	-0.84366700
C	5.40250700	-1.86081100	1.00292100
H	4.08530900	-3.32090800	1.87116000
H	6.45483500	-0.26868400	0.01569400
H	6.30060400	-2.31950500	1.39584400
Br	0.22109300	-2.22875300	-1.18794900
Br	2.35671300	2.69087800	-0.44590400

Cartesian coordinates of the optimized geometry for *cis*-**IV** of **3d** at B3LYP-D3BJ/def2-TZVP,aug-cc-pVTZ-PP level of theory (number of imaginary frequencies = 0):

N	-2.25037300	-2.08903400	0.28550200
C	-0.83219000	-1.27369700	2.01020900
C	0.08855500	-0.35076100	1.49707800
C	-0.95419000	-1.38216400	3.40112300
C	0.82484000	0.45794100	2.35985400
C	-0.24054500	-0.56128800	4.25262800
H	-1.62801200	-2.13666800	3.78515500
C	0.65805600	0.36487900	3.73190100
H	1.52154000	1.17857600	1.95794800
H	-0.37025100	-0.65434400	5.32290800
H	1.23369700	1.00530300	4.38624800
N	-1.53711000	-2.27596000	1.28621900
Te	0.42467900	-0.04148600	-0.59209500
C	2.50474200	0.43229800	-0.42662500
C	3.35420700	-0.38101100	0.31376700
C	2.98787200	1.53337500	-1.12171000
C	4.70664300	-0.07212400	0.36969900
H	2.97379500	-1.24929200	0.83183200
C	4.34708900	1.82318000	-1.07227100
H	2.31800600	2.17616800	-1.67505600
C	5.20396100	1.02552300	-0.32495900
H	6.26013900	1.25779100	-0.28327500
H	4.72995500	2.67784000	-1.61429800
H	5.37203800	-0.69620000	0.95178100
Br	1.05410900	-2.60373100	-0.97328800
Br	-0.16783100	2.61418800	-0.20507900
C	-2.61433100	-0.85114100	-0.24778000
C	-2.99349100	0.37923800	0.31714600
C	-2.87983200	-0.72375900	-1.64443400
N	-3.40803400	1.12937100	-0.72157300
N	-3.32397500	0.48804700	-1.91841000
C	-3.90434000	2.48866800	-0.68400400
H	-3.18465200	3.13595800	-0.18585400
H	-4.02712100	2.80866300	-1.71366800
H	-4.86690800	2.53700300	-0.17171000
C	-3.06925600	0.85492200	1.72225300
H	-2.14174900	1.35056700	2.01752000
H	-3.88635700	1.56454800	1.84897200
H	-3.23416300	0.02077800	2.39928500
C	-2.65017100	-1.74806300	-2.69757400
H	-1.58369900	-1.92554100	-2.85287400
H	-3.08492500	-2.70246100	-2.39708700
H	-3.09364100	-1.42047300	-3.63600400

Cartesian coordinates of the optimized geometry for *trans*-**I** of **1e** at B3LYP-D3BJ/def2-TZVP,aug-cc-pVTZ-PP level of theory (number of imaginary frequencies = 0):

C	3.78815400	-1.91198900	0.05470400
C	2.72511000	-1.02899800	0.05557000
C	2.91102800	0.35334600	0.01592000
C	4.23539000	0.85822200	-0.02489400
C	5.30801500	-0.02353000	-0.02713200
C	5.08869700	-1.40093400	0.01217700
H	3.60321900	-2.97428500	0.08826000
H	1.71157000	-1.40096700	0.09071900
H	6.32891500	0.32251200	-0.05895600
N	1.87334900	1.28851300	0.01955000
N	0.71258300	0.82258400	0.02189300
C	-0.33941800	1.75650500	0.03386900
C	-0.17585700	3.14441800	0.05915600
C	-1.62236300	1.19718800	0.02018400
C	-1.28985900	3.96509100	0.07023600
H	0.82746200	3.54665500	0.06968500
C	-2.73698900	2.02694600	0.03115600

C	-2.56799200	3.40619700	0.05630200
H	-1.16936900	5.04044700	0.09007100
H	-3.73222400	1.60526900	0.02038900
H	-3.43940500	4.04888100	0.06535300
O	4.37782500	2.19787000	-0.06268700
O	6.20700900	-2.16521000	0.00695500
Te	-1.70203300	-0.92731800	-0.02040200
C	-3.83638700	-1.00209800	-0.01894800
C	-4.54086700	-1.00605200	-1.22205400
C	-4.53809500	-1.04526000	1.18502200
C	-5.93102700	-1.04707500	-1.22026800
H	-4.00308800	-0.97037500	-2.16022400
C	-5.92823700	-1.08617700	1.18510000
H	-3.99815400	-1.04025300	2.12261400
C	-6.62592200	-1.08685100	-0.01711800
H	-6.46996700	-1.04741300	-2.15934500
H	-6.46501900	-1.11709600	2.12490500
H	-7.70792900	-1.11898700	-0.01638400
C	6.06499200	-3.57779500	0.04349500
H	5.51620700	-3.94333900	-0.82821200
H	7.07525500	-3.97853600	0.02992700
H	5.55694100	-3.90134400	0.95557100
C	5.68657200	2.74279600	-0.10438000
H	6.26177400	2.46483600	0.78303300
H	6.22329100	2.42028700	-1.00078100
H	5.55637900	3.82158500	-0.12854900

Cartesian coordinates of the optimized geometry for *trans*-**II** of **1e** at B3LYP-D3BJ/def2-TZVP,aug-cc-pVTZ-PP level of theory (number of imaginary frequencies = 0):

C	4.90104400	0.93288000	-0.00022800
C	3.57844300	1.33799400	-0.00012100
C	2.52474600	0.42467900	0.00009300
C	2.83287200	-0.95670400	0.00020800
C	4.15705100	-1.37169700	0.00010200
C	5.18963300	-0.43372400	-0.00011300
H	5.68670600	1.67229400	-0.00041000
H	3.33007700	2.38916400	-0.00020700
H	4.42342200	-2.41679600	0.00021800
N	1.17367300	0.80221100	0.00017800
N	0.94551500	2.03945100	0.00006500
C	-0.39912300	2.40545000	0.00008100
C	-0.63253000	3.78893500	0.00005100
C	-1.48486700	1.50510200	0.00009900
C	-1.91755800	4.28989500	0.00006200
H	0.23122600	4.44066400	0.00002400
C	-2.77737500	2.03130200	0.00011100
C	-2.99286500	3.40110800	0.00009600
H	-2.08853100	5.35812200	0.00003700
H	-3.62724700	1.36479200	0.00013600
H	-4.00825300	3.77716200	0.00011700
O	1.78974000	-1.81149900	0.00044700
O	6.44294100	-0.95044900	-0.00020200
Te	-1.13244600	-0.57862500	0.00010100
C	-3.24012500	-1.06200000	-0.00013700
C	-3.92503000	-1.24294600	-1.20160100
C	-3.92525800	-1.24292900	1.20120100
C	-5.27225400	-1.58923300	-1.20302800
H	-3.40620400	-1.10499400	-2.14217000
C	-5.27248300	-1.58922400	1.20237700
H	-3.40661200	-1.10495700	2.14186500
C	-5.94789400	-1.76196500	-0.00038800
H	-5.79380800	-1.72336700	-2.14263000
H	-5.79421300	-1.72335500	2.14188200
H	-6.99643200	-2.03131300	-0.00048700
C	7.54466200	-0.05525400	-0.00051800

H	7.54251100	0.57640700	0.89168000
H	8.43502300	-0.67877200	-0.00053800
H	7.54224500	0.57609500	-0.89294400
C	2.03873300	-3.20759700	0.00048800
H	2.59107700	-3.51120300	-0.89248900
H	2.59129400	-3.51111200	0.89336000
H	1.06021600	-3.68081600	0.00063200

Cartesian coordinates of the optimized geometry for *cis*-**III** of **1e** at B3LYP-D3BJ/def2-TZVP,aug-cc-pVTZ-PP level of theory (number of imaginary frequencies = 0):

N	-1.81743500	-2.27093700	0.12028000
C	-2.86870800	-1.31032500	0.15303600
C	-4.14607400	-1.75602500	-0.18072400
H	-4.25778400	-2.78832000	-0.48559600
C	-5.23395200	-0.90608700	-0.16892800
H	-6.21821100	-1.24220900	-0.46200200
C	-5.06236000	0.41556800	0.24530800
C	-3.80931700	0.86538800	0.66186400
H	-3.68069800	1.87195300	1.02252700
C	-2.71484000	0.00323900	0.62684400
C	-0.39698600	-0.86423000	-1.12395800
C	0.83797100	-0.26404500	-0.86114900
C	-1.19976200	-0.39558700	-2.16443700
C	1.22816900	0.84938300	-1.59684300
C	-0.78619000	0.69219600	-2.91754700
H	-2.13494800	-0.89088900	-2.38346800
C	0.41998700	1.32280300	-2.62378300
H	2.16911300	1.33748000	-1.38450800
H	-1.40252700	1.04674000	-3.73319500
H	0.74260300	2.17609600	-3.20668200
N	-0.70391100	-2.03523800	-0.38139600
Te	1.97397400	-1.08973800	0.72932500
C	3.67487400	0.14757800	0.37900000
C	4.55945800	-0.15120900	-0.65677900
C	3.91363700	1.25895400	1.18523200
C	5.66286500	0.66128400	-0.89003400
H	4.38245600	-1.01505700	-1.28356200
C	5.02387900	2.06544800	0.95591900
H	3.23077300	1.49730300	1.98997600
C	5.89728800	1.76937300	-0.08332700
H	6.75950000	2.39849400	-0.26330600
H	6.34240800	0.42473900	-1.69897700
H	5.20251500	2.92643900	1.58760300
O	-1.48603600	0.34399300	1.07212200
O	-6.17501900	1.19404700	0.23524600
C	-6.06403900	2.54496900	0.64851300
H	-7.05878300	2.97161600	0.54781400
H	-5.74328200	2.61756400	1.69183900
H	-5.36577000	3.09921800	0.01470500
C	-1.17727100	1.70690700	1.30835500
H	-1.73850600	2.10208500	2.15944500
H	-0.11413200	1.72848100	1.53297800
H	-1.37526600	2.31007300	0.41896700

Cartesian coordinates of the optimized geometry for *cis*-**IV** of **1e** at B3LYP-D3BJ/def2-TZVP,aug-cc-pVTZ-PP level of theory (number of imaginary frequencies = 0):

N	1.54248200	1.22186700	-2.17235500
C	2.17031100	0.40370900	-1.18893800
C	2.59151200	0.79474800	0.10444500
C	3.20801900	-0.12933400	0.93387400
H	3.52625700	0.13417500	1.93049800
C	3.45359700	-1.43106600	0.49257800
C	3.11948500	-1.80656100	-0.80712900
H	3.32643000	-2.79642400	-1.18163400

C	2.51151100	-0.87268500	-1.63027600
C	-0.01953200	2.22585400	-0.70941600
C	-0.66331100	1.21261400	0.01349900
C	-0.13638800	3.55625100	-0.29140500
C	-1.34058200	1.55567600	1.18633000
C	-0.79953400	3.87791100	0.87633700
H	0.32604800	4.31950400	-0.90302100
C	-1.40065700	2.86856900	1.62553900
H	-1.84109900	0.78396300	1.75493400
H	-0.86161800	4.90974300	1.19699200
H	-1.93285500	3.10481300	2.53789400
N	0.63962200	2.04748000	-1.95260500
Te	-0.75796300	-0.81736400	-0.63240600
C	-2.78954600	-1.06121800	-0.03542700
C	-3.78548600	-0.23113100	-0.54912400
C	-3.13178200	-2.07676200	0.85426100
C	-5.10738700	-0.40910900	-0.16341000
H	-3.52759200	0.55638900	-1.24466500
C	-4.46068100	-2.26490600	1.22328400
H	-2.36230800	-2.71446900	1.26995600
C	-5.44876300	-1.42922600	0.71955800
H	-6.48103200	-1.57001300	1.01294800
H	-4.71876700	-3.05783600	1.91392400
H	-5.87415100	0.24384700	-0.56091200
H	2.24497100	-1.13066300	-2.64642600
O	2.40923900	2.08673800	0.44280400
O	4.04652300	-2.24746100	1.39819300
C	4.33087500	-3.58423400	1.01528500
H	4.78262800	-4.05407300	1.88515800
H	3.41776300	-4.12059400	0.74395300
H	5.03368100	-3.61624100	0.17843100
C	2.61130300	2.48413000	1.79046900
H	3.66421200	2.40723300	2.07324800
H	2.29278800	3.52140500	1.84115700
H	2.00010100	1.88314200	2.46741000

Cartesian coordinates of the optimized geometry for *trans*-**I** of **2e** at B3LYP-D3BJ/def2-TZVP,aug-cc-pVTZ-PP level of theory (number of imaginary frequencies = 0):

C	-5.59430100	0.76554300	0.38547300
C	-4.40371400	1.45968800	0.28736500
C	-3.18949300	0.84400000	-0.02523200
C	-3.19654600	-0.55757200	-0.29645300
C	-4.39300300	-1.25413900	-0.21359100
C	-5.58161600	-0.60758100	0.13426400
H	-6.50302000	1.28547200	0.64364400
H	-4.37572500	2.52623800	0.46441800
H	-4.43930900	-2.31365600	-0.40983500
N	-2.10546200	1.71326700	-0.07399900
N	-0.95476300	1.22695900	-0.12046100
C	0.11682300	2.13307500	-0.19670100
C	0.03061900	3.51721900	-0.34998700
C	1.36825300	1.52775900	-0.11168700
C	1.19705000	4.26285400	-0.40509600
H	-0.94430400	3.97827600	-0.42185600
C	2.53617800	2.26511200	-0.15189200
C	2.44347800	3.64495500	-0.30435900
H	1.14086100	5.33702700	-0.52544800
H	3.50008100	1.78473600	-0.06566800
H	3.34667500	4.23960300	-0.34489600
O	-2.04095600	-1.13773000	-0.65535700
O	-6.67280100	-1.40321400	0.19351100
Te	1.24285700	-0.57723300	0.15042400
C	3.37511100	-0.91197500	0.14646800
C	3.99367400	-1.34924900	1.31333600
C	4.11491900	-0.73006500	-1.01830100

C	5.36127400	-1.60132600	1.31158300
H	3.42369200	-1.47108200	2.22270200
C	5.48339400	-0.97290700	-1.00556700
H	3.63036000	-0.41158400	-1.92939300
C	6.10800000	-1.41067900	0.15612100
H	5.83999800	-1.94254400	2.22039500
H	6.05820800	-0.82268100	-1.91041400
H	7.17312900	-1.60340000	0.16004300
C	-7.92124300	-0.82185900	0.54431000
H	-8.22188800	-0.06339300	-0.18278700
H	-8.64042300	-1.63653300	0.53699400
H	-7.88316800	-0.37734900	1.54190800
C	-2.05323700	-2.47791400	-1.13442100
H	-2.30028200	-3.17842200	-0.33289600
H	-2.76315300	-2.59224100	-1.95650100
H	-1.04636100	-2.66212100	-1.49802200
Cl	1.25278900	-0.14463400	2.64030100
Cl	1.14332700	-0.83559400	-2.36807900

Cartesian coordinates of the optimized geometry for *trans*-**II** of **2e** at B3LYP-D3BJ/def2-TZVP,aug-cc-pVTZ-PP level of theory (number of imaginary frequencies = 0):

C	-4.93208100	0.88986200	-0.63328200
C	-3.64652400	1.35578600	-0.43652400
C	-2.65054400	0.54257000	0.10206700
C	-2.96834800	-0.79279200	0.45345500
C	-4.25727200	-1.26503500	0.26426000
C	-5.23614400	-0.42925800	-0.27803700
H	-5.67693400	1.53811000	-1.06725000
H	-3.37949600	2.36558400	-0.71325900
H	-4.53734800	-2.27362200	0.52442800
N	-1.33481300	0.94985900	0.29213000
N	-1.07733100	2.16972600	0.21211700
C	0.28859700	2.48364500	0.36214100
C	0.57182300	3.83928400	0.53140200
C	1.34122300	1.55758700	0.36110200
C	1.87366300	4.26875100	0.73446000
H	-0.25778500	4.53329200	0.51265000
C	2.63836700	1.98816200	0.57597900
C	2.90700000	3.34163600	0.76533500
H	2.08047700	5.32167300	0.87246000
H	3.45070900	1.27649700	0.59509800
H	3.92686700	3.66368400	0.92959500
O	-1.96964200	-1.52385100	0.98033700
O	-6.45573900	-0.99240700	-0.42754800
Te	0.96657900	-0.51667400	0.01970400
C	3.02410700	-0.98987700	-0.46530600
C	3.71602900	-1.88382100	0.34636300
C	3.62729700	-0.46664300	-1.60569000
C	5.01651300	-2.25063400	0.01583900
H	3.25839300	-2.27336700	1.24456300
C	4.93020100	-0.83027300	-1.92289600
H	3.08434100	0.21345500	-2.24587600
C	5.62563200	-1.72336300	-1.11548600
H	5.55233800	-2.94537500	0.64989700
H	5.39921200	-0.41609400	-2.80630500
H	6.63928500	-2.00698600	-1.36820900
C	-7.50942100	-0.20823300	-0.97085000
H	-7.27457500	0.11916700	-1.98660500
H	-8.38109400	-0.85686600	-0.99126100
H	-7.71709500	0.66268300	-0.34433400
C	-2.22298900	-2.85571000	1.40364900
H	-2.99782600	-2.88054200	2.17341400
H	-2.51896900	-3.48720900	0.56220700
H	-1.28442200	-3.20959100	1.82130300
Cl	0.47810900	0.08678300	-2.38809200

Cl	1.39466200	-0.88762700	2.49003000

Cartesian coordinates of the optimized geometry for *cis*-**III** of **2e** at B3LYP-D3BJ/def2-TZVP,aug-cc-pVTZ-PP level of theory (number of imaginary frequencies = 0):

N	1.94738800	-1.71445900	-0.97956100
C	3.14555500	-1.07424900	-0.58864900
C	4.30116300	-1.85944800	-0.58244800
H	4.19468200	-2.91178100	-0.81145400
C	5.53345700	-1.33032200	-0.26815200
H	6.42192100	-1.94390200	-0.23222600
C	5.64491500	0.04106700	-0.01321100
C	4.52201600	0.86467000	-0.09661300
H	4.60803500	1.92687100	0.05461900
C	3.27232600	0.31438900	-0.38691500
C	0.60735000	-0.78302800	0.74416200
C	-0.65632500	-0.20099000	0.81275200
C	1.44301700	-0.72176100	1.86254500
C	-1.06486400	0.52554300	1.91607500
C	1.02128200	-0.03550300	2.98987900
H	2.40885500	-1.20476800	1.84701100
C	-0.21420900	0.60818600	3.01273400
H	-2.03618600	0.99832800	1.93777600
H	1.66444900	0.00270600	3.85939800
H	-0.52733300	1.15327900	3.89337900
N	0.84109600	-1.51839100	-0.44634900
Te	-1.89189800	-0.59223500	-0.87389900
C	-3.64332300	0.40171800	-0.09173900
C	-4.31250000	-0.12814700	1.00717200
C	-4.10931500	1.55365200	-0.71630800
C	-5.44671100	0.51424200	1.49103900
H	-3.95989700	-1.03295100	1.47937000
C	-5.25131200	2.18086300	-0.22945900
H	-3.58252700	1.97100000	-1.56148900
C	-5.91881800	1.66674600	0.87484200
H	-6.80496500	2.16044900	1.25228800
H	-5.96170600	0.10397500	2.35025100
H	-5.61284500	3.07658000	-0.71799700
O	2.14849000	1.04648500	-0.51586100
O	6.88447900	0.49038400	0.29332500
C	7.07976100	1.88188400	0.47644800
H	8.14338200	2.01491900	0.65199400
H	6.77924900	2.44201100	-0.41318200
H	6.52496800	2.25088600	1.34320400
C	2.19404200	2.45643200	-0.36330000
H	2.86564400	2.90859200	-1.09706600
H	1.17857200	2.79762400	-0.53713700
H	2.51182200	2.72892700	0.64642600
Cl	-2.54024900	-2.75048100	0.23155500
Cl	-1.05419700	1.59946900	-1.86954900

Cartesian coordinates of the optimized geometry for *cis*-**IV** of **2e** at B3LYP-D3BJ/def2-TZVP,aug-cc-pVTZ-PP level of theory (number of imaginary frequencies = 0):

N	-1.64412100	-1.57787300	-2.00511200
C	-2.26798500	-0.60422900	-1.17380800
C	-2.79758800	-0.81618500	0.12028700
C	-3.42444200	0.22601000	0.78129000
H	-3.81531800	0.10589600	1.77920300
C	-3.54092800	1.48144500	0.18356100
C	-3.08375100	1.68618500	-1.11795500
H	-3.18901000	2.63959000	-1.61051400
C	-2.49004200	0.62857200	-1.78517800
C	-0.17798400	-2.40166000	-0.34434300
C	0.50975700	-1.34797700	0.26312500
C	-0.17523100	-3.65173400	0.28288000

C	1.10986500	-1.52692000	1.50674500
C	0.41410200	-3.82501600	1.51993800
H	-0.66954800	-4.46925200	-0.22406400
C	1.05158400	-2.75585300	2.14394400
H	1.62368100	-0.70290900	1.97997100
H	0.38685400	-4.79642900	1.99640200
H	1.52290300	-2.88198200	3.10936700
N	-0.77336200	-2.37968500	-1.63463100
Te	0.76294900	0.56632200	-0.65035200
C	2.72360100	0.92764200	0.13516600
C	3.73802200	0.00079000	-0.07295800
C	2.96382600	2.11408100	0.81628000
C	5.00738400	0.26390800	0.42496900
H	3.54710600	-0.90845400	-0.62370400
C	4.24344400	2.37462700	1.29514400
H	2.16319100	2.81598900	0.99914200
C	5.26231400	1.45037400	1.10430100
H	6.25521100	1.65325700	1.48429400
H	4.43695100	3.29941600	1.82281100
H	5.79840500	-0.45932200	0.27523200
H	-2.14356600	0.74683300	-2.80354500
O	-2.69466800	-2.06297000	0.62690200
O	-4.13084900	2.43094500	0.94398700
C	-4.18652900	3.75822300	0.44253600
H	-4.63915000	4.35513100	1.23000600
H	-3.18415400	4.13601500	0.22673500
H	-4.80375200	3.81778200	-0.45791600
C	-3.01211900	-2.27472600	1.99529800
H	-4.07833200	-2.12537900	2.18206400
H	-2.74698700	-3.30782300	2.20204300
H	-2.42627800	-1.61128200	2.63496900
Cl	-0.22332800	1.62604200	1.43943800
Cl	1.78869100	-0.52263200	-2.65122700

Cartesian coordinates of the optimized geometry for *trans*-**I** of **3e** at B3LYP-D3BJ/def2-TZVP,aug-cc-pVTZ-PP level of theory (number of imaginary frequencies = 0):

C	-5.68514900	0.86466700	-0.15862300
C	-4.51186400	1.50070900	0.19853600
C	-3.29982500	0.82443200	0.35405000
C	-3.29263300	-0.59279600	0.18166800
C	-4.47221400	-1.23569700	-0.16127800
C	-5.65767300	-0.51878300	-0.34343100
H	-6.59239900	1.43424100	-0.28192400
H	-4.49561500	2.57005600	0.35936600
H	-4.50779700	-2.30429900	-0.30268300
N	-2.23064400	1.63987800	0.70710600
N	-1.07719600	1.16641300	0.62447000
C	-0.01040800	1.99000100	1.01822200
C	-0.09557700	3.23400300	1.64263700
C	1.24018000	1.43507900	0.75627700
C	1.07317900	3.89092800	1.99385000
H	-1.06998000	3.65534300	1.84606300
C	2.41019900	2.07967500	1.10802100
C	2.31867900	3.31956600	1.73342700
H	1.01929300	4.85488600	2.48299900
H	3.37350100	1.63189500	0.91179700
H	3.22266000	3.83820100	2.02457400
O	-2.14200800	-1.25088200	0.39721000
O	-6.73108200	-1.26239000	-0.69163100
Te	1.08690900	-0.45114600	-0.21372200
C	3.19599600	-0.65068700	-0.60898900
C	3.87404100	-1.75392200	-0.10108600
C	3.85840200	0.28010300	-1.40368300
C	5.22421900	-1.92142600	-0.38832000
H	3.36658100	-2.46574700	0.53406300

C	5.21075000	0.11083700	-1.67425000
H	3.32606800	1.12231900	-1.82125900
C	5.89441900	-0.98948400	-1.17024700
H	5.74994200	-2.78071700	0.00745200
H	5.72603600	0.83944700	-2.28678100
H	6.94658100	-1.12030500	-1.38793600
C	-7.97467500	-0.60710500	-0.90169700
H	-7.90376000	0.12347000	-1.71124400
H	-8.67844500	-1.38780300	-1.17762700
H	-8.31857100	-0.11137200	0.00948500
C	-2.14700300	-2.67410900	0.42693200
H	-2.88365600	-3.04780400	1.14142000
H	-2.35152800	-3.08545500	-0.56448600
H	-1.15013800	-2.95856700	0.75280300
Br	0.67066200	0.82880800	-2.55192100
Br	1.32714600	-1.64029400	2.22619700

Cartesian coordinates of the optimized geometry for *trans*-**II** of **3e** at B3LYP-D3BJ/def2-TZVP,aug-cc-pVTZ-PP level of theory (number of imaginary frequencies = 0):

C	-5.06131400	0.98659400	-0.27881400
C	-3.78246500	1.41975400	0.01141100
C	-2.76353400	0.52244400	0.32849000
C	-3.05053300	-0.86518300	0.35165000
C	-4.33253300	-1.30628200	0.06672300
C	-5.33473100	-0.38604400	-0.24912300
H	-5.82474400	1.70326800	-0.53738000
H	-3.53816500	2.47185300	-0.01864300
H	-4.58951700	-2.35371300	0.07813500
N	-1.45305700	0.89950600	0.59296800
N	-1.21248200	2.10861500	0.79828900
C	0.15211600	2.39643500	0.99998600
C	0.42620700	3.67277900	1.49263200
C	1.21371300	1.51190100	0.75981000
C	1.72645500	4.05256300	1.78448100
H	-0.40929800	4.34097400	1.65255800
C	2.50965600	1.88842900	1.06892100
C	2.76822300	3.15622200	1.58267800
H	1.92579600	5.04166500	2.17483600
H	3.32692000	1.19973000	0.91331700
H	3.78619900	3.43753700	1.81769000
O	-2.03036500	-1.67923500	0.67722800
O	-6.54486100	-0.92305200	-0.51854900
Te	0.85094100	-0.42146800	-0.06782800
C	2.86635600	-0.68400200	-0.81046400
C	3.53677600	-1.86292800	-0.50244500
C	3.45488100	0.25862000	-1.64832100
C	4.80170100	-2.09628500	-1.03204800
H	3.09371300	-2.58728600	0.16657000
C	4.72401300	0.02591700	-2.16197500
H	2.92624200	1.16373700	-1.91035600
C	5.39771400	-1.15206600	-1.85764300
H	5.32058800	-3.01495600	-0.79043800
H	5.18217400	0.76429300	-2.80746200
H	6.38450900	-1.33256400	-2.26424300
C	-7.62075300	-0.05519200	-0.85083600
H	-7.40497300	0.51021000	-1.76061400
H	-8.47945300	-0.69952900	-1.01914300
H	-7.83762200	0.63589300	-0.03265500
C	-2.24705400	-3.08160200	0.75260800
H	-3.00865600	-3.31961300	1.49869000
H	-2.54220900	-3.48651100	-0.21849800
H	-1.29412900	-3.50488100	1.05827200
Br	1.55297400	-1.54470800	2.30934600
Br	0.15230900	0.69600100	-2.43300600

Cartesian coordinates of the optimized geometry for *cis*-**III** of **3e** at B3LYP-D3BJ/def2-TZVP,aug-cc-pVTZ-PP level of theory (number of imaginary frequencies = 0):

N	-2.08572700	1.42357100	-1.29143600
C	-3.32711300	0.96353100	-0.79150400
C	-4.43114900	1.78656900	-1.02254700
H	-4.25531300	2.73849400	-1.50567700
C	-5.70031800	1.42581000	-0.62276500
H	-6.54853100	2.07828000	-0.77149100
C	-5.89863100	0.17406100	-0.03580400
C	-4.82662700	-0.70286900	0.13464700
H	-4.98367600	-1.68320700	0.55083300
C	-3.54178800	-0.31719000	-0.24426900
C	-0.81648800	0.89968700	0.64719100
C	0.41455800	0.29306400	0.88439100
C	-1.66083300	1.16556100	1.72680800
C	0.76469900	-0.17395000	2.13799600
C	-1.29094600	0.75226500	2.99669900
H	-2.59410200	1.68563900	1.57161800
C	-0.09993400	0.05872200	3.20150000
H	1.70028500	-0.69055100	2.29468500
H	-1.94112900	0.96408400	3.83548800
H	0.16797700	-0.27986100	4.19359300
N	-1.00100700	1.31683700	-0.69468500
Te	1.66690400	0.18829800	-0.83100700
C	3.44018900	-0.47621800	0.18833800
C	3.97941600	0.26897500	1.23168800
C	4.07131400	-1.63183500	-0.25808400
C	5.14869700	-0.16289700	1.84540500
H	3.50684600	1.18485800	1.55464600
C	5.25053300	-2.04545900	0.35135100
H	3.64154800	-2.22147900	-1.05480900
C	5.78683800	-1.31657600	1.40504100
H	6.70205300	-1.64459200	1.88066600
H	5.56389800	0.41219700	2.66288500
H	5.74373000	-2.94288700	0.00132900
O	-2.46698500	-1.12077400	-0.14852800
O	-7.17214300	-0.12134700	0.32064500
C	-7.44416400	-1.38594300	0.90277200
H	-8.51199700	-1.39793000	1.10484400
H	-7.19568100	-2.20064100	0.21693000
H	-6.89583200	-1.51833900	1.83967300
C	-2.60077800	-2.41996800	0.40887000
H	-3.28897500	-3.03134100	-0.18016000
H	-1.60506200	-2.85082500	0.37112700
H	-2.94672900	-2.36382300	1.44441600
Br	0.85805500	-2.41018100	-1.13396800
Br	2.35557300	2.74711100	-0.43170800

Cartesian coordinates of the optimized geometry for *cis*-**IV** of **3e** at B3LYP-D3BJ/def2-TZVP,aug-cc-pVTZ-PP level of theory (number of imaginary frequencies = 0):

N	-1.44297100	-2.16415600	-1.63116900
C	-2.20240200	-1.07479300	-1.11812000
C	-2.87393100	-1.03163000	0.12572900
C	-3.63181200	0.07891400	0.45474200
H	-4.13447100	0.15595700	1.40571500
C	-3.73566600	1.15681200	-0.42518100
C	-3.13060500	1.10124700	-1.68061800
H	-3.22003300	1.91098900	-2.38697600
C	-2.40829500	-0.03022100	-2.01812200
C	-0.13659300	-2.48174000	0.31332600
C	0.44342400	-1.27619900	0.71779500
C	-0.15249900	-3.55121500	1.21494600
C	0.91707300	-1.12787200	2.01954400
C	0.30994600	-3.39911900	2.50709300

H	-0.55929700	-4.49210500	0.87020400
C	0.83914400	-2.17843100	2.91871000
H	1.34538300	-0.18722600	2.33306600
H	0.26890200	-4.23451400	3.19396300
H	1.21043300	-2.04959000	3.92644500
N	-0.58500700	-2.79643900	-0.99735300
Te	0.70158100	0.39648200	-0.58804300
C	2.56671300	1.04039000	0.24374300
C	3.59400500	0.12723900	0.44838500
C	2.73857100	2.38985300	0.52204300
C	4.80580800	0.57573100	0.95636900
H	3.45976200	-0.91696100	0.20682700
C	3.96276200	2.83061100	1.01334800
H	1.92614700	3.08964200	0.38744200
C	4.99271500	1.92565100	1.23461100
H	5.94175700	2.27077700	1.62382800
H	4.10348900	3.88170100	1.22864400
H	5.60628900	-0.13220200	1.12714100
H	-1.94708500	-0.11694100	-2.99328700
O	-2.76630700	-2.11947000	0.91738000
O	-4.45858300	2.20480500	0.02807100
C	-4.49575900	3.38802100	-0.75672800
H	-5.06502200	4.11082200	-0.17819700
H	-3.48730600	3.77068400	-0.93121400
H	-4.99544900	3.21518200	-1.71363700
C	-3.23964200	-2.04276200	2.25480100
H	-4.32767500	-1.94667500	2.28454300
H	-2.94293900	-2.97599600	2.72533600
H	-2.77813900	-1.20431100	2.78066700
Br	-0.61044300	1.97401100	1.21150700
Br	2.04273700	-1.09537400	-2.35878600

Cartesian coordinates of the optimized geometry for *trans*-**I** of **1f** at B3LYP-D3BJ/def2-TZVP,aug-cc-pVTZ-PP level of theory (number of imaginary frequencies = 0):

N	2.91982700	1.07051900	-0.00000900
C	3.88871900	0.04721600	-0.00002800
C	3.62026200	-1.32402100	-0.00008400
H	2.59825900	-1.67417700	-0.00011800
C	4.69143300	-2.19623100	-0.00009600
H	4.53752100	-3.26720800	-0.00014000
C	5.98586700	-1.67667400	-0.00005300
H	6.84223700	-2.34234900	-0.00006200
C	5.22045400	0.46553400	0.00001000
C	0.73561000	1.66947300	-0.00000900
C	-0.57702500	1.18034500	0.00000300
C	0.98014600	3.04653100	-0.00001700
C	-1.64055200	2.07620100	0.00000700
C	-0.08309300	3.92977500	-0.00001300
H	2.00399000	3.39265000	-0.00002600
C	-1.39112900	3.44247100	-0.00000100
H	-2.65887400	1.71419900	0.00001700
H	0.09812800	4.99643400	-0.00002000
H	-2.22415900	4.13416900	0.00000200
N	1.73465800	0.68101000	-0.00001300
Te	-0.80081300	-0.93335100	0.00001700
C	-2.93271800	-0.86890000	0.00002500
C	-3.63433500	-0.84685400	1.20450700
C	-3.63434300	-0.84682900	-1.20445100
C	-5.02407900	-0.79650000	1.20314200
H	-3.09544200	-0.86293100	2.14248400
C	-5.02408700	-0.79647400	-1.20307500
H	-3.09545700	-0.86288700	-2.14243200
C	-5.71973300	-0.77069000	0.00003600
H	-6.80144100	-0.73200700	0.00004100
H	-5.56192800	-0.77755800	2.14251700

H	-5.56194300	-0.77751100	-2.14244500
H	5.43529000	1.52848300	0.00005200
N	6.25376200	-0.37171000	-0.00000100

Cartesian coordinates of the optimized geometry for *trans*-**II** of **1f** at B3LYP-D3BJ/def2-TZVP,aug-cc-pVTZ-PP level of theory (number of imaginary frequencies = 0):

N	-2.00873800	0.29636100	-0.04323300
C	-3.29725100	-0.27115400	-0.00532200
C	-3.38322100	-1.65413400	-0.17995500
H	-2.47676400	-2.22562200	-0.34462500
C	-5.64950800	-1.62842500	0.03466400
H	-6.57270800	-2.19736100	0.04784900
C	-5.66973100	-0.24776600	0.22072200
H	-6.60945400	0.26436400	0.38015100
C	-4.47544600	0.44837700	0.20222800
C	-0.67290000	2.09247400	-0.02800900
C	0.52007500	1.33786700	-0.04112700
C	-0.62417700	3.49420100	-0.02979500
C	1.73221400	2.02791900	-0.06017400
C	0.58466000	4.15742400	-0.04909500
H	-1.56550000	4.02739000	-0.01745400
C	1.76533300	3.41444200	-0.06458400
H	2.66214100	1.47872900	-0.07028400
H	0.61654500	5.23851500	-0.05189200
H	2.72295300	3.91940200	-0.07924600
N	-1.95628800	1.54968900	-0.00868200
Te	0.46014900	-0.77459600	-0.02360900
C	2.60487500	-0.98165600	0.02705800
C	3.32784000	-1.11456800	-1.15779600
C	3.28168100	-1.02324500	1.24556900
C	4.70873200	-1.27816600	-1.12536700
H	2.81341400	-1.08401000	-2.11006200
C	4.66238600	-1.18616300	1.27861400
H	2.73092400	-0.92210900	2.17227200
C	5.37714900	-1.31300100	0.09292600
H	6.45173300	-1.44082800	0.11851100
H	5.26140800	-1.37839100	-2.05106900
H	5.17901700	-1.21499300	2.22976700
H	-4.43130500	1.51838400	0.34466700
N	-4.53017300	-2.32477200	-0.16297500

Cartesian coordinates of the optimized geometry for *cis*-**III** of **1f** at B3LYP-D3BJ/def2-TZVP,aug-cc-pVTZ-PP level of theory (number of imaginary frequencies = 0):

N	-2.72313700	-1.44262900	-0.77657900
C	-3.59685100	-0.65572400	0.02697800
C	-4.94887900	-0.61268300	-0.29083000
H	-5.30460100	-1.09906200	-1.18972100
C	-5.80434800	0.07279500	0.55715400
H	-6.86034300	0.15169000	0.33642900
C	-5.28371000	0.64565800	1.71152700
H	-5.92554300	1.18779200	2.39663500
C	-3.17280400	-0.07597300	1.22791200
C	-1.16984500	0.31225500	-1.01481800
C	0.16658200	0.46873600	-0.63532300
C	-1.94305100	1.41998400	-1.36408700
C	0.69699900	1.74999300	-0.53096100
C	-1.39221800	2.68910400	-1.29903800
H	-2.96243200	1.28099000	-1.69504800
C	-0.07877900	2.85288300	-0.86654500
H	1.71936000	1.88924800	-0.20902500
H	-1.98536900	3.54878700	-1.58050200
H	0.35082100	3.84443300	-0.80458700
N	-1.61706000	-1.03284300	-1.16021000
Te	1.25500500	-1.30685500	-0.20994400

C	3.05214800	-0.27976700	0.29568400
C	3.98821600	0.02046900	-0.69262800
C	3.29762400	0.09739200	1.61493900
C	5.15650700	0.69922700	-0.36380800
H	3.80222100	-0.27019000	-1.71791000
C	4.46724000	0.77552000	1.94053400
H	2.57405900	-0.13271200	2.38549300
C	5.39658500	1.07740300	0.95202200
H	6.30673900	1.60488200	1.20684000
H	5.87845500	0.93107500	-1.13647900
H	4.65065300	1.06754900	2.96670900
H	-2.13438000	-0.15223700	1.52934400
N	-3.99671400	0.55555400	2.05345400

Cartesian coordinates of the optimized geometry for *cis*-**IV** of **1f** at B3LYP-D3BJ/def2-TZVP,aug-cc-pVTZ-PP level of theory (number of imaginary frequencies = 0):

N	2.55401200	0.40792700	-1.70082000
C	2.87071800	-0.31727600	-0.51314200
C	3.05286800	-1.69403400	-0.67293600
H	2.88148800	-2.13487200	-1.64858300
C	3.65603900	-1.95951700	1.50522200
H	3.95822100	-2.63986000	2.29361800
C	3.55658000	-0.59400500	1.75303500
H	3.79127700	-0.19977400	2.73260700
C	3.15324600	0.24731400	0.73098200
C	1.07429700	1.88105100	-0.60774700
C	0.09634900	1.12935900	0.05791800
C	1.25281500	3.23228000	-0.29649800
C	-0.64179800	1.74777900	1.06856800
C	0.52953400	3.82146600	0.72396500
H	1.97632900	3.79784700	-0.86927900
C	-0.42372200	3.07341200	1.41026000
H	-1.40209600	1.18412900	1.59142900
H	0.69444100	4.86135800	0.97296000
H	-1.00754000	3.52499000	2.20164600
N	1.82662700	1.41144100	-1.72523200
Te	-0.35884300	-0.89431000	-0.43912900
C	-2.44688200	-0.66808700	-0.09593000
C	-3.19541700	0.22867700	-0.85721700
C	-3.07314600	-1.43552600	0.88308400
C	-4.55808400	0.36501400	-0.62737600
H	-2.71459800	0.82171800	-1.62356100
C	-4.44260600	-1.30908900	1.09685800
H	-2.49440000	-2.12335200	1.48544100
C	-5.18471800	-0.40638600	0.34651800
H	-6.24828900	-0.30314800	0.51828600
H	-4.92432500	-1.91011200	1.85758000
H	-5.13329300	1.06768200	-1.21679700
H	3.06607100	1.31124700	0.89126500
N	3.41365900	-2.50627100	0.31386900

Cartesian coordinates of the optimized geometry for *trans*-**I** of **2f** at B3LYP-D3BJ/def2-TZVP,aug-cc-pVTZ-PP level of theory (number of imaginary frequencies = 0):

N	3.02939300	1.14417100	-0.13667600
C	3.93808800	0.07073200	-0.09749600
C	3.59765500	-1.25959500	-0.35541700
H	2.58262000	-1.51714800	-0.62368200
C	4.60371000	-2.20534200	-0.29912300
H	4.39879000	-3.24784200	-0.50259700
C	5.89968600	-1.79445800	0.00926100
H	6.70552900	-2.51867500	0.05759700
C	5.27048900	0.38589900	0.17442200
C	0.87277700	1.84165400	-0.13438100
C	-0.44609900	1.41198600	-0.00792700

C	1.14184000	3.19346800	-0.35608200
C	-1.50339300	2.30061900	-0.07931000
C	0.08738500	4.08661500	-0.43769300
H	2.16882000	3.51442400	-0.45694700
C	-1.22886800	3.64567400	-0.29921000
H	-2.52178600	1.95971100	0.03881600
H	0.28593200	5.13666700	-0.60739900
H	-2.04504200	4.35354800	-0.35958500
N	1.83256500	0.81826100	-0.02808200
Te	-0.67664100	-0.66803400	0.36312300
C	-2.76689400	-0.75163700	-0.11908400
C	-3.66517200	-1.16350100	0.85863300
C	-3.20300800	-0.43048000	-1.40004900
C	-5.01726700	-1.25273400	0.54633600
H	-3.32509500	-1.38800500	1.85909200
C	-4.55831200	-0.50906800	-1.69565200
H	-2.49750300	-0.13373900	-2.16201400
C	-5.46494800	-0.92279300	-0.72644100
H	-6.51884500	-0.98838600	-0.96383600
H	-5.71823400	-1.57577800	1.30486200
H	-4.90173300	-0.25106300	-2.68902200
H	5.54263800	1.42038600	0.35280800
N	6.23559300	-0.52502900	0.24078700
Cl	-0.07066700	-1.13130800	-2.04689200
Cl	-1.22343000	-0.04341900	2.74410200

Cartesian coordinates of the optimized geometry for *trans*-**II** of **2f** at B3LYP-D3BJ/def2-TZVP,aug-cc-pVTZ-PP level of theory (number of imaginary frequencies = 0):

N	2.12435400	0.42205200	0.17523300
C	3.37997900	-0.18815600	0.02777100
C	3.50006700	-1.49048800	0.51587900
H	2.64801900	-1.95933700	0.99559300
C	5.67212700	-1.62036100	-0.14775600
H	6.57472500	-2.21852200	-0.20793800
C	5.64930600	-0.32834600	-0.67265100
H	6.53251300	0.08068900	-1.14468300
C	4.48291000	0.40646600	-0.58739900
C	0.76618100	2.19806900	0.04792500
C	-0.42646200	1.47231700	0.18007800
C	0.72268000	3.59230600	0.01604900
C	-1.62614300	2.14598300	0.31994000
C	-0.48255700	4.26331900	0.14641600
H	1.65785500	4.12319500	-0.10040000
C	-1.65614500	3.53882000	0.30477700
H	-2.54771800	1.59554000	0.44034800
H	-0.50564100	5.34452200	0.12766800
H	-2.60371800	4.05007900	0.41203800
N	2.05666200	1.64307300	-0.05911800
Te	-0.43560300	-0.66347500	0.17471000
C	-2.54935500	-0.85962800	-0.21878200
C	-3.35333400	-1.46393900	0.74278100
C	-3.08847400	-0.44936200	-1.43458900
C	-4.70654500	-1.65391400	0.48389700
H	-2.93927200	-1.76335100	1.69487900
C	-4.44375900	-0.63349500	-1.67871500
H	-2.45891000	0.00261000	-2.18703400
C	-5.25316500	-1.23707000	-0.72296600
H	-6.30751900	-1.38271200	-0.91961800
H	-5.33108200	-2.12455100	1.23211400
H	-4.86412900	-0.30769800	-2.62144200
H	4.40145700	1.40755300	-0.98569600
N	4.62278200	-2.19642500	0.43753600
Cl	0.08645200	-0.52983300	-2.28890200
Cl	-0.83678700	-0.50448000	2.66347800

Cartesian coordinates of the optimized geometry for *cis*-**III** of **2f** at B3LYP-D3BJ/def2-TZVP,aug-cc-pVTZ-PP level of theory (number of imaginary frequencies = 0):

N	2.79079000	-1.04376000	-1.16158200
C	3.74718200	-0.13945900	-0.62242000
C	3.42278900	1.16621700	-0.26036800
H	2.40128100	1.51498600	-0.32835600
C	4.45129600	1.99753300	0.14761800
H	4.25408500	3.02426500	0.42524400
C	5.74849100	1.49577000	0.19115200
H	6.57035000	2.12382100	0.51627800
C	5.08403600	-0.53625800	-0.59955100
C	1.34101600	-0.84095200	0.69681900
C	0.00263000	-0.49436000	0.85554300
C	2.16520500	-0.91807300	1.81966200
C	-0.51385000	-0.15999800	2.09419900
C	1.64592400	-0.61210700	3.06822300
H	3.19518500	-1.22599000	1.71589100
C	0.31798700	-0.21880000	3.20696300
H	-1.55316400	0.11555100	2.20110600
H	2.28203200	-0.68256300	3.94052200
H	-0.07876800	0.01885900	4.18489800
N	1.70607800	-1.27593700	-0.61409100
Te	-1.17540700	-0.55922600	-0.91100100
C	-2.83205300	0.57219300	-0.14328200
C	-4.08558000	-0.02750300	-0.10143600
C	-2.65664400	1.89019900	0.26360500
C	-5.17481400	0.70484600	0.35759200
H	-4.21376300	-1.05822400	-0.39768000
C	-3.75008700	2.60635600	0.73477900
H	-1.68555100	2.35849500	0.20413100
C	-5.00847900	2.01764700	0.77930000
H	-5.85804000	2.58229700	1.14081800
H	-6.15208700	0.24117500	0.38796000
H	-3.61463900	3.62929700	1.06111100
H	5.35125800	-1.53643800	-0.92256600
N	6.06534800	0.25280600	-0.17442800
Cl	-0.00740800	1.58688900	-1.61885400
Cl	-2.17746500	-2.67965700	-0.03038100

Cartesian coordinates of the optimized geometry for *cis*-**IV** of **2f** at B3LYP-D3BJ/def2-TZVP,aug-cc-pVTZ-PP level of theory (number of imaginary frequencies = 0):

N	-2.64983000	0.87270200	1.52794700
C	-2.96672400	-0.13557700	0.56912200
C	-3.09818700	-1.42993300	1.08105200
H	-2.90063500	-1.60001400	2.13386700
C	-3.72592000	-2.25939400	-0.94160900
H	-4.01099100	-3.12781300	-1.52418500
C	-3.68203600	-1.00048400	-1.53135800
H	-3.94103100	-0.87881000	-2.57408300
C	-3.28956000	0.08509000	-0.76999800
C	-1.22524000	2.04339100	0.06334800
C	-0.20202800	1.21808200	-0.40500200
C	-1.51329800	3.22429900	-0.62323500
C	0.46966400	1.53572400	-1.57970700
C	-0.85041000	3.53221000	-1.79861600
H	-2.27348100	3.88013200	-0.21967900
C	0.14032600	2.68431600	-2.28386700
H	1.24653900	0.88257300	-1.95004400
H	-1.09969900	4.43998300	-2.33206300
H	0.66804800	2.92247800	-3.19739600
N	-1.92699700	1.85005500	1.29400600
Te	0.39729700	-0.54654400	0.63629600
C	2.44828900	-0.51154300	0.02647800
C	3.22968100	0.60979900	0.27713100

C	2.97987700	-1.63876300	-0.58705100
C	4.56293300	0.60319700	-0.11196700
H	2.81216600	1.47095100	0.77747400
C	4.32108100	-1.63927900	-0.95465400
H	2.35828000	-2.49567000	-0.80191500
C	5.11001300	-0.52001000	-0.72254600
H	6.15159900	-0.52210400	-1.01641000
H	4.74268800	-2.51620100	-1.42805800
H	5.17436700	1.47728900	0.07012800
H	-3.24386500	1.07440600	-1.19900200
N	-3.44500500	-2.47677600	0.34297400
Cl	-0.16972700	-1.85323300	-1.45235600
Cl	0.96729400	0.86996600	2.61555500

Cartesian coordinates of the optimized geometry for *trans*-**I** of **3f** at B3LYP-D3BJ/def2-TZVP,aug-cc-pVTZ-PP level of theory (number of imaginary frequencies = 0):

N	-3.11327800	0.98546900	0.72644800
C	-4.01793100	0.14318500	0.05588800
C	-3.68906500	-1.10450500	-0.48046600
H	-2.68555200	-1.49233500	-0.37741600
C	-4.69345400	-1.82741900	-1.09517400
H	-4.49734400	-2.80448800	-1.51591600
C	-5.97670900	-1.28547600	-1.15282400
H	-6.78106900	-1.83537900	-1.62907700
C	-5.33897400	0.58695700	-0.02886000
C	-0.96291400	1.51252100	1.20956100
C	0.36276600	1.20013500	0.91706500
C	-1.24825900	2.51652400	2.13621400
C	1.41156100	1.87931300	1.50978200
C	-0.20296500	3.19237700	2.74210600
H	-2.28037200	2.74706000	2.35855700
C	1.12028500	2.87897500	2.43104000
H	2.43586200	1.64607600	1.25824100
H	-0.41424000	3.97371200	3.46018300
H	1.92941100	3.41886500	2.90500200
N	-1.91106300	0.74295100	0.51074600
Te	0.60556800	-0.32398900	-0.54531300
C	2.68056100	-0.70046800	-0.15067400
C	3.58376300	-0.62695100	-1.20472400
C	3.10032200	-1.05861800	1.12586300
C	4.92438700	-0.91406100	-0.97369800
H	3.25864700	-0.32517900	-2.19009200
C	4.44493300	-1.32793100	1.34820200
H	2.39080100	-1.14098000	1.93617900
C	5.35614500	-1.25972100	0.30046400
H	6.40147200	-1.47696500	0.47752900
H	5.62911700	-0.85967800	-1.79305300
H	4.77585500	-1.59893400	2.34230100
H	-5.60255100	1.54514400	0.40523500
N	-6.30218300	-0.10260100	-0.63052200
Br	-0.13790800	-2.18566400	1.27677500
Br	1.22674800	1.63825600	-2.29582400

Cartesian coordinates of the optimized geometry for *trans*-**II** of **3f** at B3LYP-D3BJ/def2-TZVP,aug-cc-pVTZ-PP level of theory (number of imaginary frequencies = 0):

N	-2.14589600	0.45431500	-0.27286000
C	-3.39912400	-0.15700600	-0.11421900
C	-3.47736500	-1.51179400	-0.44179000
H	-2.59682400	-2.02015700	-0.81887300
C	-5.67864400	-1.59883500	0.12584000
H	-6.57526700	-2.20206300	0.21659500
C	-5.69901600	-0.25151900	0.48566700
H	-6.61008000	0.19611300	0.85944900
C	-4.53911000	0.48886500	0.36666700

C	-0.81834600	2.25526600	-0.31935600
C	0.38600500	1.53617600	-0.30208700
C	-0.78828100	3.64291900	-0.46066200
C	1.58543800	2.20412800	-0.46969500
C	0.41624600	4.30988300	-0.61518800
H	-1.73268100	4.17016200	-0.45771900
C	1.60226300	3.58821000	-0.62645200
H	2.51621300	1.65662000	-0.48118800
H	0.42941400	5.38518200	-0.73075500
H	2.54944500	4.09564200	-0.75332400
N	-2.10636100	1.69740000	-0.20850100
Te	0.39962100	-0.58348600	-0.03956600
C	2.50880200	-0.73122400	0.40022700
C	3.30127600	-1.52446400	-0.42379900
C	3.05350600	-0.11611300	1.52381600
C	4.64759900	-1.69749700	-0.12144400
H	2.88519800	-1.98661500	-1.30781900
C	4.40208700	-0.28532600	1.81091900
H	2.43369400	0.48233300	2.17575200
C	5.19938600	-1.07689100	0.99182300
H	6.24850400	-1.21001200	1.22221900
H	5.26274100	-2.31471100	-0.76324300
H	4.82620100	0.19854000	2.68126800
H	-4.49034000	1.53278400	0.64107300
N	-4.59372400	-2.22321200	-0.33122600
Br	-0.23647000	-0.15741500	2.55165600
Br	0.80158500	-0.74517800	-2.71164100

Cartesian coordinates of the optimized geometry for *cis*-**III** of **3f** at B3LYP-D3BJ/def2-TZVP,aug-cc-pVTZ-PP level of theory (number of imaginary frequencies = 0):

N	-2.86172400	-0.80368000	1.31675300
C	-3.93026900	-0.23534000	0.57362900
C	-5.20431900	-0.77159700	0.72115000
H	-5.34957800	-1.65097400	1.33470400
C	-6.25442000	-0.16283100	0.05324000
H	-7.25895300	-0.55838000	0.11851100
C	-5.99626900	0.98132600	-0.69338100
H	-6.79452400	1.48010100	-1.23130500
C	-3.77388800	0.94365300	-0.16470000
C	-1.43420300	-0.99332800	-0.56584200
C	-0.12110800	-0.62073200	-0.84075100
C	-2.25091400	-1.43569700	-1.60720200
C	0.37205900	-0.61246400	-2.13259600
C	-1.75398800	-1.45463100	-2.90112100
H	-3.25675800	-1.77150600	-1.40458400
C	-0.45585700	-1.02945700	-3.16873500
H	1.39224300	-0.31828000	-2.33171900
H	-2.38389900	-1.80518500	-3.70774000
H	-0.07692400	-1.04719300	-4.18180400
N	-1.75391600	-1.05084400	0.82414800
Te	1.05865500	-0.15279000	0.86550100
C	2.66218000	0.80143900	-0.19619900
C	3.95725500	0.33863500	0.00623400
C	2.41414100	1.89028800	-1.02463000
C	5.01508300	0.97505800	-0.63326200
H	4.14481200	-0.52172800	0.63237600
C	3.47744700	2.50748300	-1.67155100
H	1.40981700	2.26527000	-1.15593000
C	4.77676500	2.05427700	-1.47450400
H	5.60228200	2.54327600	-1.97514600
H	6.02436600	0.61853100	-0.47465700
H	3.28610900	3.34947100	-2.32393600
H	-2.80431600	1.42415800	-0.22006600
N	-4.78672900	1.53780800	-0.78285200
Br	2.21593900	-2.56854200	0.56417700

Br	-0.27783500	2.19991400	1.05668700

Cartesian coordinates of the optimized geometry for *cis*-**IV** of **3f** at B3LYP-D3BJ/def2-TZVP,aug-cc-pVTZ-PP level of theory (number of imaginary frequencies = 0):

N	-2.48656100	-1.83748800	-0.82534400
C	-2.97532000	-0.51881100	-0.58660400
C	-3.14528500	0.27303100	-1.72651500
H	-2.84047700	-0.12198800	-2.68956100
C	-4.06963800	1.96466400	-0.51943200
H	-4.48964400	2.96381900	-0.52033500
C	-3.99450600	1.23455700	0.66203700
H	-4.36238300	1.65702200	1.58685400
C	-3.43158800	-0.02829500	0.63723100
C	-1.15527400	-1.87529400	1.11673000
C	-0.22557600	-0.83425300	1.11343400
C	-1.44945700	-2.51886500	2.32041800
C	0.34213200	-0.39982300	2.30623700
C	-0.89040900	-2.07774200	3.50690600
H	-2.13194700	-3.35827600	2.29988100
C	0.00406300	-1.01159400	3.50356600
H	1.04266400	0.42245100	2.30258800
H	-1.14509800	-2.57190700	4.43516100
H	0.44976000	-0.66548300	4.42617700
N	-1.73218000	-2.44904400	-0.05794700
Te	0.37354700	0.13795200	-0.69130900
C	2.36125900	0.62534600	-0.07091000
C	3.18169600	-0.35095400	0.48030000
C	2.81796300	1.92041800	-0.27732000
C	4.47761900	-0.01318300	0.84747300
H	2.82549400	-1.36212100	0.61133500
C	4.12418600	2.24062400	0.07602800
H	2.16496100	2.68035800	-0.68181300
C	4.95061600	1.27834900	0.64178300
H	5.96423600	1.53344200	0.92222200
H	4.48824700	3.24641500	-0.08690000
H	5.11912000	-0.76560500	1.28712400
H	-3.35933300	-0.61962600	1.53716600
N	-3.65714000	1.49679900	-1.69730300
Br	-0.50776100	2.47633800	0.36003700
Br	1.26336900	-2.19442200	-1.66306100

Cartesian coordinates of the optimized geometry structure for the singlet ground state (S_0; *trans* isomer) of **S1** at PBE0-D3(SMD,DCM)/def2-TZVP level of theory (number of imaginary frequencies = 0; energy: -879.629458 a.u.):

N	-0.98729100	0.45549000	-0.07853700
C	-2.39186500	0.37953100	-0.03736100
C	-2.95092400	-0.86033200	-0.34017400
H	-2.29491600	-1.68348700	-0.60007600
C	-4.32663400	-1.02310800	-0.31915700
H	-4.75934600	-1.98707500	-0.56195100
C	-5.14703700	0.04627800	0.01451000
H	-6.22363100	-0.08121900	0.03608500
C	-4.58661700	1.28115700	0.32830400
H	-5.22785000	2.11325800	0.59782100
C	-3.21469300	1.45461100	0.30454800
C	0.89955200	1.63234700	-0.05349000
C	1.72501000	0.48959900	-0.02861100
C	1.45892600	2.91452400	-0.08133000
C	3.10567400	0.68829100	-0.03945300
C	2.82609100	3.08690500	-0.09151800
H	0.78112300	3.76122900	-0.09636600
C	3.64807700	1.96309800	-0.07075600
H	3.77831400	-0.15942000	-0.02358100
H	3.25404100	4.08220100	-0.11486800

H	4.72659900	2.07888900	-0.07757700
N	-0.49119000	1.60069700	-0.04579400
Te	0.89979200	-1.43434200	0.03631500
H	-2.76906600	2.40913100	0.55550100
C	2.79208000	-2.45055400	0.18577000
H	3.40015600	-2.29848600	-0.70616900
H	3.33659700	-2.14809000	1.08053400
H	2.53233300	-3.50852200	0.26488600

Cartesian coordinates of the optimized geometry structure for the singlet ground state (S_0; *cis* isomer) of **S1** at PBE0-D3(SMD,DCM)/def2-TZVP level of theory (number of imaginary frequencies = 0; energy: -879.601118 a.u.):

N	1.17581100	0.81567800	1.89426400
C	1.91721200	0.63666800	0.69281400
C	2.80202000	-0.44039700	0.69432600
H	2.82697400	-1.09554200	1.55807800
C	3.61737800	-0.66834800	-0.40137700
H	4.28515400	-1.52230400	-0.40737200
C	3.59279600	0.20861300	-1.47765000
H	4.24249200	0.04176500	-2.32943500
C	2.74921200	1.31318400	-1.45384500
H	2.74800500	2.01322700	-2.28185000
C	1.90338500	1.52947200	-0.37909400
C	-0.80788100	1.45208600	0.79845600
C	-1.21196300	0.43342400	-0.07377400
C	-1.34927000	2.73268600	0.68777200
C	-2.12258700	0.75621200	-1.08033800
C	-2.23276700	3.03641600	-0.32981800
H	-1.04949600	3.48517600	1.40938200
C	-2.62157200	2.04107300	-1.21847800
H	-2.44705800	-0.00915300	-1.77578300
H	-2.62641100	4.04220100	-0.42231400
H	-3.32329000	2.26043100	-2.01545400
N	0.01438100	1.23474600	1.93943400
Te	-0.55889300	-1.57110300	0.11032200
H	1.25521900	2.39560500	-0.36473700
C	-2.41210200	-2.44023100	-0.49305600
H	-2.56986000	-2.31488900	-1.56239400
H	-2.32969400	-3.50186600	-0.25626000
H	-3.22412600	-1.99539000	0.07988000

Cartesian coordinates of the optimized geometry structure for the singlet ground state (S_0; transition state) of **S1** at PBE0-D3(SMD,DCM)/def2-TZVP level of theory (number of imaginary frequencies = 1; energy: -879.574090 a.u.):

N	-0.95927000	0.73629100	-1.44765600
C	-2.01079200	0.69420700	-0.57368100
C	-2.99649500	-0.28639700	-0.80150400
H	-2.87829300	-0.95214100	-1.64896100
C	-4.07631200	-0.39272800	0.04918800
H	-4.82761100	-1.15412300	-0.12846400
C	-4.20641000	0.47442500	1.13310500
H	-5.05911700	0.39058000	1.79713300
C	-3.24180900	1.45480200	1.35722100
H	-3.34827800	2.13508500	2.19523400
C	-2.15078600	1.57280700	0.52021600
C	1.06876800	1.51119500	-0.58629100
C	1.48746500	0.32216400	0.08534200
C	1.89139400	2.66491900	-0.51520500
C	2.66582100	0.36655900	0.82542900
C	3.04851600	2.67102900	0.22020800
H	1.56015000	3.54529900	-1.05456300
C	3.43793000	1.51599100	0.90342100
H	3.00126200	-0.51459200	1.35885100
H	3.65567700	3.56770900	0.27008300

H	4.34856600	1.50847200	1.49168400
N	-0.04487700	1.64615300	-1.33259600
Te	0.44309500	-1.49098400	-0.10549700
H	-1.40461600	2.34006300	0.68811100
C	2.07538100	-2.74889900	0.44824200
H	2.28777400	-2.66534800	1.51295100
H	1.74404100	-3.76317300	0.22064100
H	2.95250600	-2.50420600	-0.14924800

Cartesian coordinates of the optimized geometry structure for the singlet ground state (S_0; *trans* isomer) of **S2** at PBE0-D3(SMD,DCM)/def2-TZVP level of theory (number of imaginary frequencies = 0; energy: -1799.811160 a.u.):

N	-1.27116900	0.62677900	-0.13640600
C	-2.65941200	0.42421100	-0.16752700
C	-3.07245700	-0.90065100	-0.29012700
H	-2.32306400	-1.68147100	-0.35321200
C	-4.42433000	-1.20075000	-0.33322000
H	-4.74673900	-2.23094600	-0.43105000
C	-5.36064900	-0.17994700	-0.24974600
H	-6.41941100	-0.41191800	-0.28187300
C	-4.94513800	1.14399400	-0.12221900
H	-5.68162400	1.93686900	-0.05381200
C	-3.59975600	1.45380100	-0.08029800
C	0.53148700	1.93779900	-0.05878800
C	1.46407200	0.89122000	-0.01344600
C	0.97910100	3.25638100	-0.07872600
C	2.81558300	1.18647300	-0.01363000
C	2.33265100	3.54460800	-0.06745600
H	0.23455500	4.04404800	-0.10771000
C	3.25110000	2.50779100	-0.03953600
H	3.55584000	0.39633300	0.00200600
H	2.66829400	4.57479000	-0.08384800
H	4.31482100	2.71637800	-0.03627600
N	-0.86675100	1.79773200	-0.09197400
Te	0.85810900	-1.13847200	0.05501700
H	-3.26535600	2.47858500	0.02117700
C	2.71037500	-1.92278800	0.73923300
H	3.40648900	-1.97512700	-0.09540600
H	3.08490900	-1.30269500	1.55080400
H	2.47645100	-2.92351000	1.10508000
Cl	0.26172300	-0.79210000	2.46256300
Cl	1.67373900	-1.33997100	-2.33581000

Cartesian coordinates of the optimized geometry structure for the singlet ground state (S_0; *cis* isomer) of **S2** at PBE0-D3(SMD,DCM)/def2-TZVP level of theory (number of imaginary frequencies = 0; energy: -1799.786430 a.u.):

N	-1.25786300	0.65118100	-2.10958100
C	-2.15277300	0.16875500	-1.11453400
C	-2.56408600	-1.15303800	-1.27840900
H	-2.14459000	-1.73617200	-2.09077100
C	-3.48531300	-1.70230100	-0.40330400
H	-3.78319600	-2.73852400	-0.51502100
C	-4.04088100	-0.91879100	0.60015000
H	-4.77624800	-1.34219900	1.27489900
C	-3.66747700	0.41304000	0.72897900
H	-4.11807800	1.03197000	1.49655500
C	-2.71686300	0.96187600	-0.11585800
C	0.06472600	1.85907200	-0.58671600
C	0.68486300	1.03498700	0.35171500
C	-0.04328300	3.22429300	-0.33199700
C	1.12891200	1.56751200	1.55648200
C	0.38940500	3.74536900	0.87263400
H	-0.48925500	3.86064200	-1.08845800
C	0.97192700	2.91696100	1.82374200

H	1.59552600	0.93071200	2.29866100
H	0.27753000	4.80584100	1.06773000
H	1.32039900	3.32147300	2.76665100
N	-0.32054100	1.42245300	-1.88805400
Te	1.02436800	-1.01996500	-0.02942900
H	-2.44116800	2.00302800	-0.01773100
C	2.83479600	-1.20657600	1.04056700
H	3.42397100	-0.31302000	0.84625200
H	2.60336700	-1.33473500	2.09503300
H	3.32530500	-2.09045300	0.63334500
Cl	-0.19982100	-1.58129200	2.08876900
Cl	2.46831500	-0.38004700	-1.97783200

Cartesian coordinates of the optimized geometry structure for the singlet ground state (S_0; transition state) of **S2** at PBE0-D3(SMD,DCM)/def2-TZVP level of theory (number of imaginary frequencies = 1; energy: -1799.757971 a.u.):

N	-1.02045800	0.63774800	-1.65079700
C	-2.16641200	0.52023900	-0.91480300
C	-2.91153900	-0.66394100	-1.08025700
H	-2.54861600	-1.41214300	-1.77565400
C	-4.06802300	-0.85556000	-0.35562400
H	-4.63379300	-1.77217500	-0.47749700
C	-4.51088800	0.12421200	0.53150000
H	-5.42209200	-0.02947000	1.09806400
C	-3.78769400	1.30594600	0.68472000
H	-4.14124800	2.07039800	1.36764500
C	-2.62528100	1.51489000	-0.02697300
C	0.65317000	1.85927300	-0.58890200
C	1.13402500	0.85591400	0.28914100
C	1.17713500	3.16339700	-0.46849600
C	2.02991100	1.18899400	1.28788900
C	2.06908200	3.47674500	0.53163100
H	0.82643400	3.91262200	-1.16887000
C	2.48704800	2.49526900	1.42626200
H	2.39461400	0.43524100	1.97537600
H	2.44240600	4.49026000	0.62180000
H	3.18543300	2.73491000	2.21927500
N	-0.26885200	1.68109200	-1.57056800
Te	0.65763800	-1.18392200	-0.00697000
H	-2.06615000	2.43586200	0.08426900
C	2.35565100	-2.00889600	0.94634400
H	3.23122200	-1.44696800	0.62899300
H	2.20353300	-1.97584900	2.02259200
H	2.40979600	-3.03821300	0.59120900
Cl	-0.52994100	-1.17177600	2.21531100
Cl	2.05519700	-1.11830100	-2.08883000

7. Crystal Structure Data

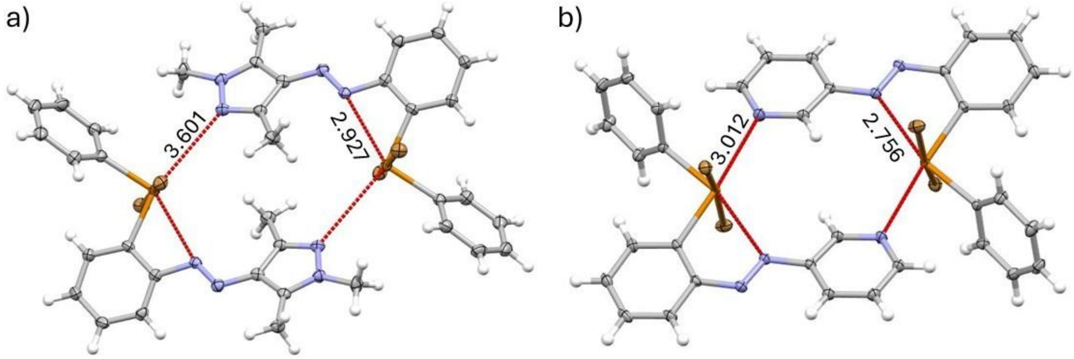

Figure S71. Solid state structures of a) **3d** and b) **3f**. Te···N interactions are visualized as red dashed lines. The distances are given in angstrom.

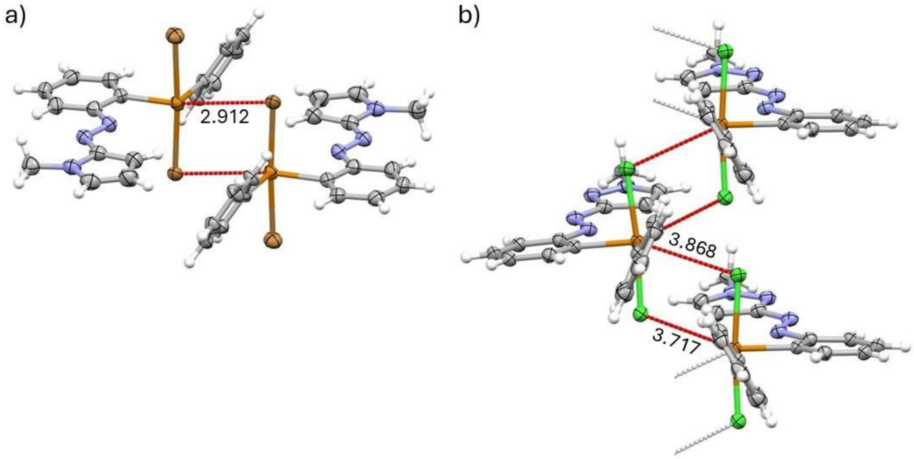

Figure S72. Solid state structures of a) **3c** and b) **2b**. Te···X interactions are visualized as red dashed lines. The distances are given in angstrom.

Crystal Structure Data of 6e

Table S3. Crystal structure data and structure refinement for **6e**.

Deposition number	2444469
Empirical formula	$C_{14}H_{13}IN_2O_2$
Formula weight	368.16
Density (calculated)	1.795 g·cm^{-3}
F(000)	2880
Temperature	100(2) K
Crystal size	0.463 × 0.250 × 0.194 mm
Crystal color	orange
Crystal description	tablet
Wavelength	0.71073 Å
Crystal system	tetragonal
Space group	$I4_1/a$
Unit cell dimensions	
a [Å]	20.8327(5)
b [Å]	20.8327(5)
c [Å]	12.5560(4)
α [°]	90
β [°]	90
γ [°]	90
Volume	5449.3(3) Å^3
Z	16
Cell measurement reflections used	9388
Cell measurement θ min/max	2.77°/40.32°
Diffractometer control software	BRUKER APEX3(v2019.1-0)
Diffractometer measurement device	Bruker D8 KAPPA II (APEX II detector)
Diffractometer measurement method	Data collection strategy APEX 3/Queen
θ range for data collection	2.722°- 40.444°
Completeness to θ = 25.242°	99.9%
Completeness to θ_{max} = 30.595°	99.9%
Index ranges	$-37 \leq h \leq 38$
	$-37 \leq k \leq 38$
	$-22 \leq l \leq 22$
Computing data reduction	BRUKER APEX3(v2019.1-0)
Absorption coefficient	2.350 mm^{-1}
Absorption correction	Numerical
Computation absorption correction	SADABS
Max./min. Transmission	0.33/0.23
R_{merg} before/after correction	0.0507/0.0408
Computing structure solution	BRUKER APEX3(v2019.1-0)
Computing structure refinement	SHELXL-2017/1 (Sheldrick, 2017)
Refinement method	Full-matrix least-squares on F^2
Reflections collected	199601
Independent reflections	8674
R_{int}	0.0265
Reflections with $I > 2\sigma(I)$	7950
Restraints	0
Parameter	174
GooF	1.174
Weighting details	$w = 1/[\sigma^2(F_{obs}^2) + (0.0266P)^2 + 5.0786P]$

	where P = $(F_{obs}^2 + 2F_{calc}^2)/3$
R_1 [$I > 2\sigma(I)$]	0.0231
wR_2 [$I > 2\sigma(I)$]	0.0601
R_1 [all data]	0.0264
wR_2 [all data]	0.0616
Largest diff. peak and hole	1.791/-0.388

Comments

Treatment of hydrogen atoms

Riding model on idealized geometries with the 1.2 fold isotropic displacement parameters of the equivalent U_{ij} of the corresponding carbon atom. The methyl groups are idealized with tetrahedral angles in a combined rotating and rigid group refinement with the 1.5 fold isotropic displacement parameters of the equivalent U_{ij} of the corresponding carbon atom.

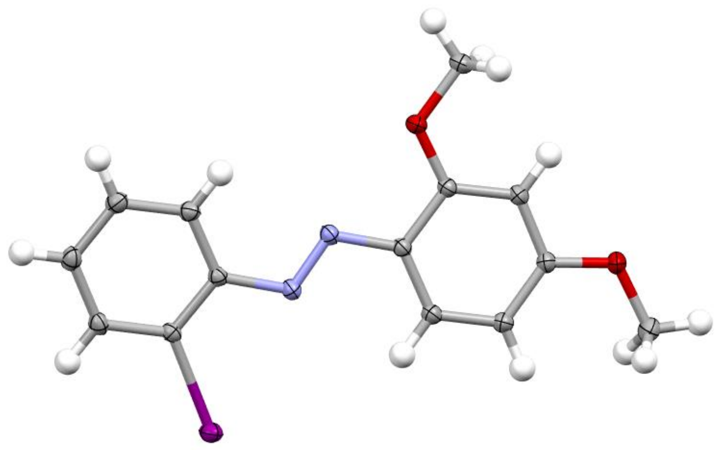

Figure S73. Molecular structure of **6e** with thermal ellipsoids at 50% probability level.

Crystal Structure Data of 6f

Table S4. Crystal structure data and structure refinement for **6f**.

Deposition number	2444470
Empirical formula	$C_{11}H_8IN_3$
Formula weight	309.10
Density (calculated)	1.902 g·cm^{-3}
F(000)	1184
Temperature	100(2) K
Crystal size	0.174 × 0.084 × 0.050 mm
Crystal color	orange
Crystal description	plate
Wavelength	0.71073 Å
Crystal system	monoclinic
Space group	*C*2/*c*
Unit cell dimensions	
a [Å]	28.927(2)
b [Å]	4.3401(4)
c [Å]	18.4175(15)
α [°]	90
β [°]	110.9852(16)
γ [°]	90
Volume	2158.9(3) Å^3
Z	8
Cell measurement reflections used	9882
Cell measurement θ min/max	3.23°/33.19°
Diffractometer control software	BRUKER APEX3(v2019.1-0)
Diffractometer measurement device	Bruker D8 KAPPA II (APEX II detector)
Diffractometer measurement method	Data collection strategy APEX 3/Queen
θ range for data collection	3.017°- 33.297°
Completeness to θ = 25.242°	99.8%
Completeness to θ_{max} = 33.297°	99.8%
Index ranges	$-44 \leq h \leq 44$
	$-6 \leq k \leq 6$
	$-28 \leq l \leq 28$
Computing data reduction	BRUKER APEX3(v2019.1-0)
Absorption coefficient	2.935 mm^{-1}
Absorption correction	Semi-empirical from equivalents
Computation absorption correction	SADABS
Max./min. Transmission	0.75/0.60
R_{merg} before/after correction	0.0742/0.0463
Computing structure solution	BRUKER APEX3(v2019.1-0)
Computing structure refinement	SHELXL-2017/1 (Sheldrick, 2017)
Refinement method	Full-matrix least-squares on F^2
Reflections collected	40339
Independent reflections	4161
R_{int}	0.0384
Reflections with $I > 2\sigma(I)$	3675
Restraints	0
Parameter	136
GooF	1.120
Weighting details	$w = 1/[\sigma^2(F_{obs}^2) + (0.0289P)^2 + 4.7952P]$

	where P = (F_{obs}^2 + 2F_{calc}^2)/3
R_1 [$I > 2\sigma(I)$]	0.0285
wR_2 [$I > 2\sigma(I)$]	0.0615
R_1 [all data]	0.0354
wR_2 [all data]	0.0641
Largest diff. peak and hole	1.569/-1.114

Comments

Treatment of hydrogen atoms

Riding model on idealized geometries with the 1.2 fold isotropic displacement parameters of the equivalent U_{ij} of the corresponding carbon atom.

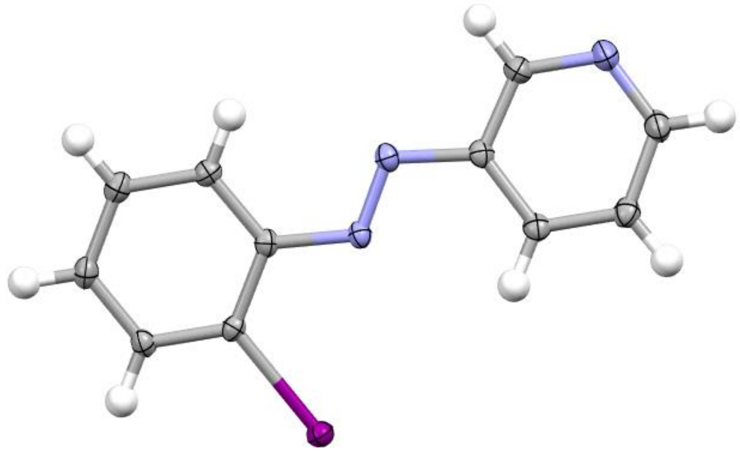

Figure S74. Molecular structure of **6f** with thermal ellipsoids at 50% probability level.

Crystal Structure Data of 3a

Table S5. Crystal structure data and structure refinement for **3a**.

Deposition number	2444464
Empirical formula	$C_{18}H_{12d}r_2N_2Te$
Formula weight	545.73
Density (calculated)	1.992 g·cm^{-3}
F(000)	1032
Temperature	200(2) K
Crystal size	0.207 × 0.193 × 0.041 mm
Crystal color	orange
Crystal description	tablet
Wavelength	0.71073 Å
Crystal system	monoclinic
Space group	*Pc*
Unit cell dimensions	
a [Å]	19.490(2)
b [Å]	11.5255(12)
c [Å]	8.1027(9)
α [°]	90
β [°]	90.022(6)
γ [°]	90
Volume	1820.1(3) Å^3
Z	4
Cell measurement reflections used	9947
Cell measurement θ min/max	2.74°/31.63°
Diffractometer control software	BRUKER APEX3(v2019.1-0)
Diffractometer measurement device	Bruker D8 KAPPA II (APEX II detector)
Diffractometer measurement method	Data collection strategy APEX 3/Queen
θ range for data collection	1.767°- 33.161°
Completeness to θ = 25.242°	99.9%
Completeness to θ_{max} = 33.161°	99.8%
Index ranges	$-29 \leq h \leq 29$
	$-17 \leq k \leq 17$
	$-12 \leq l \leq 12$
Computing data reduction	BRUKER APEX3(v2019.1-0)
Absorption coefficient	6.025 mm^{-1}
Absorption correction	Semi-empirical from equivalents
Computation absorption correction	SADABS
Max./min. Transmission	0.75/0.54
R_{merg} before/after correction	0.0954/0.0538
Computing structure solution	BRUKER APEX3(v2019.1-0)
Computing structure refinement	SHELXL-2017/1 (Sheldrick, 2017)
Refinement method	Full-matrix least-squares on F^2
Reflections collected	36261
Independent reflections	10416
R_{int}	0.0472
Reflections with $I > 2\sigma(I)$	8733

Restraints	4220
Parameter	628
GooF	1.031
Weighting details	$w = 1/[\sigma^2(F_{obs}^2) + (0.0823P)^2 + 3.2422P]$ where $P = (F_{obs}^2 + 2F_{calc}^2)/3$
R_1 [$I > 2\sigma(I)$]	0.0530
wR_2 [$I > 2\sigma(I)$]	0.1268
R_1 [all data]	0.0713
wR_2 [all data]	0.1396
Absolute structure parameter	see below
Largest diff. peak and hole	2.149/-4.319

Comments

Treatment of hydrogen atoms

Riding model on idealized geometries with the 1.2 fold isotropic displacement parameters of the equivalent U_{ij} of the corresponding carbon atom. The methyl groups are idealized with tetrahedral angles in a combined rotating and rigid group refinement with the 1.5 fold isotropic displacement parameters of the equivalent U_{ij} of the corresponding carbon atom.

Twinning

The crystal is pseudo-orthorhombically twinned by two-fold rotation about *a*. In addition, twinning by inversion was included leading to four twin components in total. The combination of pseudo-merohedral and inversion twinning does not allow a Flack-Parameter to be determined.

Disorder

One of the molecules shows a full-body disorder. One orientation approximately matches $Pna2_1$ symmetry the other $Pca2_1$. The structure can be solved in both space groups but a refinement fails thus *Pc* - the subgroup common to both - and the pseudo-merohedral twinning model was chosen. Changing the measurement temperature to 100 K does not resolve the disorder. In the end, the data set that lead to the most convincing refinement was chosen. Different measurements at 100 K suggest that the scale factors for the twin components differ from specimen to specimen. The bond lengths and angles of all phenyl rings were restrained to be equal (SADI) and the ring was restrained to planarity (FLAT). Global RIGU and SIMU restraints were used for the displacement parameters to reduce correlations. Considering twinning and disorder quantitative results may be unreliable and should be carefully scrutinized and if possible confirmed by other means.

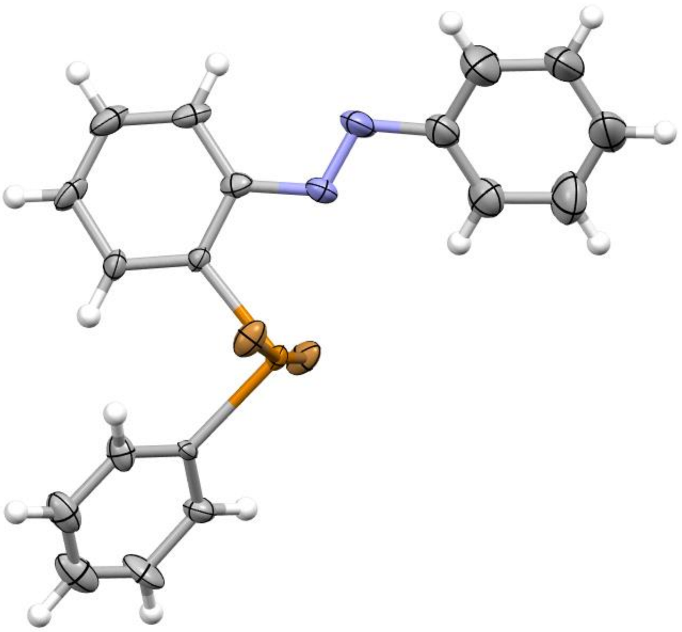

Figure S75. Molecular structure of **3a** with thermal ellipsoids at 50% probability level.

Crystal Structure Data of 2b

Table S5. Crystal structure data and structure refinement for **2b**.

Deposition number	2444459
Empirical formula	$C_{16}H_{13d}l_2N_4Te$
Formula weight	460.81
Density (calculated)	1.782 g·cm^{-3}
F(000)	896
Temperature	100(2) K
Crystal size	0.354 × 0.206 × 0.018 mm
Crystal color	yellow
Crystal description	plate
Wavelength	1.54178 Å
Crystal system	monoclinic
Space group	$P2_1/c$
Unit cell dimensions	
a [Å]	20.159(2)
b [Å]	11.0278(11)
c [Å]	7.8652(8)
α [°]	90
β [°]	100.850(4)
γ [°]	90
Volume	1717.3(3) Å^3
Z	4
Cell measurement reflections used	9016
Cell measurement θ min/max	4.59°/80.17°
Diffractometer control software	Bruker APEX3(v2017.3-0)
Diffractometer measurement device	Bruker D8 Venture (Photon II detector)
Diffractometer measurement method	Data collection strategy APEX 3/Queen
θ range for data collection	2.231°- 80.263°
Completeness to θ = 67.679°	100.0%
Completeness to θ_{max} = 80.263°	98.7%
Index ranges	$-25 \leq h \leq 25$<br>$-14 \leq k \leq 13$<br>$-9 \leq l \leq 10$
Computing data reduction	Bruker APEX3(v2017.3-0)
Absorption coefficient	16.566 mm^{-1}
Absorption correction	Numerical
Computation absorption correction	SADABS
Max./min. Transmission	0.21/0.03
R_{merg} before/after correction	0.1462/0.1040
Computing structure solution	Bruker APEX3(v2017.3-0)
Computing structure refinement	SHELXL-2017/1 (Sheldrick, 2017)
Refinement method	Full-matrix least-squares on F^2
Reflections collected	61323
Independent reflections	3716
R_{int}	0.0771
Reflections with $I > 2\sigma(I)$	3447
Restraints	0
Parameter	209
GooF	1.108
Weighting details	$w = 1/[\sigma^2(F_{obs}{}^2) + (0.0126P)^2 + 9.8014P]$

	where P = $(F_{obs}^2 + 2F_{calc}^2)/3$
R_1 [$I > 2\sigma(I)$]	0.0366
wR_2 [$I > 2\sigma(I)$]	0.0883
R_1 [all data]	0.0396
wR_2 [all data]	0.0901
Largest diff. peak and hole	1.338/-0.900

Comments

Treatment of hydrogen atoms

Riding model on idealized geometries with the 1.2 fold isotropic displacement parameters of the equivalent U_{ij} of the corresponding carbon atom. The methyl groups are idealized with tetrahedral angles in a combined rotating and rigid group refinement with the 1.5 fold isotropic displacement parameters of the equivalent U_{ij} of the corresponding carbon atom.

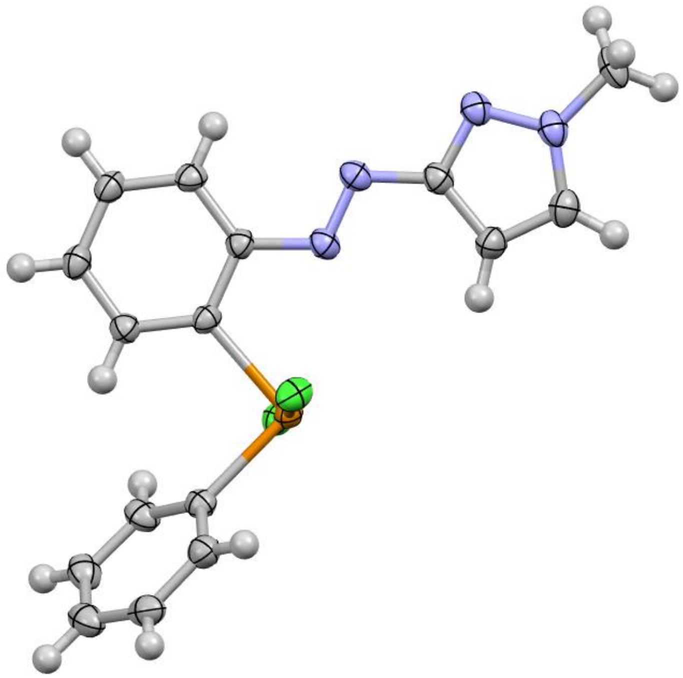

Figure S76. Molecular structure of **2b** with thermal ellipsoids at 50% probability level.

Crystal Structure Data of 1c

Table S6. Crystal structure data and structure refinement for **1c**.

Deposition number	2444456
Empirical formula	$C_{17}H_{15}N_3Te$
Formula weight	388.92
Density (calculated)	1.652 g·cm^{-3}
F(000)	380
Temperature	100(2) K
Crystal size	0.399 × 0.311 × 0.302 mm
Crystal color	red
Crystal description	block
Wavelength	0.71073 Å
Crystal system	triclinic
Space group	$P\bar{1}$
Unit cell dimensions	
a [Å]	7.8417(12)
b [Å]	9.5534(15)
c [Å]	11.4733(18)
α [°]	77.546(5)
β [°]	83.869(7)
γ [°]	68.791(5)
Volume	782.1(2) Å^3
Z	2
Cell measurement reflections used	9540
Cell measurement θ min/max	3.40°/40.60°
Diffractometer control software	BRUKER APEX3(v2019.1-0)
Diffractometer measurement device	Bruker D8 KAPPA II (APEX II detector)
Diffractometer measurement method	Data collection strategy APEX 3/Queen
θ range for data collection	2.330°- 40.443°
Completeness to θ = 25.242°	99.7%
Completeness to θ_{max} = 40.443°	99.1%
Index ranges	$-14 \leq h \leq 14$<br>$-17 \leq k \leq 17$<br>$-20 \leq l \leq 20$
Computing data reduction	BRUKER APEX3(v2019.1-0)
Absorption coefficient	1.898 mm^{-1}
Absorption correction	Semi-empirical from equivalents
Computation absorption correction	SADABS
Max./min. Transmission	0.75/0.59
R_{merg} before/after correction	0.0543/0.0279
Computing structure solution	BRUKER APEX3(v2019.1-0)
Computing structure refinement	SHELXL-2017/1 (Sheldrick, 2017)
Refinement method	Full-matrix least-squares on F^2
Reflections collected	113309
Independent reflections	9879
R_{int}	0.0167
Reflections with $I > 2\sigma(I)$	9741
Restraints	0
Parameter	191
GooF	1.241
Weighting details	$w = 1/[\sigma^2(F_{obs}^2) + (0.0152P)^2 + 0.1804P]$

	where P = $(F_{obs}^2 + 2F_{calc}^2)/3$
R_1 [$I > 2\sigma(I)$]	0.0127
wR_2 [$I > 2\sigma(I)$]	0.0354
R_1 [all data]	0.0130
wR_2 [all data]	0.0355
Largest diff. peak and hole	0.861/-0.456

Comments

Treatment of hydrogen atoms

Riding model on idealized geometries with the 1.2 fold isotropic displacement parameters of the equivalent U_{ij} of the corresponding carbon atom. The methyl groups are idealized with tetrahedral angles in a combined rotating and rigid group refinement with the 1.5 fold isotropic displacement parameters of the equivalent U_{ij} of the corresponding carbon atom.

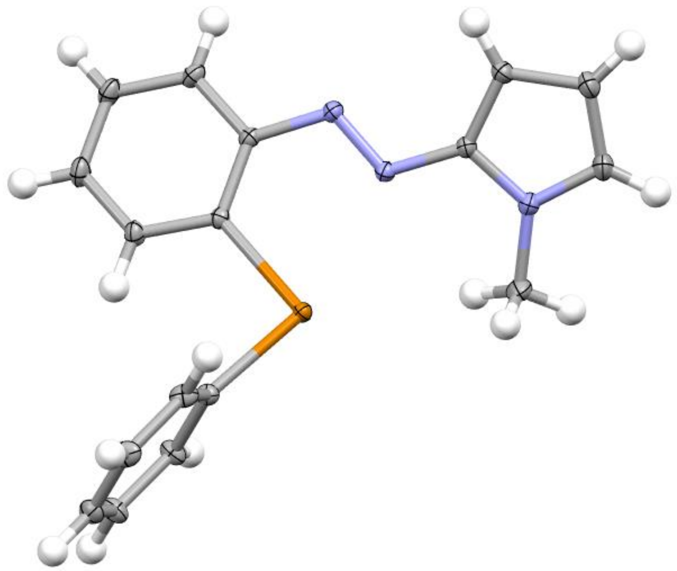

Figure S77. Molecular structure of **1c** with thermal ellipsoids at 50% probability level.

Crystal Structure Data of 2c

Table S7. Crystal structure data and structure refinement for **2c**.

Deposition number	2444460
Empirical formula	$C_{17}H_{13e}I_2N_3Te$
Formula weight	459.82
Density (calculated)	1.746 g·cm^{-3}
F(000)	896
Temperature	100(2) K
Crystal size	0.508 × 0.302 × 0.291 mm
Crystal color	brownish red
Crystal description	block
Wavelength	0.71073 Å
Crystal system	monoclinic
Space group	$P2_1/n$
Unit cell dimensions	
a [Å]	10.1825(3)
b [Å]	13.3056(4)
c [Å]	12.9261(4)
α [°]	90
β [°]	93.0528(9)
γ [°]	90
Volume	1748.80(9) Å^3
Z	4
Cell measurement reflections used	9984
Cell measurement θ min/max	2.20°/40.45°
Diffractometer control software	BRUKER APEX3(v2019.1-0)
Diffractometer measurement device	Bruker D8 KAPPA II (APEX II detector)
Diffractometer measurement method	Data collection strategy APEX 3/Queen
θ range for data collection	2.198°- 40.473°
Completeness to θ = 25.242°	99.8%
Completeness to θ_{max} = 40.473°	99.8%
Index ranges	$-18 \leq h \leq 18$
	$-24 \leq k \leq 24$
	$-23 \leq l \leq 23$
Computing data reduction	BRUKER APEX3(v2019.1-0)
Absorption coefficient	2.008 mm^{-1}
Absorption correction	Semi-empirical from equivalents
Computation absorption correction	SADABS
Max./min. Transmission	0.75/0.51
R_{merg} before/after correction	0.0880/0.0357
Computing structure solution	BRUKER APEX3(v2019.1-0)
Computing structure refinement	SHELXL-2017/1 (Sheldrick, 2017)
Refinement method	Full-matrix least-squares on F^2
Reflections collected	118616
Independent reflections	11137
R_{int}	0.0203
Reflections with $I > 2\sigma(I)$	10379
Restraints	0
Parameter	209
GooF	1.147
Weighting details	$w = 1/[\sigma^2(F_{obs}^2) + (0.0172P)^2 + 0.9401P]$

	where P = $(F_{obs}^2 + 2F_{calc}^2)/3$
R_1 [$I > 2\sigma(I)$]	0.0186
wR_2 [$I > 2\sigma(I)$]	0.0450
R_1 [all data]	0.0213
wR_2 [all data]	0.0469
Largest diff. peak and hole	0.891/-0.619

Comments

Treatment of hydrogen atoms

Riding model on idealized geometries with the 1.2 fold isotropic displacement parameters of the equivalent U_{ij} of the corresponding carbon atom. The methyl groups are idealized with tetrahedral angles in a combined rotating and rigid group refinement with the 1.5 fold isotropic displacement parameters of the equivalent U_{ij} of the corresponding carbon atom.

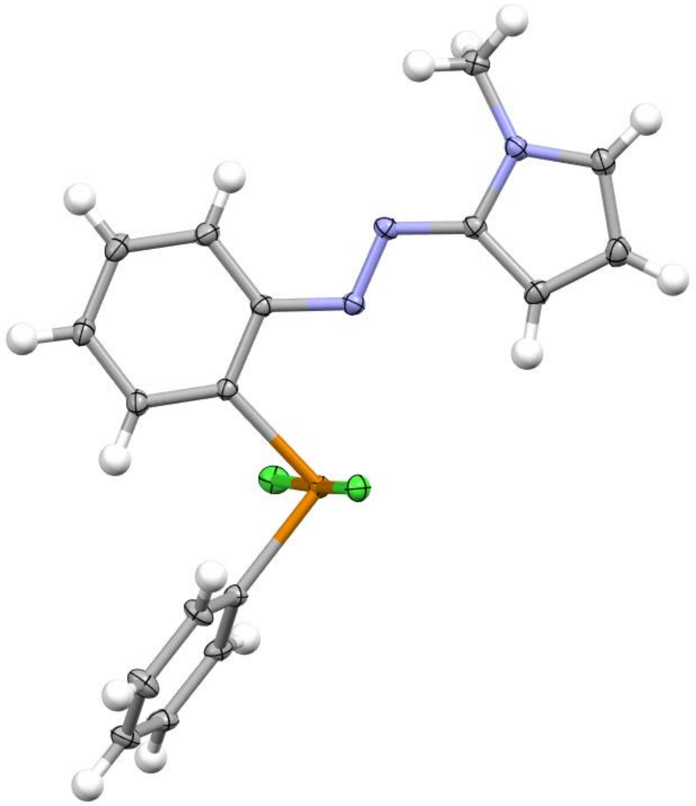

Figure S78. Molecular structure of **2c** with thermal ellipsoids at 50% probability level.

Crystal Structure Data of 3c

Table S8. Crystal structure data and structure refinement for **3c**.

Deposition number	2444465
Empirical formula	$C_{17}H_{15}Br_2N_3Te$
Formula weight	548.74
Density (calculated)	2.027 g·cm^{-3}
F(000)	1040
Temperature	100(2) K
Crystal size	0.244 × 0.146 × 0.065 mm
Crystal color	orange
Crystal description	tablet
Wavelength	1.54178 Å
Crystal system	monoclinic
Space group	$P2_1/n$
Unit cell dimensions	
a [Å]	13.6351(10)
b [Å]	8.9188(7)
c [Å]	14.9851(11)
α [°]	90
β [°]	99.2697(19)
γ [°]	90
Volume	1798.5(2) Å^3
Z	4
Cell measurement reflections used	9319
Cell measurement *θ* min/max	2.99°/79.91°
Diffractometer control software	Bruker APEX3(v2017.3-0)
Diffractometer measurement device	Bruker D8 Venture (Photon II detector)
Diffractometer measurement method	Data collection strategy APEX 3/Queen
θ range for data collection	4.070°- 80.410°
Completeness to *θ* = 67.679°	100.0%
Completeness to θ_{max} = 80.410°	99.6%
Index ranges	$-17 \leq h \leq 17$
	$-11 \leq k \leq 9$
	$-19 \leq l \leq 19$
Computing data reduction	Bruker APEX3(v2017.3-0)
Absorption coefficient	18.272 mm^{-1}
Absorption correction	Numerical
Computation absorption correction	SADABS
Max./min. Transmission	0.12/0.02
R_{merg} before/after correction	0.1406/0.1007
Computing structure solution	Bruker APEX3(v2017.3-0)
Computing structure refinement	SHELXL-2017/1 (Sheldrick, 2017)
Refinement method	Full-matrix least-squares on F^2
Reflections collected	78450
Independent reflections	3932
R_{int}	0.0634
Reflections with $I > 2\sigma(I)$	3923

Restraints	0
Parameter	209
GooF	1.094
Weighting details	$w = 1/[\sigma^2(F_{obs}^2) + (0.0574P)^2 + 3.5603P]$ where $P = (F_{obs}^2 + 2F_{calc}^2)/3$
R_1 [$I > 2\sigma(I)$]	0.0338
wR_2 [$I > 2\sigma(I)$]	0.0961
R_1 [all data]	0.0338
wR_2 [all data]	0.0961
Largest diff. peak and hole	1.632/-1.241

Comments

Treatment of hydrogen atoms

Riding model on idealized geometries with the 1.2 fold isotropic displacement parameters of the equivalent U_{ij} of the corresponding carbon atom. The methyl groups are idealized with tetrahedral angles in a combined rotating and rigid group refinement with the 1.5 fold isotropic displacement parameters of the equivalent U_{ij} of the corresponding carbon atom.

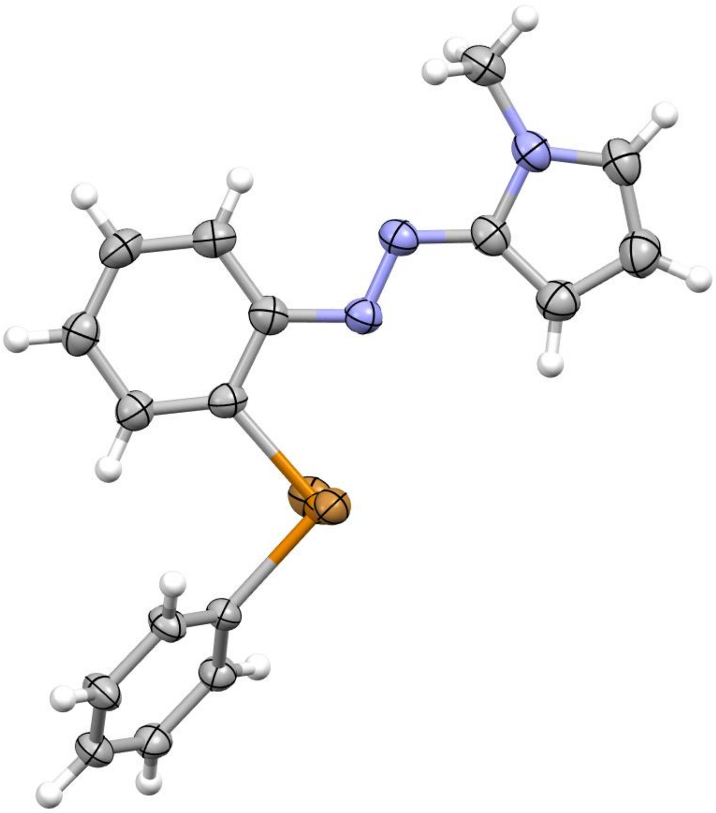

Figure S79. Molecular structure of **3c** with thermal ellipsoids at 50% probability level.

Crystal Structure Data of 1d

Table S9. Crystal structure data and structure refinement for **1d**.

Deposition number	2444457
Empirical formula	$C_{17.72}H_{17.77}I_{0.05}N_4Te_{0.95}$
Formula weight	414.39
Density (calculated)	1.617 g·cm^{-3}
F(000)	817
Temperature	100(2) K
Crystal size	0.324 × 0.150 × 0.122 mm
Crystal color	orange
Crystal description	block
Wavelength	0.71073 Å
Crystal system	monoclinic
Space group	$P2_1/c$
Unit cell dimensions	
a [Å]	8.467(2)
b [Å]	20.680(5)
c [Å]	10.558(3)
α [°]	90
β [°]	112.921(4)
γ [°]	90
Volume	1702.7(7) Å^3
Z	4
Cell measurement reflections used	9822
Cell measurement θ min/max	2.31°/36.52°
Diffractometer control software	BRUKER APEX3(v2019.1-0)
Diffractometer measurement device	Bruker D8 KAPPA II (APEX II detector)
Diffractometer measurement method	Data collection strategy APEX 3/Queen
θ range for data collection	1.970°- 36.530°
Completeness to θ = 25.242°	99.9%
Completeness to θ_{max} = 36.530°	99.8%
Index ranges	$-14 \leq h \leq 14$
	$-34 \leq k \leq 34$
	$-17 \leq l \leq 17$
Computing data reduction	BRUKER APEX3(v2019.1-0)
Absorption coefficient	1.757 mm^{-1}
Absorption correction	Numerical
Computation absorption correction	SADABS
Max./min. Transmission	0.31/0.24
R_{merg} before/after correction	0.0520/0.0477
Computing structure solution	BRUKER APEX3(v2019.1-0)
Computing structure refinement	SHELXL-2017/1 (Sheldrick, 2017)
Refinement method	Full-matrix least-squares on F^2
Reflections collected	78828
Independent reflections	8364
R_{int}	0.0353
Reflections with $I > 2\sigma(I)$	7853

Restraints	99
Parameter	281
GooF	1.292
Weighting details	$w = 1/[\sigma^2(F_{obs}^2) + (0.0161P)^2 + 1.1738P]$ where $P = (F_{obs}^2 + 2F_{calc}^2)/3$
R_1 [$I > 2\sigma(I)$]	0.0241
wR_2 [$I > 2\sigma(I)$]	0.0613
R_1 [all data]	0.0263
wR_2 [all data]	0.0620
Largest diff. peak and hole	0.747/-0.966

Comments

Treatment of hydrogen atoms

Riding model on idealized geometries with the 1.2 fold isotropic displacement parameters of the equivalent U_{ij} of the corresponding carbon atom. The methyl groups are idealized with tetrahedral angles in a combined rotating and rigid group refinement with the 1.5 fold isotropic displacement parameters of the equivalent U_{ij} of the corresponding carbon atom.

Disorder

The crystal contains about 5% of the starting material. The five-membered ring of both molecules coincides. The displacement parameters of the partially occupied sites of the starting material were refined with RIGU and SIMU restraints. Its phenyl ring was constrained to a regular hexagon of 1.39 Å edge length (AFIX 66).

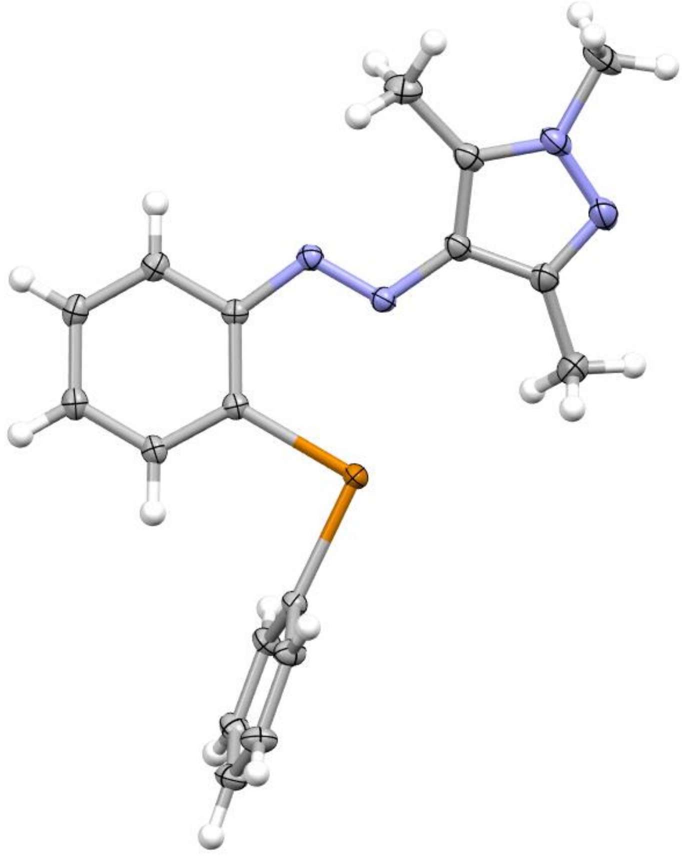

Figure S80. Molecular structure of **1d** with thermal ellipsoids at 50% probability level.

Crystal Structure Data of 2d

Table S10. Crystal structure data and structure refinement for **2d**.

Deposition number	2444461
Empirical formula	$C_{18}H_{18}Cl_2N_4Te$
Formula weight	488.86
Density (calculated)	1.733 g·cm^{-3}
F(000)	1920
Temperature	100(2) K
Crystal size	0.103 × 0.081 × 0.056 mm
Crystal color	yellow
Crystal description	tablet
Wavelength	1.54178 Å
Crystal system	monoclinic
Space group	*C*2/*c*
Unit cell dimensions	
a [Å]	15.0381(7)
b [Å]	8.2865(4)
c [Å]	30.2366(14)
α [°]	90
β [°]	96.1125(15)
γ [°]	90
Volume	3746.5(3) Å^3
Z	8
Cell measurement reflections used	9985
Cell measurement θ min/max	2.94°/80.29°
Diffractometer control software	Bruker APEX3(v2017.3-0)
Diffractometer measurement device	Bruker D8 Venture (Photon II detector)
Diffractometer measurement method	Data collection strategy APEX 3/Queen
θ range for data collection	2.940°- 80.292°
Completeness to θ = 67.679°	100.0%
Completeness to θ_{max} = 80.292°	99.7%
Index ranges	$-18 \leq h \leq 19$
	$-10 \leq k \leq 10$
	$-38 \leq l \leq 38$
Computing data reduction	Bruker APEX3(v2017.3-0)
Absorption coefficient	15.225 mm^{-1}
Absorption correction	Numerical
Computation absorption correction	SADABS
Max./min. Transmission	0.21/0.08
R_{merg} before/after correction	0.1332/0.0868
Computing structure solution	Bruker APEX3(v2017.3-0)
Computing structure refinement	SHELXL-2017/1 (Sheldrick, 2017)
Refinement method	Full-matrix least-squares on F^2
Reflections collected	77739
Independent reflections	4082
R_{int}	0.0669
Reflections with $I > 2\sigma(I)$	3967

Restraints	0
Parameter	229
GooF	1.021
Weighting details	$w = 1/[\sigma^2(F_{obs}^2) + (0.0374P)^2 + 4.4801P]$ where $P = (F_{obs}^2 + 2F_{calc}^2)/3$
R_1 [$I > 2\sigma(I)$]	0.0215
wR_2 [$I > 2\sigma(I)$]	0.0578
R_1 [all data]	0.0222
wR_2 [all data]	0.0585
Largest diff. peak and hole	0.863/-0.671

Comments

Treatment of hydrogen atoms

Riding model on idealized geometries with the 1.2 fold isotropic displacement parameters of the equivalent U_{ij} of the corresponding carbon atom. The methyl groups are idealized with tetrahedral angles in a combined rotating and rigid group refinement with the 1.5 fold isotropic displacement parameters of the equivalent U_{ij} of the corresponding carbon atom.

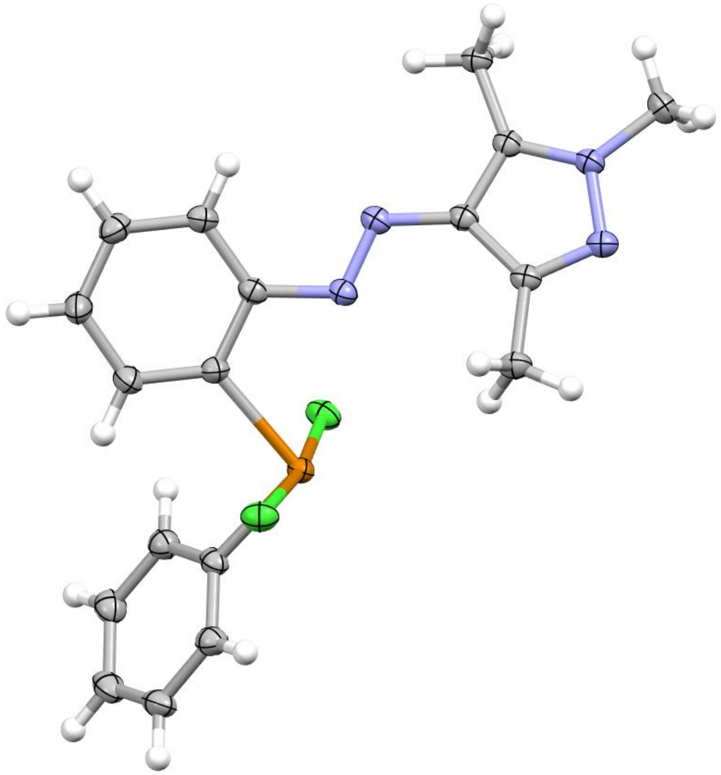

Figure S81. Molecular structure of **2d** with thermal ellipsoids at 50% probability level.

Crystal Structure Data of 3d

Table S11. Crystal structure data and structure refinement for **3d**.

Deposition number	2444466
Empirical formula	$C_{18}H_{18}Br_2N_4Te$
Formula weight	577.78
Density (calculated)	1.964 g·cm^{-3}
F(000)	2208
Temperature	100(2) K
Crystal size	0.109 × 0.078 × 0.041 mm
Crystal color	yellow
Crystal description	tablet
Wavelength	1.54178 Å
Crystal system	monoclinic
Space group	*C*2/*c*
Unit cell dimensions	
a [Å]	15.0844(7)
b [Å]	8.5581(4)
c [Å]	30.4578(14)
α [°]	90
β [°]	96.3142(15)
γ [°]	90
Volume	3908.1(3) Å^3
Z	8
Cell measurement reflections used	9671
Cell measurement θ min/max	2.92°/79.53°
Diffractometer control software	Bruker APEX3(v2017.3-0)
Diffractometer measurement device	Bruker D8 Venture (Photon II detector)
Diffractometer measurement method	Data collection strategy APEX 3/Queen
θ range for data collection	2.919°- 79.893°
Completeness to θ = 67.679°	99.8%
Completeness to θ_{max} = 79.893°	98.8%
Index ranges	$-19 \leq h \leq 17$
	$0 \leq k \leq 10$
	$0 \leq l \leq 38$
Computing data reduction	Bruker APEX3(v2017.3-0)
Absorption coefficient	16.872 mm^{-1}
Absorption correction	Semi-empirical from equivalents
Computation absorption correction	TWINABS
Max./min. Transmission	0.33/0.10
R_{merg} before/after correction	0.1420/0.0829 and 0.1858/0.0895
Computing structure solution	Bruker APEX3(v2017.3-0)
Computing structure refinement	SHELXL-2017/1 (Sheldrick, 2017)
Refinement method	Full-matrix least-squares on F^2
Reflections collected	118402
Independent reflections	4192
R_{int}	0.0733
Reflections with $I > 2\sigma(I)$	4101

Restraints	0
Parameter	229
GooF	1.169
Weighting details	$w = 1/[\sigma^2(F_{obs}^2) + (0.0619P)^2 + 17.7911P]$ where $P = (F_{obs}^2 + 2F_{calc}^2)/3$
R_1 [$I > 2\sigma(I)$]	0.0334
wR_2 [$I > 2\sigma(I)$]	0.0986
R_1 [all data]	0.0345
wR_2 [all data]	0.1035
Largest diff. peak and hole	1.399/-0.880

Comments

Treatment of hydrogen atoms

Riding model on idealized geometries with the 1.2 fold isotropic displacement parameters of the equivalent U_{ij} of the corresponding carbon atom. The methyl groups are idealized with tetrahedral angles in a combined rotating and rigid group refinement with the 1.5 fold isotropic displacement parameters of the equivalent U_{ij} of the corresponding carbon atom.

Twinning

The crystal was a non-merohedral twin and the model refined against de-twinned hklf4 data.

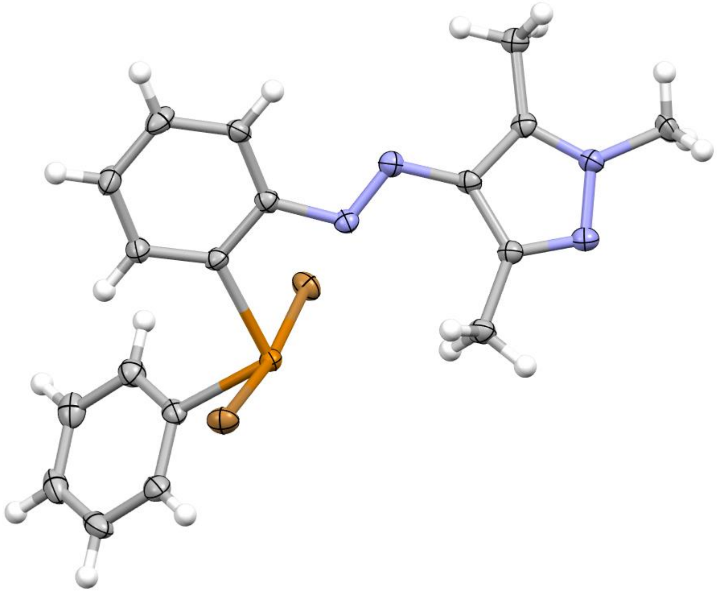

Figure S82. Molecular structure of **3d** with thermal ellipsoids at 50% probability level.

Crystal Structure Data of 1e

Table S12. Crystal structure data and structure refinement for **1e**.

Deposition number	2444458
Empirical formula	$C_{20}H_{18}N_2O_2Te$
Formula weight	445.96
Density (calculated)	1.635 g·cm^{-3}
F(000)	880
Temperature	100(2) K
Crystal size	0.240 × 0.142 × 0.028 mm
Crystal color	orange
Crystal description	plate
Wavelength	1.54178 Å
Crystal system	monoclinic
Space group	$P2_1/c$
Unit cell dimensions	
a [Å]	8.3421(8)
b [Å]	8.1143(8)
c [Å]	26.762(3)
α [°]	90
β [°]	90.181(4)
γ [°]	90
Volume	1811.5(3) Å^3
Z	4
Cell measurement reflections used	9661
Cell measurement *θ* min/max	3.30°/79.07°
Diffractometer control software	Bruker APEX3(v2017.3-0)
Diffractometer measurement device	Bruker D8 Venture (Photon II detector)
Diffractometer measurement method	Data collection strategy APEX 3/Queen
θ range for data collection	3.303°- 80.058°
Completeness to *θ* = 67.679°	99.9%
Completeness to θ_{max} = 80.058°	98.0%
Index ranges	$-10 \leq h \leq 10$
	$-8 \leq k \leq 9$
	$-34 \leq l \leq 33$
Computing data reduction	Bruker APEX3(v2017.3-0)
Absorption coefficient	13.086 mm^{-1}
Absorption correction	Numerical
Computation absorption correction	SADABS
Max./min. Transmission	0.20/0.03
R_{merg} before/after correction	0.1347/0.0967
Computing structure solution	Bruker APEX3(v2017.3-0)
Computing structure refinement	SHELXL-2017/1 (Sheldrick, 2017)
Refinement method	Full-matrix least-squares on F^2
Reflections collected	78605
Independent reflections	3886
R_{int}	0.0735
Reflections with $I > 2\sigma(I)$	3732
Restraints	0
Parameter	228
GooF	1.323
Weighting details	$w = 1/[\sigma^2(F_{obs}^2) + 10.1396P]$

	where P = $(F_{obs}^2 + 2F_{calc}^2)/3$
R_1 [$I > 2\sigma(I)$]	0.0420
wR_2 [$I > 2\sigma(I)$]	0.1036
R_1 [all data]	0.0433
wR_2 [all data]	0.1042
Largest diff. peak and hole	1.135/-1.144

Comments

Treatment of hydrogen atoms

Riding model on idealized geometries with the 1.2 fold isotropic displacement parameters of the equivalent U_{ij} of the corresponding carbon atom. The methyl groups are idealized with tetrahedral angles in a combined rotating and rigid group refinement with the 1.5 fold isotropic displacement parameters of the equivalent U_{ij} of the corresponding carbon atom.

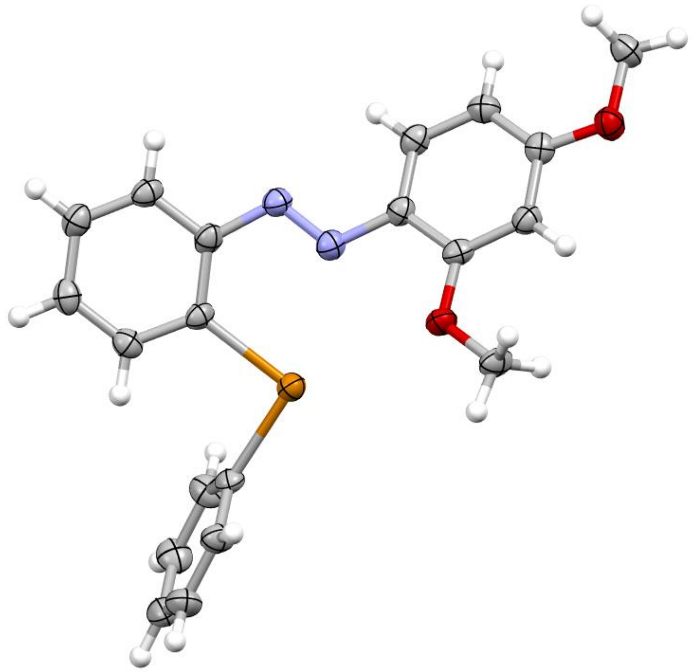

Figure S83. Molecular structure of **1e** with thermal ellipsoids at 50% probability level.

Crystal Structure Data of 2e

Table S13. Crystal structure data and structure refinement for **2e**.

Deposition number	2444462
Empirical formula	$C_{20}H_{18}Cl_2N_2O_2Te$
Formula weight	516.86
Density (calculated)	1.733 g·cm^{-3}
F(000)	2032
Temperature	100(2) K
Crystal size	0.258 × 0.149 × 0.020 mm
Crystal color	orange
Crystal description	plate
Wavelength	1.54178 Å
Crystal system	monoclinic
Space group	*C*2/*c*
Unit cell dimensions	
a [Å]	14.5245(15)
b [Å]	8.8145(9)
c [Å]	30.951(3)
α [°]	90
β [°]	90.381(2)
γ [°]	90
Volume	3962.5(7) Å^3
Z	8
Cell measurement reflections used	9717
Cell measurement θ min/max	2.86°/79.37°
Diffractometer control software	Bruker APEX3(v2017.3-0)
Diffractometer measurement device	Bruker D8 Venture (Photon II detector)
Diffractometer measurement method	Data collection strategy APEX 3/Queen
θ range for data collection	2.855°- 79.473°
Completeness to θ = 67.679°	96.8%
Completeness to θ_{max} = 79.473°	96.6%
Index ranges	$-18 \leq h \leq 18$
	$-10 \leq k \leq 11$
	$-39 \leq l \leq 39$
Computing data reduction	Bruker APEX3(v2017.3-0)
Absorption coefficient	14.484 mm^{-1}
Absorption correction	Semi-empirical from equivalents
Computation absorption correction	SADABS
Max./min. Transmission	0.75/0.26
R_{merg} before/after correction	0.1497/0.0744
Computing structure solution	Bruker APEX3(v2017.3-0)
Computing structure refinement	SHELXL-2017/1 (Sheldrick, 2017)
Refinement method	Full-matrix least-squares on F^2
Reflections collected	81604
Independent reflections	4150
R_{int}	0.0471
Reflections with $I > 2\sigma(I)$	4119
Restraints	0
Parameter	246
GooF	1.145
Weighting details	$w = 1/[\sigma^2(F_{obs}^2) + (0.0283P)^2 + 9.8579P]$

	where P = $(F_{obs}^2 + 2F_{calc}^2)/3$
R_1 [$I > 2\sigma(I)$]	0.0231
wR_2 [$I > 2\sigma(I)$]	0.0619
R_1 [all data]	0.0233
wR_2 [all data]	0.0620
Largest diff. peak and hole	0.934/-0.567

Comments

Treatment of hydrogen atoms

Riding model on idealized geometries with the 1.2 fold isotropic displacement parameters of the equivalent U_{ij} of the corresponding carbon atom. The methyl groups are idealized with tetrahedral angles in a combined rotating and rigid group refinement with the 1.5 fold isotropic displacement parameters of the equivalent U_{ij} of the corresponding carbon atom.

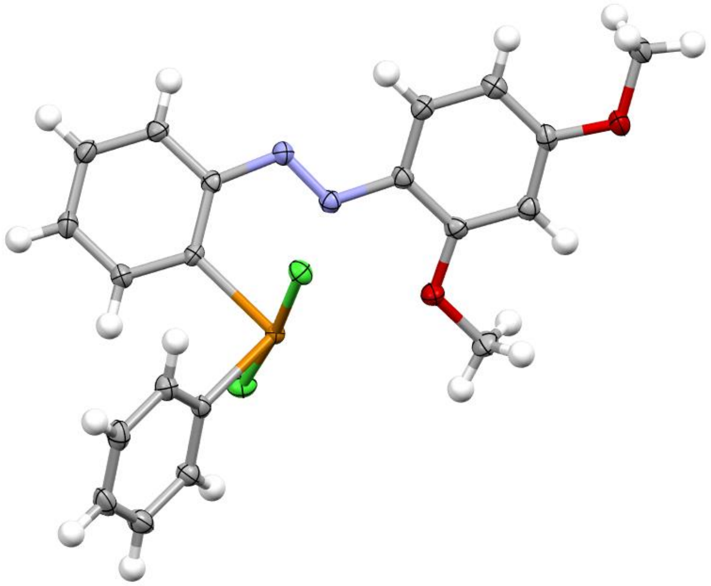

Figure S84. Molecular structure of **2e** with thermal ellipsoids at 50% probability level.

Crystal Structure Data of 3e

Table S14. Crystal structure data and structure refinement for **3e**.

Deposition number	2444467
Empirical formula	$C_{20}H_{18}Br_2N_2O_2Te$
Formula weight	605.78
Density (calculated)	1.957 g·cm^{-3}
F(000)	2320
Temperature	100(2) K
Crystal size	0.360 × 0.074 × 0.035 mm
Crystal color	orange
Crystal description	plate
Wavelength	0.71073 Å
Crystal system	monoclinic
Space group	*C*2/*c*
Unit cell dimensions	
a [Å]	14.9179(15)
b [Å]	8.9025(10)
c [Å]	30.980(3)
α [°]	90
β [°]	92.030(3)
γ [°]	90
Volume	4111.8(7) Å^3
Z	8
Cell measurement reflections used	9964
Cell measurement θ min/max	2.66°/32.74°
Diffractometer control software	BRUKER APEX3(v2019.1-0)
Diffractometer measurement device	Bruker D8 KAPPA II (APEX II detector)
Diffractometer measurement method	Data collection strategy APEX 3/Queen
θ range for data collection	2.631°- 33.346°
Completeness to θ = 25.242°	99.9%
Completeness to θ_{max} = 33.346°	99.9%
Index ranges	$-23 \leq h \leq 22$
	$-13 \leq k \leq 13$
	$-47 \leq l \leq 47$
Computing data reduction	BRUKER APEX3(v2019.1-0)
Absorption coefficient	5.352 mm^{-1}
Absorption correction	Semi-empirical from equivalents
Computation absorption correction	SADABS
Max./min. Transmission	0.75/0.55
R_{merg} before/after correction	0.0974/0.0581
Computing structure solution	BRUKER APEX3(v2019.1-0)
Computing structure refinement	SHELXL-2017/1 (Sheldrick, 2017)
Refinement method	Full-matrix least-squares on F^2
Reflections collected	87937
Independent reflections	7975
R_{int}	0.0632
Reflections with $I > 2\sigma(I)$	6647
Restraints	0
Parameter	246
GooF	1.113
Weighting details	$w = 1/[\sigma^2(F_{obs}^2) + (0.0187P)^2 + 10.8674P]$

	where P = $(F_{obs}^2 + 2F_{calc}^2)/3$
R_1 [$I > 2\sigma(I)$]	0.0322
wR_2 [$I > 2\sigma(I)$]	0.0579
R_1 [all data]	0.0454
wR_2 [all data]	0.0611
Largest diff. peak and hole	1.477/-1.025

Comments

Treatment of hydrogen atoms

Riding model on idealized geometries with the 1.2 fold isotropic displacement parameters of the equivalent U_{ij} of the corresponding carbon atom. The methyl groups are idealized with tetrahedral angles in a combined rotating and rigid group refinement with the 1.5 fold isotropic displacement parameters of the equivalent U_{ij} of the corresponding carbon atom.

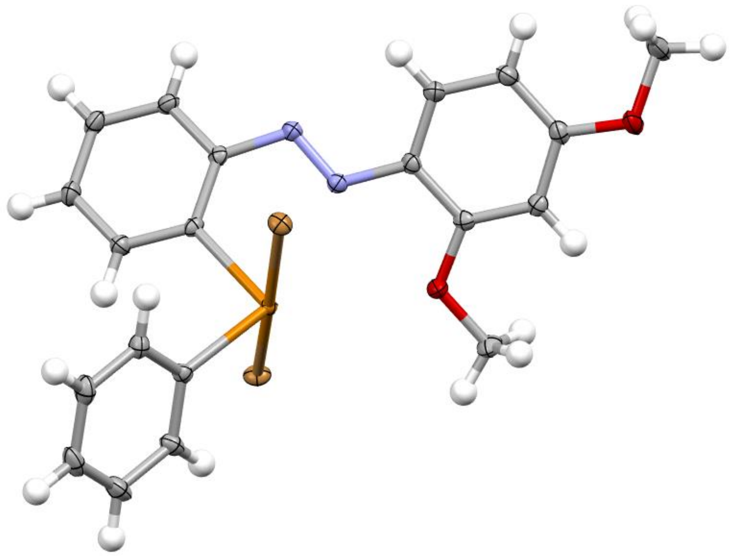

Figure S85. Molecular structure of **3e** with thermal ellipsoids at 50% probability level.

Crystal Structure Data of 2f

Table S15. Crystal structure data and structure refinement for **2f**.

Deposition number	2444463
Empirical formula	$C_{17}H_{13}Cl_2N_3Te$
Formula weight	457.80
Density (calculated)	1.829 g·cm^{-3}
F(000)	444
Temperature	100(2) K
Crystal size	0.227 × 0.138 × 0.109 mm
Crystal color	orange
Crystal description	tablet
Wavelength	0.71073 Å
Crystal system	triclinic
Space group	$P\bar{1}$
Unit cell dimensions	
a [Å]	8.0709(4)
b [Å]	9.0713(4)
c [Å]	12.1097(6)
α [°]	85.595(2)
β [°]	71.6224(19)
γ [°]	81.222(2)
Volume	831.13(7) Å^3
Z	2
Cell measurement reflections used	9437
Cell measurement *θ* min/max	2.27°/36.46°
Diffractometer control software	BRUKER APEX3(v2019.1-0)
Diffractometer measurement device	Bruker D8 KAPPA II (APEX II detector)
Diffractometer measurement method	Data collection strategy APEX 3/Queen
θ range for data collection	2.273°- 36.604°
Completeness to *θ* = 25.242°	100.0%
Completeness to θ_{max} = 36.604°	99.7%
Index ranges	$-12 \leq h \leq 13$
	$-15 \leq k \leq 15$
	$0 \leq l \leq 20$
Computing data reduction	BRUKER APEX3(v2019.1-0)
Absorption coefficient	2.112 mm^{-1}
Absorption correction	Semi-empirical from equivalents
Computation absorption correction	TWINABS
Max./min. Transmission	0.75/0.58
R_{merg} before/after correction	0.0561/0.0367 and 0.0863/0.0492
Computing structure solution	BRUKER APEX3(v2019.1-0)
Computing structure refinement	SHELXL-2017/1 (Sheldrick, 2017)
Refinement method	Full-matrix least-squares on F^2
Reflections collected	167446
Independent reflections	8224
R_{int}	0.0464
Reflections with $I > 2\sigma(I)$	7820

Restraints	0
Parameter	208
GooF	1.062
Weighting details	$w = 1/[\sigma^2(F_{obs}^2) + (0.0317P)^2 + 0.7333P]$<br>where $P = (F_{obs}^2 + 2F_{calc}^2)/3$
R_1 [$I > 2\sigma(I)$]	0.0222
wR_2 [$I > 2\sigma(I)$]	0.0571
R_1 [all data]	0.0240
wR_2 [all data]	0.0582
Largest diff. peak and hole	2.490/-0.593

Comments

Treatment of hydrogen atoms

Riding model on idealized geometries with the 1.2 fold isotropic displacement parameters of the equivalent U_{ij} of the corresponding carbon atom.

Twinning

The crystal was a non-merhedral twin and the model was refined against de-twinned hklf4 data.

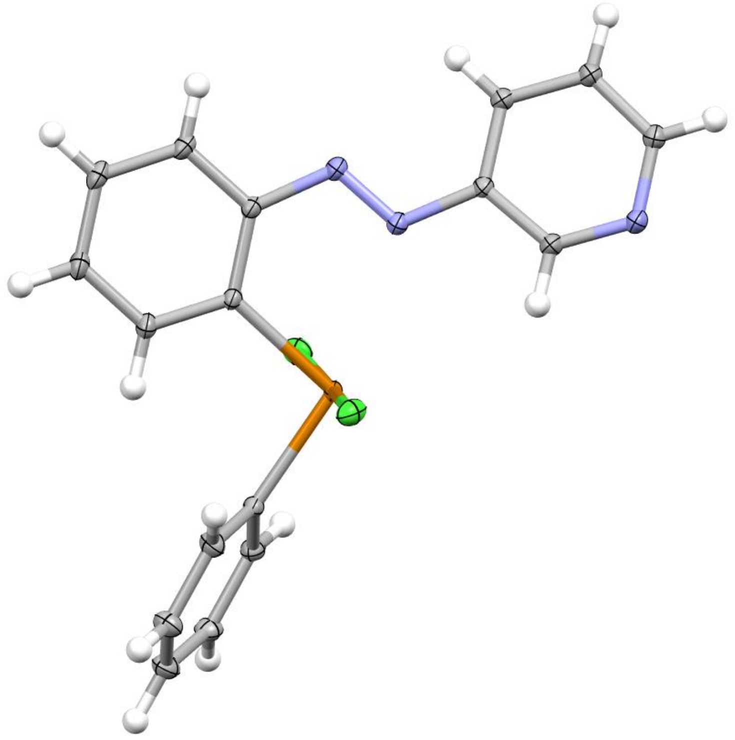

Figure S86. Molecular structure of **2f** with thermal ellipsoids at 50% probability level.

Crystal Structure Data of 3f

Table S16. Crystal structure data and structure refinement for **3f**.

Deposition number	2444468
Empirical formula	$C_{17}H_{13}Br_2N_3Te$
Formula weight	546.72
Density (calculated)	2.112 g·cm^{-3}
F(000)	516
Temperature	100(2) K
Crystal size	0.150 × 0.120 × 0.050 mm
Crystal color	dark orange
Crystal description	tablet
Wavelength	0.71073 Å
Crystal system	triclinic
Space group	$P\bar{1}$
Unit cell dimensions	
a [Å]	8.3353(6)
b [Å]	9.3679(6)
c [Å]	11.8284(8)
α [°]	84.512(3)
β [°]	71.509(3)
γ [°]	79.217(3)
Volume	859.81(10) Å^3
Z	2
Cell measurement reflections used	9900
Cell measurement *θ* min/max	2.81°/33.49°
Diffractometer control software	BRUKER APEX3(v2019.1-0)
Diffractometer measurement device	Bruker D8 KAPPA II (APEX II detector)
Diffractometer measurement method	Data collection strategy APEX 3/Queen
θ range for data collection	1.817°- 33.660°
Completeness to *θ* = 25.242°	100.0%
Completeness to θ_{max} = 33.660°	98.9%
Index ranges	$-12 \leq h \leq 12$
	$-14 \leq k \leq 14$
	$-18 \leq l \leq 18$
Computing data reduction	BRUKER APEX3(v2019.1-0)
Absorption coefficient	6.379 mm^{-1}
Absorption correction	Semi-empirical from equivalents
Computation absorption correction	SADABS
Max./min. Transmission	0.75/0.53
R_{merg} before/after correction	0.0878/0.0416
Computing structure solution	BRUKER APEX3(v2019.1-0)
Computing structure refinement	SHELXL-2017/1 (Sheldrick, 2017)
Refinement method	Full-matrix least-squares on F^2
Reflections collected	73070
Independent reflections	6755
R_{int}	0.0275
Reflections with $I > 2\sigma(I)$	6285

Restraints	0
Parameter	208
GooF	1.036
Weighting details	$w = 1/[\sigma^2(F_{obs}^2) + (0.0222P)^2 + 0.4000P]$ where $P = (F_{obs}^2 + 2F_{calc}^2)/3$
R_1 [$I > 2\sigma(I)$]	0.0158
wR_2 [$I > 2\sigma(I)$]	0.0408
R_1 [all data]	0.0182
wR_2 [all data]	0.0415
Largest diff. peak and hole	0.891/-0.685

Comments

Treatment of hydrogen atoms

Riding model on idealized geometries with the 1.2 fold isotropic displacement parameters of the equivalent U_{ij} of the corresponding carbon atom.

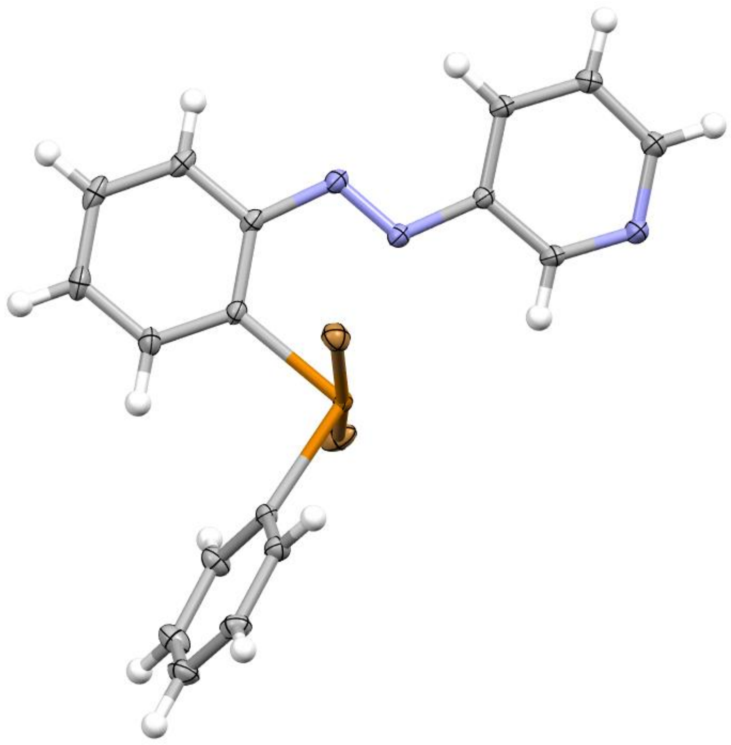

Figure S87. Molecular structure of **3f** with thermal ellipsoids at 50% probability level.

8. ^{1}H NMR, ^{13}C NMR and ^{125}Te NMR Spectra of the New Compounds

The field strengths and solvents of the following spectra are given in the Experimental Section.

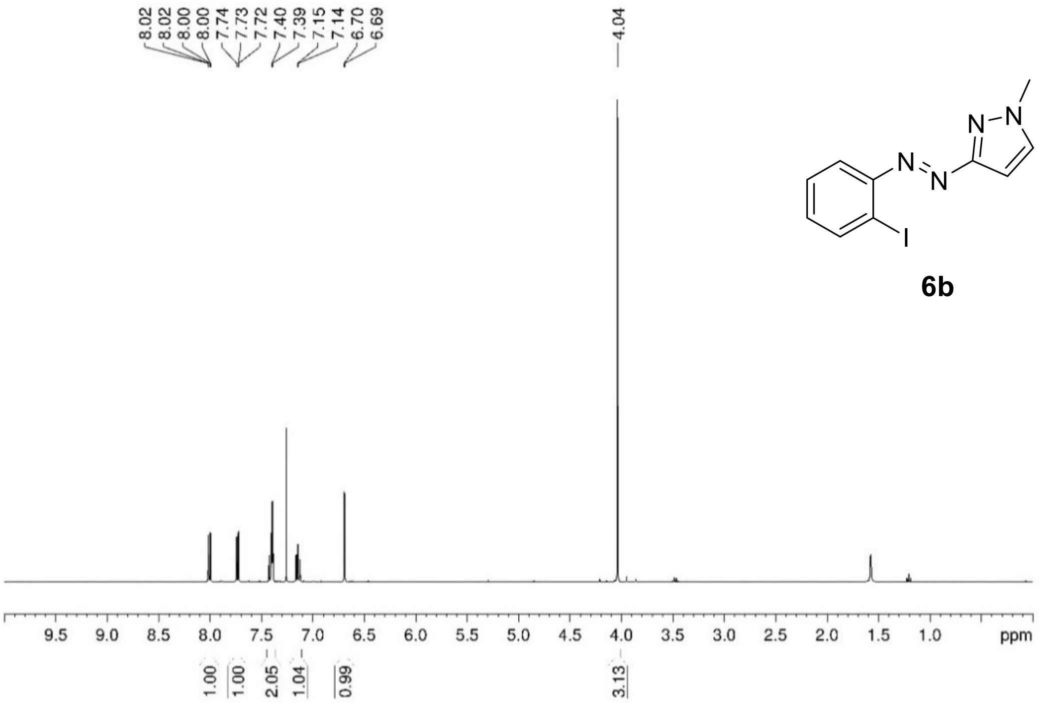

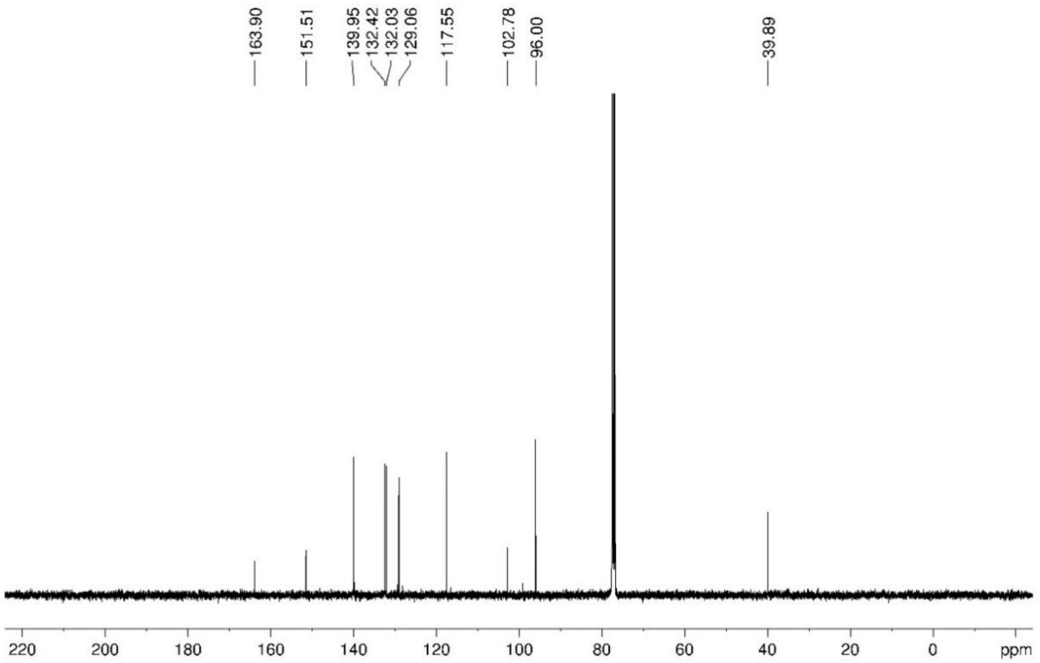

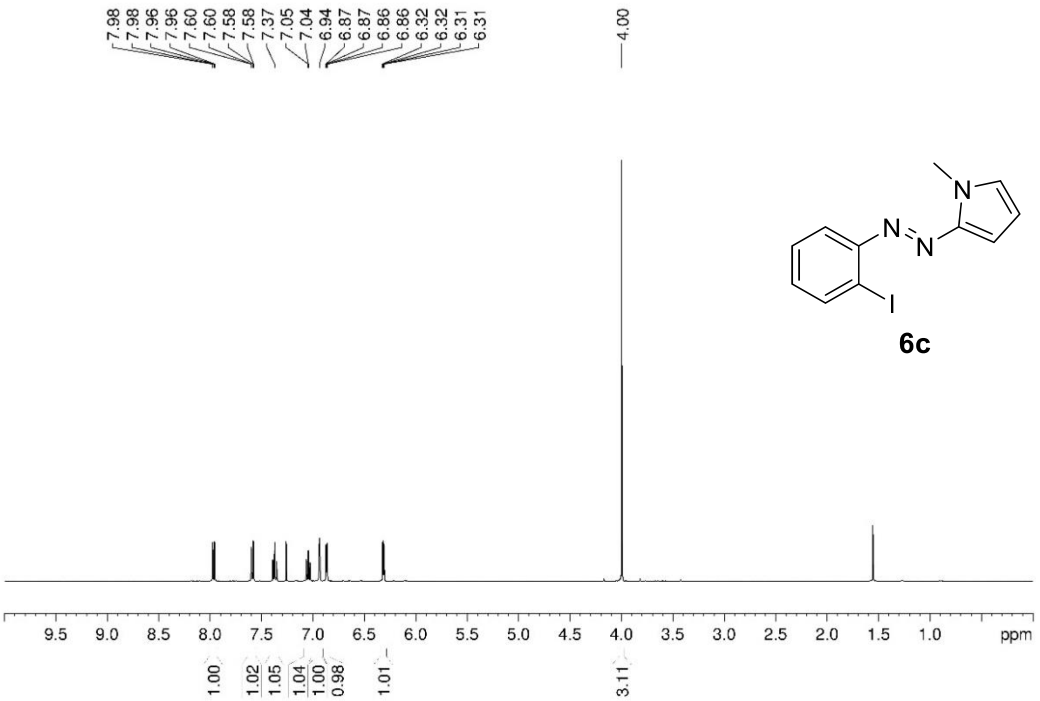
7.98
7.98
7.96
7.96
7.60
7.60
7.58
7.58
7.37
7.05
7.04
6.94
6.87
6.87
6.86
6.86
6.32
6.32
6.31
6.31
4.00
9.5 9.0 8.5 8.0 7.5 7.0 6.5 6.0 5.5 5.0 4.5 4.0 3.5 3.0 2.5 2.0 1.5 1.0 ppm
1.00
1.02
1.05
1.04
1.00
0.98
1.01
3.11
N
N
N
I

6c

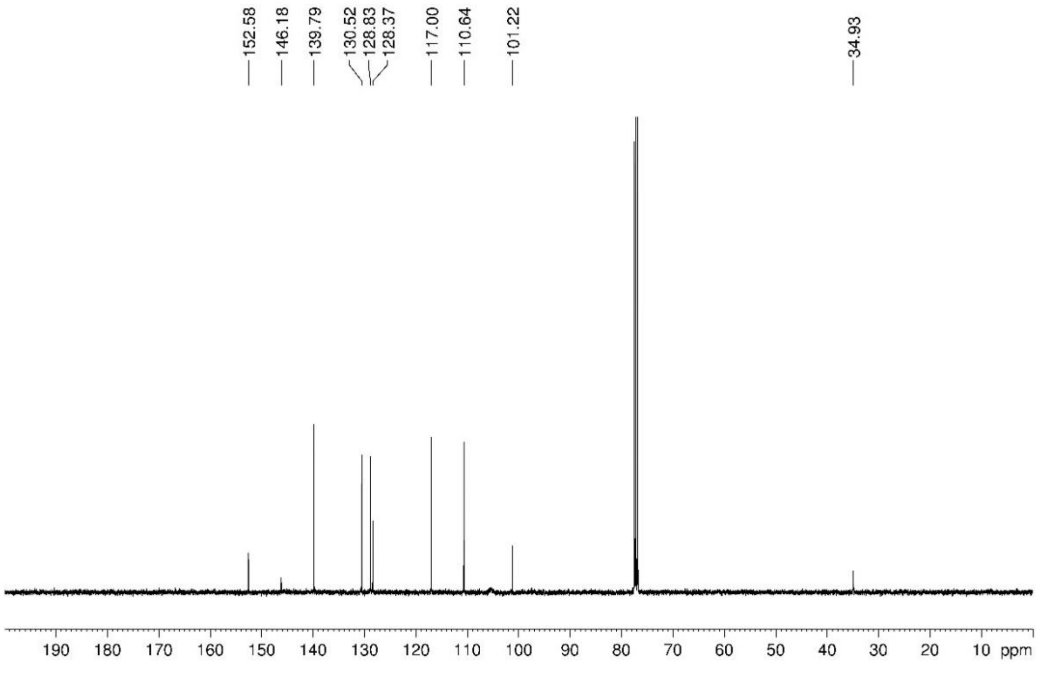
152.58
146.18
139.79
130.52
128.83
128.37
117.00
110.64
101.22
34.93
190 180 170 160 150 140 130 120 110 100 90 80 70 60 50 40 30 20 10 ppm

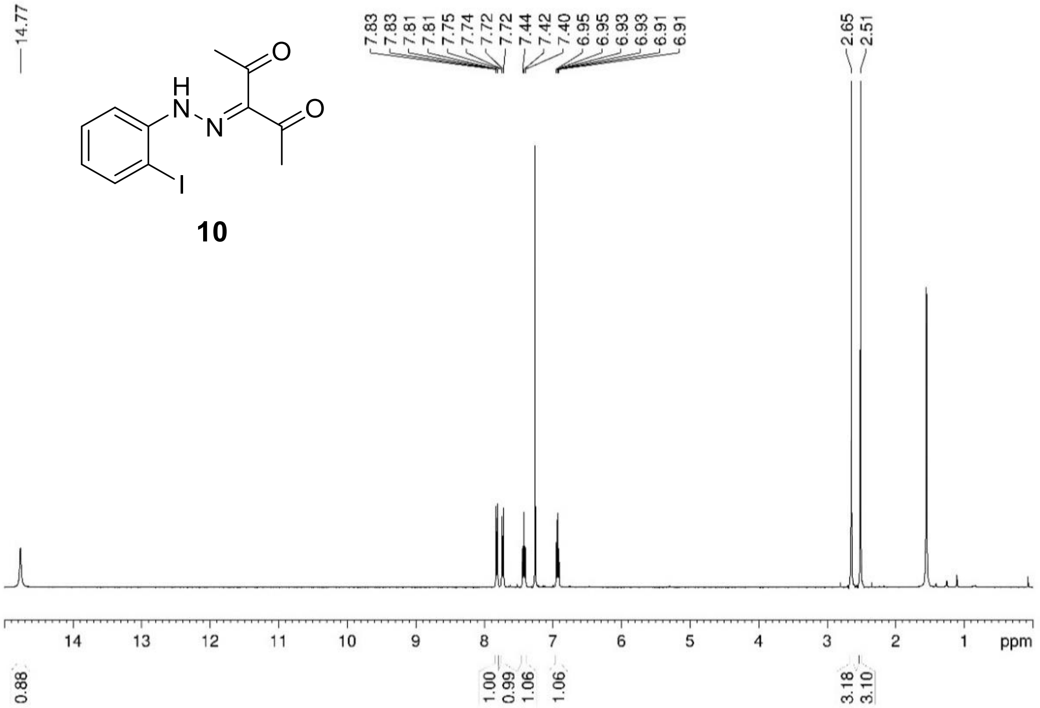
14.77
7.83
7.83
7.81
7.81
7.75
7.74
7.72
7.72
7.44
7.42
7.40
6.95
6.95
6.93
6.93
6.91
6.91
2.65
2.51
10
14
13
12
11
10
9
8
7
6
5
4
3
2
1
ppm
0.88
1.00
0.99
1.06
1.06
3.18
3.10

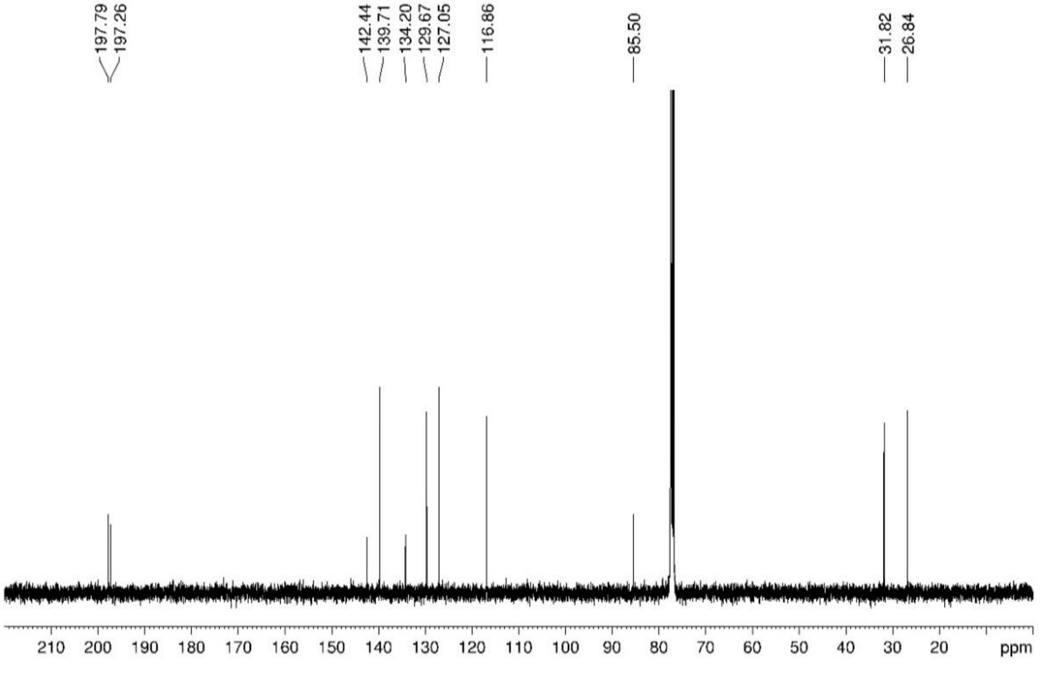
197.79
197.26
142.44
139.71
134.20
129.67
127.05
116.86
85.50
31.82
26.84
210
200
190
180
170
160
150
140
130
120
110
100
90
80
70
60
50
40
30
20
ppm

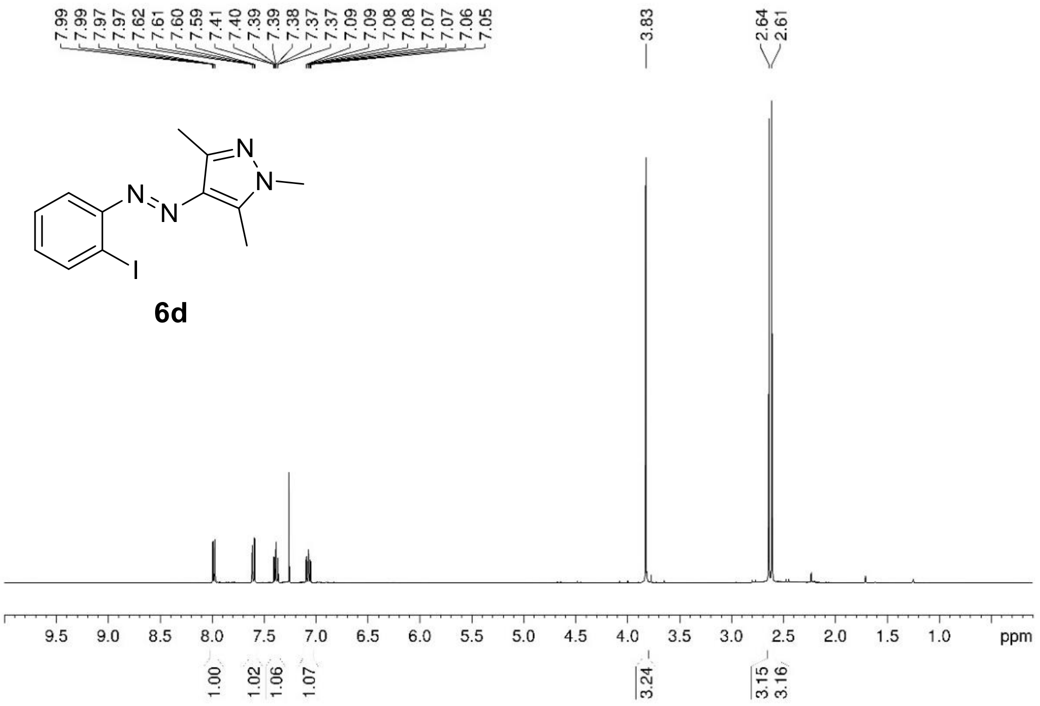
7.99 7.99 7.97 7.97 7.62 7.61 7.60 7.59 7.41 7.40 7.39 7.39 7.38 7.37 7.37 7.09 7.09 7.08 7.08 7.07 7.07 7.06 7.05
3.83
2.64 2.61
N
N
N
N
I
6d
9.5 9.0 8.5 8.0 7.5 7.0 6.5 6.0 5.5 5.0 4.5 4.0 3.5 3.0 2.5 2.0 1.5 1.0 ppm
1.00 1.02 1.06 1.07 3.24 3.15 3.16

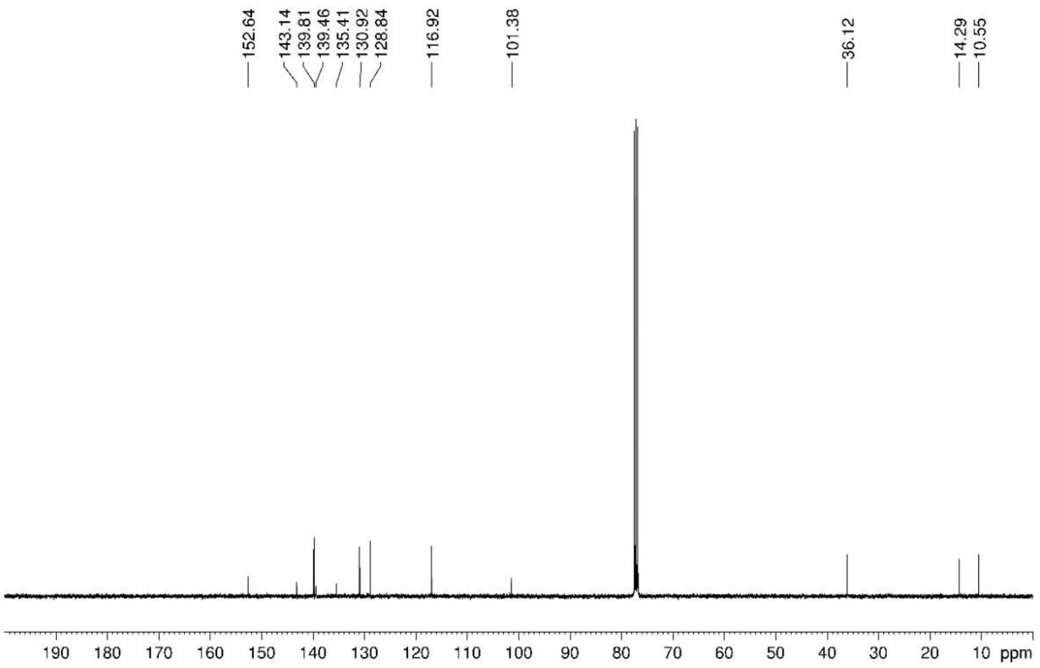
152.64 143.14 139.81 139.46 135.41 130.92 128.84 116.92 101.38 36.12 14.29 10.55
190 180 170 160 150 140 130 120 110 100 90 80 70 60 50 40 30 20 10 ppm

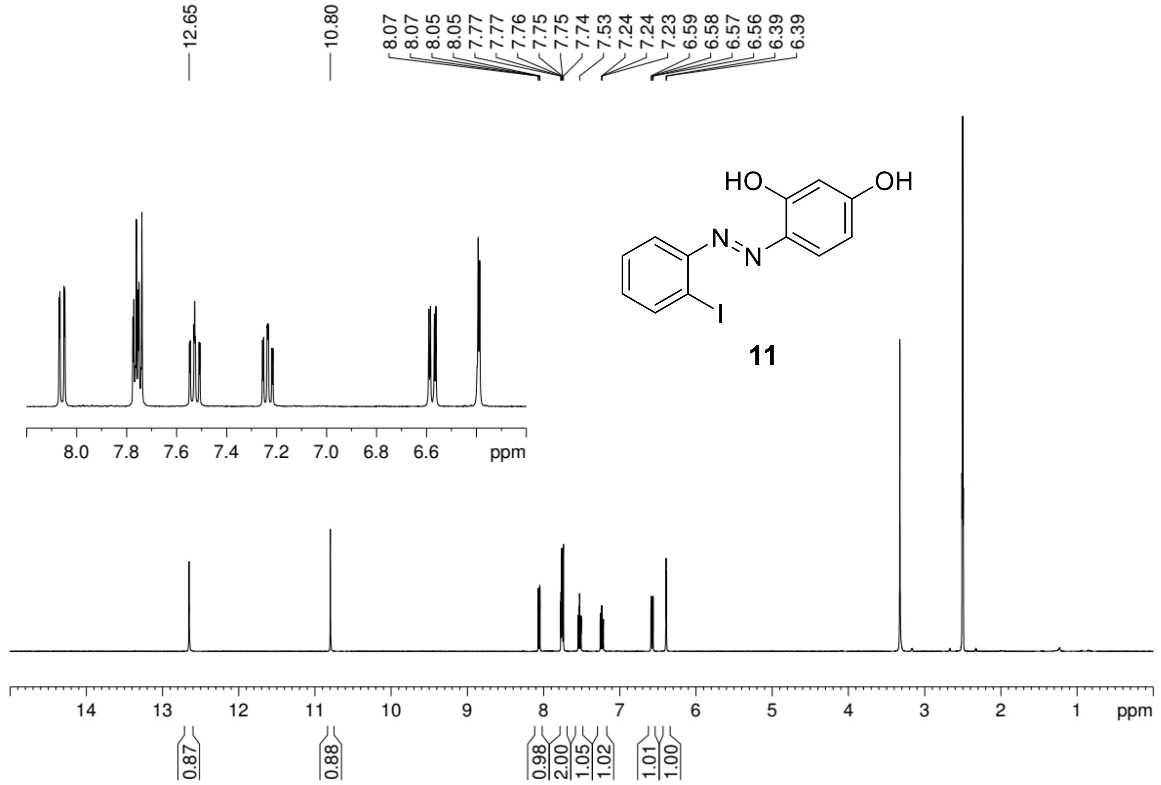
12.65
10.80
8.07 8.07 8.05 8.05 7.77 7.77 7.76 7.75 7.75 7.74 7.53 7.24 7.24 7.23 6.59 6.58 6.57 6.56 6.39 6.39
HO
OH
N
N
I
11
8.0 7.8 7.6 7.4 7.2 7.0 6.8 6.6 ppm
14 13 12 11 10 9 8 7 6 5 4 3 2 1 ppm
0.87
0.88
0.98 2.00 1.05 1.02
1.01 1.00

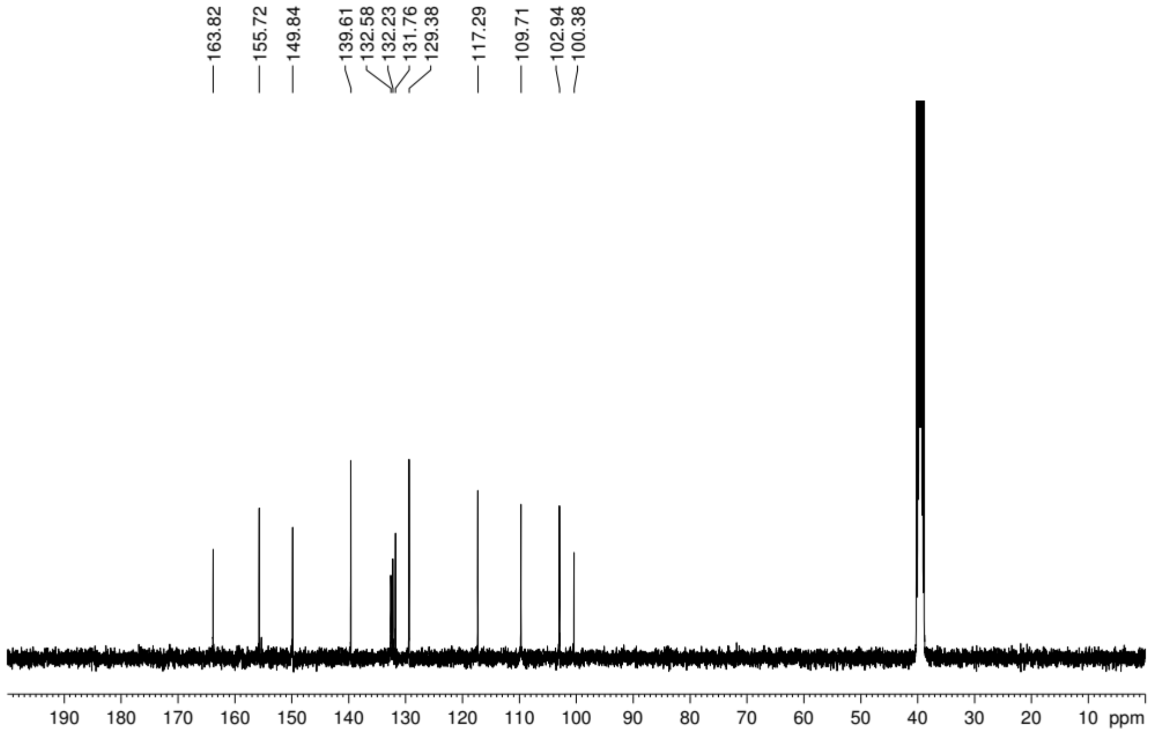
163.82
155.72
149.84
139.61 132.58 132.23 131.76 129.38
117.29
109.71
102.94 100.38
190 180 170 160 150 140 130 120 110 100 90 80 70 60 50 40 30 20 10 ppm

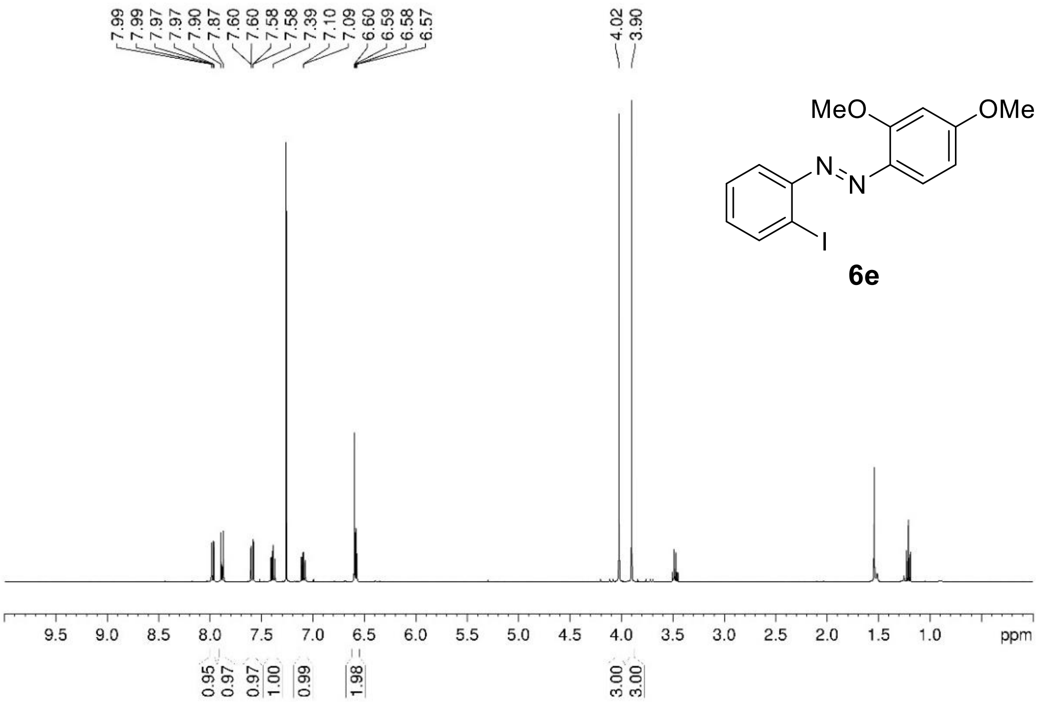
7.99
7.99
7.97
7.97
7.90
7.87
7.60
7.60
7.58
7.58
7.39
7.10
7.09
6.60
6.59
6.58
6.57
4.02
3.90
MeO
OMe
N
N
I
6e
9.5
9.0
8.5
8.0
7.5
7.0
6.5
6.0
5.5
5.0
4.5
4.0
3.5
3.0
2.5
2.0
1.5
1.0
ppm
0.95
0.97
0.97
1.00
0.99
1.98
3.00
3.00

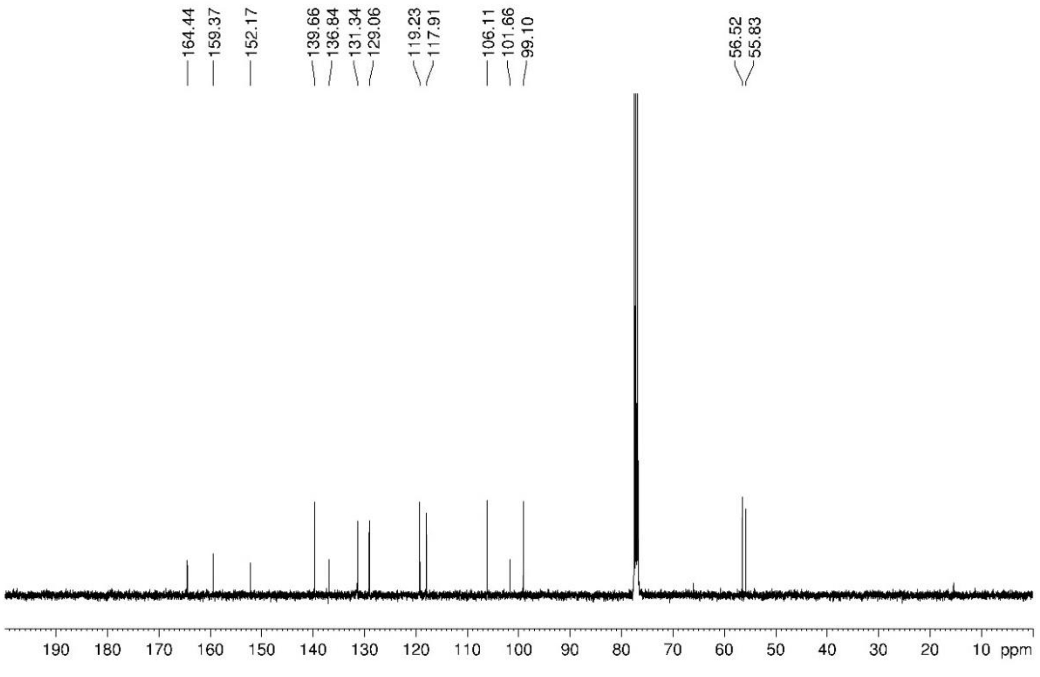
164.44
159.37
152.17
139.66
136.84
131.34
129.06
119.23
117.91
106.11
101.66
99.10
56.52
55.83
190
180
170
160
150
140
130
120
110
100
90
80
70
60
50
40
30
20
10
ppm

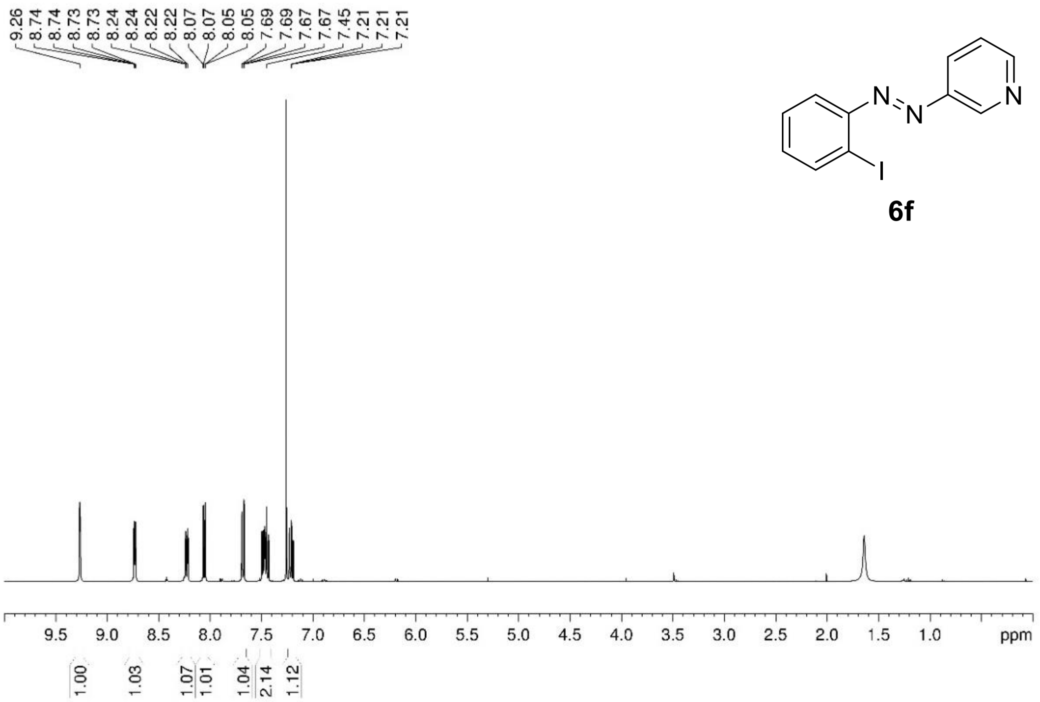
9.26
8.74
8.74
8.73
8.73
8.24
8.24
8.22
8.22
8.07
8.07
8.05
8.05
7.69
7.69
7.67
7.67
7.45
7.21
7.21
7.21
9.5 9.0 8.5 8.0 7.5 7.0 6.5 6.0 5.5 5.0 4.5 4.0 3.5 3.0 2.5 2.0 1.5 1.0 ppm
1.00
1.03
1.07
1.01
1.04
2.14
1.12
N
N
N
I

6f

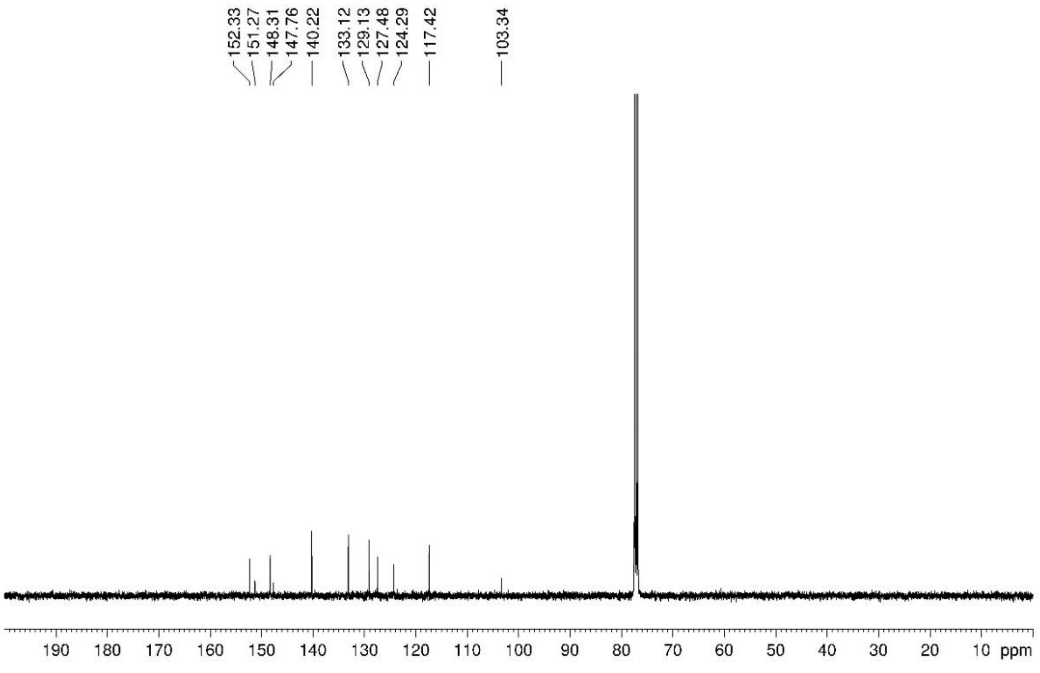
152.33
151.27
148.31
147.76
140.22
133.12
129.13
127.48
124.29
117.42
103.34
190 180 170 160 150 140 130 120 110 100 90 80 70 60 50 40 30 20 10 ppm

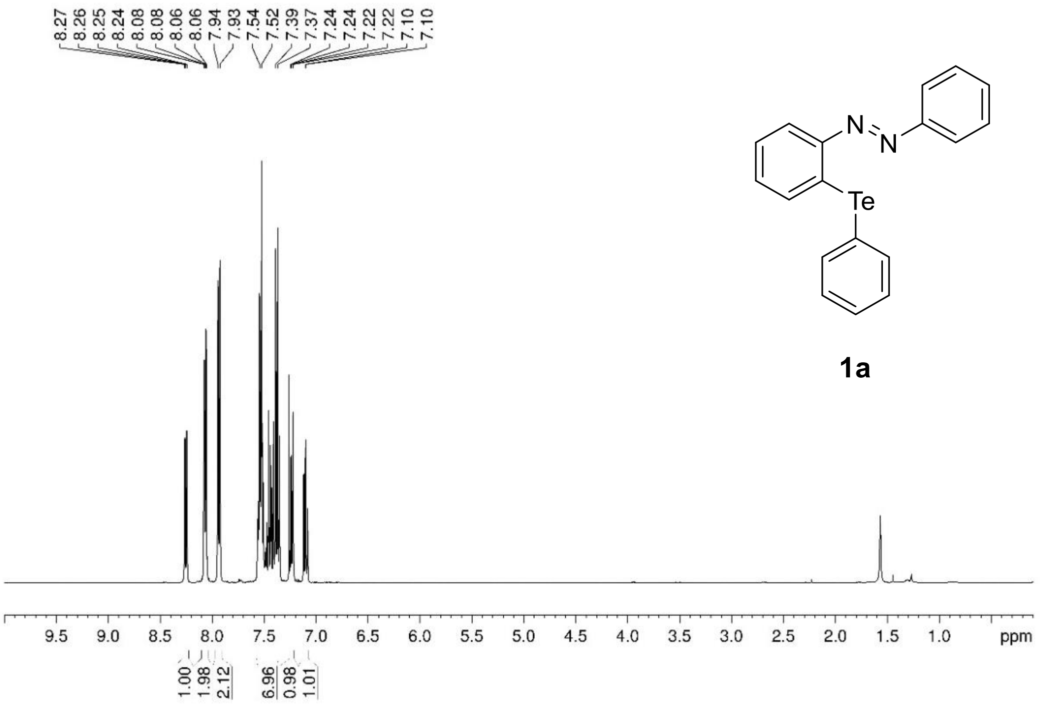

8.27 8.26 8.25 8.24 8.08 8.08 8.06 8.06 7.94 7.93 7.54 7.52 7.39 7.37 7.24 7.24 7.22 7.22 7.10 7.10
9.5 9.0 8.5 8.0 7.5 7.0 6.5 6.0 5.5 5.0 4.5 4.0 3.5 3.0 2.5 2.0 1.5 1.0 ppm
1.00 1.98 2.12 6.96 0.98 1.01
N
N
Te

1a

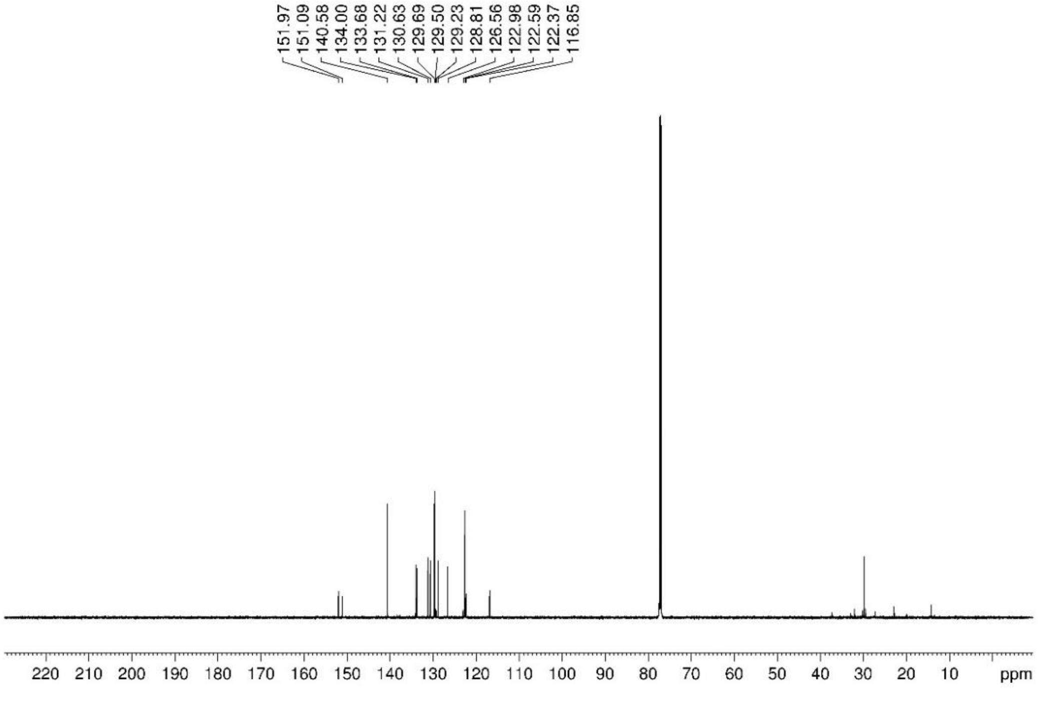

151.97 151.09 140.58 134.00 133.68 131.22 130.63 129.69 129.50 129.23 128.81 126.56 122.98 122.59 122.37 116.85
220 210 200 190 180 170 160 150 140 130 120 110 100 90 80 70 60 50 40 30 20 10 ppm

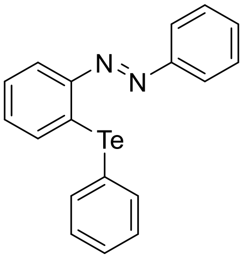

N
N
Te

1a

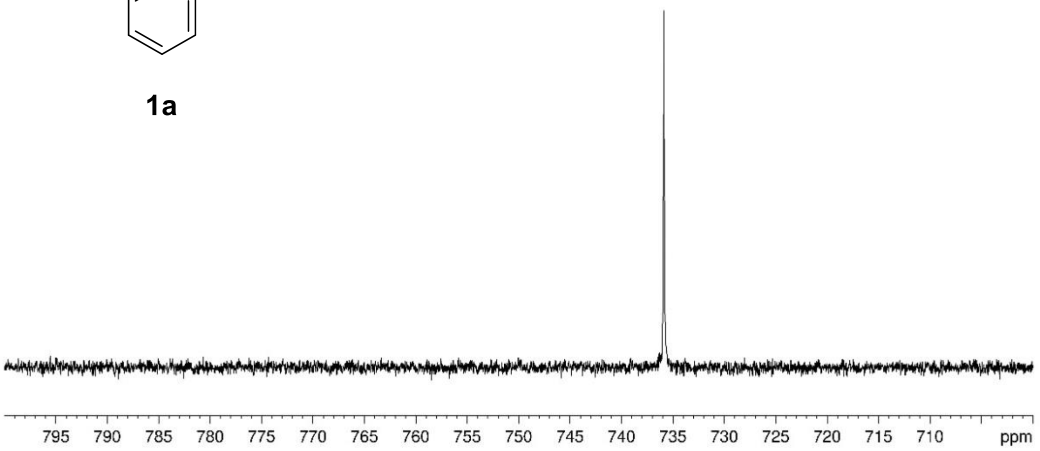

735.88
795 790 785 780 775 770 765 760 755 750 745 740 735 730 725 720 715 710
ppm

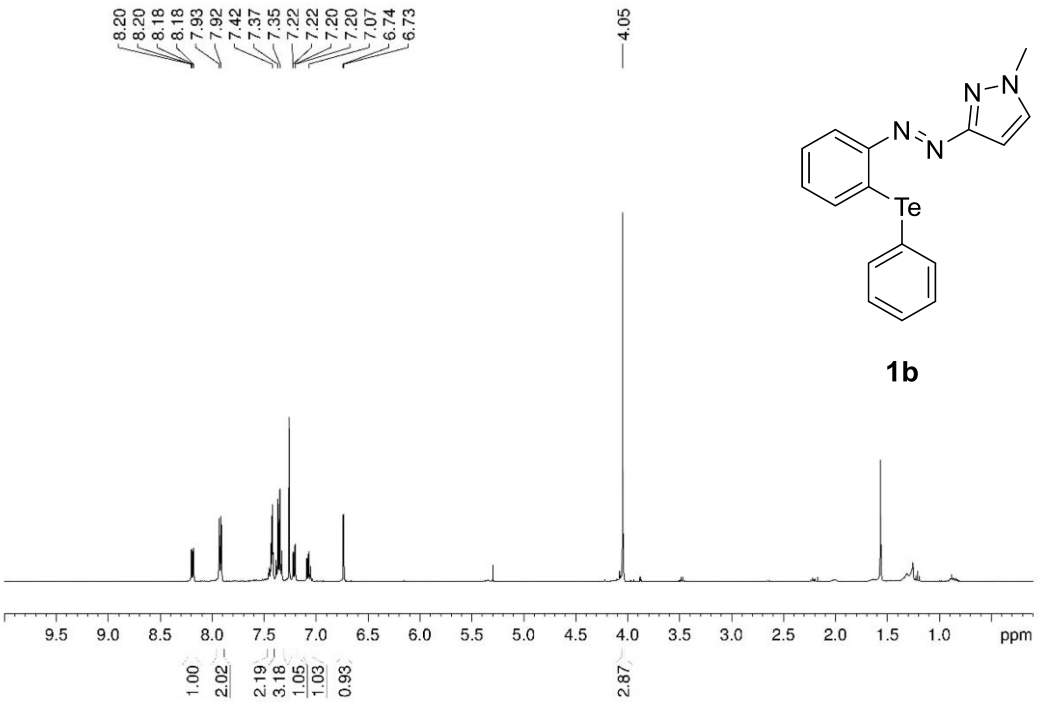
8.20
8.20
8.18
8.18
7.93
7.92
7.42
7.37
7.35
7.22
7.22
7.20
7.20
7.07
6.74
6.73
4.05
N
N
N
N
Te
1b
9.5 9.0 8.5 8.0 7.5 7.0 6.5 6.0 5.5 5.0 4.5 4.0 3.5 3.0 2.5 2.0 1.5 1.0 ppm
1.00
2.02
2.19
3.18
1.05
1.03
0.93
2.87

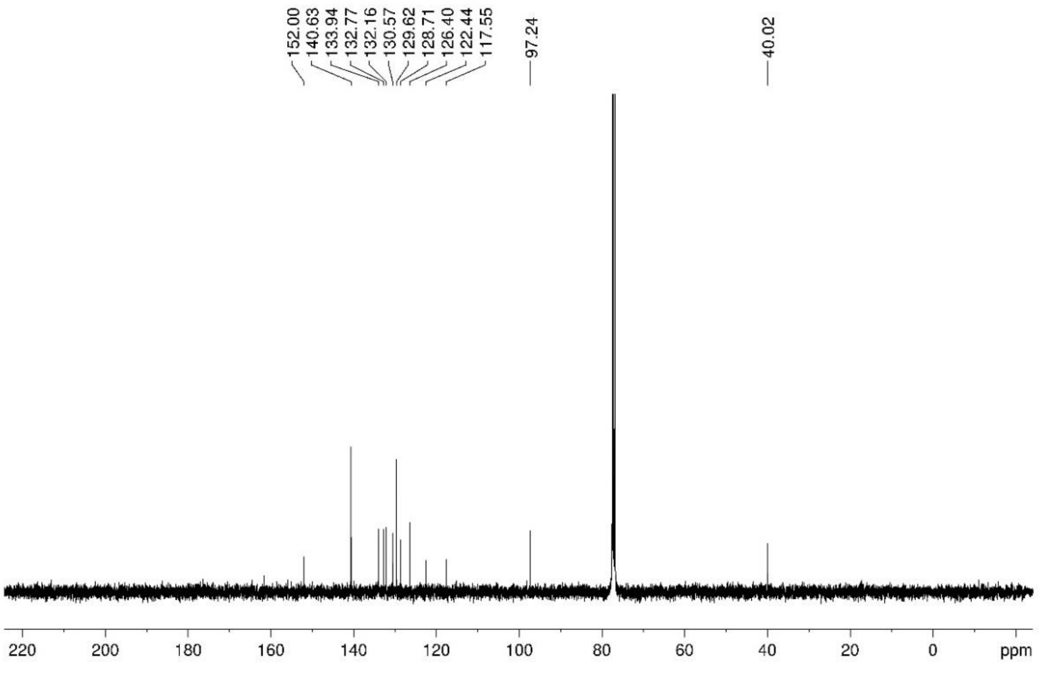
152.00
140.63
133.94
132.77
132.16
130.57
129.62
128.71
126.40
122.44
117.55
97.24
40.02
220 200 180 160 140 120 100 80 60 40 20 0 ppm

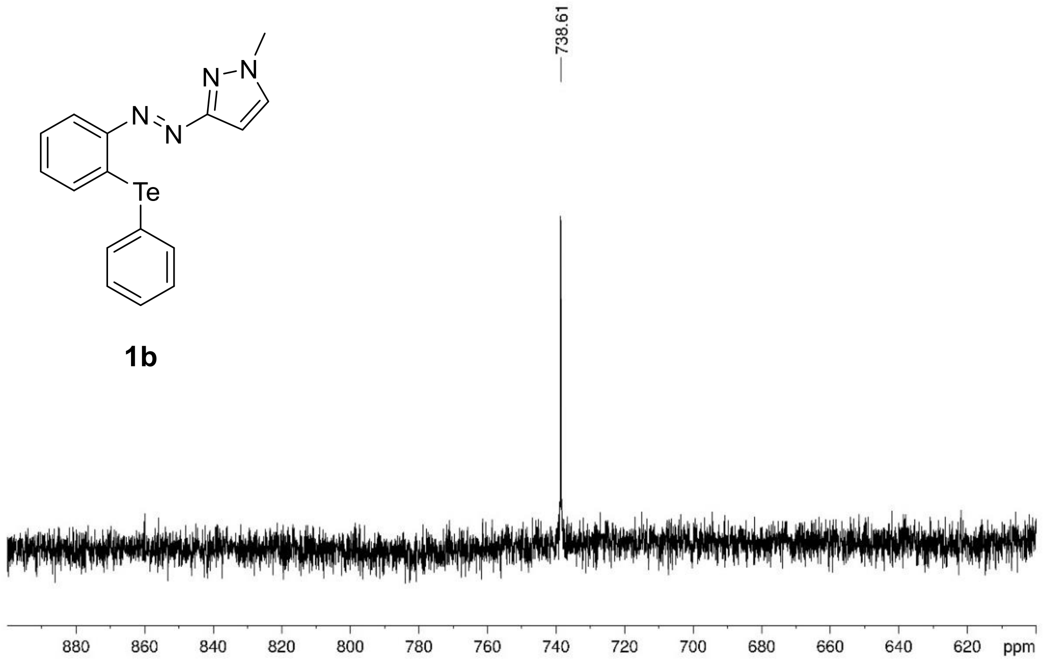
1b
738.61
880
860
840
820
800
780
760
740
720
700
680
660
640
620
ppm

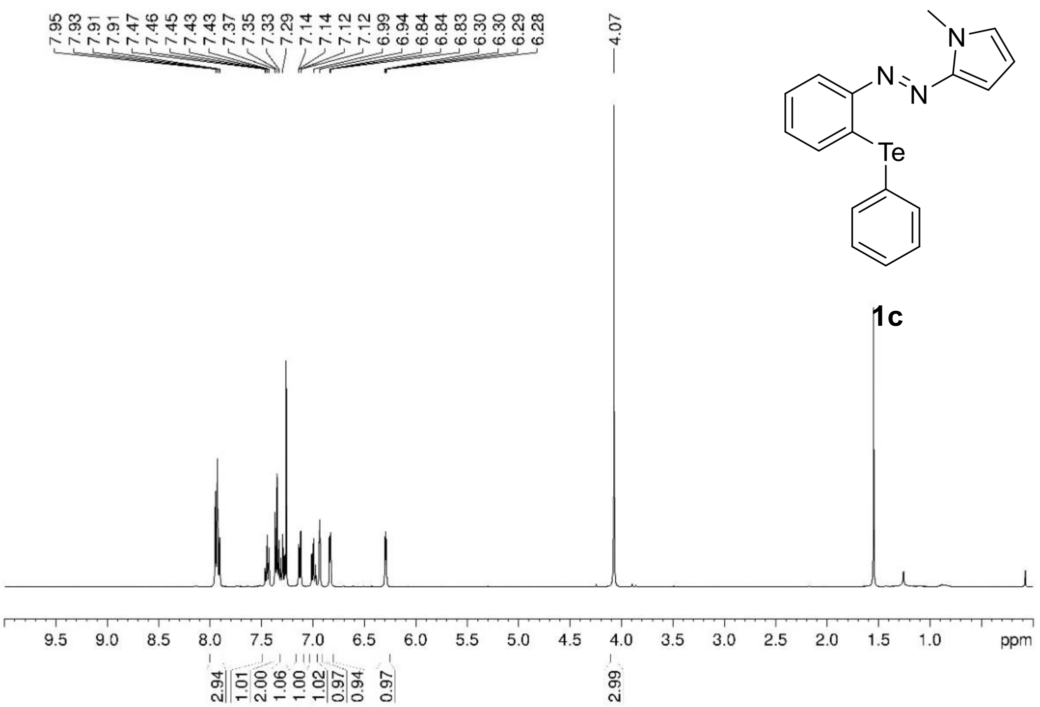
7.95 7.93 7.91 7.91 7.47 7.46 7.45 7.43 7.43 7.37 7.35 7.33 7.29 7.14 7.14 7.12 7.12 6.99 6.94 6.84 6.84 6.83 6.30 6.30 6.29 6.28
4.07
N
N
N
Te
1c
9.5 9.0 8.5 8.0 7.5 7.0 6.5 6.0 5.5 5.0 4.5 4.0 3.5 3.0 2.5 2.0 1.5 1.0 ppm
2.94 1.01 2.00 1.06 1.00 1.02 0.97 0.94 0.97
2.99

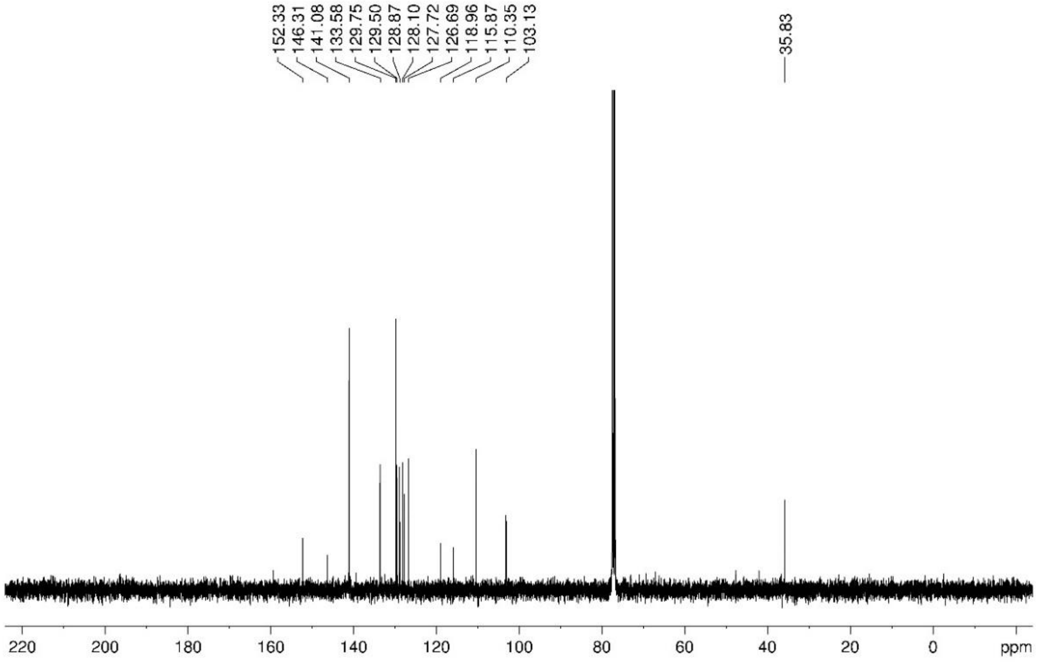
152.33 146.31 141.08 133.58 129.75 129.50 128.87 128.10 127.72 126.69 118.96 115.87 110.35 103.13
35.83
220 200 180 160 140 120 100 80 60 40 20 0 ppm

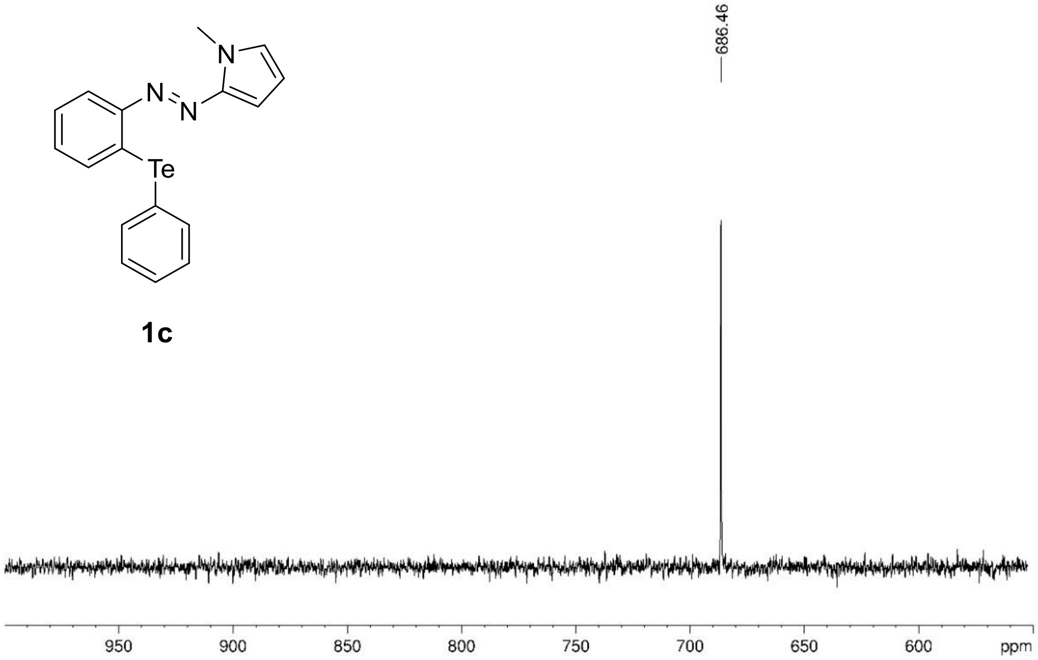
686.46
1c
950
900
850
800
750
700
650
600
ppm

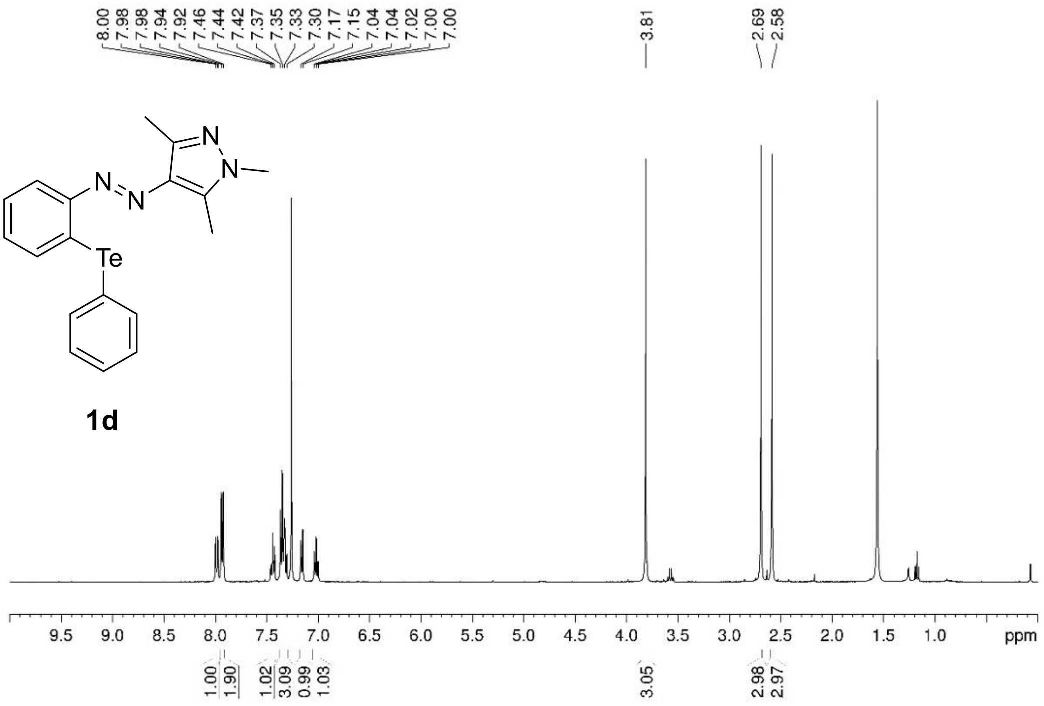
8.00 7.98 7.98 7.94 7.92 7.46 7.44 7.42 7.37 7.35 7.33 7.30 7.17 7.15 7.04 7.04 7.02 7.00 7.00
3.81
2.69 2.58
1d
9.5 9.0 8.5 8.0 7.5 7.0 6.5 6.0 5.5 5.0 4.5 4.0 3.5 3.0 2.5 2.0 1.5 1.0 ppm
1.00 1.90 1.02 3.09 0.99 1.03
3.05
2.98 2.97

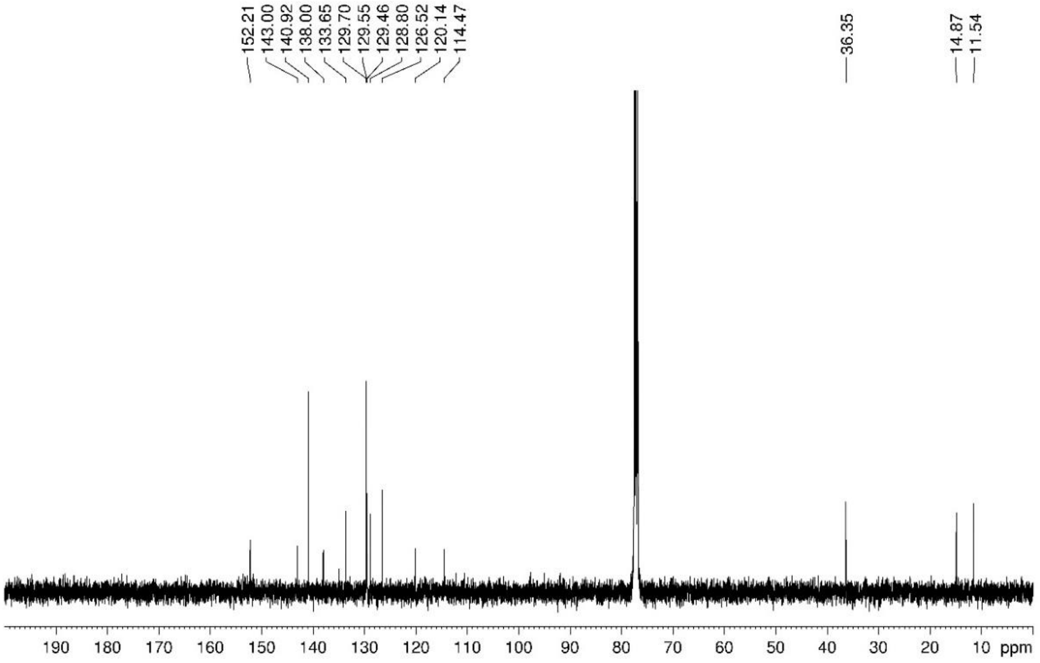
152.21 143.00 140.92 138.00 133.65 129.70 129.55 129.46 128.80 126.52 120.14 114.47
36.35
14.87 11.54
190 180 170 160 150 140 130 120 110 100 90 80 70 60 50 40 30 20 10 ppm

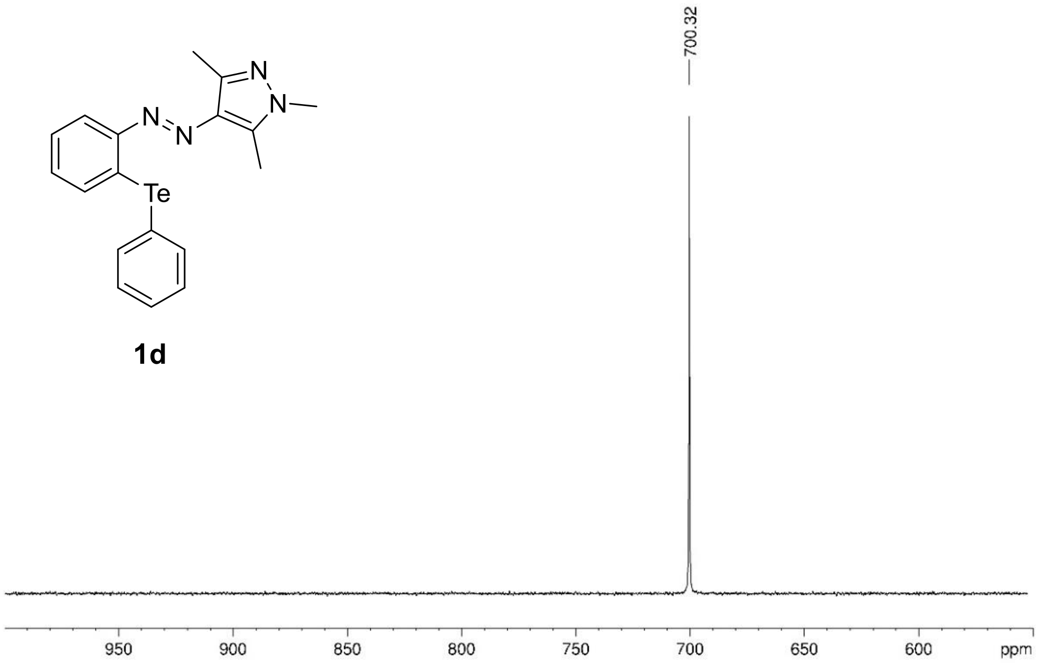

1d

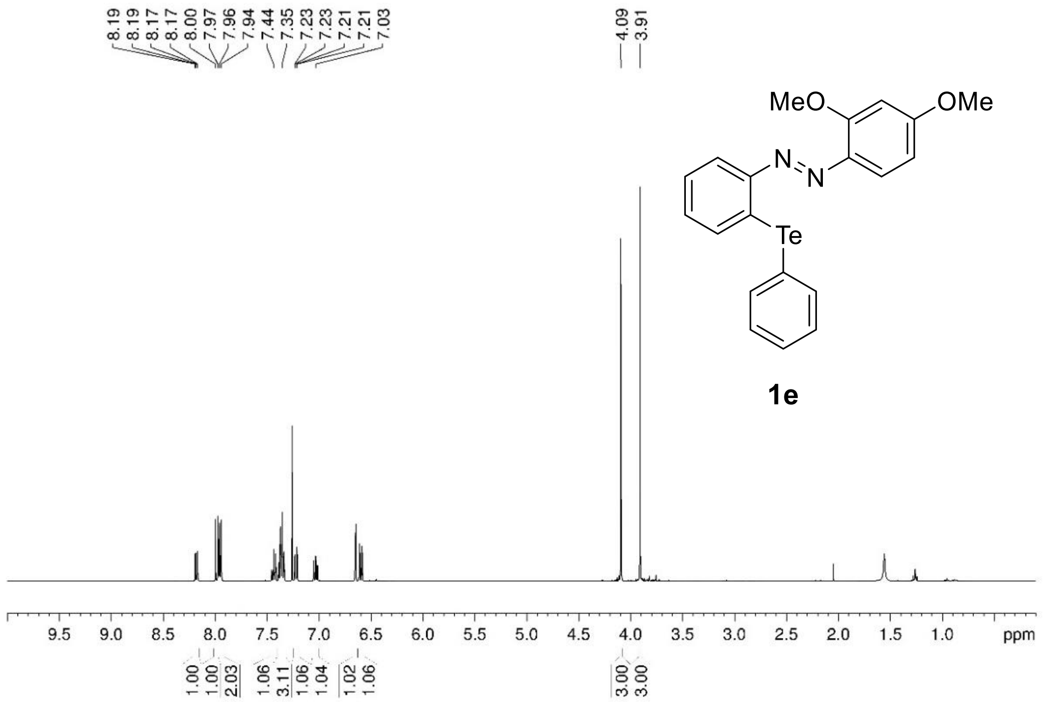
8.19 8.19 8.17 8.17 8.00 7.97 7.96 7.94 7.44 7.35 7.23 7.23 7.21 7.21 7.03
4.09 3.91
MeO
OMe
N
N
Te
1e
9.5 9.0 8.5 8.0 7.5 7.0 6.5 6.0 5.5 5.0 4.5 4.0 3.5 3.0 2.5 2.0 1.5 1.0 ppm
1.00 1.00 2.03 1.06 3.11 1.06 1.04 1.02 1.06
3.00 3.00

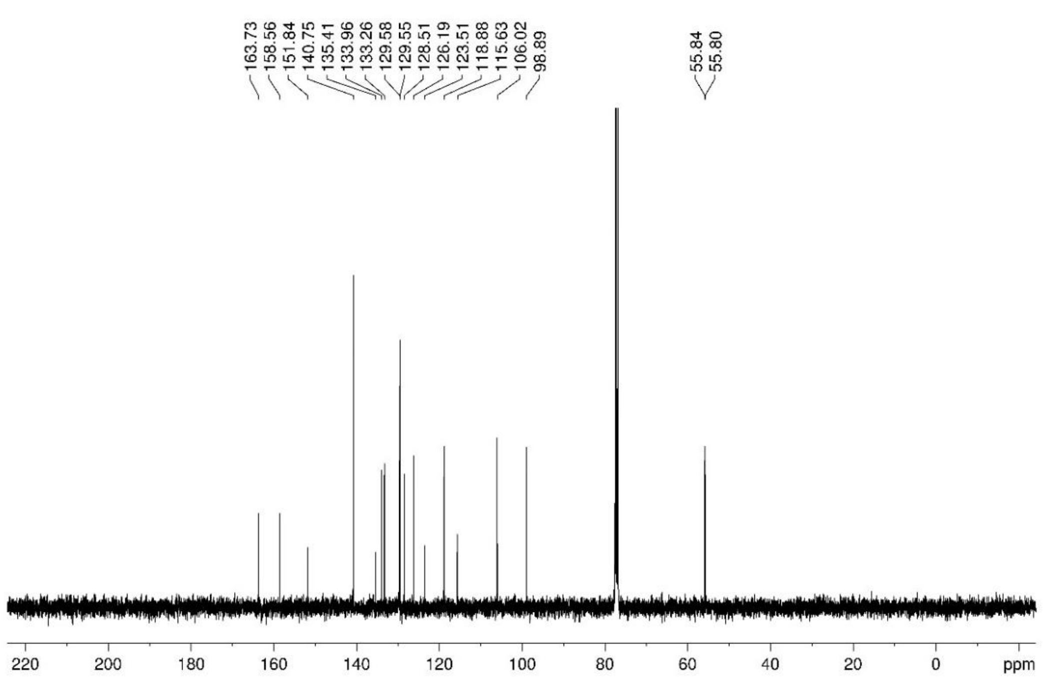
163.73 158.56 151.84 140.75 135.41 133.96 133.26 129.58 129.55 128.51 126.19 123.51 118.88 115.63 106.02 98.89
55.84 55.80
220 200 180 160 140 120 100 80 60 40 20 0 ppm

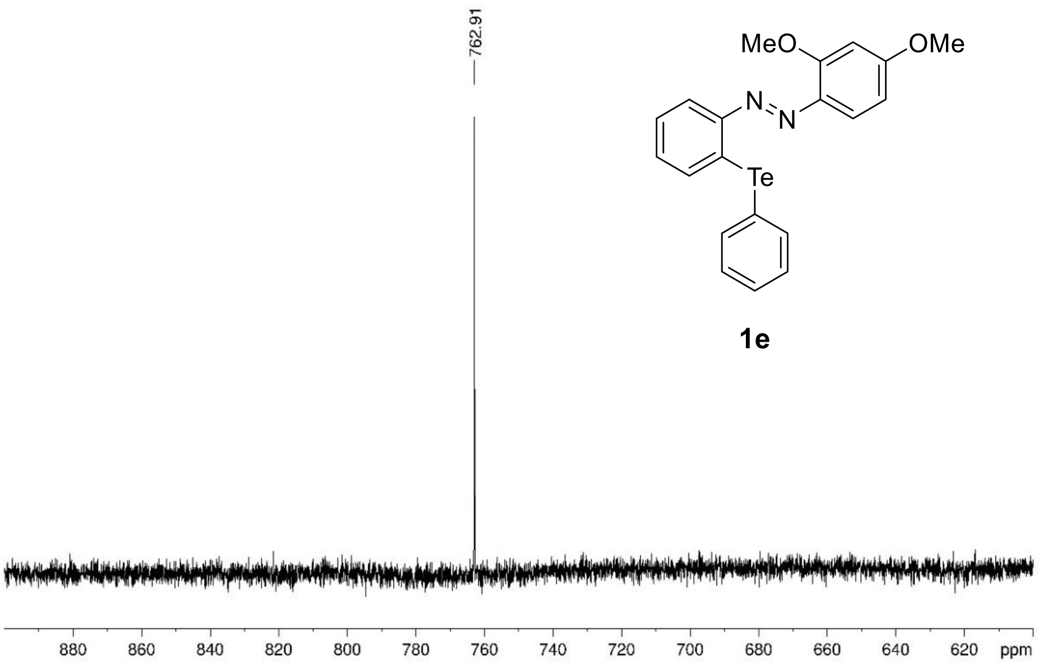
762.91
MeO
OMe
N
N
Te
1e
880
860
840
820
800
780
760
740
720
700
680
660
640
620
ppm

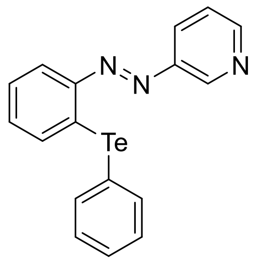
N
N
N
Te

1f

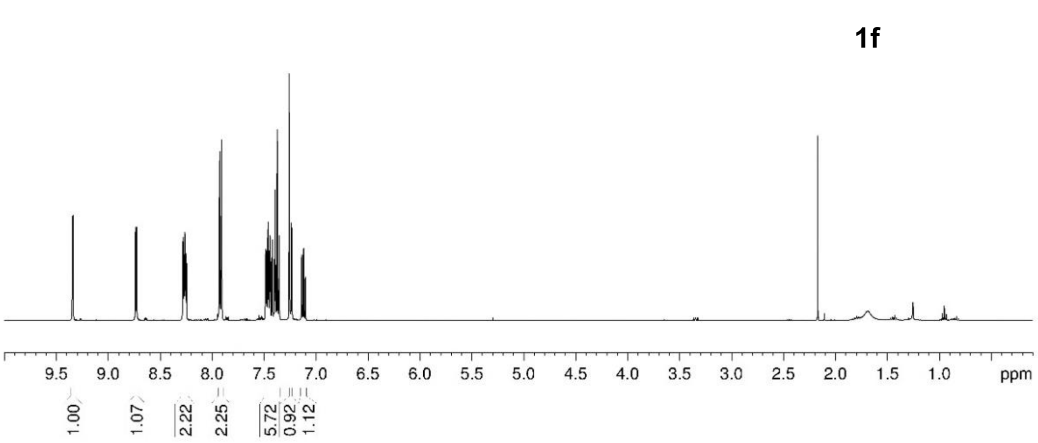
9.35
9.34
8.74
8.74
8.73
8.73
8.28
8.28
8.26
8.26
7.93
7.91
7.46
7.39
7.37
7.26
7.24
7.24
7.12
9.5 9.0 8.5 8.0 7.5 7.0 6.5 6.0 5.5 5.0 4.5 4.0 3.5 3.0 2.5 2.0 1.5 1.0 ppm
1.00
1.07
2.22
2.25
5.72
0.92
1.12

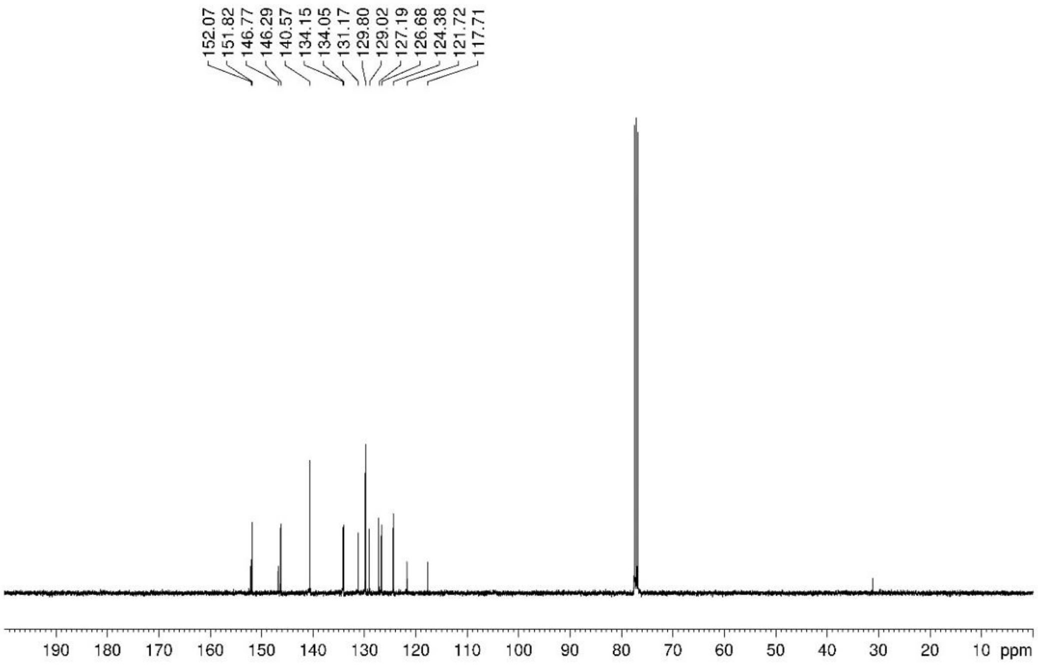
152.07
151.82
146.77
146.29
140.57
134.15
134.05
131.17
129.80
129.02
127.19
126.68
124.38
121.72
117.71
190 180 170 160 150 140 130 120 110 100 90 80 70 60 50 40 30 20 10 ppm

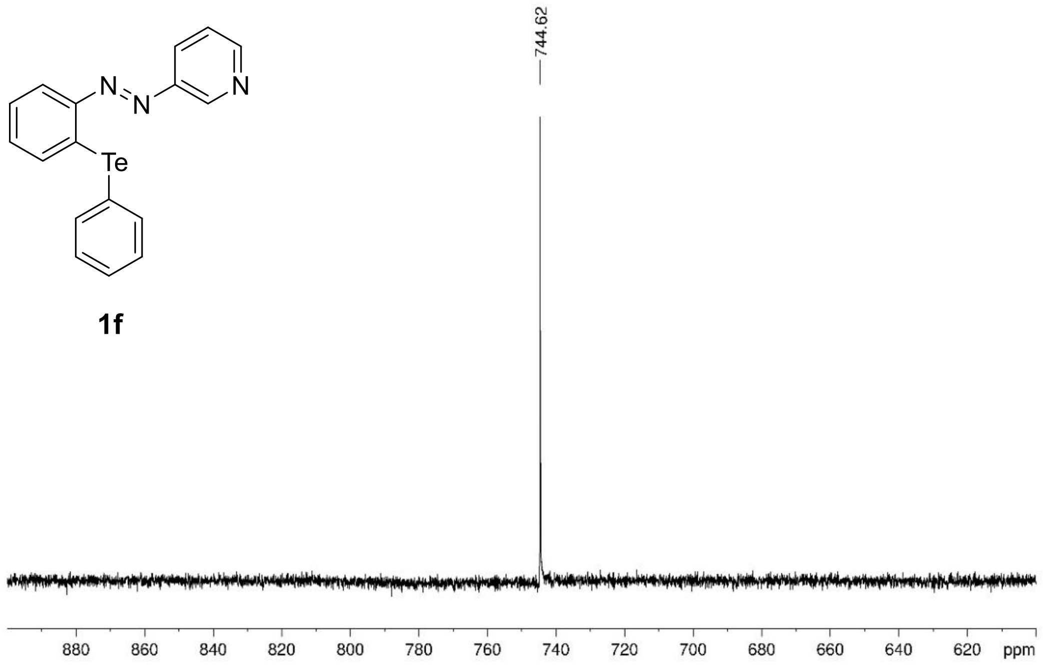
N
N
N
Te
1f
744.62
880 860 840 820 800 780 760 740 720 700 680 660 640 620 ppm

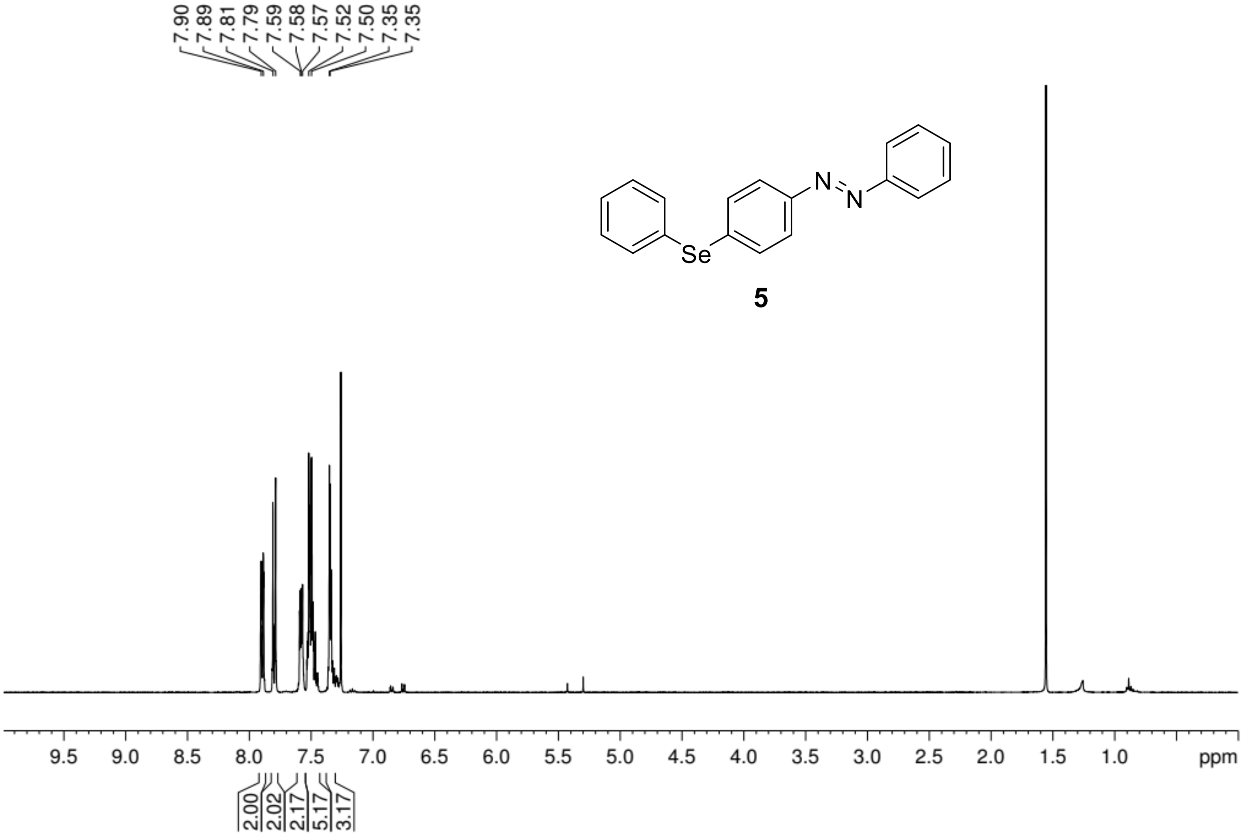
7.90
7.89
7.81
7.79
7.59
7.58
7.57
7.52
7.50
7.35
7.35
Se
N
N
5
9.5 9.0 8.5 8.0 7.5 7.0 6.5 6.0 5.5 5.0 4.5 4.0 3.5 3.0 2.5 2.0 1.5 1.0 ppm
2.00
2.02
2.17
5.17
3.17

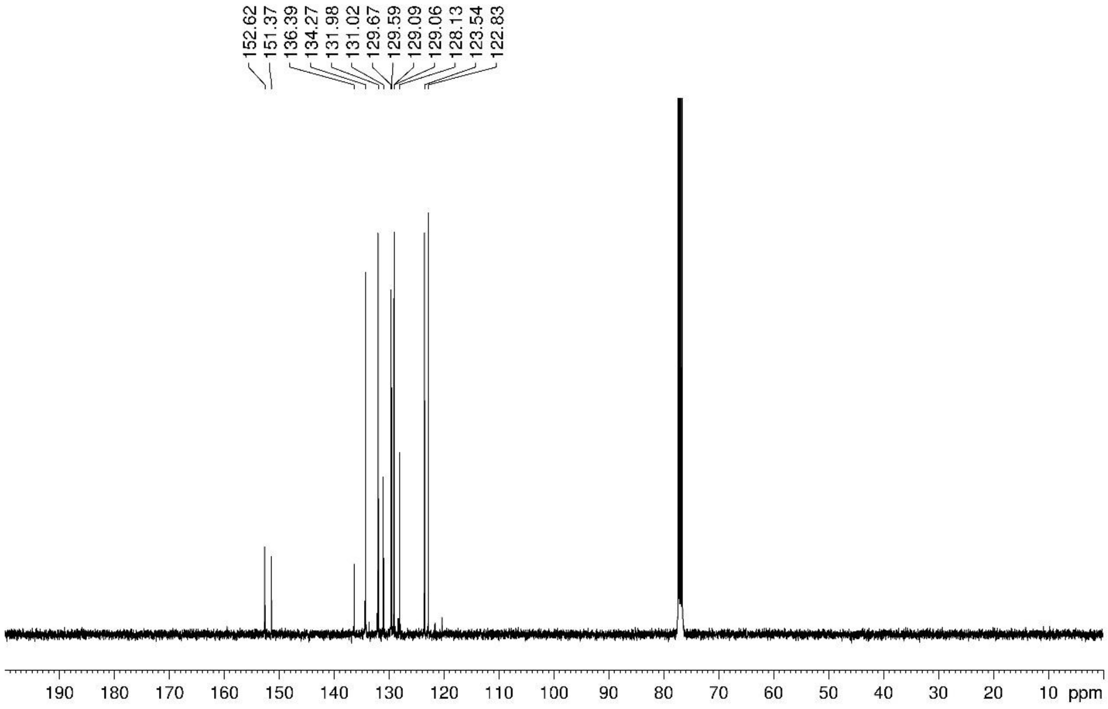
152.62
151.37
136.39
134.27
131.98
131.02
129.67
129.59
129.09
129.06
128.13
123.54
122.83
190 180 170 160 150 140 130 120 110 100 90 80 70 60 50 40 30 20 10 ppm

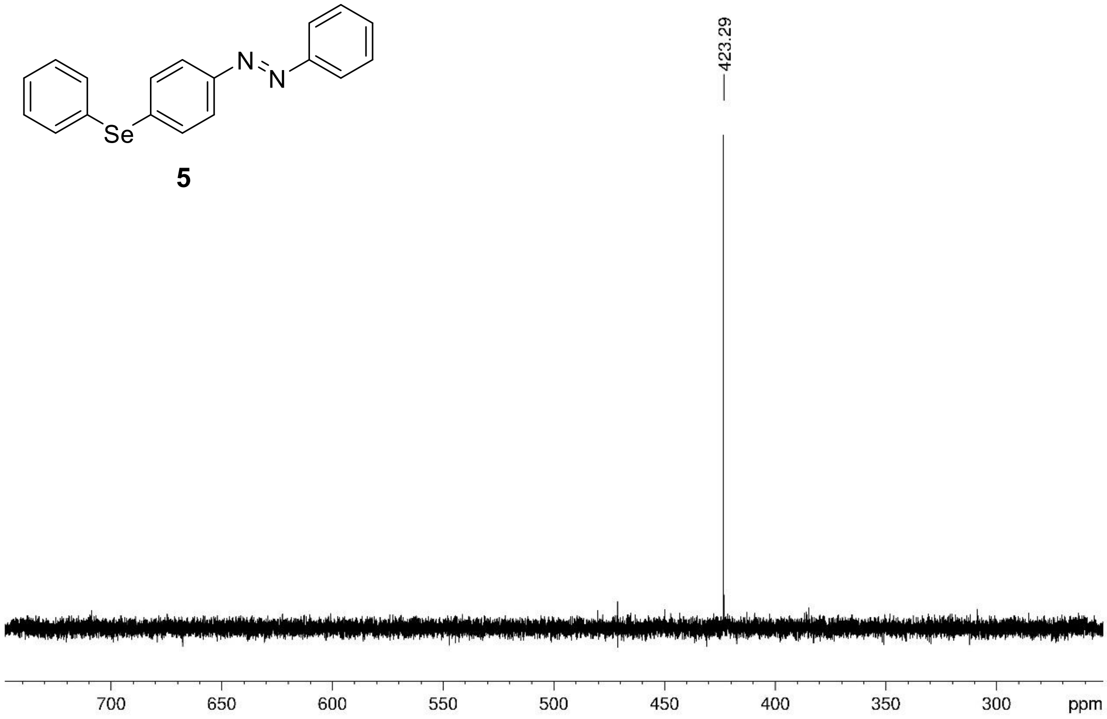
Se
N
N
5
423.29
700
650
600
550
500
450
400
350
300
ppm

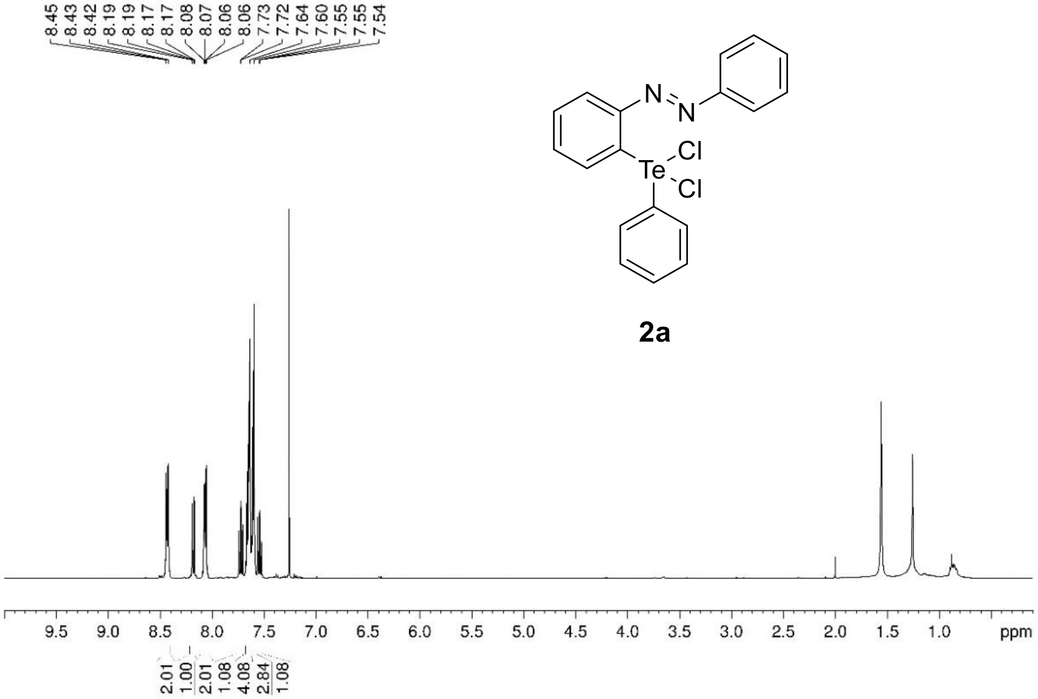

2a

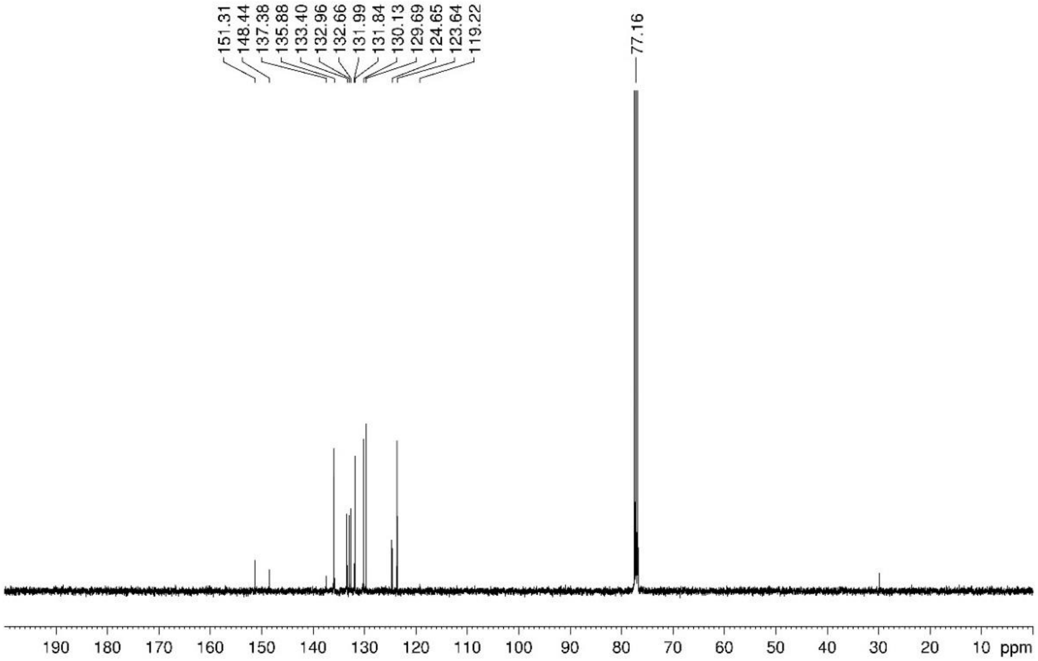

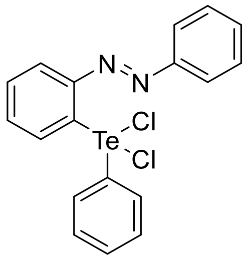

2a

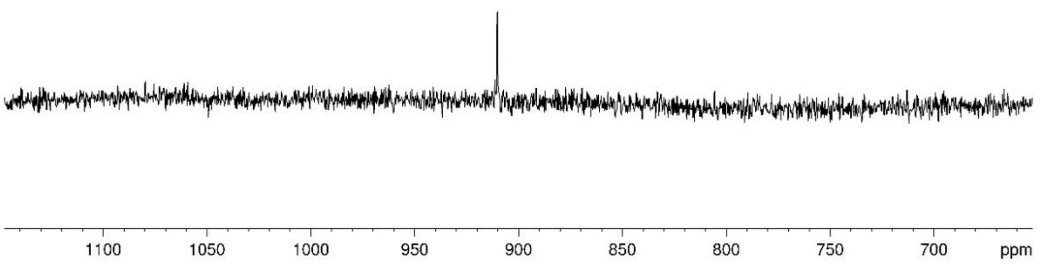

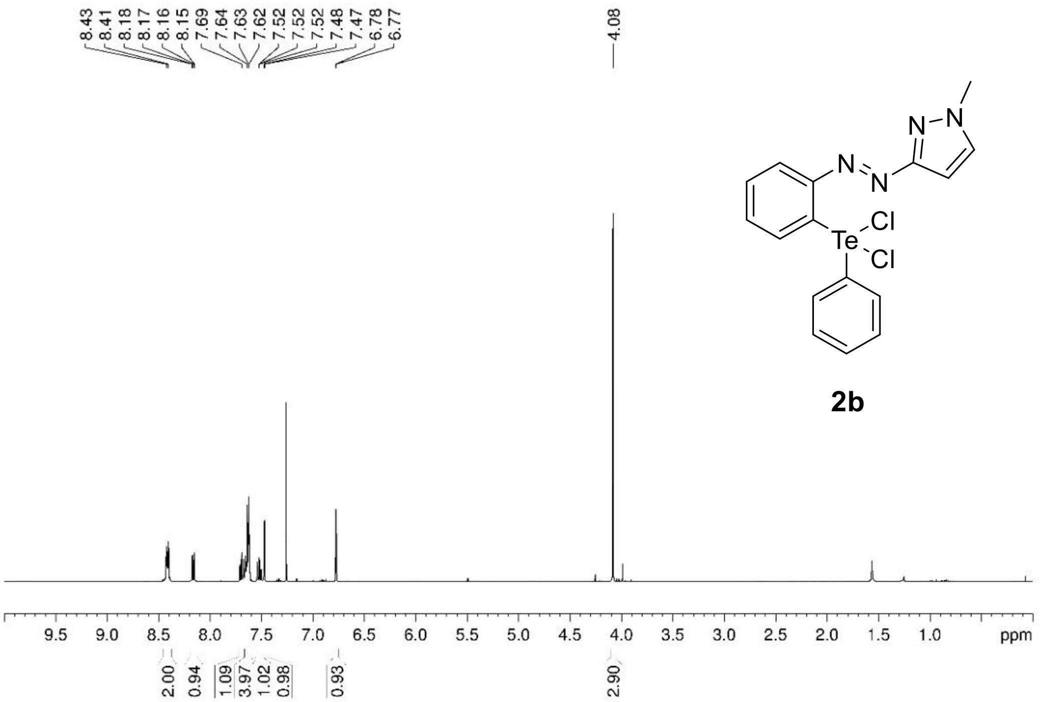
8.43
8.41
8.18
8.17
8.16
8.15
7.69
7.64
7.63
7.62
7.52
7.52
7.48
7.47
6.78
6.77
4.08
2.00
0.94
1.09
3.97
1.02
0.98
0.93
2.90
ppm
N
N
N
N
Te
Cl
Cl

2b

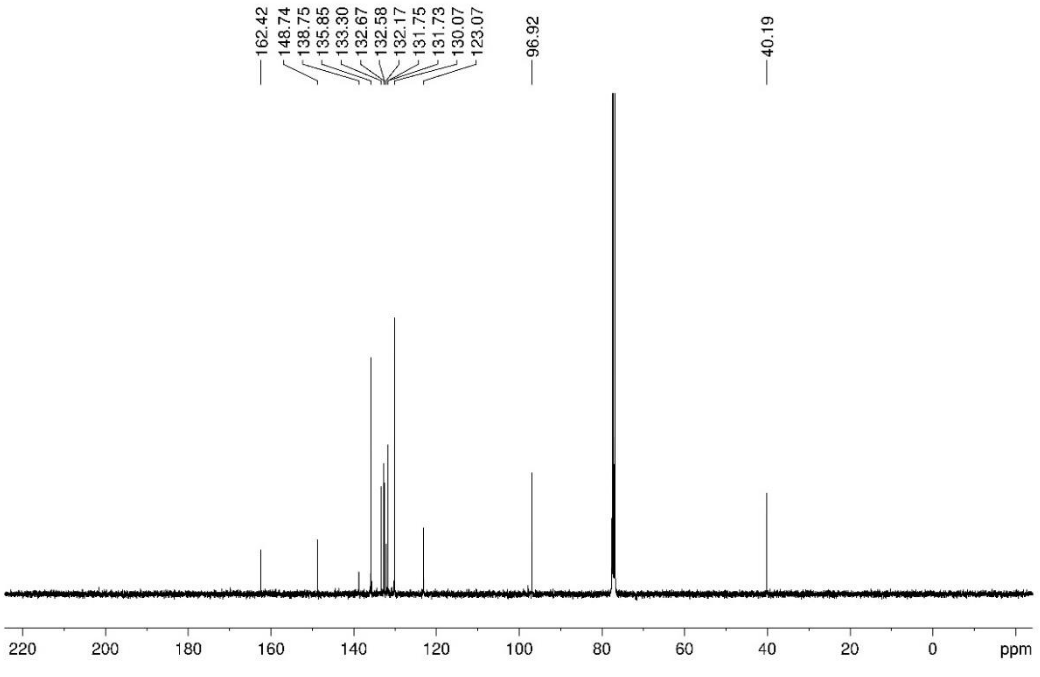
162.42
148.74
138.75
135.85
133.30
132.67
132.58
132.17
131.75
131.73
130.07
123.07
96.92
40.19
ppm

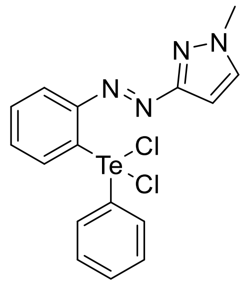
N
N
N
N
Te
Cl
Cl

2b

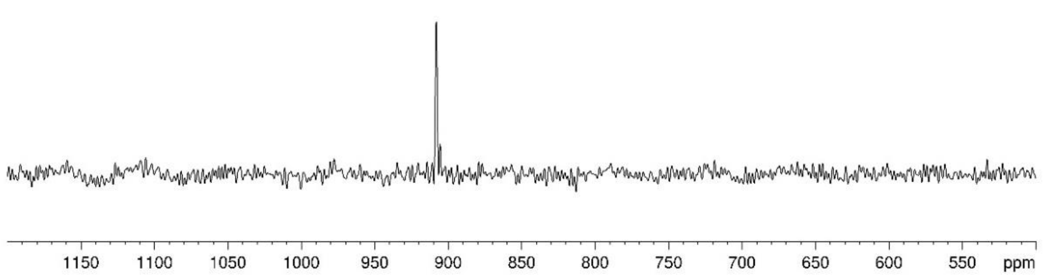
908.10
1150
1100
1050
1000
950
900
850
800
750
700
650
600
550
ppm

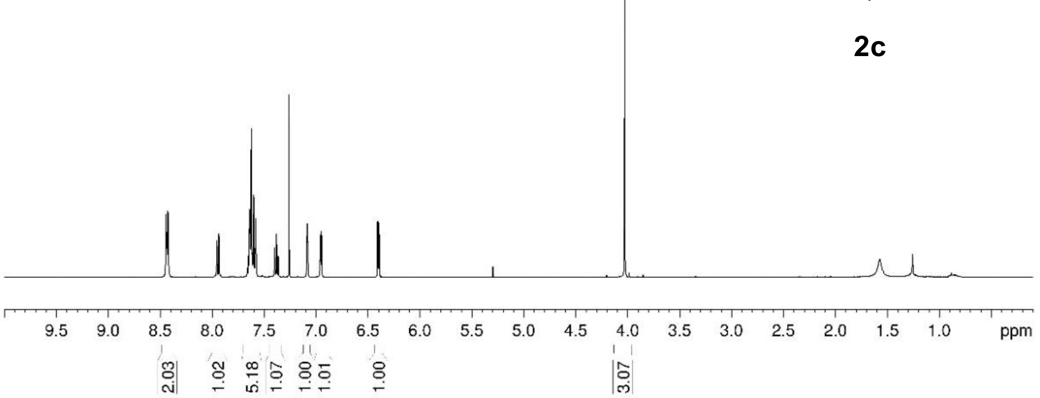

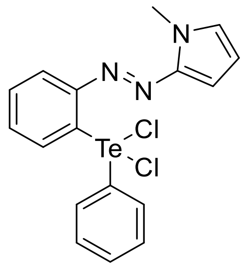

2c

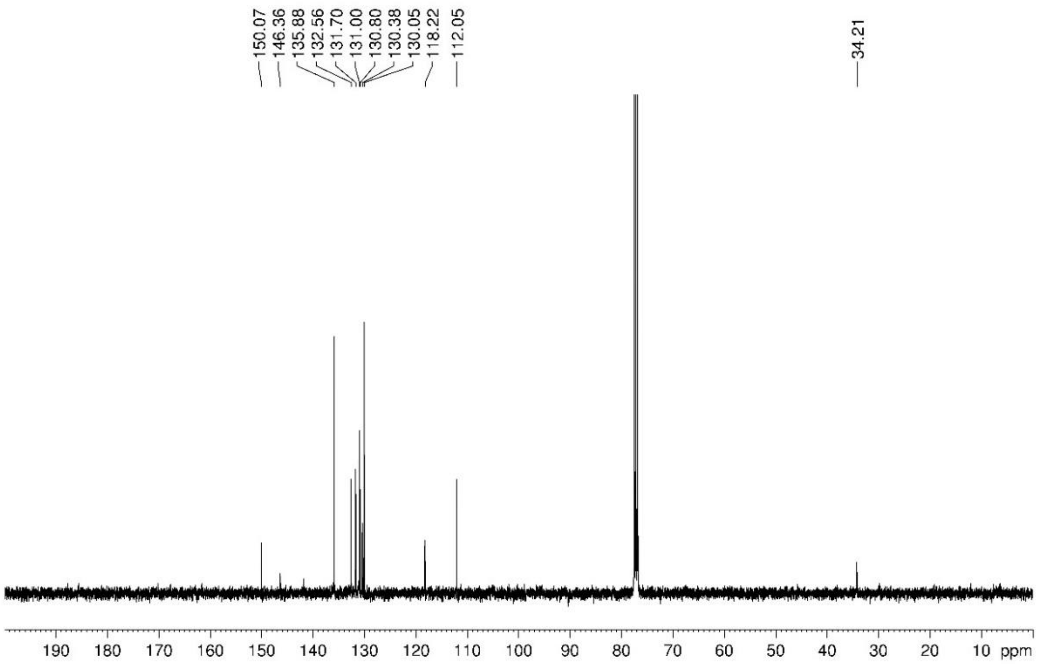

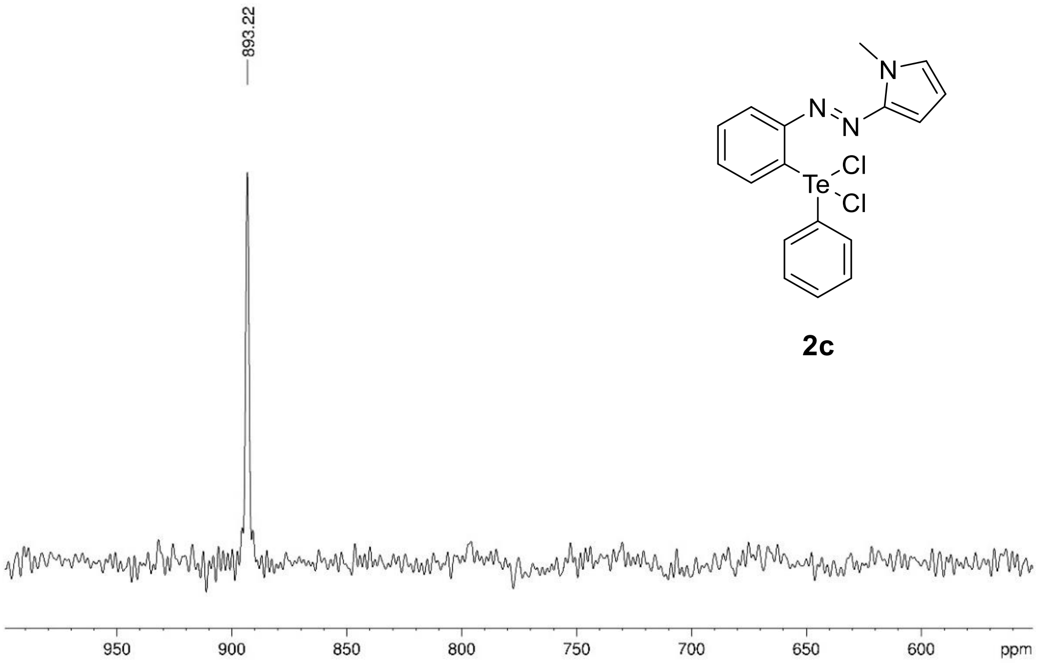

2c

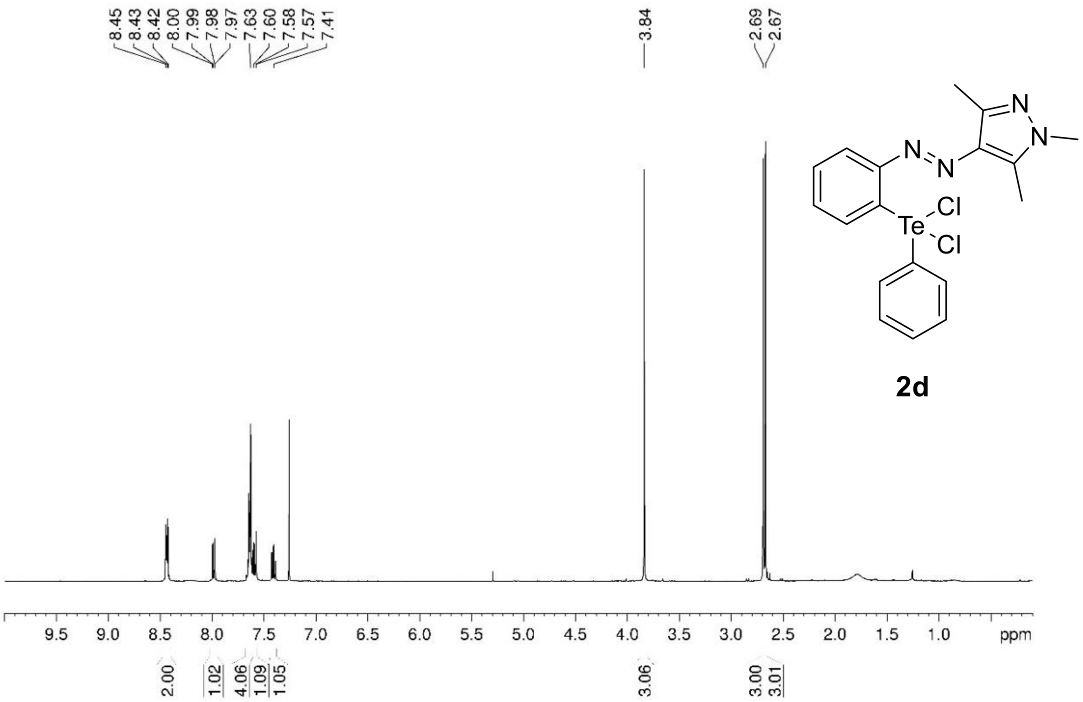
8.45
8.43
8.42
8.00
7.99
7.98
7.97
7.63
7.60
7.58
7.57
7.41
3.84
2.69
2.67
9.5 9.0 8.5 8.0 7.5 7.0 6.5 6.0 5.5 5.0 4.5 4.0 3.5 3.0 2.5 2.0 1.5 1.0 ppm
2.00
1.02
4.06
1.09
1.05
3.06
3.00
3.01
N
N
N
N
Te
Cl
Cl

2d

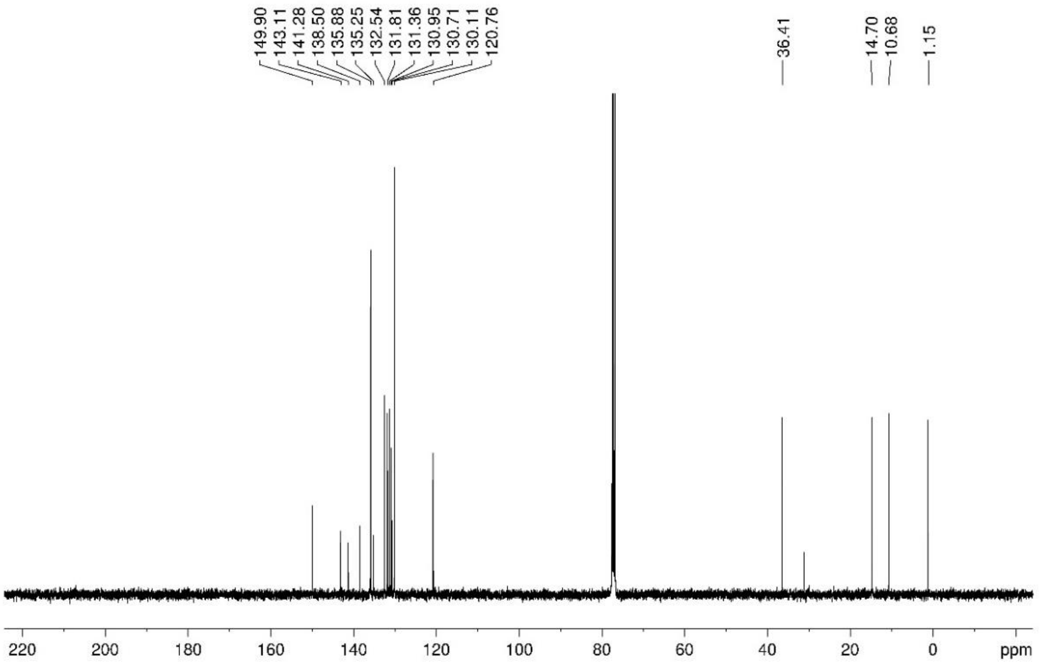
149.90
143.11
141.28
138.50
135.88
135.25
132.54
131.81
131.36
130.95
130.71
130.11
120.76
36.41
14.70
10.68
1.15
220 200 180 160 140 120 100 80 60 40 20 0 ppm

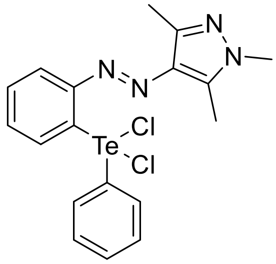

2d

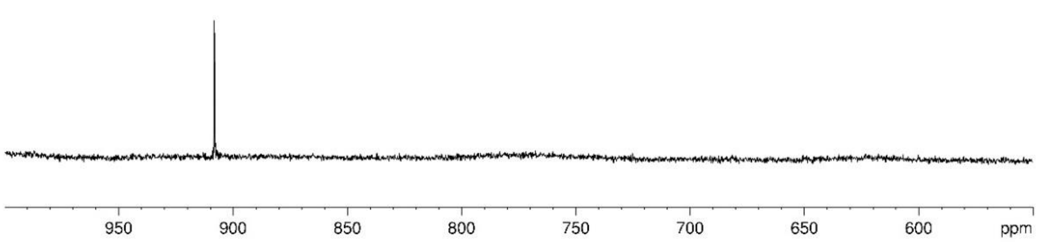

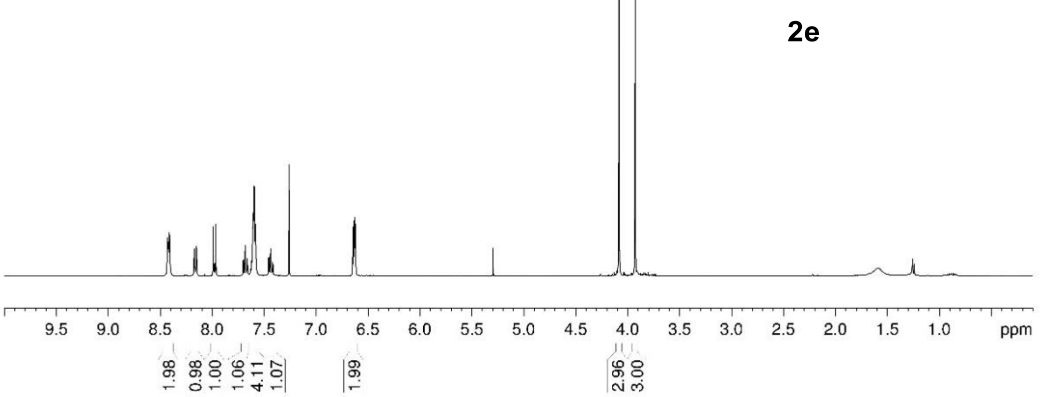
8.43
8.41
8.41
8.17
8.17
8.15
8.15
7.99
7.97
7.68
7.60
7.59
7.44
6.63
6.63
4.08
3.93
9.5 9.0 8.5 8.0 7.5 7.0 6.5 6.0 5.5 5.0 4.5 4.0 3.5 3.0 2.5 2.0 1.5 1.0 ppm
1.98
0.98
1.00
1.06
4.11
1.07
1.99
2.96
3.00

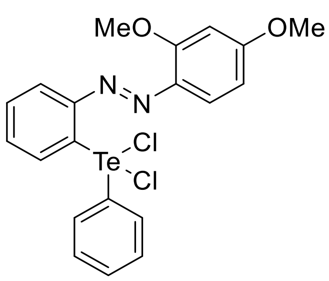
MeO
OMe
N
N
Te
Cl
Cl

2e

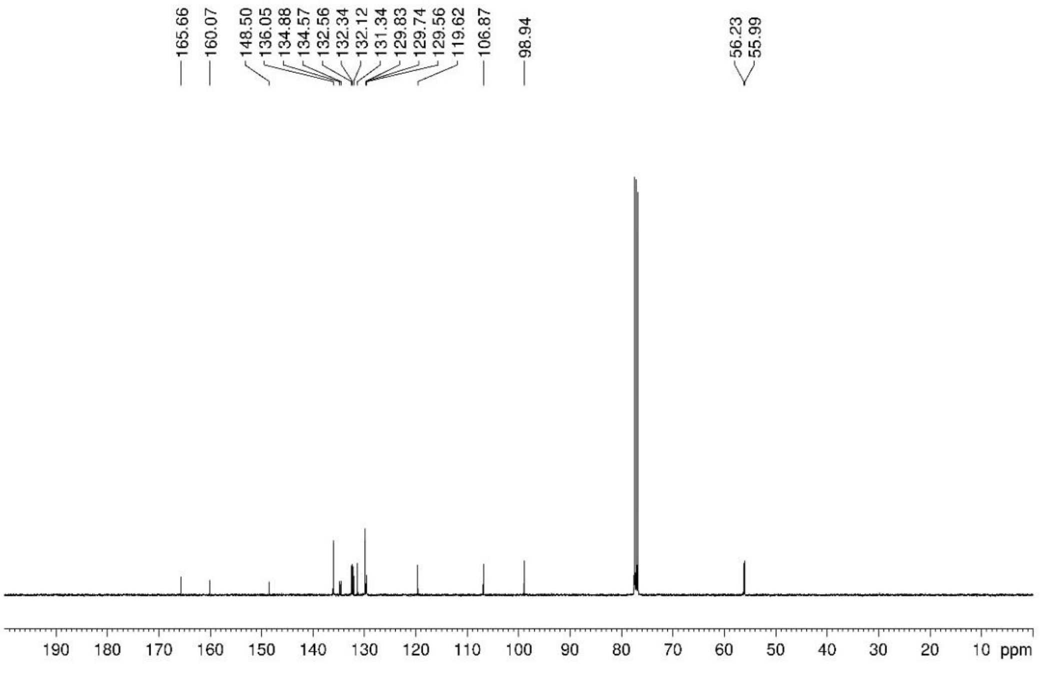
165.66
160.07
148.50
136.05
134.88
134.57
132.56
132.34
132.12
131.34
129.83
129.74
129.56
119.62
106.87
98.94
56.23
55.99
190 180 170 160 150 140 130 120 110 100 90 80 70 60 50 40 30 20 10 ppm

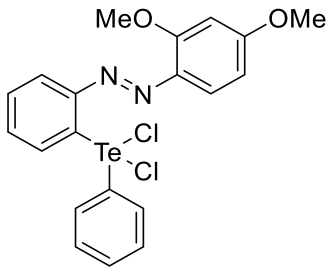

2e

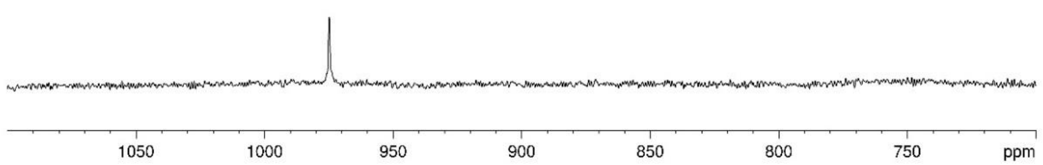

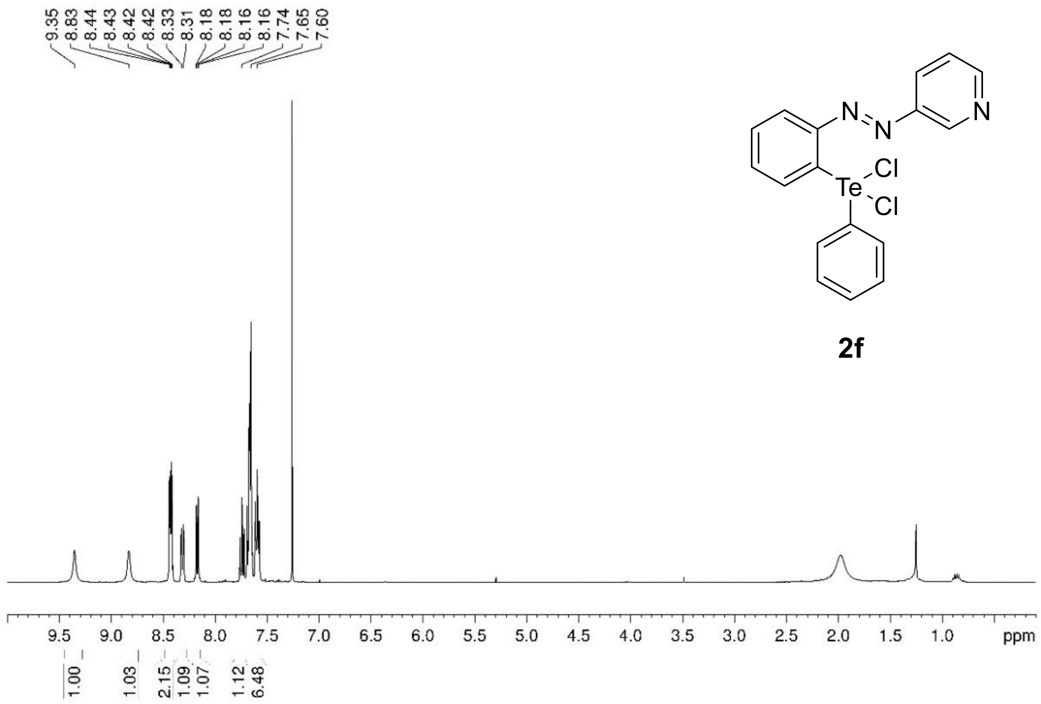
9.35
8.83
8.44
8.43
8.42
8.42
8.33
8.31
8.18
8.18
8.16
8.16
7.74
7.65
7.60
N
N
N
Te
Cl
Cl
9.5 9.0 8.5 8.0 7.5 7.0 6.5 6.0 5.5 5.0 4.5 4.0 3.5 3.0 2.5 2.0 1.5 1.0 ppm
1.00
1.03
2.15
1.09
1.07
1.12
6.48

2f

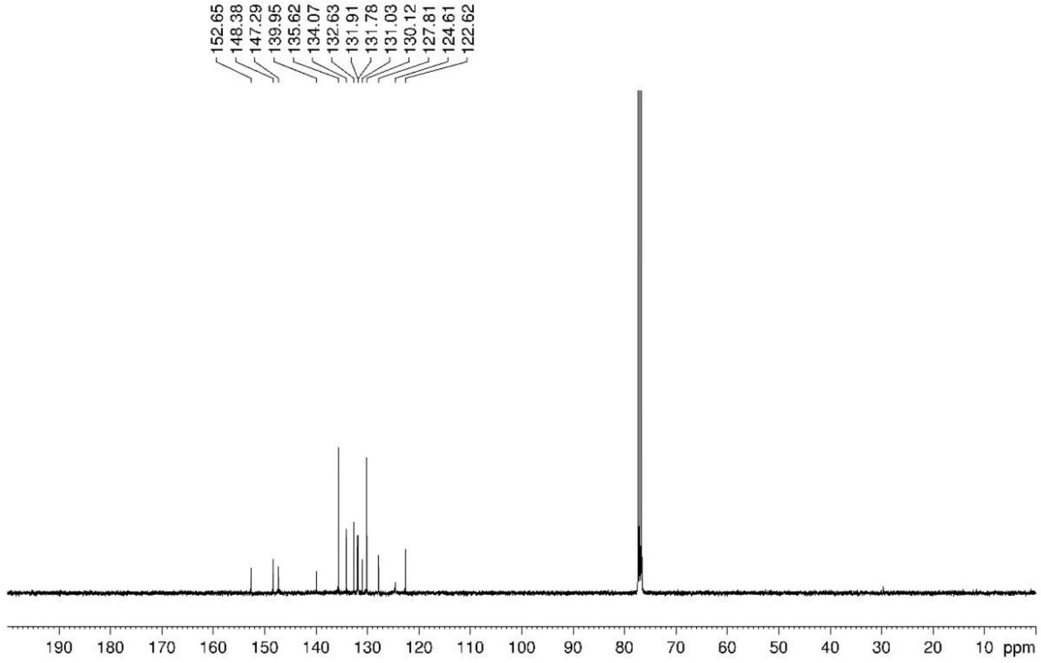
152.65
148.38
147.29
139.95
135.62
134.07
132.63
131.91
131.78
131.03
130.12
127.81
124.61
122.62
190 180 170 160 150 140 130 120 110 100 90 80 70 60 50 40 30 20 10 ppm

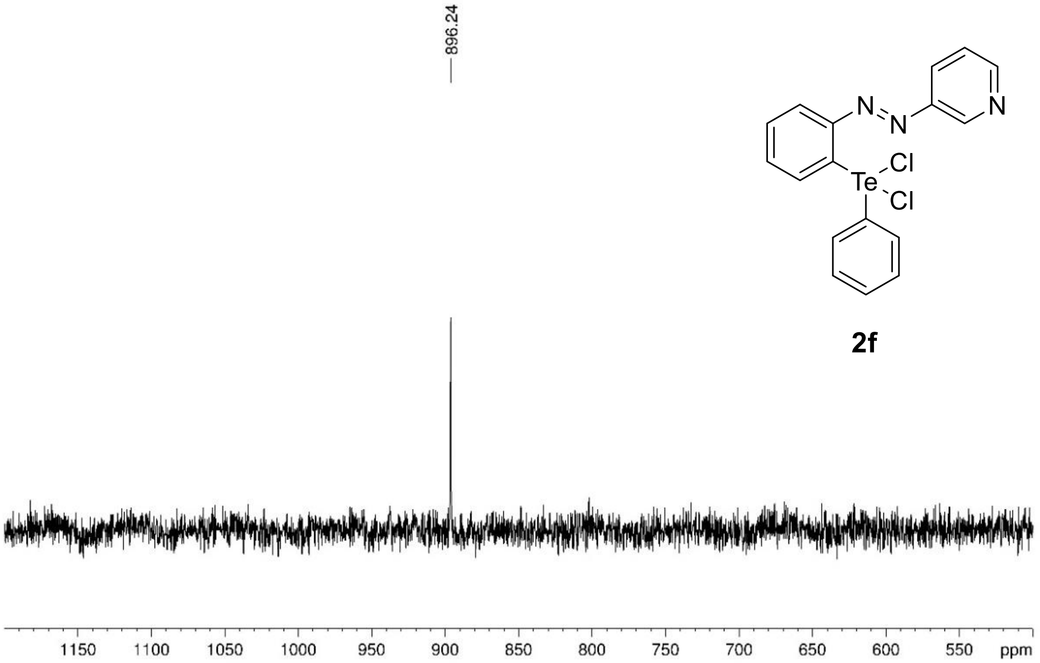
896.24
N
N
N
Te
Cl
Cl
1150
1100
1050
1000
950
900
850
800
750
700
650
600
550
ppm

2f

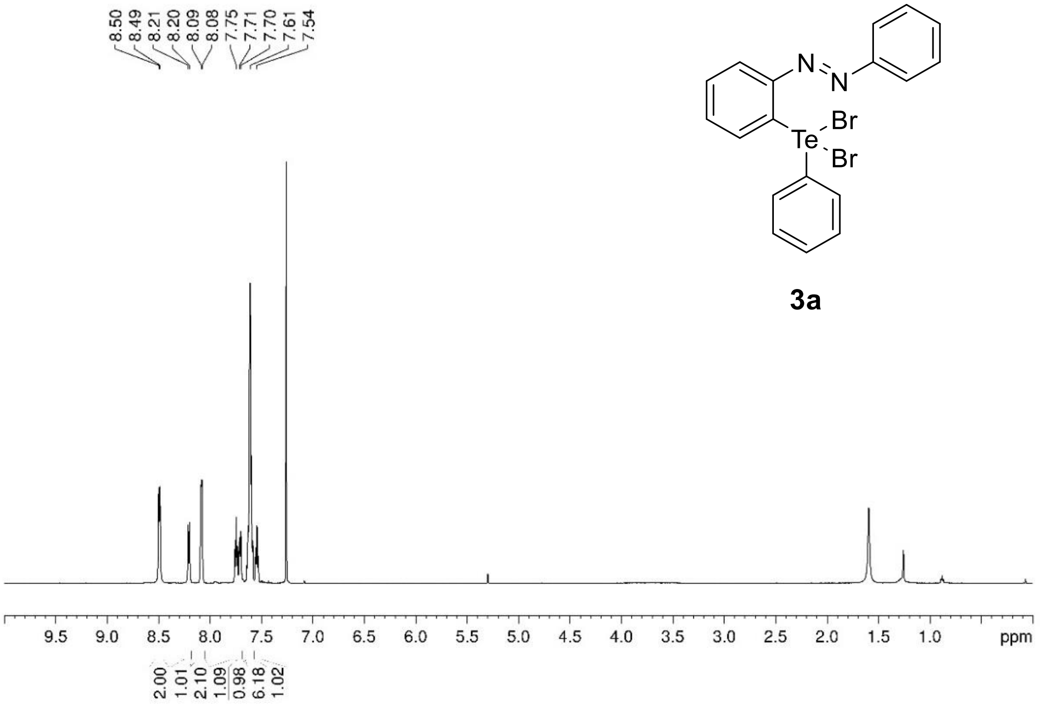
8.50
8.49
8.21
8.20
8.09
8.08
7.75
7.71
7.70
7.61
7.54
N
N
Te
Br
Br
9.5 9.0 8.5 8.0 7.5 7.0 6.5 6.0 5.5 5.0 4.5 4.0 3.5 3.0 2.5 2.0 1.5 1.0 ppm
2.00
1.01
2.10
1.09
0.98
6.18
1.02

3a

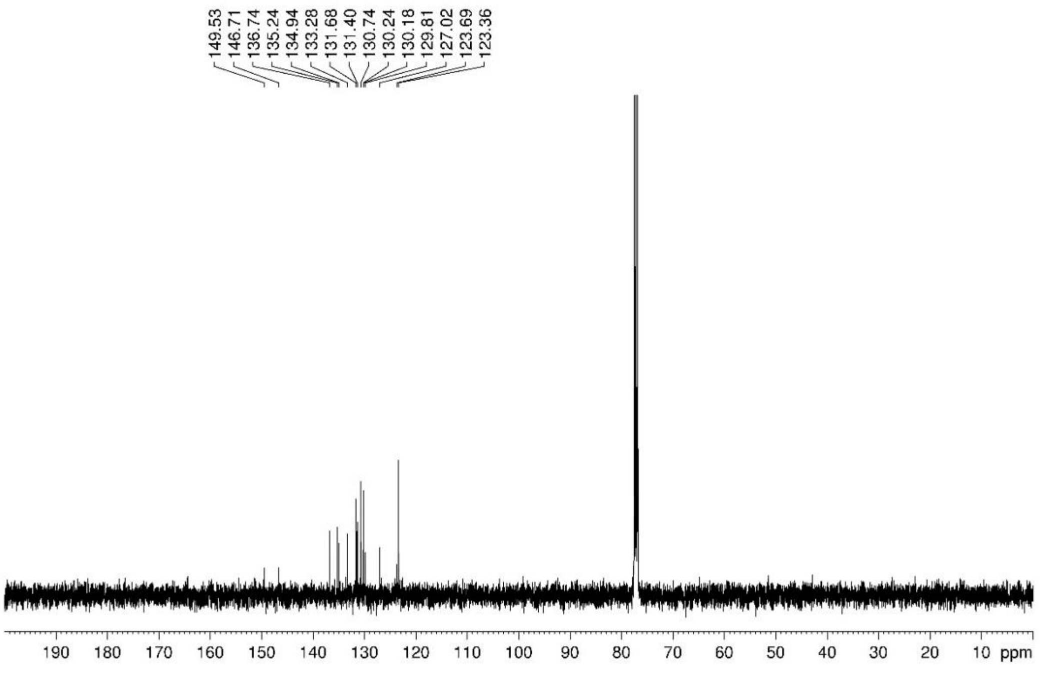
149.53
146.71
136.74
135.24
134.94
133.28
131.68
131.40
130.74
130.24
130.18
129.81
127.02
123.69
123.36
190 180 170 160 150 140 130 120 110 100 90 80 70 60 50 40 30 20 10 ppm

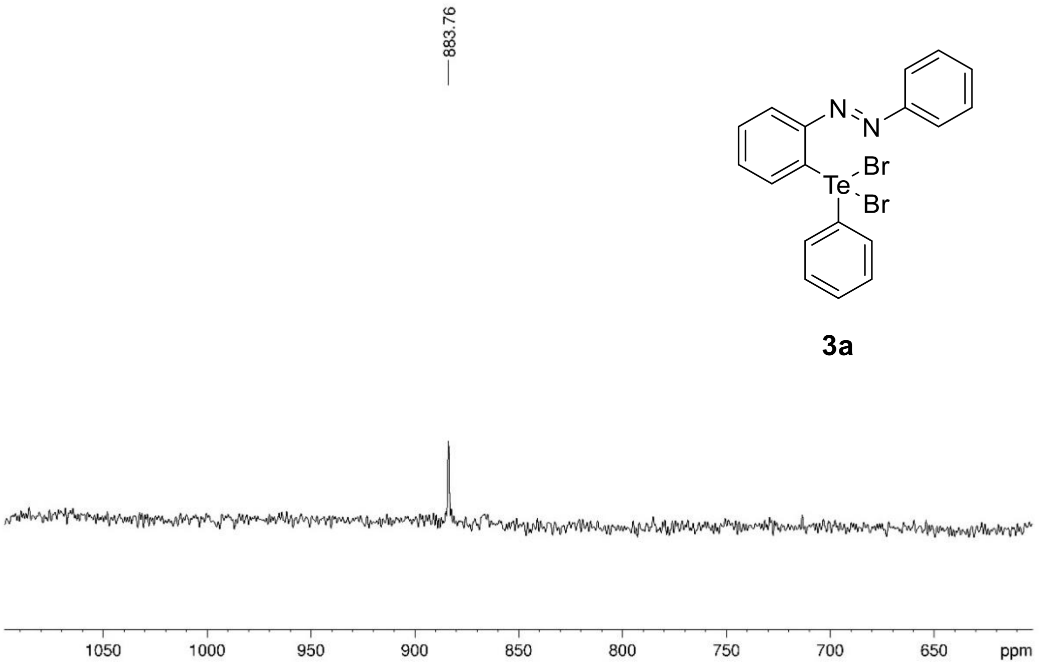

3a

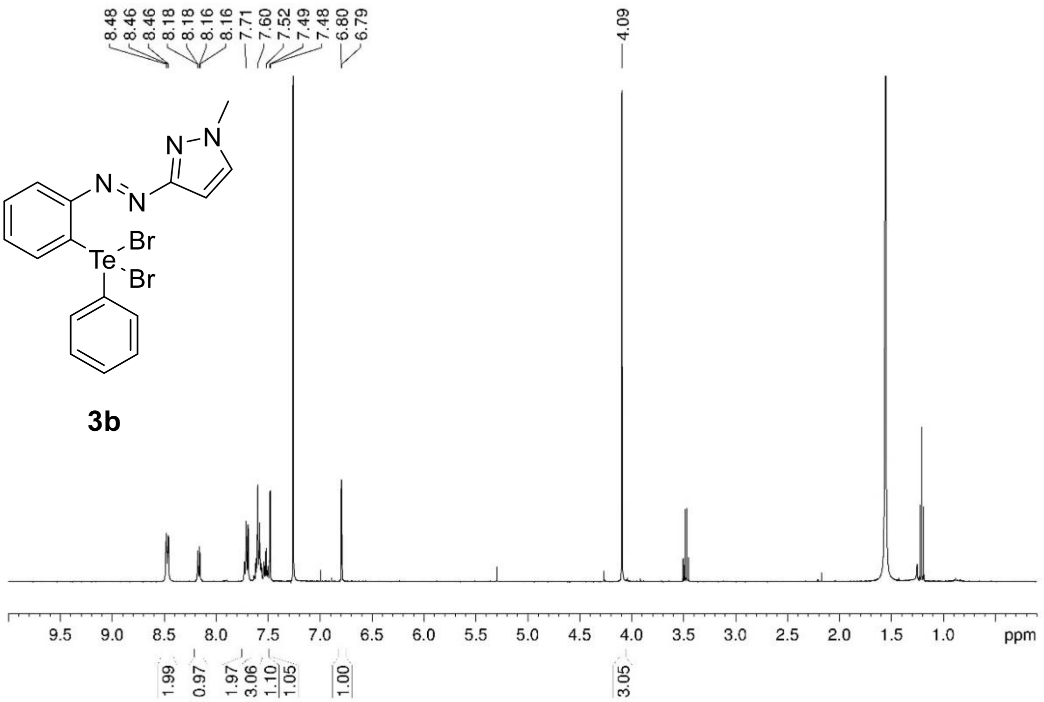

3b

8.48
8.46
8.46
8.18
8.18
8.16
8.16
7.71
7.60
7.52
7.49
7.48
6.80
6.79
4.09
9.5 9.0 8.5 8.0 7.5 7.0 6.5 6.0 5.5 5.0 4.5 4.0 3.5 3.0 2.5 2.0 1.5 1.0 ppm
1.99
0.97
1.97
3.06
1.10
1.05
1.00
3.05

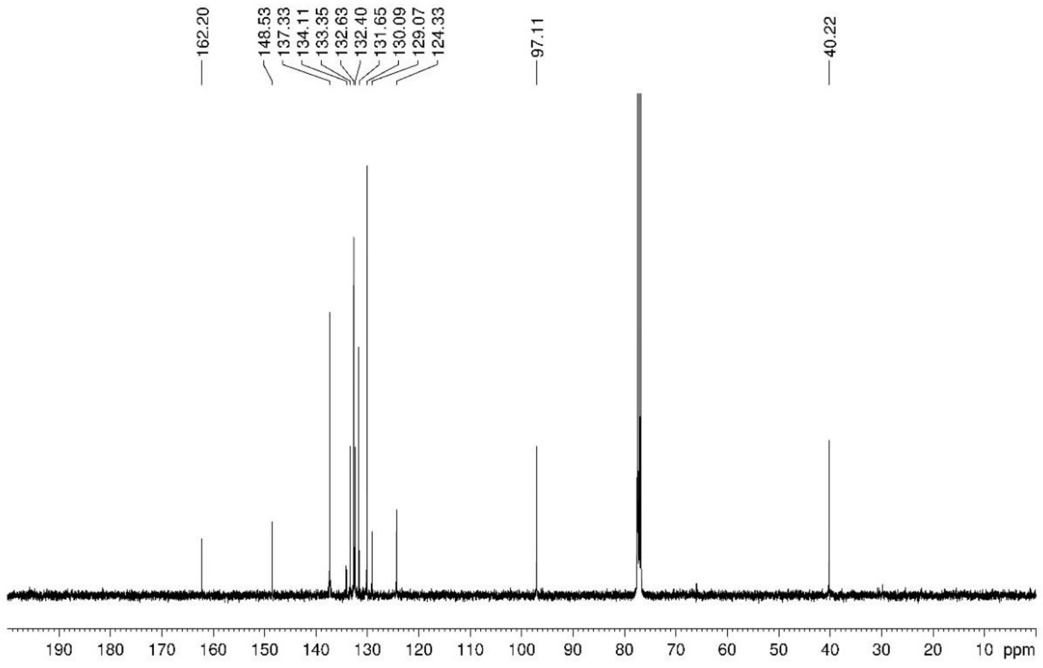

162.20
148.53
137.33
134.11
133.35
132.63
132.40
131.65
130.09
129.07
124.33
97.11
40.22
190 180 170 160 150 140 130 120 110 100 90 80 70 60 50 40 30 20 10 ppm

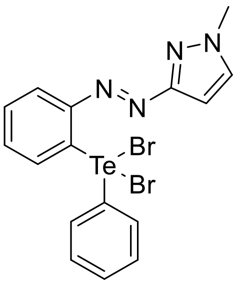

3b

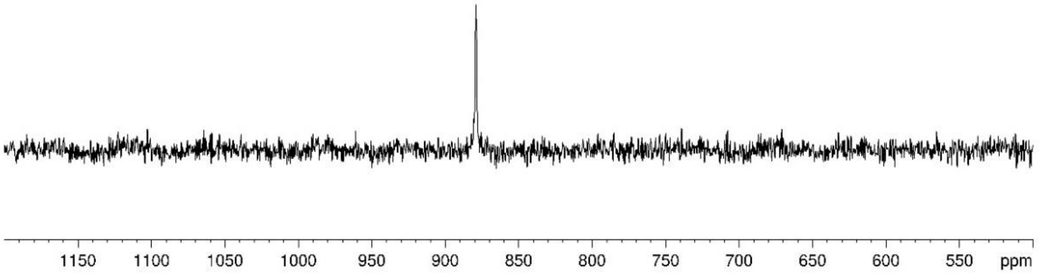

878.99
1150
1100
1050
1000
950
900
850
800
750
700
650
600
550
ppm

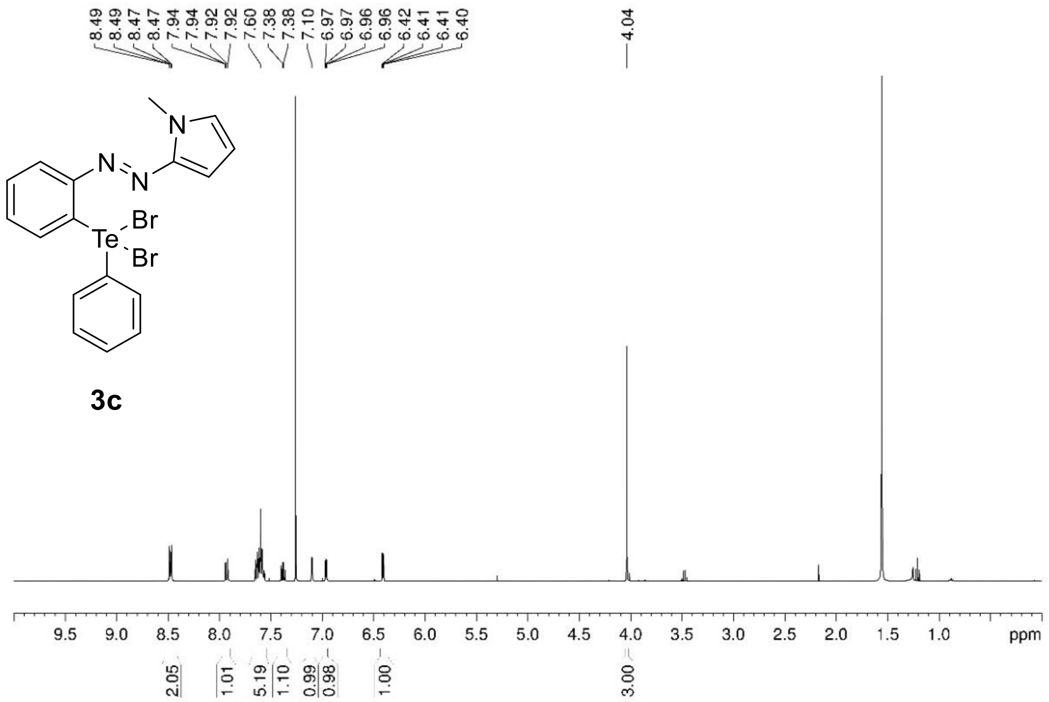
N
N
N
Te
Br
Br
3c
8.49
8.49
8.47
8.47
7.94
7.94
7.92
7.92
7.60
7.38
7.38
7.10
6.97
6.97
6.96
6.96
6.42
6.41
6.41
6.40
4.04
9.5 9.0 8.5 8.0 7.5 7.0 6.5 6.0 5.5 5.0 4.5 4.0 3.5 3.0 2.5 2.0 1.5 1.0 ppm
2.05
1.01
5.19
1.10
0.99
0.98
1.00
3.00

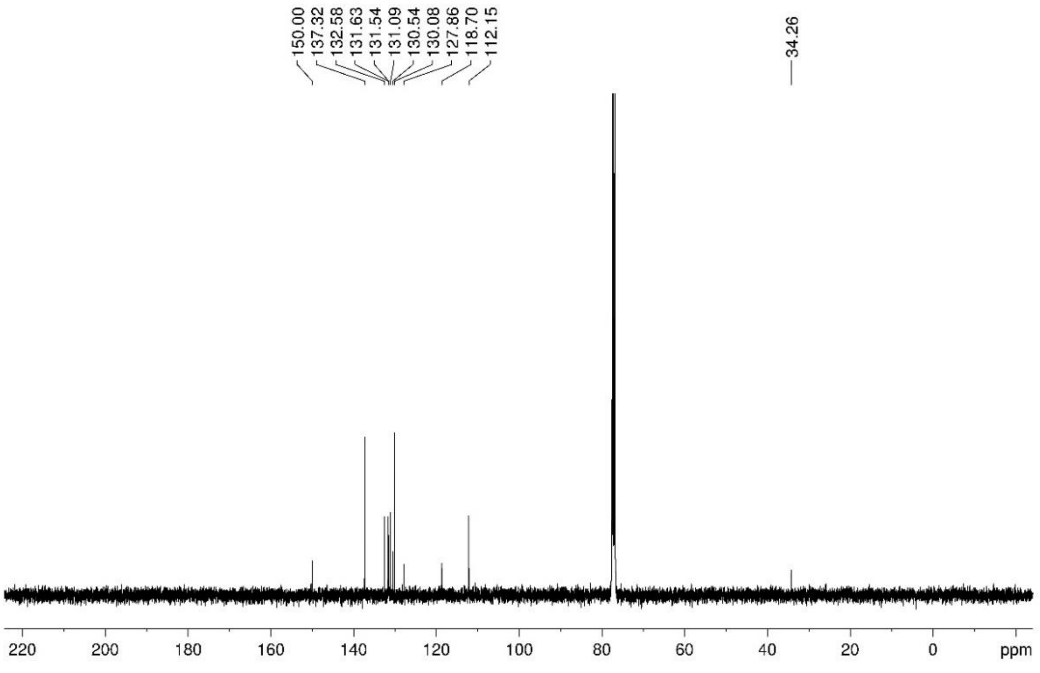
150.00
137.32
132.58
131.63
131.54
131.09
130.54
130.08
127.86
118.70
112.15
34.26
220 200 180 160 140 120 100 80 60 40 20 0 ppm

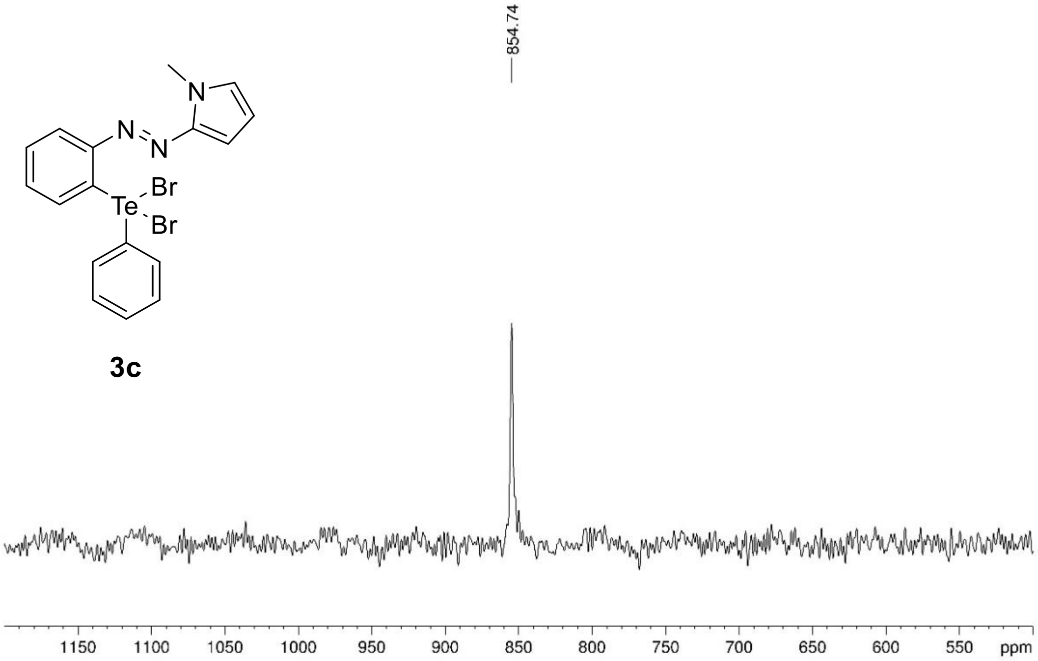

N
N
N
Te
Br
Br
854.74
1150
1100
1050
1000
950
900
850
800
750
700
650
600
550
ppm

3c

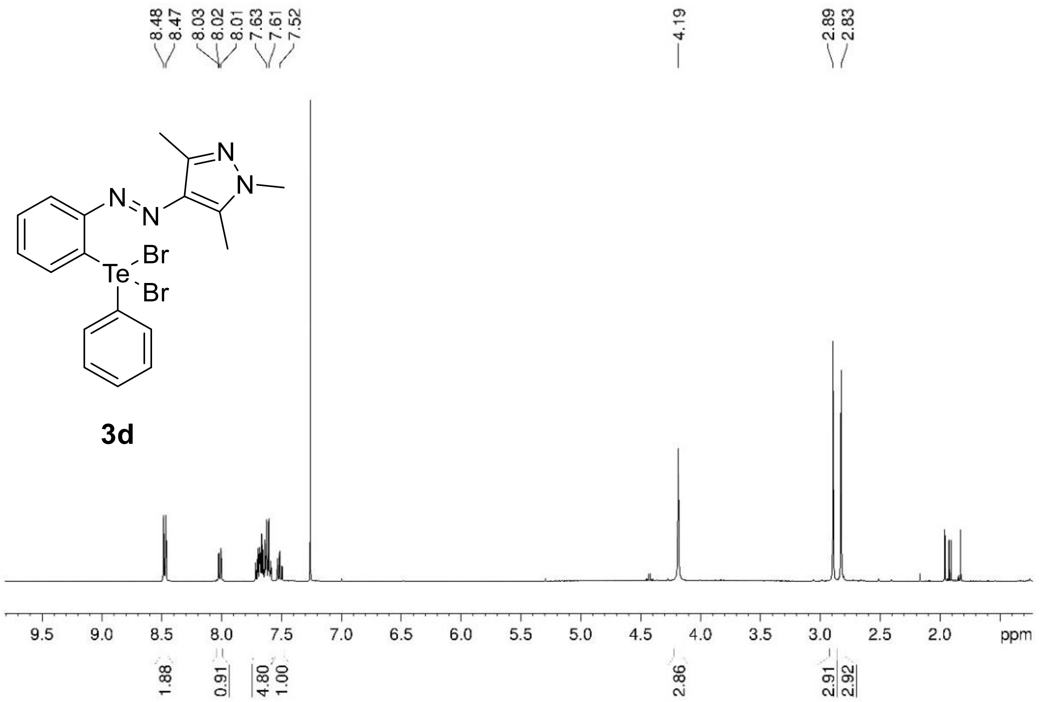
N
N
N
N
Te
Br
Br
3d
8.48
8.47
8.03
8.02
8.01
7.63
7.61
7.52
4.19
2.89
2.83
9.5 9.0 8.5 8.0 7.5 7.0 6.5 6.0 5.5 5.0 4.5 4.0 3.5 3.0 2.5 2.0 ppm
1.88
0.91
4.80
1.00
2.86
2.91
2.92

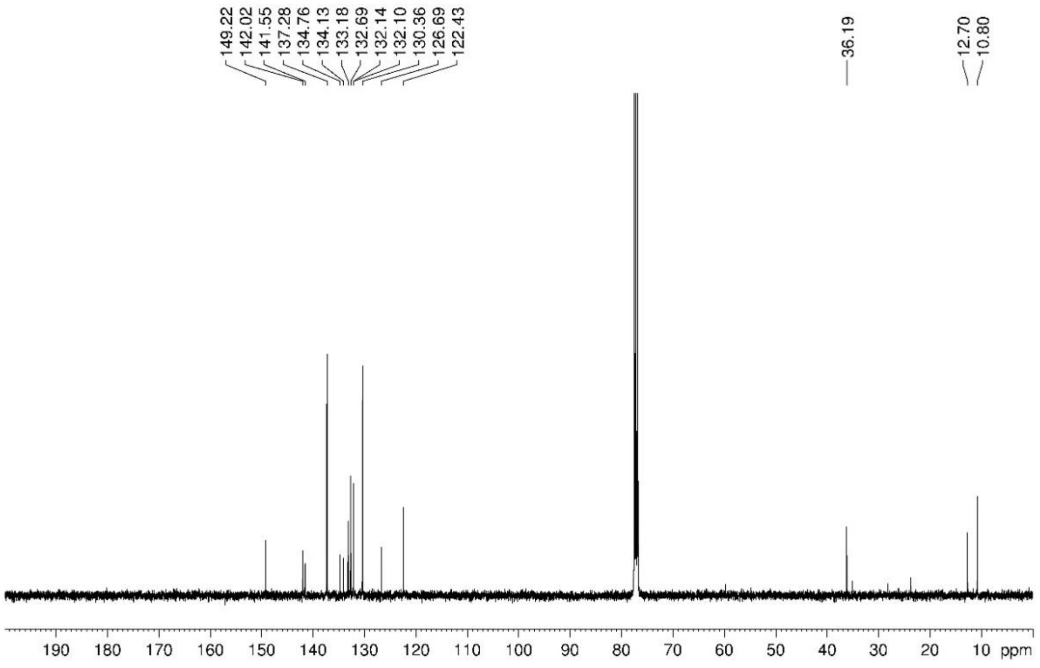
149.22
142.02
141.55
137.28
134.76
134.13
133.18
132.69
132.14
132.10
130.36
126.69
122.43
36.19
12.70
10.80
190 180 170 160 150 140 130 120 110 100 90 80 70 60 50 40 30 20 10 ppm

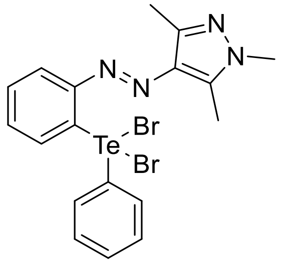
N
N
N
N
Te
Br
Br

3d

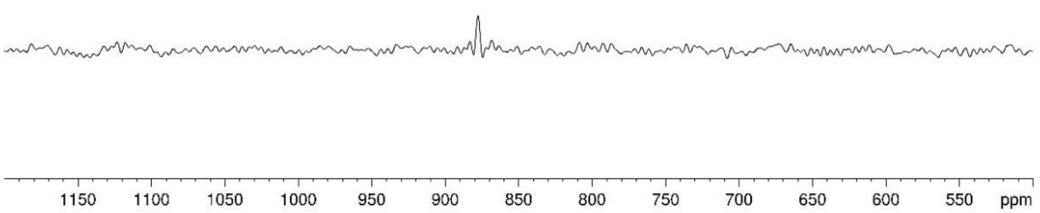
877.68
1150
1100
1050
1000
950
900
850
800
750
700
650
600
550
ppm

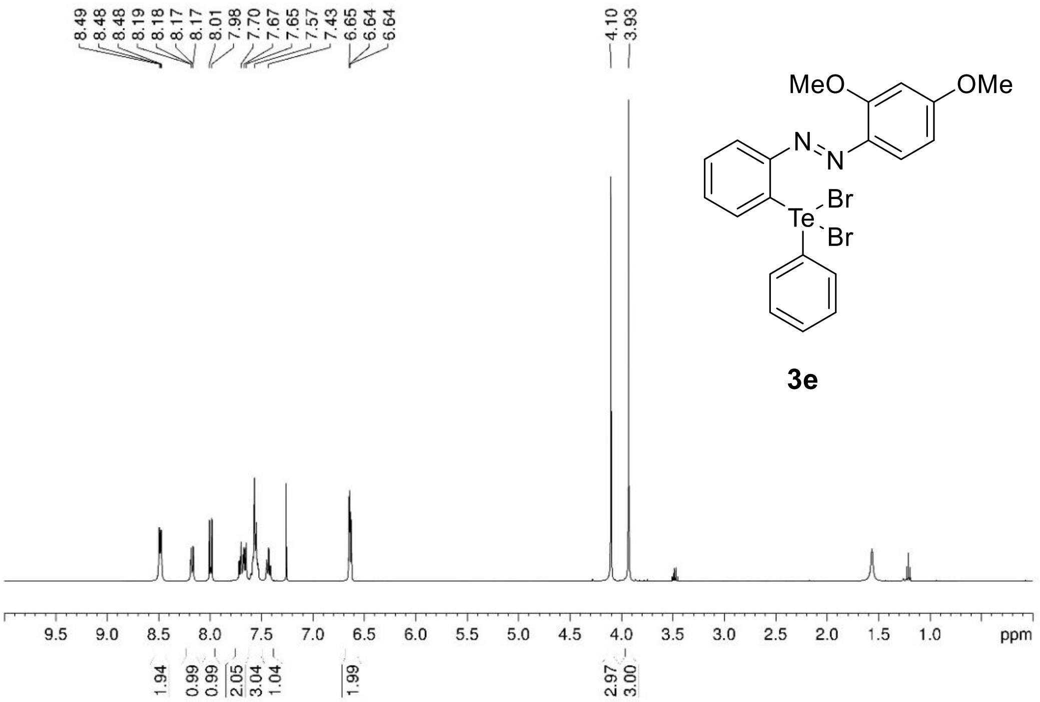
8.49
8.48
8.48
8.19
8.18
8.17
8.17
8.01
7.98
7.70
7.67
7.65
7.57
7.43
6.65
6.64
6.64
4.10
3.93
9.5 9.0 8.5 8.0 7.5 7.0 6.5 6.0 5.5 5.0 4.5 4.0 3.5 3.0 2.5 2.0 1.5 1.0 ppm
1.94
0.99
0.99
2.05
3.04
1.04
1.99
2.97
3.00
MeO
OMe
N
N
Te
Br
Br

3e

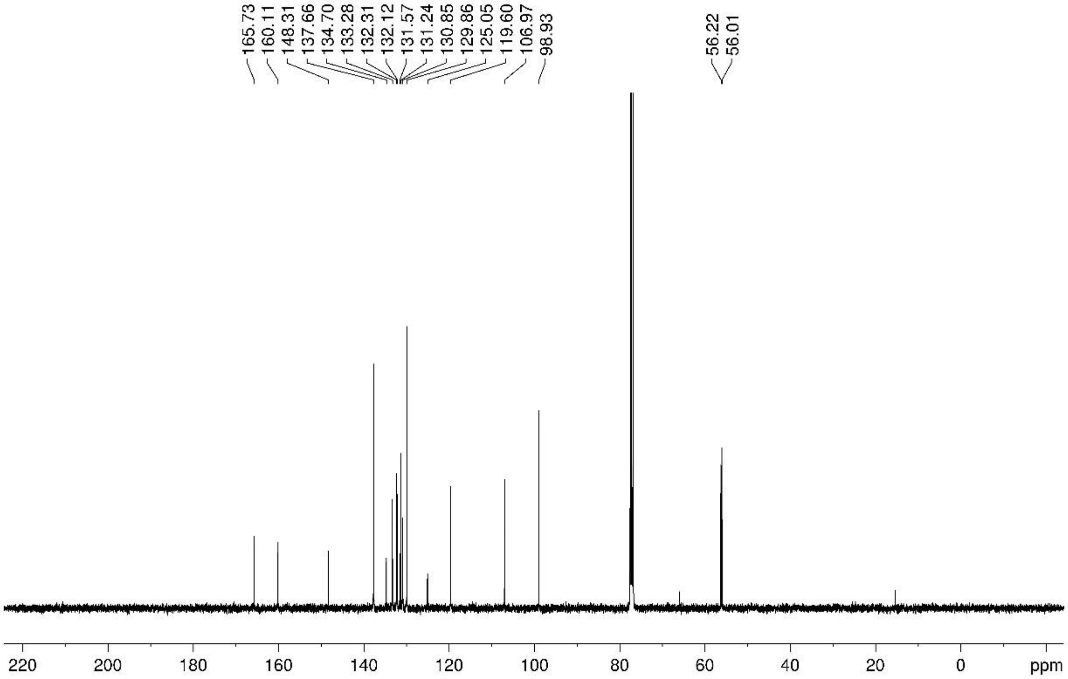
165.73
160.11
148.31
137.66
134.70
133.28
132.31
132.12
131.57
131.24
130.85
129.86
125.05
119.60
106.97
98.93
56.22
56.01
220 200 180 160 140 120 100 80 60 40 20 0 ppm

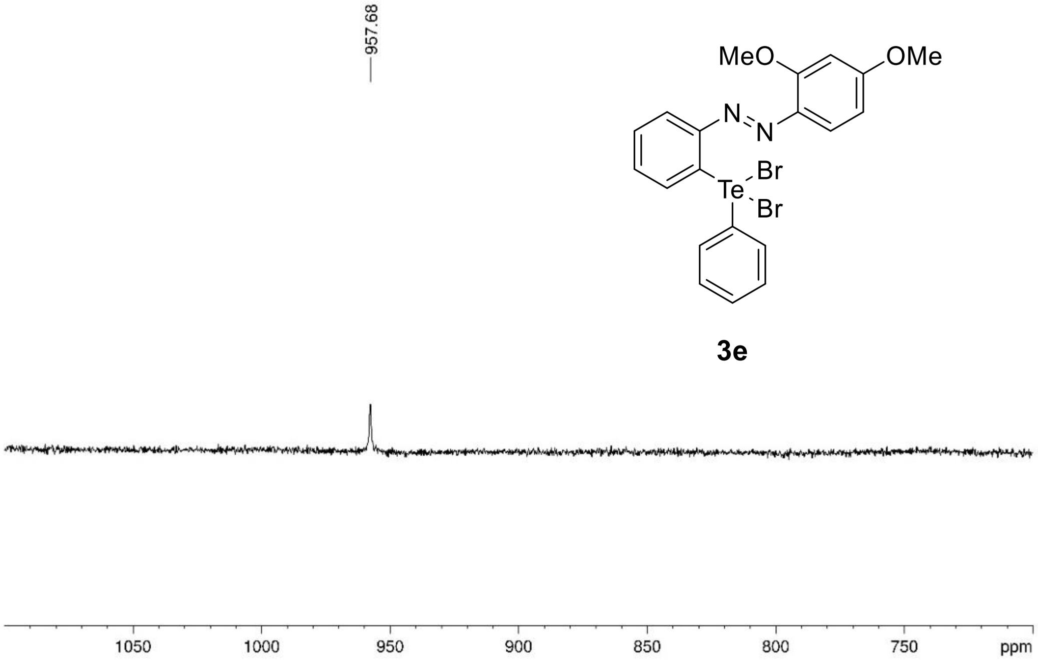

3e

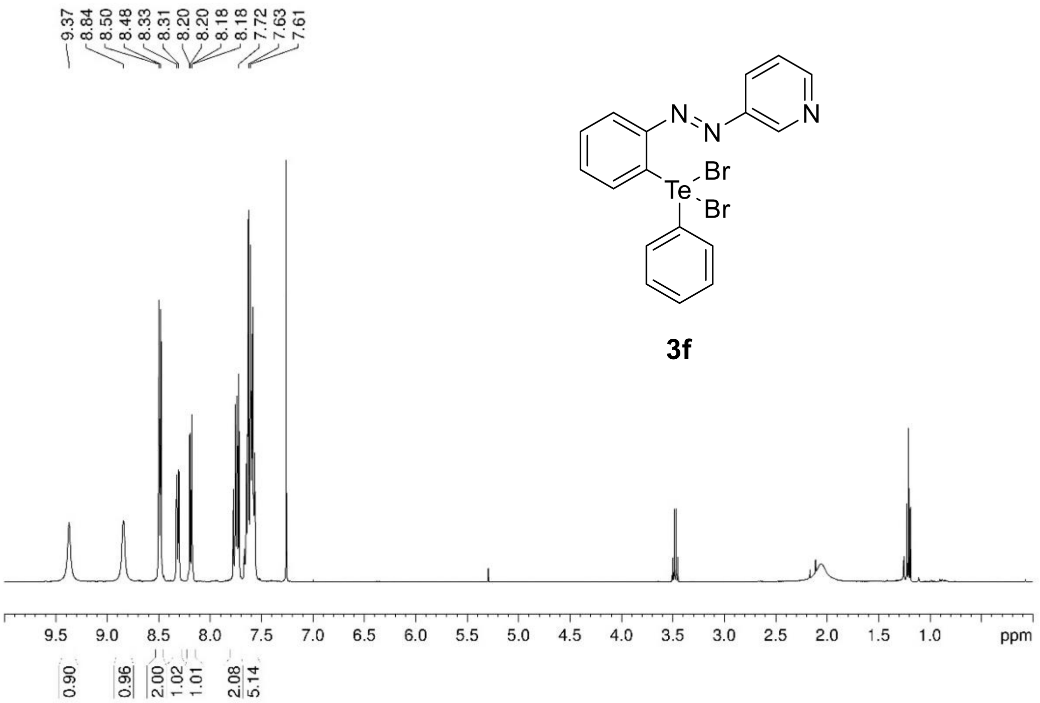
9.37
8.84
8.50
8.48
8.33
8.31
8.20
8.20
8.18
8.18
7.72
7.63
7.61
9.5 9.0 8.5 8.0 7.5 7.0 6.5 6.0 5.5 5.0 4.5 4.0 3.5 3.0 2.5 2.0 1.5 1.0 ppm
0.90
0.96
2.00
1.02
1.01
2.08
5.14
N
N
N
Br
Te
Br

3f

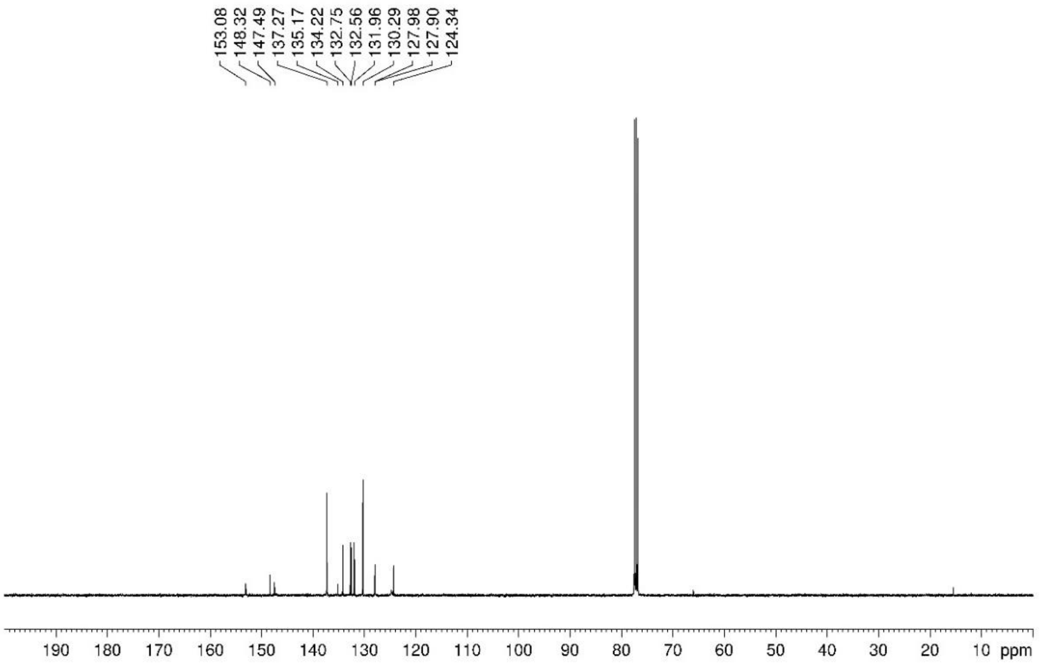
153.08
148.32
147.49
137.27
135.17
134.22
132.75
132.56
131.96
130.29
127.98
127.90
124.34
190 180 170 160 150 140 130 120 110 100 90 80 70 60 50 40 30 20 10 ppm

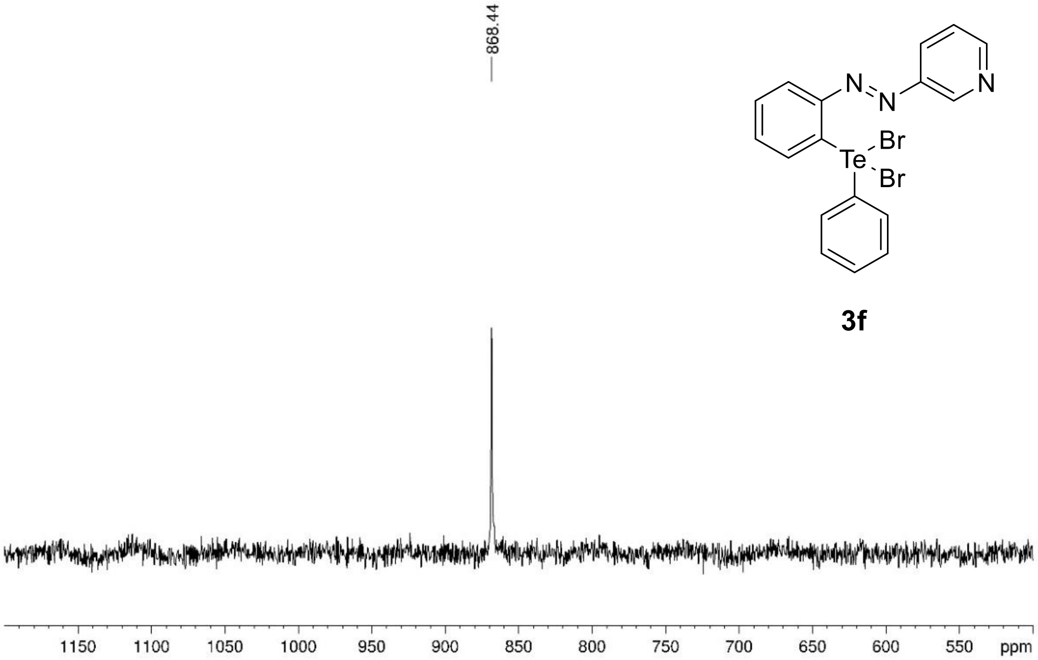
868.44
N
N
N
Te
Br
Br
1150
1100
1050
1000
950
900
850
800
750
700
650
600
550
ppm

3f

9. Supporting Information References

[1] T. Alturaifi, G. Scofield, S. Wang, P. A. Liu, *ChemRxiv.* **2025**, *preprint, doi:10.26434/chemrxiv-2025-8nrh7.*

[2] S. Mehrparvar, Z. N. Scheller, C. Wölper, G. Haberhauer, *J. Am. Chem. Soc.* **2021**, *143*, 19856-19864.

[3] J. H. Griwatz, A. Kunz, H. A. Wegner, *Beilstein J. Org. Chem.* **2022**, *18*, 781-787.

[4] M. J. Frisch, G. W. Trucks, H. B. Schlegel, G. E. Scuseria, M. A. Robb, J. R. Cheeseman, G. Scalmani, V. Barone, G. A. Petersson, H. Nakatsuji, X. Li, M. Caricato, A. V. Marenich, J. Bloino, B. G. Janesko, R. Gomperts, B. Mennucci, H. P. Hratchian, J. V. Ortiz, A. F. Izmaylov, J. L. Sonnenberg, D. Williams-Young, F. Ding, F. Lipparini, F. Egidi, J. Goings, B. Peng, A. Petrone, T. Henderson, D. Ranasinghe, V. G. Zakrzewski, J. Gao, N. Rega, G. Zheng, W. Liang, M. Hada, M. Ehara, K. Toyota, R. Fukuda, J. Hasegawa, M. Ishida, T. Nakajima, Y. Honda, O. Kitao, H. Nakai, T. Vreven, K. Throssell, J. A. Montgomery, Jr.; J. E. Peralta, F. Ogliaro, M. J. Bearpark, J. J. Heyd, E. N. Brothers, K. N. Kudin, V. N. Staroverov, T. A. Keith, R. Kobayashi, J. Normand, K. Raghavachari, A. P. Rendell, J. C. Burant, S. S. Iyengar, J. Tomasi, M. Cossi, J. M. Millam, M. Klene, C. Adamo, R. Cammi, J. W. Ochterski, R. L. Martin, K. Morokuma, O. Farkas, J. B. Foresman, D. J. Fox, Gaussian, Inc., Wallingford CT, **2016**.

[5] F. Neese, *WIREs Comput. Mol. Sci.* **2022**, *12*, e1606.

[6] A. D. Becke, *Phys. Rev. A* **1988**, *38*, 3098-3100.

[7] C. Lee, W. Yang, R. G. Parr, *Phys. Rev. B* **1988**, *37*, 785-789.

[8] B. Miehlich, A. Savin, H. Stoll, H. Preuss, *Chem. Phys. Lett.* **1989**, *157*, 200-206.

[9] S. Grimme, S. Ehrlich, L. Goerigk, *J. Comp. Chem.* **2011**, *32*, 1456-1465.

[10] F. Weigend, R. Ahlrichs, *Phys. Chem. Chem. Phys.* **2005**, *7*, 3297-3305.

[11] B. P. Pritchard, D. Altarawy, B. Didier, T. D. Gibson, T. L. Windus, *J. Chem. Inf. Model.* **2019**, *59*, 4814-4820.

[12] C. Adamo, V. Barone, *J. Chem. Phys.* **1999**, *110*, 6158-6170.

[13] S. Grimme, J. Antony, S. Ehrlich, H. Krieg, *J. Chem. Phys.* **2010**, *132*, 154104.

[14] A. V. Marenich, C. J. Cramer, D. G. Truhlar, *J. Phys. Chem. B* **2009**, *113*, 6378-6396.

[15] A. Hellweg, C. Hättig, S. Höfener, W. Klopper, *Theor. Chem. Acc.* **2007**, *117*, 587-597.
